# High-resolution modeling of the selection on local mRNA folding strength in coding sequences across the tree of life

**DOI:** 10.1186/s13059-020-01971-y

**Published:** 2020-03-09

**Authors:** Michael Peeri, Tamir Tuller

**Affiliations:** 1 Department of Biomedical Engineering, Tel-Aviv University, Tel-Aviv, Israel; 2 Sagol School of Neuroscience, Tel-Aviv University, Tel-Aviv, Israel

**Keywords:** Protein-coding sequence evolution, mRNA secondary structure, Gene expression regulation, Comparative genomics, Codon usage

## Abstract

**Background:**

mRNA can form local secondary structure within the protein-coding sequence, and the strength of this structure is thought to influence gene expression regulation. Previous studies suggest that secondary structure strength may be maintained under selection, but the details of this phenomenon are not well understood.

**Results:**

We perform a comprehensive study of the selection on local mRNA folding strengths considering variation between species across the tree of life. We show for the first time that local folding strength selection tends to follow a conserved characteristic profile in most phyla, with selection for weak folding at the two ends of the coding region and for strong folding elsewhere in the coding sequence, with an additional peak of selection for strong folding located downstream of the start codon. The strength of this pattern varies between species and organism groups, and we highlight contradicting cases.

To better understand the underlying evolutionary process, we show that selection strengths in the different regions are strongly correlated, and report four factors which have a clear predictive effect on local mRNA folding selection within the coding sequence in different species.

**Conclusions:**

The correlations observed between selection for local secondary structure strength in the different regions and with the four genomic and environmental factors suggest that they are shaped by the same evolutionary process throughout the coding sequence, and might be maintained under direct selection related to optimization of gene expression and specifically translation regulation.

## Background

There is growing evidence that local mRNA folding (i.e., short-range secondary structure) inside the coding region is often stronger or weaker than expected, but the explanation for this phenomenon is yet to be fully understood. mRNA folding strength affects many central cellular processes, including the transcription rate and termination [1–3], translation initiation [4–14], translation elongation and ribosomal traffic jams [15–18], co-translational folding [19–21], mRNA aggregation [22], mRNA stability [23, 24], and mRNA splicing [10, 25] (reviewed in [26–28]). Many of these effects are mediated by interactions of mRNA within the CDS (protein-coding sequence) with proteins and other RNAs and may include structure-specific or non-structure-specific interactions.

**Fig. 1:**
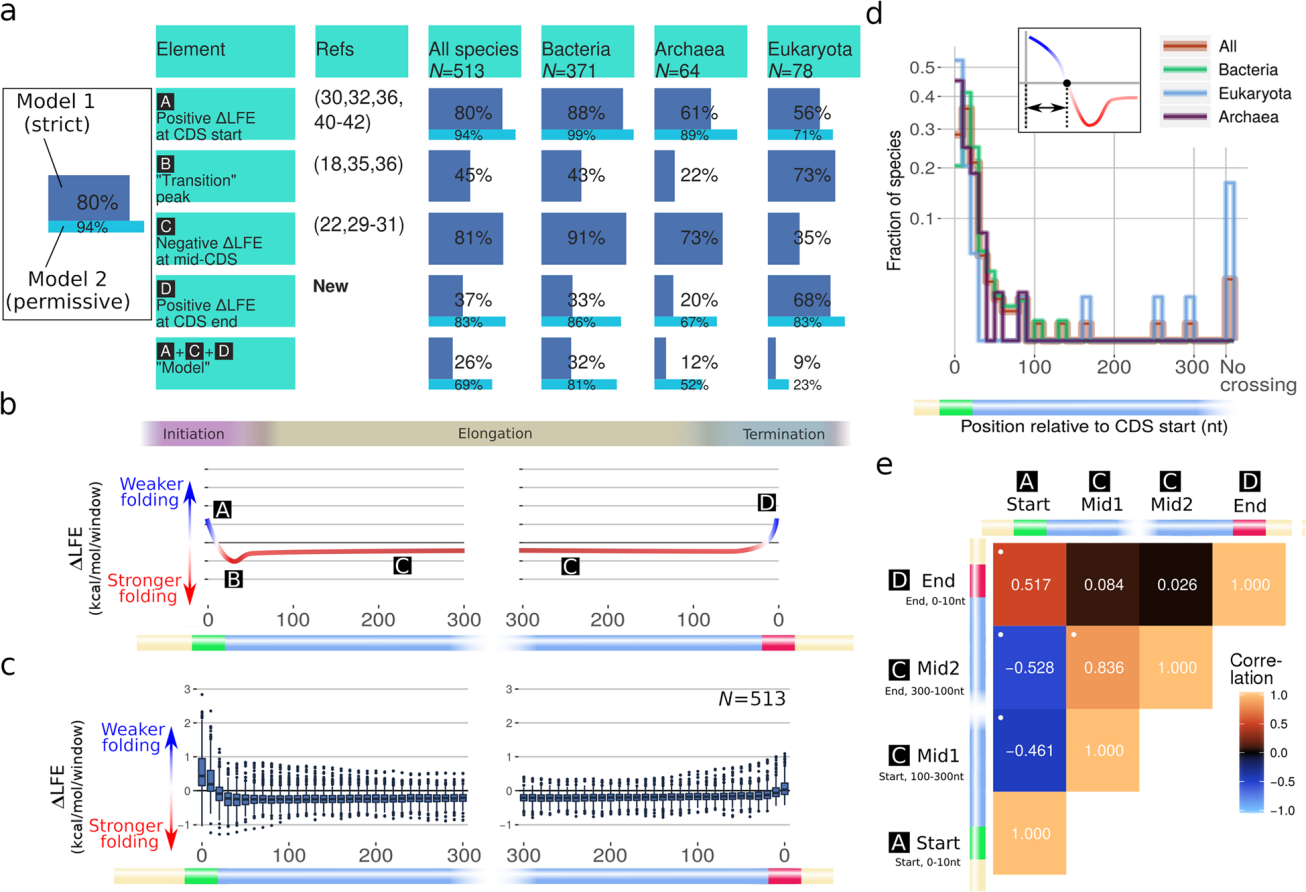
Common regions of folding bias (∆LFE) are present across the tree of life, but are not universal. There is correlation between the strengths of these regions in different species, indicating there are factors influencing the bias throughout the coding sequence. **a** Summary of profile features with the fraction of species in which each feature appears in each domain (based on model 1 rules; see “Analysis” under the “Methods” section for details). The results based on the less restrictive model 2 rules (with weaker ∆LFE near the CDS edges not required to be positive; see “Analysis” under the “Methods” section) are shown in bright blue below each bar. References shown here are based on comparison to randomized sequences (i.e., equivalent to ∆LFE). **b** Scheme illustrating profile features reported separately in previous studies within the CDS, showing features [A]–[D] from **a**. **c** Observed distribution of ∆LFE profile values at different positions relative to CDS start (left) and end (right). **d** The distances (in nt) from the start codon where ∆LFE transitions from positive to negative, for species belonging to different domain. The lengths of the initial weak folding region range up to 150 nt in some bacteria. **e** Spearman’s correlations between mean ∆LFE profile values in regions [A], [C], and [D]. White dots indicate significant correlation (*p* value < 0.01)

In recent years, several studies showed evidence for selection acting directly to affect mRNA folding strength within the CDS (Fig. 1a). Studies looking at the CDS as a whole found selection for strong mRNA folding in most species [22, 29–32]. Studies focusing on the beginning of the coding region (i.e., the first 40–50 nucleotides) found evidence for the inverse, with selection acting to weaken mRNA folding in that region [30, 32–34]. In addition, there is some evidence for specifically strong folding in nucleotides 30–70, which may slow down translation elongation near the 5′ end of the mRNA, possibly to prevent ribosomal traffic jams [18, 35, 36]. Finally, it has been suggested that folding is weakened in the region leading to the stop codon [32–34], but not in a way that attributes this weakening to direct selection on folding strength rather than a side effect of some other bias in this region. These results are generally in agreement with available small-scale (e.g., [13, 14]) and large-scale [10–12, 24, 37–39] experimental validation performed in model organisms. Some of these characteristic regions were found to be correlated with genomic GC-content and to be stronger in highly expressed genes [29, 36, 40–42]. However, the previous studies cited did not systematically examine how the selection on folding strength changes along the coding sequence and how this phenomenon varies across the tree of life. In this study, using high-resolution analysis of the folding selection profiles in over 500 organisms from the three domains of life, we examine all data under a common framework and under more stringent controls (including accounting for the evolutionary distances between species), to determine which correlations are likely to stem from causal relationships involved in maintaining mRNA folding. We show that the previously proposed patterns of local selection on mRNA folding are not universal and examine their association with genomic and environmental factors in different taxonomic groups to better understand the underlying evolutionary processes.

## Results

**Fig. 2:**
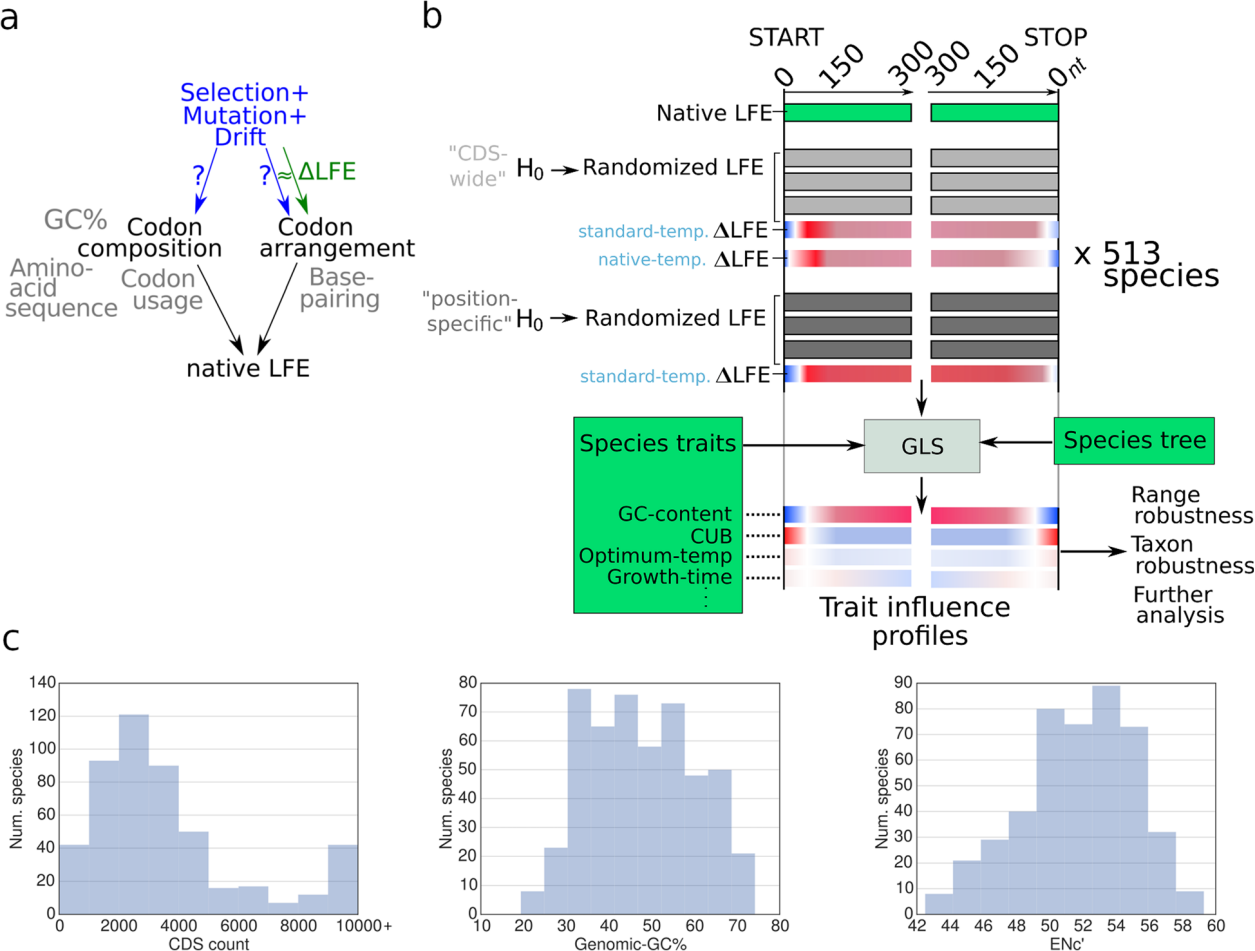
Overview of the computational analysis to measure ∆LFE while controlling for other factors known to be under selection at different regions of the coding sequence and find factors correlated with it. **a** The variables and concepts involved in determining local folding strength and calculating ∆LFE. The effects of the compositional factors on the left side are removed in order to specifically measure the contribution of codon arrangements to the native folding energy. Blue arrows indicate possible selection forces. **b** The different steps in the computational pipeline used to estimate ∆LFE and the factors affecting it (see “Analysis” under the “Methods” section). For each genome, the CDSs are randomized based on each null model (CDS-wide and position specific), to calculate a mean ∆LFE profile based on that null model. At the next step, based on GLS, correlations between features of the ∆LFE profile and genomic/environmental features are computed. Input data sources (native CDS sequences, species trait values, species tree) are shown in green. **c** The distributions of some genomic properties within the dataset—CDS count, genomic GC-content, and genomic ENc′ (measure of CUB). The dataset was designed to represent a wide range of values (among other considerations; see “Species selection and sequence filtering” under the “Methods” section)

To test different hypotheses related to direct selection acting on the local folding energy (LFE) in different regions of the coding sequence, we measured the mean deviation in LFE between the native and randomized sequences (maintaining the amino acid sequence of all CDSs as well as codon and nucleotide composition including the GC-content, see “Analysis” under the “Methods” section for more details). The resulting deviation values, denoted ∆LFE, measure the increase or decrease in local mRNA folding energy relative to what we expect based on the encoded protein and codon frequencies. Any significant deviation from random can be attributed to a specific arrangement of codons that supports increased or decreased base-pairing and folding strength along the mRNA strand (Fig. 2a).

Specifically, if the null hypothesis used to generate the randomized sequences holds for the native sequences at some position, we expect ∆LFE to be 0. Otherwise, a significant deviation from ∆LFE = 0 indicates that the local folding energy values cannot be explained by selection on amino acid content, codon bias, or GC-content alone and serves as evidence for direct selection on local folding energy (Fig. 2a). Positive ∆LFE indicates putative selection for weaker secondary structure, while negative ∆LFE corresponds with selection for stronger secondary structure. We specifically aimed at finding nearly universal patterns in ∆LFE, as well as groups of organisms and specific organisms with profiles deviating from such patterns. The resulting ∆LFE profiles were subsequently used with the evolutionary tree of the analyzed organisms to detect association between ∆LFE and genomic and environmental traits that cannot be explained by taxonomic relatedness alone and therefore may hint at underlying causal relations. We discuss the influence of genomic features such as codon usage bias (see the “Correlation between codon usage bias and ∆LFE” section) and GC-content (see the “Correlation between GC-content and ∆LFE” section), and of environmental features like intracellular life (see the “Weak ∆LFE in endosymbionts and intracellular organisms” section) and growth temperature (see the “Weak ∆LFE in hyperthermophiles” section).

### Conserved regions of folding bias (∆LFE)

**Fig. 3:**
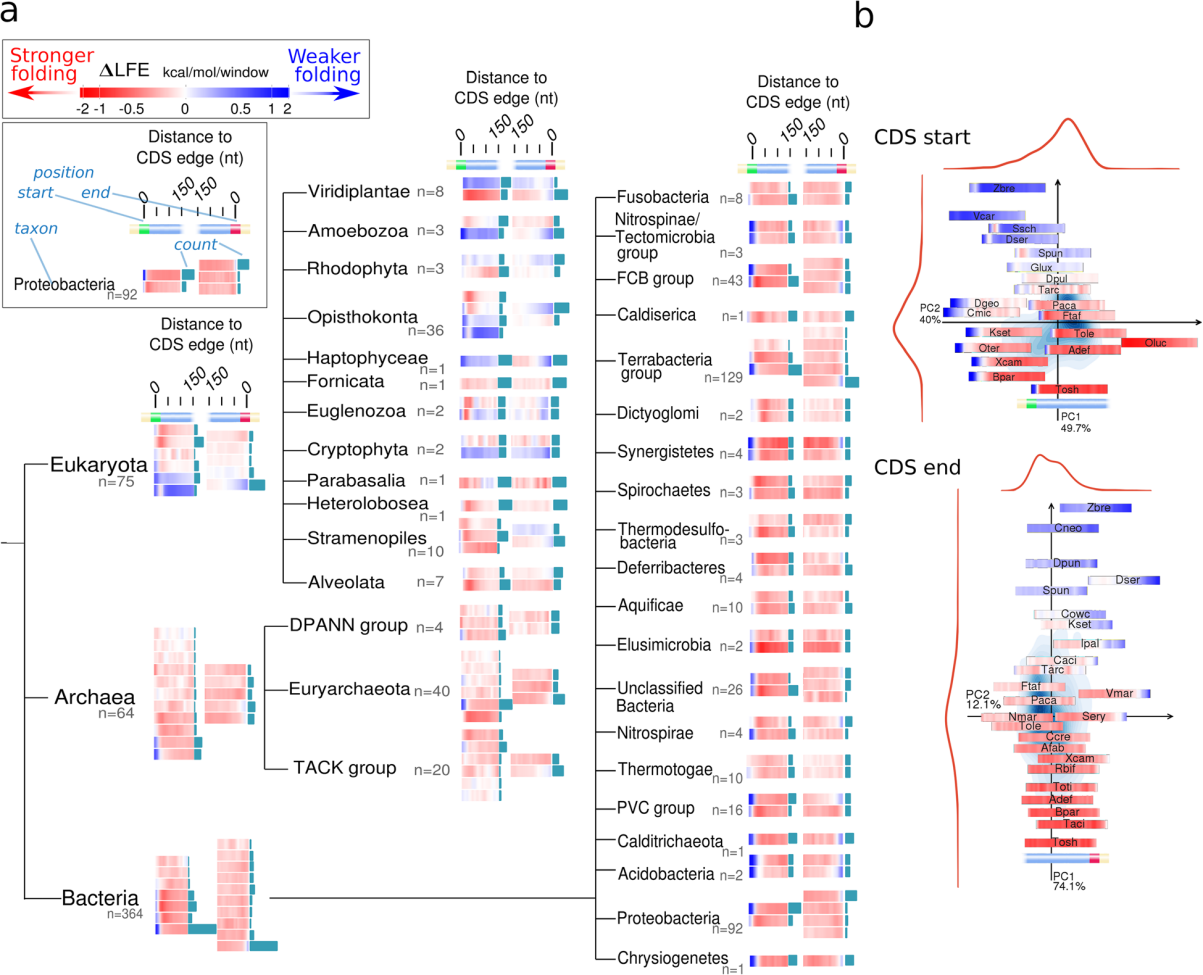
Two summaries of the ∆LFE profiles demonstrate the consistency and diversity found. **a** Characteristic ∆LFE profiles for species belonging to different taxons. The format of the plots appears in the upper left corner: ∆LFE bias is shown (by color) for windows starting in the range 0–150 nt relative to the CDS start, on the left, and CDS end, on the right; red denotes negative ∆LFE (stronger-than-expected folding) while blue denotes positive ∆LFE (weaker-than-expected folding; see the scale at the upper-right corner). The characteristic profiles for each taxon were calculated using clustering analysis, by grouping similar species according to the correlation between their profiles (see “Visualization” under the “Methods” section for details). The bars (in turquoise) appearing to the right of each characteristic profile indicate the relative number of species it represents. The full ∆LFE profiles for all species appear in Additional file 1: Figure S7. **b**. Summary of ∆LFE profile diversity for all species using dimensionality reduction to 2 dimensions with PCA (see explanations about PCA in the main text), with similar values (profiles) mapped to nearby positions. Background shading (blue) indicates density (see “Visualization” under the “Methods” section for details). This shows most species have similar profiles (located near the center), but different kinds of less typical profiles are also represented. Top, CDS start; bottom, CDS end. Short species names are listed in Additional file 1: Table S3

We observed that significant ∆LFE is present in most species and in most regions of the CDS (Fig. 3, Fig. 1a, c). The mean ∆LFE profiles of most species share the same structure (Fig. 3a, Fig. 1b, c), as follows. The region immediately following the CDS start (typically extending through the windows starting at positions 0–20 nt (Fig. 1a, region A), with a median of 20 nt/10 nt/20 nt in bacteria/archaea/eukaryotes, respectively) has positive mean ∆LFE (evidence of selection for weak folding), usually followed by a transition to negative mean ∆LFE (indicating selection for strong folding) within the first 50 nt and maintained throughout most of the CDS (Fig. 1a region C, Fig. 1c, d). The negative ∆LFE tends to weaken in the area immediately preceding the last codon (typically nucleotides 50–0 nt with median of 50/90/40 nt in bacteria/archaea/eukaryotes, respectively, Fig. 1d) in 83% of the species, and ∆LFE becomes positive there (indicating weaker-than-expected folding) in 37% of the species (including 68% of eukaryotes). This evidence of selection for weak mRNA folding near the stop codon in many organisms across the tree of life is reported here for the first time; two previous studies [18, 32] reported that the local folding energy (LFE) is weak near the start codon in three organisms and without showing that it cannot be explained by direct selection on the amino acid sequence (e.g., using computation of ∆LFE as was done here).

To measure how frequently these elements appear together within the same species, we tested them against a model, based on two variants. The stricter variant, model 1, counts species in which the regions of weak folding at the beginning and end of the CDS have, on average, weaker than expected folding, i.e., significantly positive ∆LFE. The less restrictive model 2 requires folding in these regions to be significantly weaker than in the middle of the CDS, but not necessarily significantly weaker than random (see “Analysis” under the “Methods” section for details). Since the models are applied to the mean ∆LFE of a population of genes which may vary greatly in their individual values, both estimates of the adherence to the model are informative. The combined models (composed of the three regions described) are found in 23% (model 1) and 69% (model 2) of the species analyzed (Fig. 1a), appearing very frequently in bacteria but also commonly in archaea and eukaryotes. The conservation of the ∆LFE profile structure in species across the tree of life is evidence of its biological significance.

GC-content and LFE both change during evolution, and it is worthwhile to compare their level of conservation in related species. LFE is to a large degree determined by GC-content (as evident by the almost perfect correlations found between GC-content and native or randomized LFE, Additional file 1: Figure S1), so one might argue the observed ∆LFE is a side effect of selection acting on GC-content. However, we found that the ∆LFE profile is more conserved than genomic GC-content at any phylogenetic distance within the same domain (Additional file 1: Figure S2). We also found that the profile does not consistently correlate with local variation in CUB (Additional file 1: Figure S3), demonstrating that the results reported here are not side effects of selection on codon bias (e.g., due to adaptation to the tRNA pool).

Additional tests also support direct selection acting to maintain folding strength. ∆LFE profile features are also preserved when calculated using a null distribution that maintains the codon distribution at any position in the CDS relative to the CDS start; thus, local (position-specific) genomic amino acid or codon distributions are not enough to explain the ∆LFE profile (Additional file 1: Figure S4). These features appear in many cases to be stronger in highly expressed genes, genes coding for highly abundant proteins, and genes with a strong codon adaptation to translation elongation, I_TE [43] (see Additional file 1: Figure S5). Finally, these results remain after controlling for the strength of the Shine-Dalgarno binding in the 5′-UTR [44] (Bahiri Elitzur S, Cohen-Kupiec R, Fine L, Yacobi D, Apt B, Diament A, et al.: Prokaryotic rRNA-mRNA interactions are involved in all translation steps and shape bacterial transcripts, Manuscript submitted for publication 2020) and for genes with short or overlapping 5′-UTRs (see, for example, [45]). Together, these results show that the ∆LFE profiles are unlikely to be explained as side effects of selection for a genomic or CDS position-dependent compositional bias in nucleotide, codon, or amino acids acting alone, although many such biases have been reported and are believed to have important biological effects [36].

Note that the randomized LFE profiles also are not always flat, revealing some residual influence on LFE, caused by the amino acid frequencies at different regions, remains even after randomization. ∆LFE controls for this by separately measuring the folding energy biases found in each position.

The different elements making up the model profile structure have functions associated with them. The weak folding region at the beginning of the coding region may improve access to the regulatory signals in this region (e.g., the start codon) [5, 36]. The region of positive ∆LFE preceding the CDS end may help recognition of the stop codon and ribosomal dissociation from the mRNA and prevent ribosomal read-trough. Strong folding in the middle of the coding sequence may assist co-translational folding [19–21] by slowing down translation in specific positions to allow protein folding or other co-translational processes to take place, as well as regulate mRNA stability [23] or prevent mRNA aggregation [22].

The division of the profile into the three regions described here is also apparent when the data is analyzed in an unsupervised manner via principal component analysis (PCA) [46] (Fig. 3b and Additional file 1: Figure S6). This arranges species on a two-dimensional plane according to their ∆LFE profiles, so species with more similar ∆LFE profiles are placed closer together. The resulting plots (for the beginning and end of the coding sequence) show the majority of species have similar ∆LFE profiles (located very close to each other near the center of the plot), with positive ∆LFE near the ends of the coding sequence and negative ∆LFE in the middle of the coding sequence. Groups of species containing other types of profiles are arranged around them on the plots. At either end of the coding sequence, 2 variables (principal components) are sufficient to describe at least 85% of the variability between all ∆LFE profiles, supporting the division of the ∆LFE into three regions (since the mid-CDS region appears in both analyses, see Fig. 1e).

In 45% of the organisms, we found an additional feature: a peak of selection for strong mRNA folding around 30–70 nt downstream of the start codon (Fig. 1a, region B). It was suggested ([34, 35], based solely on evidence in *Eschericia coli* and *Saccharomyces cerevisiae*) that this peak is responsible for increasing translation throughput, by minimizing ribosomal traffic jams occurring because of uneven translation elongation rates throughout the CDS. There is also some evidence [4, 9] that strong secondary structure downstream of the start codon can enhance translation. Whatever the mechanism responsible for it, the results here show that this feature is common across the tree of life. This feature was also shown previously to be stronger in highly expressed genes in 3 species [45], and our results extend this claim (see Additional file 1: Figure S5).

The ∆LFE profiles of eukaryotes are much more diverse than those found in prokaryotes. One striking observation is that significant *positive* ∆LFE throughout the mid-CDS region, present in 13% of the eukaryotes tested, is not observed in any of the 371 bacterial species tested except in *Deinococcus puniceus* (Additional file1: Figure S8, see also Fig. 1a). This seemingly universal rule hints at a constraint on bacterial CDSs not obeyed in eukaryotes and is one of two major differences observed between the domains (along with the correlation with genomic-GC, see the “Correlation between GC-content and ∆LFE” section).

Despite these general trends, there is also significant variation in the ∆LFE profiles across and within taxonomic groups. In the subsequent sections, we discuss genomic and environmental factors that explain some of the variation between mean ∆LFE profiles in different species.

### Correlations between ∆LFE regions

The strengths of the three major regions of the ∆LFE profile described above are strongly correlated (Fig. 1e): organisms with relatively stronger ∆LFE (in absolute value) in one model region appear to also have stronger ∆LFE in other regions. For example, the 0–20-nt region has a strong negative correlation with the 150–300-nt region (Spearman’s *ρ* = − 0.46; *p* value < 1e−8). This correlation remains highly significant for different ranges and when testing using GLS (Additional file 1: Fig. S9). The two mid-CDS regions (relative to CDS start and end) are positively correlated (*ρ* = 0.84, *p* value < 1e−8), as are the CDS start and end regions (*ρ* = 0.52, *p* value < 1e−8). These correlations indicate ∆LFE profiles of different species can generally be ordered by magnitude from species having strong (positive or negative) ∆LFE features *throughout* the CDS to those showing weak or no ∆LFE. In eukaryotes, the negative correlation between the CDS start and mid-CDS regions is not present (results not shown), but in this case, neither do the ∆LFE profiles generally follow the structure of positive start ∆LFE and negative mid-CDS ∆LFE and the profile values may continue to change farther away from the CDS edges.

Together, these results suggest that the different elements making up the typical profile structure are influenced at the genome level by a factor or combination of factors acting jointly on all regions and strengthening or weakening |∆LFE|, as well as distinct factors acting on each region differently. Some factors contributing to this scaling effect are discussed in the following sections.

### Correlation between codon usage bias and ∆LFE

**Fig. 4:**
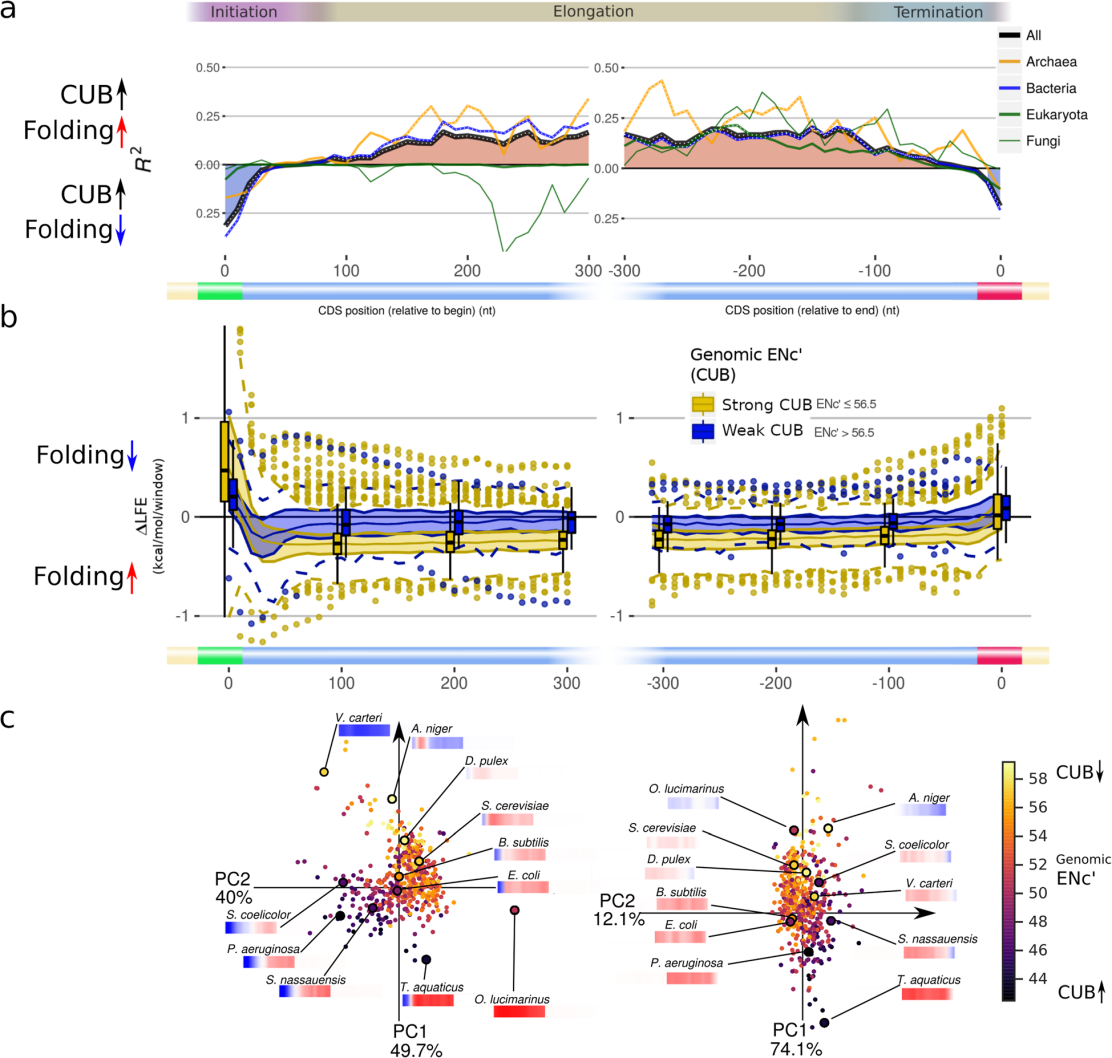
Folding bias (∆LFE) is positively correlated with genomic CUB (measured as ENc′) throughout the CDS. This correlation indicates stronger folding bias exists in species with stronger CUB at all regions of the CDS. **a** Correlation strength (*R*^2^, measured using GLS regression) between genomic ENc′ and ∆LFE at different positions relative to the CDS start (left) and end (right). *R*^2^ values below the *X*-axis indicate negative regression slope (i.e., negative correlation with ∆LFE). The regression slope generally has the opposite sign as ∆LFE, indicating strong ∆LFE is correlated with strong codon bias throughout the CDS. Major taxonomic groups are plotted as different colored lines. White dots indicate regression *p* value < 0.01. **b** Comparison of ∆LFE profile values in species with strong vs. weak CUB. Species with strong CUB (yellow, ENc′ ≤ 56.5) tend to have more extreme ∆LFE and show the conserved ∆LFE regions more clearly, while species with weak CUB (blue, ENc′ > 56.6) tend to also have weak ∆LFE. **c** Genomic ENc′ values plotted using coordinates determined by ∆LFE profiles. Species with strong CUB (left plot, lower left quadrant and right plot, right side) have stronger ∆LFE profiles that more strongly adhere to the conserved ∆LFE regions. Coordinates are based on PCA for profile positions 0–300 nt relative to CDS start (left) and end (right). The PCA coordinates are the same as those in Fig. 3b

Codon usage bias is generally correlated with adaptation to translation efficiency [47–50]. If ∆LFE is also related to selection for translation efficiency, it is reasonable to expect it would correlate with CUB. To test this hypothesis, we used ENc′ (ENc prime, [51, 52]), a measure of codon usage bias (CUB) that compensates for the influence of extreme GC-content values that skew standard ENc (effective number of codons) scores. Indeed, such a correlation is found (Fig. 4, Additional file 1: Figure S10b)—∆LFE tends to be stronger (in absolute value) in species having strong CUB (low ENc′), and this holds both near the CDS edges and in the mid-CDS regions. Similar results were obtained when using other measures of CUB (CAI [53] and DCBS [49], Additional file 1: Figure S11), and these correlations persist within many individual taxons (Fig. 9, Additional file 1: Figure S10b). In addition, species with strong CUB tend to have ∆LFE profiles that closely match the model elements (Fig. 4b, c), and further analysis shows the correlation of CUB with the ∆LFE profiles is due to correlation with the magnitude of the profiles and not due to specific profile regions (Additional file 1: Figure S12). Since ∆LFE is computed while controlling for the CUB of each sequence, the reported results suggest that organisms with higher selection on CUB also have, “independently” from a statistical point of view, higher selection on ∆LFE.

Using genomic CUB as measure of optimization for efficient translation elongation, we found that it is also a good predictor of the strength of ∆LFE. One interpretation of this is that the genomic variation in ∆LFE can largely be explained not by different species having distinct “target” ∆LFE levels, but by different species having varying “abilities” to maintain ∆LFE in the presence of mutations and drift because the selection pressure is insufficient under their effective population size (either because the selection pressure is low or because the effective population size is low).

### Correlation between GC-content and ∆LFE

GC-content is a fundamental genomic feature and is correlated with many other genomic traits and environmental aspects [54, 55]. It might be a trait maintained under direct selection, or merely a statistical measure of the genome that other traits evolve in response to because of its biological and thermodynamic consequences. GC-content is also the strongest factor determining the native LFE (Additional file 1: Figure S1a), since G-C base pairs are more stable than A-T pairs (due to the increase in the number of hydrogen bonds and more stable base stacking). Selection on folding strength (measured by ∆LFE) also influences folding strength, and we would like to measure the correlation between these two factors that influence the folding strength (namely, GC-content and ∆LFE). This is made possible since ∆LFE is calculated relative to the baseline maintaining the GC-content of the original coding regions in the randomized ones (see “Randomization procedures” under the “Methods” section for a description of the null models). This controls for the direct effect of GC-content, allowing us to directly study the interaction between ∆LFE and GC-content (see also Additional file 1: Figure S1a).

**Fig. 5:**
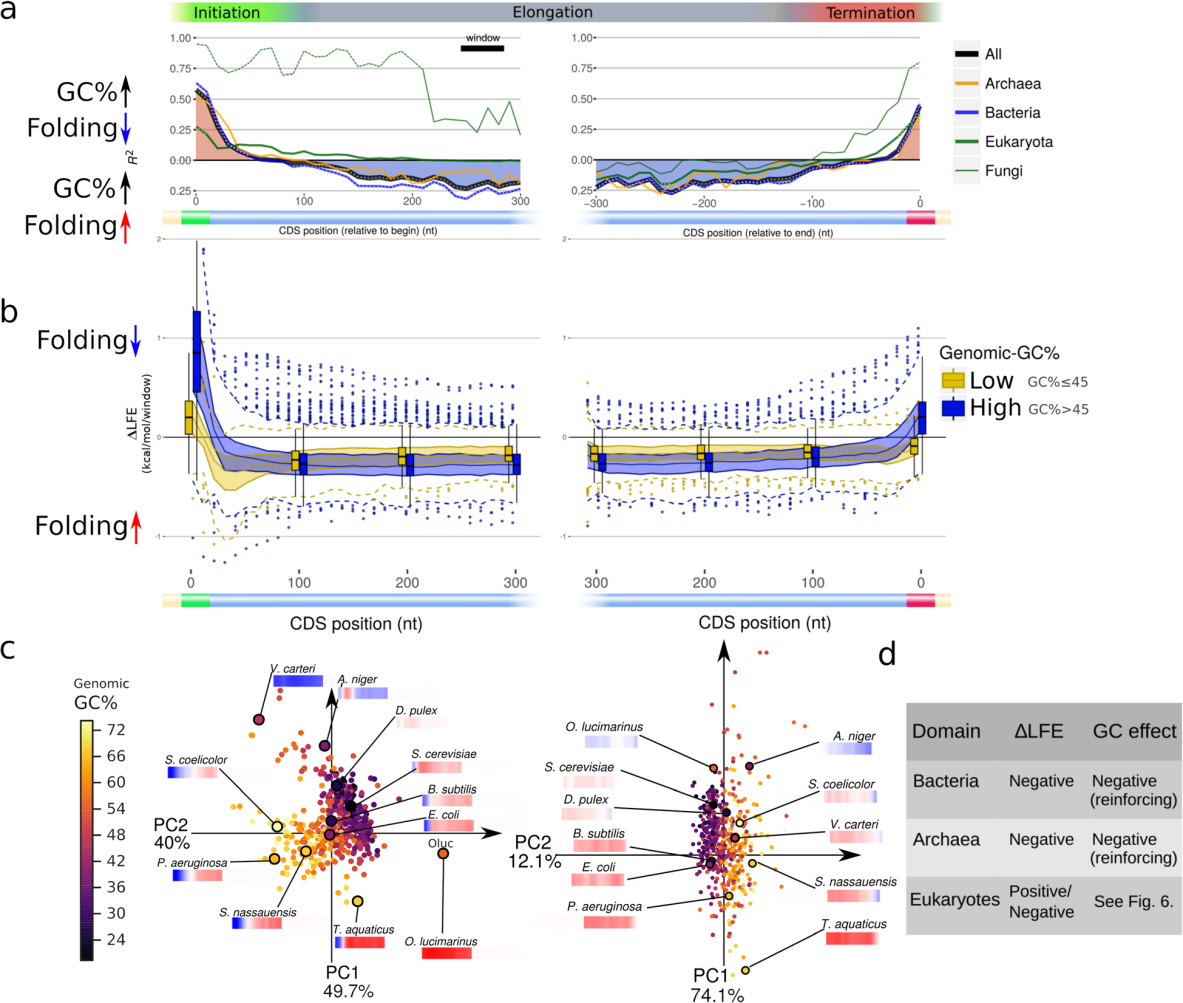
Folding bias (∆LFE) is positively correlated with genomic GC-content throughout the CDS. **a** The effect of genomic-GC on ∆LFE at each position along the CDS start (left) and end (right), measured using GLS regression *R*^2^ values. *R*^2^ values above the *X*-axis indicate positive regression slope (indicating moderating effect of GC-content); *R*^2^ values below the *X*-axis indicate negative regression slope (i.e., reinforcing effect of GC-content). Near the CDS edges (where ∆LFE is usually positive), genomic-GC generally has a moderating effect on ∆LFE. In the mid-CDS region (where ∆LFE is usually negative), genomic-GC generally has a reinforcing effect on ∆LFE. Major taxonomic groups are plotted as different colored lines. White dots indicate regression *p* value < 0.01. **b** Comparison of ∆LFE profile values in species with high vs. low genomic GC-content. Species with high GC-content (blue, genomic-GC > 45%) tend to have more extreme ∆LFE and show the conserved ∆LFE regions more clearly, while species with low GC-content (yellow, genomic-GC ≤ 45%) tend to also have weak ∆LFE. **c**. Genomic GC-content for all species plotted on the PCA coordinates of their ∆LFE profiles (same coordinates as in Fig. 3b. *N* = 513) for CDS start (left) and end (right). Low-GC species are generally clustered in a small region, indicating they have similar ∆LFE profiles, and that region is characterized by weak ∆LFE. **d** Qualitative summary of ∆LFE in relation to GC-content in the mid-CDS

The correlations (expressed as *R*^2^) between genomic GC-content and ∆LFE at different points near the CDS start and end are shown in Fig. 5a. This dependence shows a similar pattern to that seen in the ∆LFE profiles themselves (Fig. 1c, Fig. 5a, and for the correlation with CUB, see the “Correlation between codon usage bias and ∆LFE” section), with significant correlations appearing in roughly the same CDS regions described for the ∆LFE profiles. The correlation takes the opposite directions in the CDS edges than that maintained throughout the inner CDS region, which means GC-content is positively correlated with the strength of ∆LFE (in absolute value) throughout the CDS (like CUB is).

Near the CDS start, positive correlation (indicating a moderating effect) exists in the windows starting at 0–60 nt (Fig. 5a, Additional file 1: Figure S10a). This effect appears in almost all taxons analyzed, with *R*^2^ values between 0.2 and 0.9 and significant *p* values in most taxons, and may be explained as counteracting the strengthening influence of GC-content on secondary structures to prevent them from hindering the translation initiation process.

The opposite effect exists in the mid-CDS: negative (reinforcing) dependence on genomic GC-content appears in the region at 70–300 nt after CDS start in most bacterial and archaeal taxons (Fig. 5a–c, Fig. 9, Additional file 1: Figure S10a) and is generally maintained throughout the length of the CDS (excluding the edge regions). As mentioned above, selection for strong mRNA folding and mRNA structures inside the coding may be related to transcription elongation [2], co-translational folding [19–21, 26], and mRNA stability [23]. The observed ∆LFE in this region is indeed negative in nearly all bacterial and archaeal species; it is possible that the folding is further reinforced in species higher GC-content since they are under stronger selection for these processes. Note that the effects of genomic GC-content and CUB (see the “Correlation between codon usage bias and ∆LFE” section) are somewhat overlapping, but each factor significantly contributes to the total observed effect (Additional file 1: Figure S13).

**Fig. 6:**
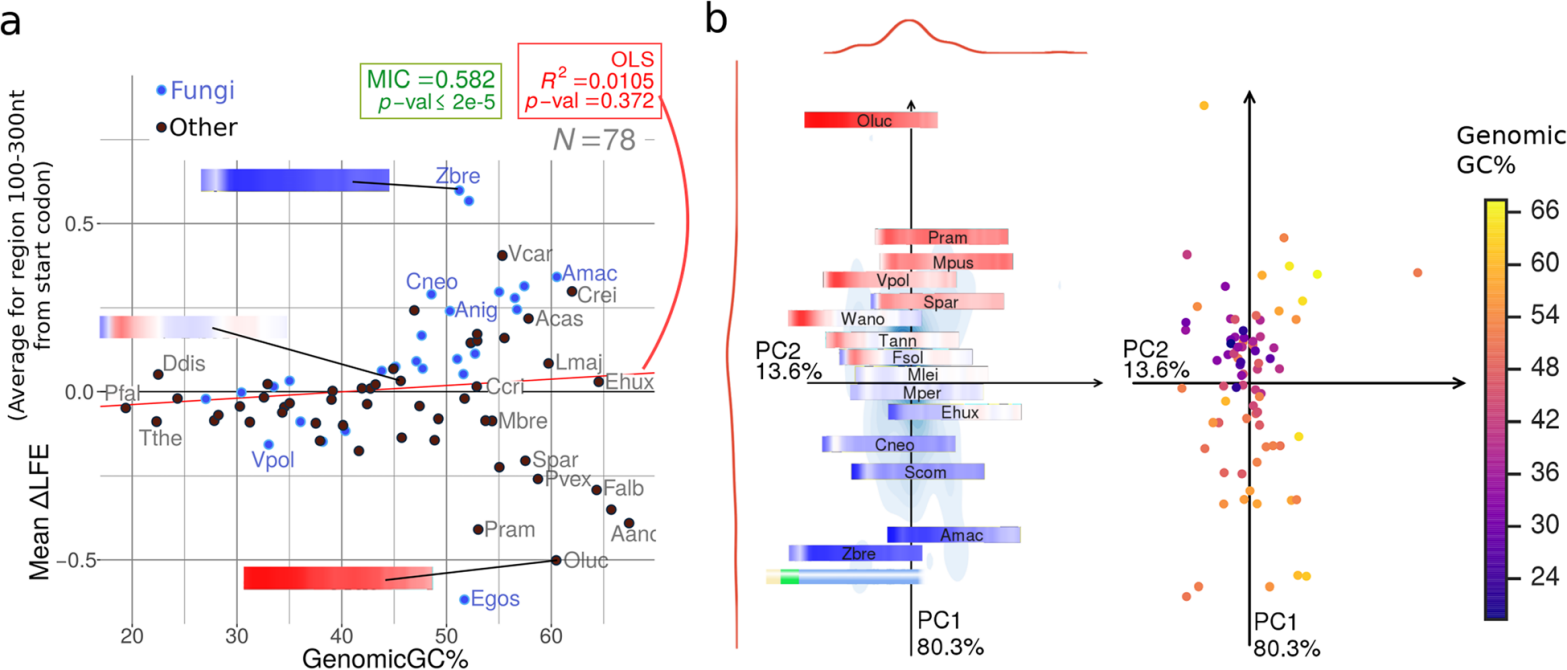
Genomic-GC effect on ∆LFE in eukaryotes shows divergence in high GC-content species that is not observed in other domains, while low GC-content species have weak ∆LFE. **a** mean ∆LFE values for eukaryotes in the range 100–300 nt from CDS start, plotted against genomic-GC. Fungi are highlighted in blue. There is no linear relation between the variables (*R*^2^ = 0.01), but there is strong statistical dependence nevertheless (MIC = 0.582, *p* value < 2e−5, *N* = 78); see some explanation on MIC in the main text. **b** PCA plot for the same species shows the same two classes of ∆LFE profiles in eukaryotes having high genomic-GC (top and bottom regions; see “Visualization” under the “Methods” section for details). On the left, ∆LFE profiles are plotted in the positions given by their first 2 PCA components. On the right, genomic-GC values for the profiles plotted at the same coordinates. Short species names are listed in Additional file 1: Table S3

In eukaryotes, we observed a wider variation in mid-CDS ∆LFEs (which is not found in other groups), from strongly positive to strongly negative, with a non-linear dependence on genomic-GC (Fig. 6, Fig. 9). Low-GC eukaryotes tend to have weak ∆LFE in the mid-CDS region, while high-GC eukaryotes tend to have strong *positive* or *negative* ∆LFE in the same region. To evaluate this relation, which is not linear, we used maximal information coefficient (MIC) [56, 57], a measure that can capture any statistical dependence including non-linear dependencies. We found that this relation is quite significant (MIC = 0.54, *p* value ≤ 2e−5; see “Analysis” under the “Methods” section). Fungi, however, show a strong positive (moderating) correlation between genomic-GC and ∆LFE (Fig. 5a, Fig. 6a; *Eremothecium gossypii*, GC% = 51.7, is the only observed fungus with GC% > 45 and negative ∆LFE in the mid-CDS region). There are also clear internal disparities in ∆LFE among fungi families (Additional file 1: Figure S7). Note that in some species (e.g., *Zymoseptoria tritici*), the positive ∆LFE seems to extend throughout the CDS. In other species, there is a transition to negative ∆LFE further downstream (as much as 500 nt from CDS start, results not shown).

The group of fungi and other eukaryotes having strong selection for weak local mRNA folding in the mid-CDS region (all of which have high genomic GC-content) runs counter to the general trend in prokaryotes. It is possible that these species are under selection for higher translation elongation speeds, which tend to be hindered by stronger mRNA folding [15–18]; however, it is not clear why such cases are not observed in other groups like bacteria. The correlation with GC-content reported here may also be partially explained by the fact that both GC-content and ∆LFE are affected by common factors such as the ability to maintain the selected sequences under the effective population size. The wide range of ∆LFE values for eukaryotic species and the absence of linear correlation with GC-content (in general) reveal additional factors are involved in this aspect of gene expression.

### Weak ∆LFE in endosymbionts and intracellular organisms

**Fig. 7:**
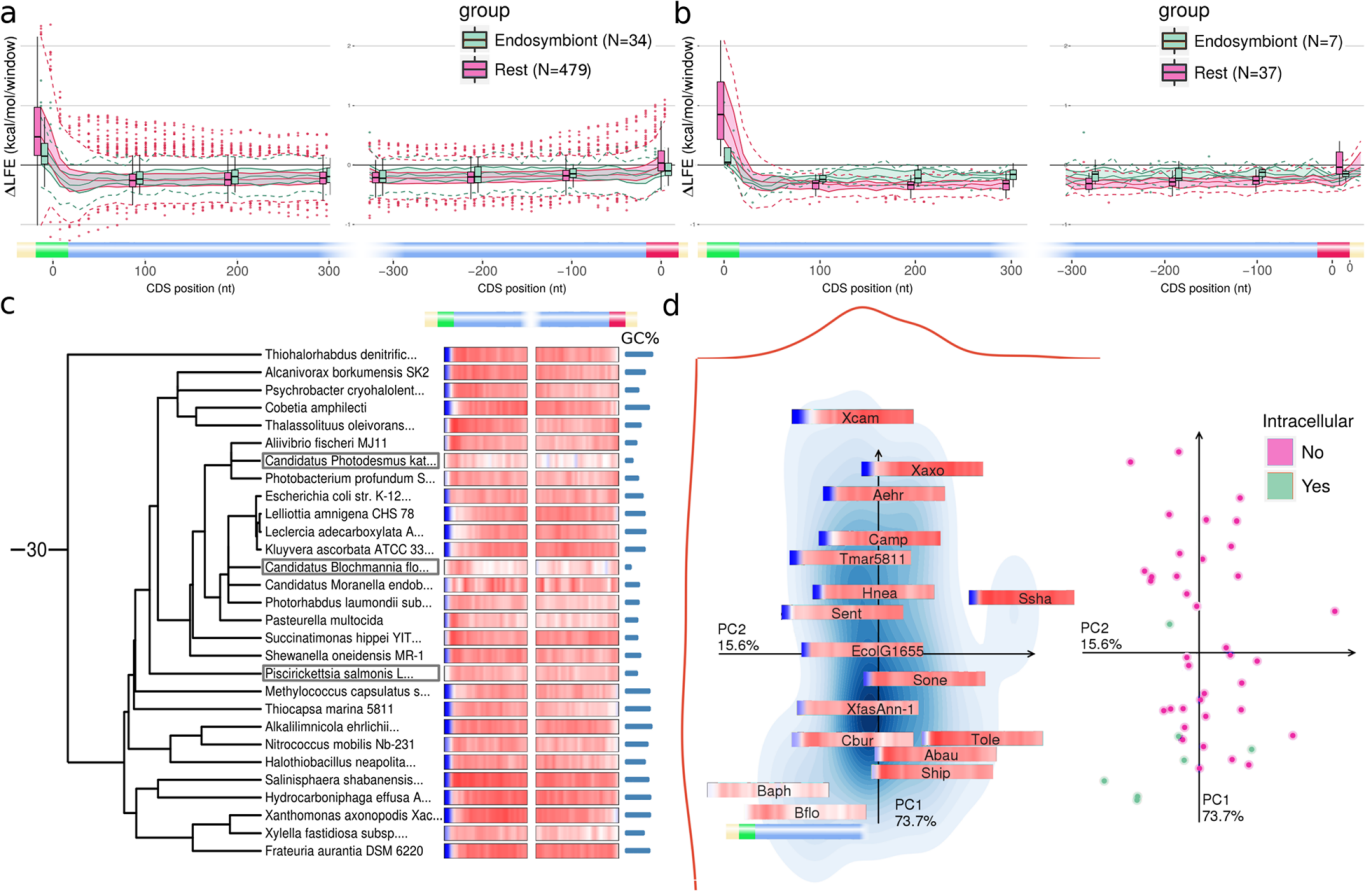
Endosymbionts and other intracellular species have generally weak ∆LFE. **a** Comparison of ∆LFE values at different CDS positions between endosymbionts (green) and other species (pink). The ∆LFE values are less extreme in endosymbionts, indicating lower selection on local folding strength. **b** Comparison of ∆LFE distributions at different CDS positions between endosymbionts (green) and other species (pink) within gammaproteobacteria (*N* = 44). **c** ∆LFE for species included in the tree within gammaproteobacteria; the endosymbionts and intracellular species (marked) have weaker ∆LFE bias compared to their relatives. **d** PCA plot for ∆LFE profiles (left, see “Visualization” under the “Methods” section) and the intracellular classification (right) for the species in gammaproteobacteria (*N* = 44). For clarity, overlapping profiles are hidden on the left (as in all PCA plots for ∆LFE profiles); all species are plotted on the right. Short species names in the PCA plot on the left panel are listed in Additional file 1: Table S3

Many endosymbionts and other species with intracellular life stages have low effective population sizes, because their life cycle includes recurring population bottlenecks [58, 59] or has lower selective pressure due to reliance on the host [60]. These species generally have weaker ∆LFE compared to their relatives, as can be clearly seen from their ∆LFE profiles (Fig. 7, also see Additional file 1: Figure S7, e.g., *Richelia intracellularis*, *Blattabacterium* sp.). The apparent disparity between endosymbionts and their relatives is strongest near the CDS start. Taken as a whole, the difference in ∆LFE is small (Fig. 7a), but when comparing within smaller taxons, the difference is much more noticeable (e.g., gammaproteobacteria in Fig. 7b–d). Endosymbionts also tend to have lower GC-content and CUB [60], but the results are still generally significant after considering this at least in proteobacteria, where we have a sufficient sample size (Additional file 1: Figure S14). The dichotomic grouping of species as endosymbionts is an oversimplification and ignores the variety of species with intracellular stages, including obligate and facultative intracellular parasites (and our annotation of species as endosymbionts, based on the literature, may not be complete). Indeed, some species we classify as endosymbionts (e.g., *Halobacteriovorax marinus SJ*) nevertheless have low genomic ENc′ and strong ∆LFE.

### Weak ∆LFE in hyperthermophiles

**Fig. 8:**
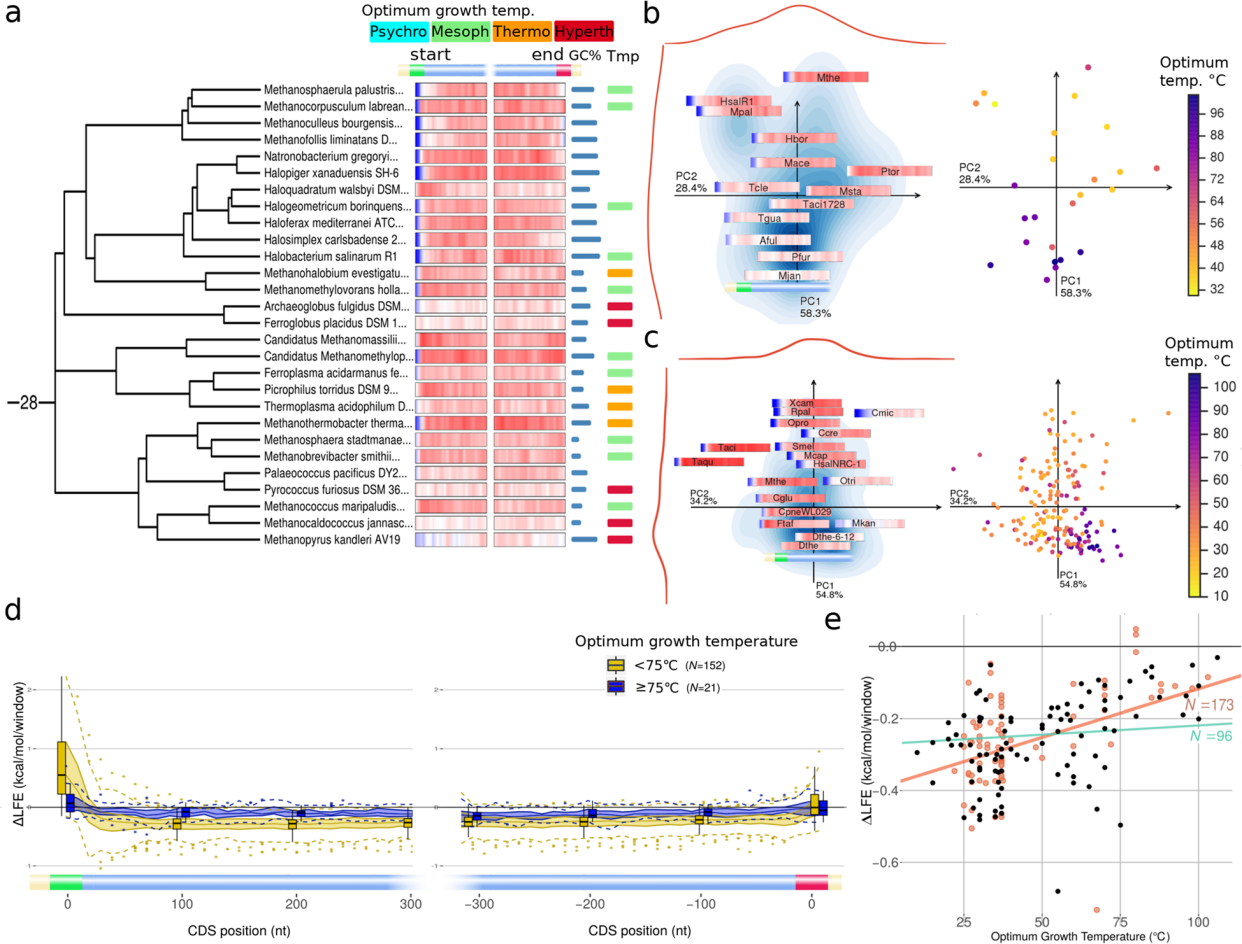
Hyperthermophiles have weak ∆LFE. **a** ∆LFE profiles (for CDS beginning and end) for members of euryarchaeota covered by the phylogenetic tree (*N* = 28) and their annotated optimum growth temperature classification (mesophile—green, moderate thermophile—orange, hyperthermophile—red) and genomic GC-contents. Hyperthermophiles have weak ∆LFE that cannot be explained by the tree topology or their genomic GC-contents. **b** ∆LFE profiles (left) and optimum growth temperatures (right) for all members of euryarchaeota having annotated optimum growth temperatures (*N* = 25), plotted using their PCA coordinates (see “Visualization” under the “Methods” section). Hyperthermophiles seem to be clustered in a small region characterized by weak ∆LFE. **c** ∆LFE profiles (left) and optimum growth temperature (right) for all species having annotated optimum growth temperature (*N* = 173), plotted using their PCA coordinates (see “Visualization” under the “Methods” section). Short species names from PCA plots are listed in Additional file 1: Table S3. **d** Comparison of ∆LFE values for species having optimum temperature above (blue) or below 75 °C (yellow), for positions relative to CDS start (left) or end (right). **e** Regression for optimum growth temperature vs. mean ∆LFE (average for positions 100–300 nt after CDS start) using GLS (green regression line, *N* = 96, *R*^2^ = 0.004, *p* value = 0.6) and OLS (red regression line, *N* = 173, *R*^2^ = 0.45). The apparent linear relation is no longer significant when controlling for the phylogenetic relationships. Points plotted in red are included only in OLS

**Fig. 9:**
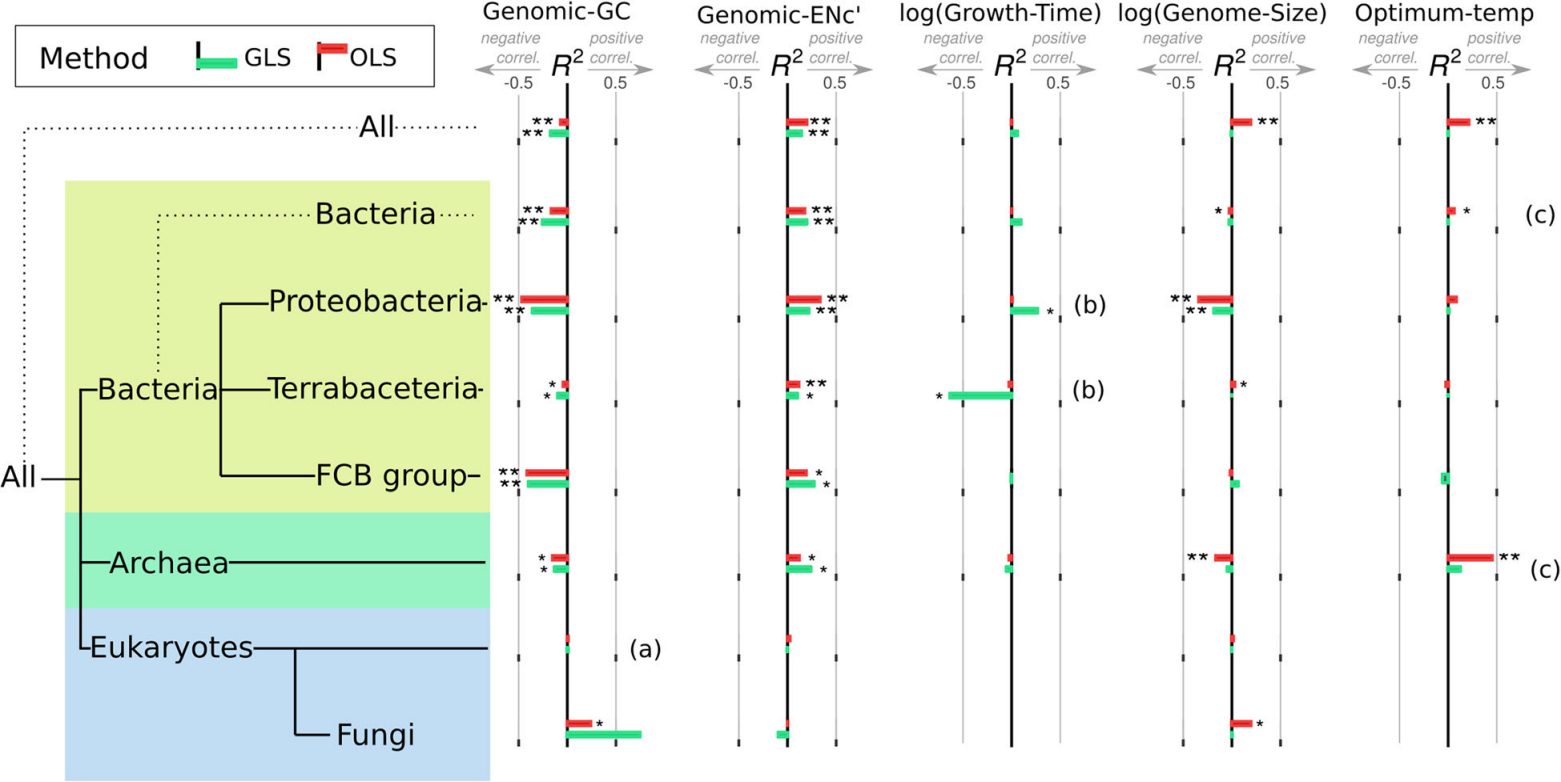
Summary of trait correlations with ∆LFE in the mid-CDS region for different taxonomic groups. Many of these correlations are discussed in the previous sections. For each taxonomic group and trait combination, correlations are measured using *R*^2^ with GLS (phylogenetically corrected, green bars) and OLS (uncorrected linear relationship, red bars). Significant correlations are marked with “∗” (*p* value < 0.05) or “∗∗” (*p* value< 0.001). Correlations with genomic-GC% and genomic-ENc′ are robust in prokaryotes, whereas other traits do not have consistent linear relationships. All correlations are for the region 100–300 nt after CDS start. (a) No linear dependence, but a significant relationship does exist (see Fig. 6). (b) Linear dependence appears in GLS but not in OLS. Small sample size exists in some taxons. (c) No significant linear relationship found over the entire range of values, but hyperthermophiles have significantly lower ∆LFE (see the “Weak ∆LFE in hyperthermophiles” section)

In temperatures approaching the RNA melting temperature, base-pairing is destabilized and it is likely that codon arrangement and ∆LFE can no longer significantly affect the secondary structure. We found hyperthermophilic archaea and bacteria to have weaker (closer to 0) ∆LFE in the mid-CDS region (Fig. 8). This effect is not apparent at lower temperatures (below 65 °C) or across all temperatures, with temperature having no significant correlation with ∆LFE (Fig. 8e, Fig. 9) when controlling for species relatedness. Our results are consistent with [40], which argued for negative correlation with growth temperature, but that paper only analyzed the beginning of the coding region and did not control for the evolutionary relations among organisms. Based on our analysis, the linear relation between temperature and ∆LFE is not generally supported by GLS (Fig. 8e, Fig. 9, Additional file 1: Figure S10c); however, since species tend to have similar temperature requirements as their close relatives, it is hard to conclusively decide if any similarity in ∆LFE is derived from association with temperature or the evolutionary relationship without having considerably more data. In hyperthermophiles (species with optimum growth temperature above 75 °C), however, there is a significant decrease in ∆LFE (even when the folding strengths are predicted at room temperature, Additional file 1: Figure S15). These results suggest that mRNA folding is not effective in higher temperatures (in general), and consequently, ∆LFE is not preserved. In moderate thermophiles, ∆LFE may follow the precedence of genomic GC-content, which previous studied concluded is not an adaptation to high temperatures at the genomic level, but may still be part of such an adaptation at specific rRNA and tRNA sites where secondary RNA structure is particularly important [61, 62].

## Discussion

The results we presented here provide a wide integrated view on the way evolution shapes local mRNA secondary structures in the coding regions of organism across the tree of life. In addition, the results include novel attempts to tie this phenomenon to genomic, evolutionary, and environmental variables in the hope of further clarifying the processes involved. In this section, we will summarize and discuss key results.

First, we show that selection on mRNA folding strength in most (but not all) species follows a conserved structure with three distinct regions (Fig. 1)—decreased local folding strength at the beginning and end of the coding region and increased folding strength in mid-CDS. The fact that this structure is more conserved than other genomic traits like GC-content (Additional file 1: Figure S2), as well as its alignment to the coding regions, suggests these features are related, at least in part, to translation regulation. Our statistical tests demonstrate that these features cannot be merely side effects of factors known to be under selection like codon usage bias and amino acid composition.

In general, the model features for the beginning and mid-CDS appear much more frequently in the analyzed organisms (appearing in around 80% of the organisms), while selection for weak folding near the stop codon, first demonstrated here, is comparatively rare (it appears in around 37% of the organisms). This may suggest that generally, the first two features tend to be under stronger selection (possibly since they tend to contribute more significantly to organism fitness).

Conformance to different model elements varies significantly between the three domains: weak folding at the beginning of the coding regions appears in the great majority of bacterial species (88%) but only in 56%/60% of eukaryotes/archaea, respectively (Fig. 1a, Fig. 3a). These differences may be related to polycistronic gene expression (see Additional file 1: Figure S16) or to generally higher effective population sizes and selection for high growth rate in bacteria; they may also indicate complementary constraints imposed by eukaryotic gene expression mechanisms (e.g., Cap-dependent translation initiation) and unique environmental constrains in archaea. On the other hand, selection for weak mRNA folding at the end of coding region (first conclusively shown here) is much more frequent in eukaryotes (appearing in 68% of the analyzed organism) than in the prokaryotes (20% in archaea and 33% in bacteria). This may be related to alternative mechanisms for efficient translation termination fidelity in prokaryotes (including mRNA folding outside the boundaries of the CDS) and/or to translation of polycistronic transcripts (see [63] for related observations in the 3′-UTR).

Second, we found that in some eukaryotes (in 13% of the analyzed eukaryotes and in one bacteria: *D. puniceus*), there is significant *positive* ∆LFE throughout the mid-CDS region (i.e., opposite to the general trend in prokaryotes, Fig. 1a, Fig. 6, Additional file 1: Figure S8). This phenomenon, more widespread than previously reported, may be related to selection improving elongation speed [18]. It is currently not clear why this type of selection appears only in these eukaryotes and is extremely rare in the other domains.

Third, we show that the “transition peak,” a region of selection for strong mRNA folding beginning around 30–70 nt downstream of the start codon that was reported elsewhere to be associated with translation efficiency [18, 35, 36, 45], appears frequently (45%) in the analyzed organisms, indicating this mechanism is common (Fig. 1a, c). This feature appears much more frequently in eukaryotes (73%) than in prokaryotes (22% in archaea and 43% in bacteria). Here, too, it is possible the lower frequency in prokaryotes hints at a complementary mechanism for translation initiation and elongation efficiency and fidelity in prokaryotes.

Forth, despite these differences, we found strong correlation between the strengths of three profile elements (found at the beginning, middle, and end of the coding regions, Fig. 1e) across the analyzed organisms. This supports the conjecture that much of the variation in their strength among organisms is caused by common factors acting jointly on the level of ∆LFE at all regions of the CDS.

Fifth, we discussed several variables that correlate with ∆LFE (and account for much of the variation mentioned above). The variables showing the strongest correlation are genomic GC-content (despite being explicitly controlled for by our randomizations as explained above, Fig. 5) and CUB (measured using ENc′, Fig. 4). Strong CUB and higher GC-content tend to be associated with more efficient selection on translation efficiency (see, for example, [64, 65]), and the fact that ∆LFE is correlated with them suggests the same underlying mechanism (or mechanisms) contributes to their selection.

The influence on ∆LFE of all traits analyzed in the mid-CDS region can be compared in Fig. 9. Other genomic and environmental traits analyzed (including genome size and growth time) were not found to have significant linear interaction with ∆LFE at the domain level. In many cases, there appears to be potential interaction with ∆LFE in smaller taxons (which may or may not be due to real interactions specific to those taxons, Additional file 1: Figure S10).

**Fig. 10:**
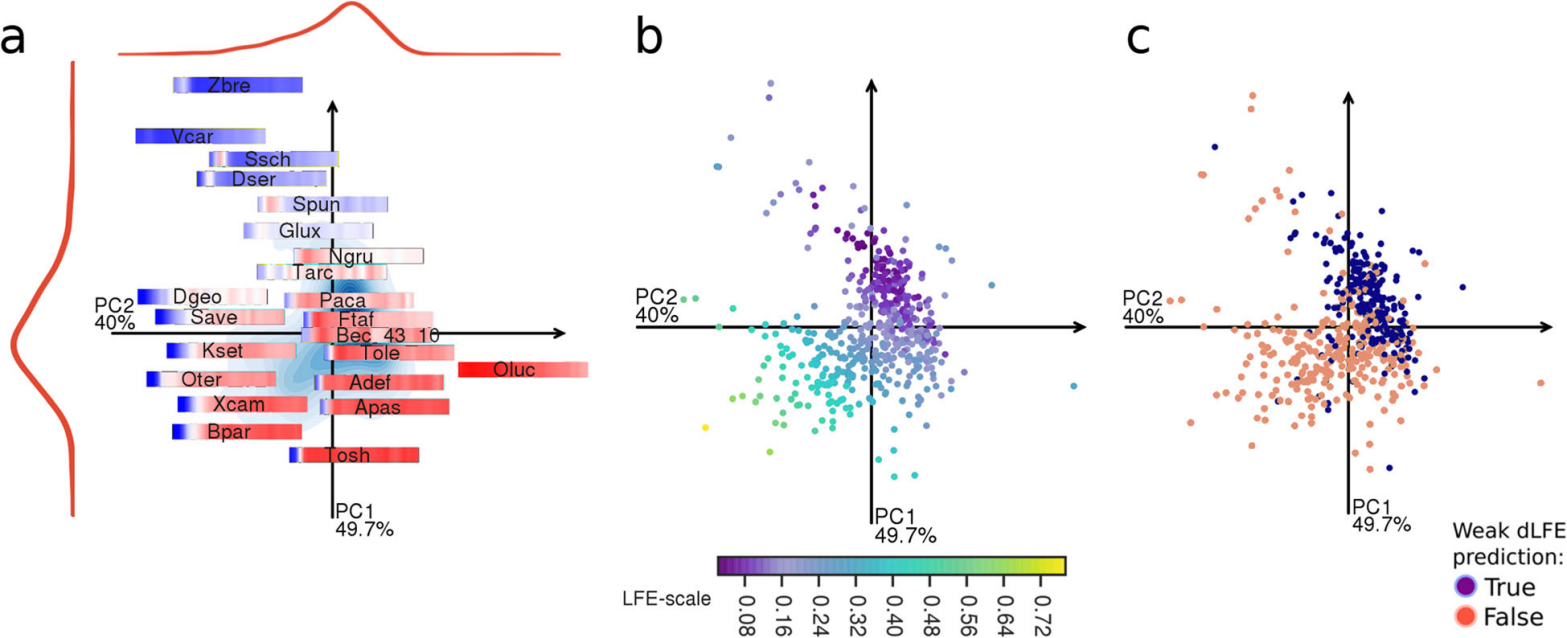
Classification model for weak ∆LFE based on four species traits. **a** PCA plot of ∆LFE profiles relative to CDS start (see “Visualization” under the “Methods” section). Short species names are listed in Additional file 1: Table S3. **b** ∆LFE profile strength, measured using standard deviation, for profile positions 0–300 nt relative to CDS start. **c** Predicted ∆LFE strength for each species using binary model for weak ∆LFE (precision = 0.66, recall = 0.82, *N* = 513, see “Binary classifier for ∆LFE strength” under the “Methods” section)

Sixth, we proposed four specific characteristics of species having weak ∆LFE (separately and together), demonstrating the conditions in which ∆LFE cannot be effectively maintained (or does not yield sufficient benefit to be maintained). The first two characteristics are based on the correlated traits described above: low GC-content and low CUB. Another characteristic is optimum growth temperature, since in higher temperatures, base-pairing is weakened, and consequently, the influence of codon arrangement and composition must also be reduced, and so is any possible effect of ∆LFE. The last disrupting factor, an intracellular life phase, stems from the fact that such organisms generally have lower effective population size (due to recurring population bottlenecks) and lower selection pressure on gene expression (because they partly rely on the host, [58, 59]). A binary classification model based on these four features has precision 0.66 and recall 0.82 in classification of ∆LFE strength (see “Analysis” under the “Methods” section and Fig. 10). Note that this binary classification discriminates species with very weak ∆LFE and has weak predictive value for ∆LFE strength in species where none of the factors hold, giving *R*^2^ = 0.2 (*p* value = 5e−25, OLS, all species) against mean |∆LFE| in the 150–300-nt region relative to CDS start. These conditions support the proposed mechanism of ∆LFE being the result of selection on secondary structure strength related to gene expression regulation and efficiency.

Our results point to cases where evolutionarily close organisms exhibit very different ∆LFE patterns and selection levels. For example, in fungi, members of *Pezizomycotina* (such as *Aspergilus niger* or *Zymoseptoria brevis*) have much more positive ∆LFE compared to members of *Saccharomycotina* (including *Eremothecium gossypii* and *Candida albicans*). Notably, a few eukaryotic species (e.g., the unrelated species *Fonticula alba* and *Saprolegnia parasitica*) have a ∆LFE profile that looks typical for bacteria (Additional file 1: Figure S7). This highlights the variety of gene expression mechanisms in eukaryotes, as well as the risk in generalizing about disparate groups based on observations on model organisms.

We would also like to emphasize the fact that ∆LFE has been considered a direct result of selection by previous studies cited here; we believe our results further support this hypothesis, for example, by showing ∆LFE is more conserved than genomic GC-content and demonstrating biologically reasonable trait interactions that may indicate a (direct or indirect) causal link. We should note however that our methodology does not assume any specific evolutionary process at work to produce the measured ∆LFE and this is an additional topic for further research.

Finally, we should note our analysis is based on average values over entire genomes. This provides important statistical power and reduces the random effects of other factors on specific genes. It is important to remember, however, that some of the gene-level factors filtered this way are nevertheless important and there is considerable variation between genes. This means that the reported features should be further analyzed in higher resolution, as well as validated experimentally to understand their origin. For example, ∆LFE in the mid-CDS region was suggested to be influence by both global factors like mRNA aggregation and local factors, like co-translational folding [29, 31], which may cause non-uniform selection pressure across the CDS. These differences may allow the effect of each factor to be experimentally validated separately. In addition, in future studies, it will be helpful and challenging to study the relation between ∆LFE and the position of genes in the operon (see [63]), and the influence of ∆LFE on the outcomes of translation initiation, termination, and splicing.

## Conclusions

1. The previously proposed regions of selection on local mRNA folding strength are widespread and appear in many species across domains. For two such regions (strong folding downstream of the beginning of the CDS and weak folding near the CDS end), this is first conclusively demonstrated here. However, none of these regions is universal and exceptions, which sometimes run opposite to the common trend, are quite common. Nevertheless, the CDS in most species does contain consistent regions of tendency for increased or decreased secondary structure strength. These regions coincide with parts of the CDS involved in different gene expression processes and in particular different stages of mRNA translation (initiation, elongation, and termination), supporting the conjecture that mRNA folding strength has a role in these stages of mRNA translation. In addition, stark differences in the prevalence of the regions suggest interactions with domain-specific regulatory mechanisms: For example, the selection for weak folding at the end of the coding region seems to be more common in eukaryotes while the selection for weak folding at the beginning of the coding region appears more commonly in prokaryotes.
2. The tendencies for increased or decreased secondary structure strength in different parts of the coding sequence are correlated among species across the tree of life, indicating common factors are affecting them throughout the coding sequence. We present four factors that predict the strength of local mRNA folding selection within the coding sequence—GC-content, CUB, intracellular life stage, and a hyperthermophilic environment. These factors are characteristic of species with strong optimization for gene expression efficiency or fidelity, suggesting mRNA folding strength also contributes to this optimization.
3. A “transition peak” of selection for strong mRNA folding around 30–70 nt downstream of the start codon appears in ~ 50% of the analyzed organisms, showing this phenomenon (suspected of being linked to optimization of translation elongation) is widespread.
4. The statistical framework we proposed for studying position-specific selection effects on traits like local mRNA folding across taxonomic groups, while controlling for confounding factors such as amino acid bias, codon, and evolutionary distance, enables inferring factors that may directly affect these traits.

## Methods

### Analysis

#### Species selection and sequence filtering

The set of species included in the dataset (Additional file 1: Table S1, Additional file 2) was chosen to maximize taxonomic coverage, include closely related species which differ in GC-contents and other traits (Fig. 2c), and take advantage of the limited overlap between available annotated genomes, NCBI environmental traits data, and the phylogenetic tree (see below). To prevent under-representation of taxons in the dataset, included species were tabulated by phylum and species from missing phyla and classes were added if possible (Additional file 1: Table S2). Over-representation of closely related species is controlled by GLS (see below).

CDS sequences and gene annotations for all species were obtained from Ensembl genomes [66], NCBI [67], JGI [68], and SGD [69] (Additional file 1: Table S3). CDS sequences were matched with their GFF3 annotations to filter suspect sequences, as follows. The dataset excludes CDSs marked as pseudo-genes or suspected pseudo-genes, incomplete CDSs, and those with sequencing ambiguities, as well as CDSs of length < 150 nt. If multiple isoforms were available, only the primary (or first) transcript was included. Genes annotated as belonging to organelle genomes were also excluded. Genomic GC-content, optimum growth temperatures, and translation tables were extracted from NCBI Entrez automatically, using a combination of Entrez and E-utilities requests (Additional file 1: Table S3). A few general characteristics of the included CDSs are shown in Fig. 2c.

The taxonomic hierarchy and classifications used to analyze and present the data were obtained from NCBI Taxonomy. Endosymbionts were annotated using a literature survey (Additional file 1: Table S3). Growth rates were extracted from [52] (Supplementary Table A1).

#### Randomization procedures

To test different hypotheses regarding local folding energy (LFE), native sequences were compared against randomized sequences preserving attributes as defined by each null hypothesis, as follows (Fig. 2a, b):

To test the hypothesis that the native *arrangement* of synonymous codons causes a significant bias in LFE, synonymous codons were randomly permuted within each CDS (i.e., all codons encoding for the same amino acid within a given CDS are randomly rearranged). This “CDS-wide” randomization preserves the encoded protein sequence, nucleotide frequencies (including GC-content), and codon frequencies of each CDS (but generally disrupts longer-range dependencies). Synonymous codons were determined according to the nuclear genetic code annotated for each species in NCBI genomes.

To test the contribution of position-specific biases in amino acid composition, nucleotide frequencies, and codon frequencies including CUB (factors that are equalized at the CDS level by the CDS-wide randomization) on the observed LFE, a second “position-specific” randomization was used. In this randomization, synonymous codons were randomly permuted between codons found at the same position (relative to the CDS start) across all CDSs in each genome. This randomization preserves the amino acid sequence of each CDS, while nucleotide (including GC-content) and codon frequencies are preserved at each *position* across a genome.

#### LFE profile calculation

Local folding energy (LFE) profiles were created by calculating the folding energy of all 40-nt-long windows, at 10-nt intervals, relative to the CDS start and end, on each native and randomized sequence. This measure estimates local secondary structure strength (ignoring the specific structures) and reflects (among other considerations) the structure of mRNA during translation, which prevents long-range structures but allows formation of local secondary structure and generally agrees with existing large-scale experimental validation results [37]. Previous studies (e.g., [35]) showed that this measure is robust to changes in the window size. The coordinates shown always refer to the window start position relative to the CDS start (e.g., window 0 includes the first 40 nt in the CDS) or to the window end position relative to the CDS end. Estimated folding energies were calculated for each window using *RNAfold* from the *ViennaRNA* package 2.3.0 [70], with the default settings. All folding energies were estimated at 37 °C so as to compare equivalent quantities between all genomes (but see below under native-temperature profiles). The ∆LFE profile for each protein, defined as the estimated excess local folding energy caused by the arrangement of synonymous codons at any CDS position, was created by subtracting the average profile of 20 randomized sequences for that protein from the native LFE profile:

$$ {\Delta  \mathrm{LFE}}_i={\mathrm{nativeLFE}}_i-\frac{1}{N}\sum \limits_{1\le n\le N}{\mathrm{randomizedLFE}}_i(n) $$

(*i*—CDS position, *N*—number of randomized sequences)

The mean ∆LFE profile for each species was created by averaging each position *i* over all proteins of sufficient length (so a different number of sequences may be averaged at each position). Note that while the native LFE of different CDSs within each genome varies considerably, the LFE of each native CDS is compared to *its own* set of randomized sequences.

To determine if the mean ∆LFE for a species in position *i* (relative to CDS start or end) is significantly different than 0, the differences *d*_*i*_(*p*, *n*) between LFE of the native and randomized sequences for each CDS *p* at position *i* were collected:

$$ {d}_i\left(p,n\right)={\mathrm{nativeLFE}}_i(p)-{\mathrm{randomizedLFE}}_i\left(p,n\right) $$

(*p*—CDS index, 1 *≤ n ≤ N =* 20—number of randomized sequences, *i*—CDS position)

The Wilcoxon signed-rank test was used on all values *d*_*i*_(*p*, *n*) (with the null hypothesis implying that the distribution is symmetrical).

#### Native-temperature profiles

The predicted folding energy calculations for native and randomized sequences for a sample of *N* = 71 bacterial and archaeal species were repeated using the same procedure but with folding predicted at the optimal growth temperature specified for that species (instead of 37 °C).

#### Phylogenetic tree preparation

To study the relation between ∆LFE profiles and other traits, the profiles were analyzed using a phylogenetic tree as follows. The phylogenetic tree is based on [71] (Supplementary Dataset 2 and Supplementary Table 1) and contains species from our dataset across the three domains of life. Since there are slight discrepancies in some node identifiers between the tree ([71] Supplementary Dataset 2) and accession table ([71] Supplementary Table 1), species names were matched by hand. Tree nodes and profiles were then matched by NCBI tax-ID at the species or lower level between the available genomes and phylogenetic tree nodes (e.g., when the tree species a species, and there is only one genome available for a specific strain of this species). The tree distances were converted to approximate relative ultrametric distances using *PATHd8* [72] version 1.9.8 with the default settings. Finally, the tree was pruned to the set of leaf nodes found in the dataset (or a subset of them which has data for both variables being correlated), by removing unused inner and leaf nodes and merging single-child inner nodes by summing distances. The resulting ultrametric tree (Additional file 3) was used to create a covariance matrix using a Brownian process (to reflect the null hypothesis that a trait is not under selection), using the *ape* package [73] in *R*.

#### Phylogenetically controlled regression

To test for correlations between traits among species while controlling for the similarity expected to exist between related species even in the absence of selection on either trait, generalized least-squared (GLS) regression was performed [74, 75] with the *nlme* package [76] in *R* and using REML optimization. Each regression included the subset of species for which data for both correlated traits was available, and which were also included in the tree. Regression *p* values are based on the null hypothesis that the slope of the explanatory variable is 0 (i.e., that the variables are independent), and estimated using the *t* test. Coefficient of determination (*R*^2^) values were calculated according to [75, 77]:

$$ {R}^2=1-\frac{\hat{u}^{\prime }{V}^{-1}\hat{u}}{{\left(Y-\overline{Y}e\right)}^{\prime }{V}^{-1}\left(Y-\overline{Y}e\right)} $$

$\hat{u}$—residuals, *V*—variance-covariance matrix, *Y*—observations, $\overline{Y}$—intercept of equivalent intercept-only model, and *e*—first column of design matrix.

For continuous traits, regression formulas included an intercept term. Discrete traits were represented by ordered or unordered factors, and the intercept term was omitted from the regression formula. For discrete traits, values of the explained variable (such as ∆LFE) were centered to have mean 0 (so regression is based on a null hypothesis that all levels have the same mean).

#### Regression robustness verification

To test the robustness of a correlation between traits at different CDS regions, the regression was repeated at all profile positions starting between 0 and 300 nt (relative to CDS start and end) and all contiguous subranges (using the mean ∆LFE value in each range) and reported only if consistent over the relevant range of positions (Additional file 1: Figure S17).

To test for specific trait correlations in individual taxons, the regression procedure was repeated for each taxonomic group (at any rank) containing at least 9 species (Additional file 1: Figure S10). For each taxonomic group, the value shown is the median *R*^2^ value for positions within the relevant range. The significance *p* value threshold was determined by applying FDR correction according to the number of taxonomic groups (treating them as independent to get a “worst-case” result).

#### Model element definition rules

Elements of the ∆LFE profile model were formalized as follows to allow estimation of their prevalence (Fig. 1a). Significance for all rules is defined using the Wilcoxon signed-rank test (see above) having *p* value < 0.05 at all positions within the range specified.

Model 1 (positive ends)

A. Positive start: ∆LFE value at positions 0–10 nt relative to CDS start is positive and significant.
B. Transition peak: the position of the minimum ∆LFE value in the range 0–300 nt, i*, is located in the range 20–80 nt relative to CDS start, and is significantly lower compared to all points in the ranges 0–10 nt and 100–200 nt relative to CDS start.
  To determine if the mean ∆LFE for a species in a given position *i* is significantly higher than the minimum (i*), the differences *w*_*i*_(*p*, *n*) between ∆LFE at the peak position and ∆LFE at the tested position were collected:
  $$ {w}_i\left(p,n\right)={d}_{i\ast}\left(p,n\right)-{d}_i\left(p,n\right) $$
  (*p*—CDS index, *N* ≤ 20—number of randomized sequences, *i*—position in CDS relative to start)

The Wilcoxon signed-rank test was used on all values *w*_*i*_(*p*, *n*).

C. Negative mid: ∆LFE values at each position in the range 200–300 nt relative to CDS start and in the range 300–200 nt relative to CDS end are all negative and significant.
D. Positive end: ∆LFE value at positions 10–0 nt relative to CDS end is positive and significant.
E. Model structure: A + C + D

Model 2 (weak ends)

A. Weak start: ∆LFE value at position 0 nt relative to CDS start is significantly higher than at positions 200–300 nt.
B. Same as in model 1.
C. Same as in model 1.
D. Weak end: ∆LFE value at position 0 nt relative to CDS end is significantly higher than at positions 200–300 nt.
E. Model structure: A + C + D

#### Binary classifier for ∆LFE strength

To measure the performances of several criteria in predicting ∆LFE strength, the following simple model was used. ∆LFE values for all species were divided into weak and strong groups based on the standard deviation of the mean ∆LFE at positions 0–300 nt. Species with standard deviation < 0.14 were included in the “weak ∆LFE” group. The binary classification of each species is based on 4 species traits as inputs, using the following rule (optimized using grid search):

$$ \mathrm{PredictedWeakLFE}=\left(\mathrm{Endosymbiont}=\mathrm{True}\right)\ \mathrm{or}\ \left(\mathrm{Genomic}\ \mathrm{GC}<38\%\right)\ \mathrm{or}\ \left(\mathrm{Genomic}\ {\mathrm{ENc}}^{\prime }>56.5\right)\ \mathrm{or}\ \left(\mathrm{Optimum}\ \mathrm{temp}>{58}^{\circ}\mathrm{C}\right) $$

#### Maximal information coefficient

Maximal information coefficient (MIC, [56, 57]) is a statistical measure of general (not necessarily linear) dependence between two variables. Informally, it is a generalization of *R*^2^ and also has values in the range 0.0–1.0, with high values indicating knowing the value of one variable allows inferring the value of the other. MIC was calculated using the *minerva* [78] package in *R*. *p* values were estimated using 10,000 random samples.

#### Correlogram plot

Correlogram plot (Additional file 1: Figure S2) was prepared using the *phylosignal* package in *R*.

#### Codon-bias metrics

Codon-bias metrics (CAI, CBI, Nc, Fop) were calculated for each genome using *codonW* [79] version 1.4.4. ENc′ [80] was calculated using *ENCprime* (github user jnovembre, commit 0ead568, October 2016) using the default settings. I_TE [43] was calculated using DAMBE7 [81], based on the included codon frequency tables for each species. DCBS was calculated according to [49].

#### Shine-Dalgarno binding strength

The Shine-Dalgarno (SD) strength for each gene was calculated according to, based on the minimal anti-SD hybridization energy found in the 20-nt region upstream of the start codon.

### Visualization

#### Taxon characteristic profile chart

The mean ∆LFE profiles for CDS positions 0–300 nt relative to the CDS start and end within each taxon were summarized (Fig. 3a) by grouping species with similar profiles and plotting one profile representing each group. The grouping was achieved by clustering the ∆LFE profiles (as vectors of length 31) using *K*-nearest neighbors agglomerative clustering with correlation distances, using *SciKit Learn* [82]. The profile plotted to represent each group is the centroid (mean) of each cluster. To allow easy viewing of the region of interest, only positions 0–150 nt are shown for each cluster. *K*, the number of clusters for each taxon, was chosen (separately for the start and end profiles) to be the smallest value for which the maximum distance of any profile to the centroid cluster mean (i.e., the profile shown) was smaller than 0.8 for the start-referenced profiles and 1.3 for the end-referenced profiles. The full ∆LFE profiles for all species appear in Additional file 1: Figure S7.

#### PCA display for ∆LFE profiles

To summarize ∆LFE profiles and show how different values related to different profile types, we used PCA to obtain a two-dimensional arrangement in which similar ∆LFE profiles are mapped to nearby positions (see, for example, Fig. 3b). Also shown are the amounts of variance explained by each of the first two principal components.

PCA for the ∆LFE profiles (treated as vectors of length 31) was performed using *SciKit Learn* [82]. Analysis was limited to the first 3 components, and only the first two components are displayed (Additional file 1: Figure S6a,b). To verify the robustness of the PCA results, they were repeated using 500 samples with replacement from the same PCA input vectors and of the same size, and the angles between the component were verified to be approximately equal (Additional file 1: Figure S6c). To reduce clutter, overlapping profiles are hidden and the relative density at each position is shown in the background as blue shading (estimated as bivariate KDE with bandwidth determined by Scott’s rule using *seaborn* [83]) and also plotted on the axes.

Evolutionary and taxonomic trees were plotted using the *ETE toolkit* [84].

## Supplementary information

**Additional file 1: Table S1**. List of species. **Table S2**. Phyla representation. **Table S3**. Genomic and environmental properties. **Figure S1.** Correlations of traits with ΔLFE are not present in its individual components. **Figure S2.** The ΔLFE profile is more conserved than other genomic traits. **Figure S3.** Local CUB vs. Local ΔLFE. **Figure S4.** Comparison between ΔLFE calculated using CDS-wide and position-specific (“vertical”) randomizations. **Figure S5.** ∆LFE is stronger in highly expressed genes and genes encoding for highly abundant proteins. **Figure S6.** Unsupervised discovery of profile regions. **Figure S7.** ΔLFE profiles for all species. **Figure S8.** Comparison between ΔLFE profiles in different domains. **Figure S9.** Autocorrelation between ΔLFE profile regions. **Figure S10.** Trait correlations in taxonomic subgroups. **Figure S11.** Correlation of ∆LFE with different genomic measures of CUB is consistent. **Figure S12.** ENc’ correlates with ΔLFE magnitude, not shape. **Figure S13.** Genomic-GC and genomic-ENc’ both predict ΔLFE. **Figure S14.** Endosymbionts have weaker ΔLFE. **Figure S15.** Range robustness for GLS regressions between ΔLFE and related traits. **Figure S16.** Additional controls for phenomenon related to translation initiation. **Figure S17**. Dependence of ΔLFE profiles on temperature.

**Additional file 2.** Species ΔLFE profiles and additional data used for GLS regression analysis.

**Additional file 3.** Processed ultrametric phylogenetic tree used for GLS regression analysis.

**Additional file 4.** Review history.

## Data Availability

All data reused in this study is publicly available from the sources specified in the methods. The annotated genomes used are available from the source specified in Additional file 1: Table S1. The dataset used for analysis is included in Additional file 2. The processed tree used for GLS analysis is included in Additional file 3. Software versions are specified in the methods. Python and R source code used for analysis is available from github repository https://github.com/michaelpeeri/rnafold-public [85]. All source code is licensed under the GNU General Public License (GPL) v3.

## References

[1] Trotta E (2013). Selection on codon bias in yeast: a transcriptional hypothesis. Nucleic Acids Res.

[2] Zamft B, Bintu L, Ishibashi T, Bustamante C (2012). Nascent RNA structure modulates the transcriptional dynamics of RNA polymerases. Proc Natl Acad Sci.

[3] Ray-Soni A, Bellecourt MJ, Landick R (2016). Mechanisms of bacterial transcription termination: all good things must end. Annu Rev Biochem.

[4] Ben-Yehezkel T, Atar S, Zur H, Diament A, Goz E, Marx T (2015). Rationally designed, heterologous S. cerevisiaetranscripts expose novel expression determinants. RNA Biol.

[5] Kozak M (2005). Regulation of translation via mRNA structure in prokaryotes and eukaryotes. Gene..

[6] Gilbert WV, Zhou K, Butler TK, Doudna JA (2007). Cap-independent translation is required for starvation-induced differentiation in yeast. Science..

[7] Xia X, Holcik M (2009). Strong eukaryotic IRESs have weak secondary structure. PLoS One.

[8] Zid BM, Rogers AN, Katewa SD, Vargas MA, Kolipinski MC, Lu TA (2009). 4E-BP extends lifespan upon dietary restriction by enhancing mitochondrial activity in Drosophila. Cell..

[9] Jagodnik J, Chiaruttini C, Guillier M (2017). Stem-loop structures within mRNA coding sequences activate translation initiation and mediate control by small regulatory RNAs. Mol Cell.

[10] Ding Y, Tang Y, Kwok CK, Zhang Y, Bevilacqua PC, Assmann SM (2014). *In vivo* genome-wide profiling of RNA secondary structure reveals novel regulatory features. Nature..

[11] Dvir S, Velten L, Sharon E, Zeevi D, Carey LB, Weinberger A (2013). Deciphering the rules by which 5′-UTR sequences affect protein expression in yeast. Proc Natl Acad Sci.

[12] Kertesz M, Wan Y, Mazor E, Rinn JL, Nutter RC, Chang HY (2010). Genome-wide measurement of RNA secondary structure in yeast. Nature..

[13] Bhattacharyya S, Jacobs WM, Adkar BV, Yan J, Zhang W, Shakhnovich EI (2018). Accessibility of the Shine-Dalgarno sequence dictates N-terminal codon bias in *E. coli*. Mol Cell.

[14] Behloul N, Wei W, Baha S, Liu Z, Wen J, Meng J (2017). Effects of mRNA secondary structure on the expression of HEV ORF2 proteins in Escherichia coli. Microb Cell Factories.

[15] Wu B, Zhang H, Sun R, Peng S, Cooperman BS, Goldman YE (2018). Translocation kinetics and structural dynamics of ribosomes are modulated by the conformational plasticity of downstream pseudoknots. Nucleic Acids Res.

[16] Wen J-D, Lancaster L, Hodges C, Zeri A-C, Yoshimura SH, Noller HF (2008). Following translation by single ribosomes one codon at a time. Nature..

[17] Qu X, Wen J-D, Lancaster L, Noller HF, Bustamante C, Tinoco I (2011). The ribosome uses two active mechanisms to unwind messenger RNA during translation. Nature..

[18] Tuller T, Veksler-Lublinsky I, Gazit N, Kupiec M, Ruppin E, Ziv-Ukelson M (2011). Composite effects of gene determinants on the translation speed and density of ribosomes. Genome Biol.

[19] Komar AA (2009). A pause for thought along the co-translational folding pathway. Trends Biochem Sci.

[20] Park C, Chen XS, Yang JR, Zhang JZ (2013). Differential requirements for mRNA folding partially explain why highly expressed proteins evolve slowly. Proc Natl Acad Sci U S A.

[21] Zhang G, Hubalewska M, Ignatova Z (2009). Transient ribosomal attenuation coordinates protein synthesis and co-translational folding. Nat Struct Mol Biol.

[22] Zur H, Tuller T (2012). Strong association between mRNA folding strength and protein abundance in S. cerevisiae. EMBO Rep.

[23] Lenz G, Doron-Faigenboim A, Ron EZ, Tuller T, Gophna U (2011). Sequence features of *E. coli* mRNAs affect their degradation. PLOS ONE.

[24] Wan Y, Qu K, Ouyang Z, Kertesz M, Li J, Tibshirani R (2012). Genome-wide measurement of RNA folding energies. Mol Cell.

[25] Zafrir Z, Zur H, Tuller T (2016). Selection for reduced translation costs at the intronic 5′ end in fungi. DNA Res.

[26] Mortimer SA, Kidwell MA, Doudna JA (2014). Insights into RNA structure and function from genome-wide studies. Nat Rev Genet.

[27] Mauger DM, Siegfried NA, Weeks KM (2013). The genetic code as expressed through relationships between mRNA structure and protein function. FEBS Lett.

[28] Jacobs E, Mills JD, Janitz M (2012). The role of RNA structure in posttranscriptional regulation of gene expression. J Genet Genomics.

[29] Faure G, Ogurtsov AY, Shabalina SA, Koonin EV (2016). Role of mRNA structure in the control of protein folding. Nucleic Acids Res.

[30] Itzkovitz S, Hodis E, Segal E. Overlapping codes within protein-coding sequences. Genome Res. 2010;20:1582–9. Available from: 10.1101/gr.105072.110. PMC2963821 20841429

[31] Katz L, Burge CB (2003). Widespread selection for local RNA secondary structure in coding regions of bacterial genes. Genome Res.

[32] Shabalina SA, Ogurtsov AY, Spiridonov NA (2006). A periodic pattern of mRNA secondary structure created by the genetic code. Nucleic Acids Res.

[33] Xia X (2017). DAMBE6: new tools for microbial genomics, phylogenetics, and molecular evolution. J Hered.

[34] Xia X. Bioinformatics and the cell: modern computational approaches in genomics. Proteomics and Transcriptomics: Springer; 2018. p. 494.

[35] Mao Y, Wang W, Cheng N, Li Q, Tao S (2013). Universally increased mRNA stability downstream of the translation initiation site in eukaryotes and prokaryotes. Gene..

[36] Tuller T, Zur H (2015). Multiple roles of the coding sequence 5′ end in gene expression regulation. Nucleic Acids Res.

[37] Del Campo C, Bartholomäus A, Fedyunin I, Ignatova Z. Secondary structure across the bacterial transcriptome reveals versatile roles in mRNA regulation and function. PLoS Genet. 2015;11(10):e1005613. 10.1371/journal.pgen.1005613. 10.1371/journal.pgen.1005613 PMC4619774 26495981

[38] Kozak M (1980). Influence of mRNA secondary structure on binding and migration of 40S ribosomal subunits. Cell..

[39] Osterman IA, Evfratov SA, Sergiev PV, Dontsova OA (2013). Comparison of mRNA features affecting translation initiation and reinitiation. Nucleic Acids Res.

[40] Gu W, Zhou T, Wilke CO. A universal trend of reduced mRNA stability near the translation-initiation site in prokaryotes and eukaryotes. PLoS Comput Biol. 2010;6(2):e1000664. 10.1371/journal.pcbi.1000664. 10.1371/journal.pcbi.1000664 PMC2816680 20140241

[41] Keller TE, Mis SD, Jia KE, Wilke CO (2012). Reduced mRNA secondary-structure stability near the start codon indicates functional genes in prokaryotes. Genome Biol Evol.

[42] Tuller T, Waldman YY, Kupiec M, Ruppin E (2010). Translation efficiency is determined by both codon bias and folding energy. Proc Natl Acad Sci U S A.

[43] Xia X (2015). A major controversy in codon-anticodon adaptation resolved by a new codon usage index. Genetics..

[44] Wei Y, Xia X (2019). Unique Shine–Dalgarno sequences in cyanobacteria and chloroplasts reveal evolutionary differences in their translation initiation. Genome Biol Evol..

[45] Xia X (2019). Optimizing phage translation initiation. OBM Genet.

[46] Dunteman GH. Principal components analysis. Newbury Park: SAGE Publication, Inc; 1989. https://uk.sagepub.com/en-gb/mst/principal-components-analysis/book2504.

[47] Bennetzen JL, Hall BD (1982). Codon selection in yeast. J Biol Chem.

[48] Grosjean H, Fiers W (1982). Preferential codon usage in prokaryotic genes: the optimal codon-anticodon interaction energy and the selective codon usage in efficiently expressed genes. Gene..

[49] Sabi R, Tuller T (2014). Modelling the efficiency of codon–tRNA interactions based on codon usage bias. DNA Res.

[50] Wright F (1990). The “effective number of codons” used in a gene. Gene..

[51] Rocha EPC (2004). Codon usage bias from tRNA’s point of view: redundancy, specialization, and efficient decoding for translation optimization. Genome Res.

[52] Vieira-Silva S, Rocha EPC (2010). The systemic imprint of growth and its uses in ecological (meta)genomics. PLoS Genet.

[53] Sharp PM, Li WH (1987). The codon adaptation index--a measure of directional synonymous codon usage bias, and its potential applications. Nucleic Acids Res.

[54] Hildebrand F, Meyer A, Eyre-Walker A (2010). Evidence of selection upon genomic GC-content in bacteria. PLoS Genet.

[55] Lee KY, Wahl R, Barbu E. Contenu en bases puriques et pyrimidiques des acides désoxyribonucléiques des bactéries. Ann Inst Pasteur (Paris). 1956;91(2):212-24. 13363015

[56] Reshef DN, Reshef YA, Finucane HK, Grossman SR, McVean G, Turnbaugh PJ (2011). Detecting novel associations in large data sets. Science..

[57] Shaham G, Tuller T (2014). Most associations between transcript features and gene expression are monotonic. Mol BioSyst.

[58] Andersson SGE, Kurland CG (1998). Reductive evolution of resident genomes. Trends Microbiol.

[59] Woolfit M (2009). Effective population size and the rate and pattern of nucleotide substitutions. Biol Lett.

[60] McCutcheon JP, Moran NA (2012). Extreme genome reduction in symbiotic bacteria. Nat Rev Microbiol.

[61] Hickey DA, Singer GA (2004). Genomic and proteomic adaptations to growth at high temperature. Genome Biol.

[62] Hurst LD, Merchant AR (2001). High guanine–cytosine content is not an adaptation to high temperature: a comparative analysis amongst prokaryotes. Proc R Soc Lond B Biol Sci.

[63] Chemla Y, Peeri M, Heltberg ML, Eichler J, Jensen MH, Tuller T, et al. mRNA secondary structure stability regulates bacterial translation insulation and re-initiation. BioRxiv. 2020; biorxiv.org. Available from: 10.1101/2020.02.10.941153.

[64] dos Reis M, Wernisch L (2009). Estimating translational selection in eukaryotic genomes. Mol Biol Evol.

[65] dos Reis M, Savva R, Wernisch L (2004). Solving the riddle of codon usage preferences: a test for translational selection. Nucleic Acids Res.

[66] Kersey PJ, Allen JE, Allot A, Barba M, Boddu S, Bolt BJ (2018). Ensembl genomes 2018: an integrated omics infrastructure for non-vertebrate species. Nucleic Acids Res.

[67] NCBI Resource Coordinators. Database resources of the National Center for Biotechnology Information. Nucleic Acids Res. 2018;46(D1):D8-D13. 10.1093/nar/gkx1095. 10.1093/nar/gkx1095 PMC5753372 29140470

[68] Nordberg H, Cantor M, Dusheyko S, Hua S, Poliakov A, Shabalov I (2014). The genome portal of the Department of Energy Joint Genome Institute: 2014 updates. Nucleic Acids Res.

[69] Engel SR, Dietrich FS, Fisk DG, Binkley G, Balakrishnan R, Costanzo MC (2013). The reference genome sequence of *Saccharomyces cerevisiae*: then and now. G3 GenesGenomesGenetics.

[70] Lorenz R, Bernhart SH, Höner zu Siederdissen C, Tafer H, Flamm C, Stadler PF, et al. ViennaRNA Package 2.0. Algorithms Mol Biol. 2011;6(1):26. 10.1186/1748-7188-6-26 PMC3319429 22115189

[71] Hug LA, Baker BJ, Anantharaman K, Brown CT, Probst AJ, Castelle CJ (2016). A new view of the tree of life. Nat Microbiol.

[72] Britton T, Anderson CL, Jacquet D, Lundqvist S, Bremer K, Anderson F (2007). Estimating divergence times in large phylogenetic trees. Syst Biol.

[73] Paradis E, Claude J, Strimmer K (2004). APE: analyses of phylogenetics and evolution in R language. Bioinformatics..

[74] Aitken AC. IV.—On least squares and linear combination of observations. Proc R Soc Edinb. 1936;55:42–8.

[75] Paradis E. Analysis of macroevolution with phylogenies. Anal Phylogenetics Evol R. 2012:203–312.

[76] Pinheiro J, Bates D, DebRoy S, Sarkar D, Heisterkamp S, Van Willigen B. nlme: linear and nonlinear mixed effects models. R Package 3rd Edn. 2017;1–336.

[77] Buse A (1973). Goodness of fit in generalized least squares estimation. Am Stat.

[78] Albanese D, Filosi M, Visintainer R, Riccadonna S, Jurman G, Furlanello C. Minerva and minepy: a C engine for the MINE suite and its R, Python and MATLAB wrappers. Bioinformatics. 2013;29(3):407-8. 10.1093/bioinformatics/bts707. Epub 2012 Dec 14. 10.1093/bioinformatics/bts707 23242262

[79] Peden JF. Analysis of codon usage. PhD dissertation. Nottingham: University of Nottingham; 1999. Available from: http://codonw.sourceforge.net/.

[80] Novembre JA (2002). Accounting for background nucleotide composition when measuring codon usage bias. Mol Biol Evol.

[81] Xia X (2018). DAMBE7: new and improved tools for data analysis in molecular biology and evolution. Mol Biol Evol.

[82] Pedregosa F, Varoquaux G, Gramfort A, Michel V, Thirion B, Grisel O (2011). Scikit-learn: machine learning in Python. J Mach Learn Res.

[83] Waskom M. Seaborn: statistical data visualization, version 0.9.0. 2019. Available from: https://seaborn.pydata.org/. Accessed 22 Apr 2019.

[84] Huerta-Cepas J, Serra F, Bork P (2016). ETE 3: reconstruction, analysis, and visualization of phylogenomic data. Mol Biol Evol.

[85] Peeri M, Tuller T. High resolution modeling of the selection on local mRNA folding strength in coding sequences across the tree of life. Source code. 2020. Available from: github https://github.com/michaelpeeri/rnafold-public/. Accessed 25 Feb 2020. 10.1186/s13059-020-01971-y PMC7063772 32151272

