# Supplementary material for: High-resolution modeling of the selection on local mRNA folding strength in coding sequences across the tree of life
Source: Genome Biol. 2020 Mar 9;21:63. doi: 10.1186/s13059-020-01971-y (PMC7063772; doi:10.1186/s13059-020-01971-y)
Supplement: Supplementary file 1 — Additional file 1: Table S1. List of species. Table S2. Phyla representation. Table S3. Genomic and environmental properties. Figure S1. Correlations of traits with ΔLFE are not present in its individual components. Figure S2. The ΔLFE profile is more conserved than other genomic traits. Figure S3. Local CUB vs. Local ΔLFE. Figure S4. Comparison between ΔLFE calculated using CDS-wide and position-specific (“vertical”) randomizations. Figure S5. ∆LFE is stronger in highly expressed genes and genes encoding for highly abundant proteins. Figure S6. Unsupervised discovery of profile regions. Figure S7. ΔLFE profiles for all species. Figure S8. Comparison between ΔLFE profiles in different domains. Figure S9. Autocorrelation between ΔLFE profile regions. Figure S10. Trait correlations in taxonomic subgroups. Figure S11. Correlation of ∆LFE with different genomic measures of CUB is consistent. Figure S12. ENc’ correlates with ΔLFE magnitude, not shape. Figure S13. Genomic-GC and genomic-ENc’ both predict ΔLFE. Figure S14. Endosymbionts have weaker ΔLFE. Figure S15. Range robustness for GLS regressions between ΔLFE and related traits. Figure S16. Additional controls for phenomenon related to translation initiation. Figure S17. Dependence of ΔLFE profiles on temperature. [file 13059_2020_1971_MOESM1_ESM.pdf]

Supplementary figures

List of supplementary figures

Table S1. List of species

Table S2. Phyla representation

Table S3. Genomic and environmental properties

Fig. S1. Correlations of traits with ΔLFE are not present in its individual components

Fig. S2. The ΔLFE profile is more conserved than other genomic traits

Fig. S3. Local CUB vs. Local ΔLFE

Fig. S4. Comparison between ΔLFE calculated using CDS-wide and position-specific (“vertical”) randomizations

Fig. S5. ΔLFE is stronger in highly expressed genes and genes encoding for highly abundant proteins

Fig. S6. Unsupervised discovery of profile regions

Fig. S7. ΔLFE profiles for all species

Fig. S8. Comparison between ΔLFE profiles in different domains

Fig. S9. Autocorrelation between ΔLFE profile regions

Fig. S10. Trait correlations in taxonomic subgroups

Fig. S11. Correlation of ΔLFE with different genomic measures of CUB is consistent

Fig. S12. ENc’ correlates with ΔLFE magnitude, not shape

Fig. S13. Genomic-GC and genomic-ENc’ both predict ΔLFE

Fig. S14. Endosymbionts have weaker ΔLFE

Fig. S15. Range robustness for GLS regressions between ΔLFE and related traits

Fig. S16. Additional controls for phenomenon related to translation initiation

Fig. S17. Dependence of ΔLFE profiles on temperature

Supplementary references

Table S1. List of species

TaxId	Species	Nick-name	Ann. GC%	CDS GC%	Num CDSs	Phylum	Domain	Source
**747**	Pasteurella multocida str. ATCC 43137	Pmul	40.30	41.03	2036	Proteobacteria	Bacteria	Ensembl
**882**	Desulfovibrio vulgaris str. Hildenborough	Dvul	67.10	63.53	3510	Proteobacteria	Bacteria	Ensembl
**979**	Cellulophaga lytica	Clyt	32.10	32.67	3168	Bacteroidetes	Bacteria	Ensembl
**1148**	Synechocystis sp. PCC 6803	SPCC	47.35	48.22	3564	Cyanobacteria	Bacteria	Ensembl
**2769**	Chondrus crispus (carragheen)	Ccri	52.86	53.68	8815		Eukaryota	Ensembl
**2898**	Cryptomonas paramecium	Crymecium	27.81	25.98	465		Eukaryota	Ensembl
**3046**	Dunaliella salina	Dsal	40.10	58.19	16005	Chlorophyta	Eukaryota	JGI
**3055**	Chlamydomonas reinhardtii	Crei	61.95	70.24	17741	Chlorophyta	Eukaryota	JGI
**3067**	Volvox carteri	Vcar	55.30	63.34	14241	Chlorophyta	Eukaryota	JGI
**3218**	Physcomitrella patens	Ppat	34.30	49.31	32108	Streptophyta	Eukaryota	Ensembl
**4781**	Plasmopara halstedii	Phal	45.70	45.97	14306		Eukaryota	Ensembl
**4927**	Wickerhamomyces anomalus NRRL Y-366-8	Wano	35.00	34.54	6262	Ascomycota	Eukaryota	Ensembl
**5061**	Aspergillus niger	Anig	50.30	53.72	13713	Ascomycota	Eukaryota	Ensembl
**5693**	Trypanosoma cruzi	Tcru	51.70	53.16	18456		Eukaryota	Ensembl
**6669**	Daphnia pulex	Dpul	42.40	47.30	30162	Arthropoda	Eukaryota	Ensembl
**10228**	Trichoplax adhaerens	Tadh	34.50	37.71	11435	Placozoa	Eukaryota	Ensembl
**27923**	Mnemiopsis leidyi	Mlei	39.10	45.66	15557	Ctenophora	Eukaryota	Ensembl
**28892**	Methanofollis liminatans DSM 4140	Mlim	61.00	61.95	2422	Euryarchaeota	Archaea	Ensembl
**29290**	Candidatus Magnetobacterium bavaricum	Mbav	47.30	48.21	5870	Nitrospirae	Bacteria	Ensembl
**29656**	Spirodela polyrhiza	Spol	42.72	55.64	19462	Streptophyta	Eukaryota	JGI
**36329**	Plasmodium falciparum 3D7	Pfal	19.36	23.74	5356	Apicomplexa	Eukaryota	Ensembl
**44056**	Aureococcus anophagefferens	Aano	67.40	70.80	11189		Eukaryota	Ensembl
**45351**	Nematostella vectensis	Nvec	41.90	47.35	24239	Cnidaria	Eukaryota	Ensembl
**45670**	Salinicoccus roseus	Sros	50.00	51.23	2399	Firmicutes	Bacteria	Ensembl
**46234**	Anabaena sp. 90	A90	38.09	38.76	4501	Cyanobacteria	Bacteria	Ensembl
**49280**	Gelidibacter algens	Galg	37.30	38.19	3654	Bacteroidetes	Bacteria	Ensembl
**59374**	Fibrobacter succinogenes subsp. succinogenes S85	Fsuc	48.00	48.89	3079	Fibrobacteres	Bacteria	Ensembl
**63737**	Nostoc punctiforme PCC 73102	Npun	41.34	42.59	6620	Cyanobacteria	Bacteria	Ensembl
**64091**	Halobacterium salinarum NRC-1	HsalNRC-1	65.70	66.88	2586	Euryarchaeota	Archaea	Ensembl
**65357**	Albugo candida	Acan	43.20	44.63	13222		Eukaryota	Ensembl
**70601**	Pyrococcus horikoshii OT3	Phor	41.90	42.32	2061	Euryarchaeota	Archaea	Ensembl

TaxId	Species	Nick-name	Ann. GC%	CDS GC%	Num CDSs	Phylum	Domain	Source
**83332**	Mycobacterium tuberculosis H37Rv	Mtub	65.60	65.90	4016	Actinobacteria	Bacteria	Ensembl
**85962**	Helicobacter pylori 26695	Hpyl	38.90	39.61	1554	Proteobacteria	Bacteria	Ensembl
**93061**	Staphylococcus aureus subsp. aureus NCTC 8325	Saur	32.90	33.51	2625	Firmicutes	Bacteria	Ensembl
**96563**	Pseudomonas stutzeri	Pstu	60.60	64.52	4052	Proteobacteria	Bacteria	Ensembl
**99287**	Salmonella enterica subsp. enterica serovar Typhimurium str. LT2	Sent	51.88	53.35	4545	Proteobacteria	Bacteria	Ensembl
**100226**	Streptomyces coelicolor A3(2)	Scoe	71.98	72.34	8109	Actinobacteria	Bacteria	Ensembl
**104782**	Adineta vaga	Avag	31.20	33.33	47746	Rotifera	Eukaryota	Ensembl
**107806**	Buchnera aphidicola str. APS (Acyrthosiphon pisum)	Baph	25.30	27.43	574	Proteobacteria	Bacteria	Ensembl
**115713**	Chlamydophila pneumoniae CWL029	CpneWL029	40.60	41.34	1052	Chlamydiae	Bacteria	Ensembl
**122586**	Neisseria meningitidis MC58	Nmen	51.50	53.08	2048	Proteobacteria	Bacteria	Ensembl
**123214**	Persephonella marina EX-H1	PmarEX-H1	37.12	37.31	2048	Aquificae	Bacteria	Ensembl
**130081**	Galdieria sulphuraria	Gsul	37.90	39.68	7089		Eukaryota	Ensembl
**138677**	Chlamydophila pneumoniae J138	CpneJ138	40.60	41.36	1068	Chlamydiae	Bacteria	Ensembl
**145458**	Rathayibacter toxicus	Rtox	61.50	61.94	1740	Actinobacteria	Bacteria	Ensembl
**153151**	Parageobacillus toebii	Ptoe	42.10	42.95	3780	Firmicutes	Bacteria	Ensembl
**155920**	Xylella fastidiosa subsp. sandyi Ann-1	XfasAnn-1	52.64	53.57	2626	Proteobacteria	Bacteria	Ensembl
**156889**	Magnetococcus marinus MC-1	Mmar	54.20	54.79	3716	Proteobacteria	Bacteria	Ensembl
**158189**	Sphaerochaeta globosa str. Buddy	Sglo	48.90	49.41	3017	Spirochaetes	Bacteria	
**160490**	Streptococcus pyogenes M1 GAS	Spyo	38.50	39.15	1686	Firmicutes	Bacteria	Ensembl
**160492**	Xylella fastidiosa 9a5c	Xfas9a5c	52.64	53.72	2823	Proteobacteria	Bacteria	Ensembl
**163003**	Thermococcus cleftensis	Tcle	55.80	56.66	1989	Euryarchaeota	Archaea	Ensembl
**164328**	Phytophthora ramorum	Pram	53.00	58.02	15109		Eukaryota	Ensembl
**167546**	Prochlorococcus marinus str. MIT 9301	Pmar	36.40	32.06	1891	Cyanobacteria	Bacteria	Ensembl
**169963**	Listeria monocytogenes EGD-e	Lmon	38.00	38.44	2843	Firmicutes	Bacteria	Ensembl
**176280**	Staphylococcus epidermidis ATCC 12228	Sepi	32.05	32.90	2429	Firmicutes	Bacteria	Ensembl
**176299**	Agrobacterium fabrum str. C58	Afab	59.06	59.82	5352	Proteobacteria	Bacteria	Ensembl
**178306**	Pyrobaculum aerophilum str. IM2	PaerIM2	51.40	51.90	2594	Crenarchaeota	Archaea	Ensembl
**184922**	Giardia lamblia ATCC 50803	Glam	49.20	49.02	7313		Eukaryota	Ensembl
**186497**	Pyrococcus furiosus DSM 3638	Pfur	40.80	41.09	2060	Euryarchaeota	Archaea	Ensembl
**187272**	Alkalilimnicola ehrlichii MLHE-1	Aehr	67.50	67.82	2863	Proteobacteria	Bacteria	Ensembl
**187420**	Methanothermobacter thermautotrophicus str. Delta	Mthe	49.50	50.56	1867	Euryarchaeota	Archaea	Ensembl

TaxId	Species	Nick-name	Ann. GC%	CDS GC%	Num CDSs	Phylum	Domain	Source
	H							
**188937**	Methanosarcina acetivorans C2A	Mace	42.70	45.17	4539	Euryarchaeota	Archaea	Ensembl
**190192**	Methanopyrus kandleri AV19	Mkan	61.20	61.20	1687	Euryarchaeota	Archaea	Ensembl
**190304**	Fusobacterium nucleatum subsp. nucleatum ATCC 25586	Fnuc	27.20	27.39	2036	Fusobacteria	Bacteria	Ensembl
**190485**	Xanthomonas campestris pv. campestris str. ATCC 33913	Xcam	65.10	65.58	4177	Proteobacteria	Bacteria	Ensembl
**190650**	Caulobacter crescentus CB15	Ccre	67.20	67.68	3728	Proteobacteria	Bacteria	Ensembl
**192222**	Campylobacter jejuni subsp. jejuni NCTC 11168 = ATCC 700819	Cjej	30.50	30.83	1610	Proteobacteria	Bacteria	Ensembl
**194439**	Chlorobium tepidum TLS	Ctep	56.50	57.63	2220	Chlorobi	Bacteria	Ensembl
**195522**	Thermococcus nautili	Tnau	54.80	55.51	2161	Euryarchaeota	Archaea	Ensembl
**196162**	Nocardioides sp. JS614	NJS6	71.48	71.67	4888	Actinobacteria	Bacteria	Ensembl
**196164**	Corynebacterium efficiens YS-314	Ceff	62.93	63.68	2996	Actinobacteria	Bacteria	Ensembl
**196600**	Vibrio vulnificus YJ016	Vvul	46.67	47.48	5024	Proteobacteria	Bacteria	Ensembl
**196627**	Corynebacterium glutamicum ATCC 13032	Cglu	53.80	54.78	3053	Actinobacteria	Bacteria	Ensembl
**203123**	Oenococcus oeni PSU-1	Ooen	37.90	38.88	1677	Firmicutes	Bacteria	Ensembl
**203124**	Trichodesmium erythraeum IMS101	Tery	34.10	36.77	4440	Cyanobacteria	Bacteria	Ensembl
**203267**	Tropheryma whipplei str. Twist	Twhi	46.30	46.46	808	Actinobacteria	Bacteria	Ensembl
**203907**	Candidatus Blochmannia floridanus	Bflo	27.40	28.90	582	Proteobacteria	Bacteria	Ensembl
**204536**	Sulfurihydrogenibium azorense Az-Fu1	Sazo	32.80	32.80	1720	Aquificae	Bacteria	Ensembl
**208964**	Pseudomonas aeruginosa PAO1	Paer	66.60	67.16	5523	Proteobacteria	Bacteria	Ensembl
**211586**	Shewanella oneidensis MR-1	Sone	45.93	46.94	4191	Proteobacteria	Bacteria	Ensembl
**212717**	Clostridium tetani E88	Ctet	28.59	29.00	2432	Firmicutes	Bacteria	Ensembl
**213585**	Methanosarcina mazei S-6	Mmaz	41.40	44.14	3335	Euryarchaeota	Archaea	Ensembl
**214684**	Cryptococcus neoformans var. neoformans JEC21	Cneo	48.54	51.16	6570	Basidiomycota	Eukaryota	Ensembl
**216432**	Croceibacter atlanticus HTCC2559	Catl	33.90	34.33	2696	Bacteroidetes	Bacteria	Ensembl
**218497**	Chlamydia abortus S26-3	Cabo	39.90	40.49	932	Chlamydiae	Bacteria	Ensembl
**220668**	Lactobacillus plantarum WCFS1	Lpla	44.45	45.47	3101	Firmicutes	Bacteria	Ensembl
**221109**	Oceanobacillus iheyensis HTE831	Oihe	35.70	36.10	3490	Firmicutes	Bacteria	Ensembl
**223926**	Vibrio parahaemolyticus RIMD 2210633	Vpar	45.40	46.28	4522	Proteobacteria	Bacteria	Ensembl
**224308**	Bacillus subtilis subsp. subtilis str. 168	Bsub	43.50	44.22	4120	Firmicutes	Bacteria	Ensembl
**224324**	Aquifex aeolicus VF5	AaeoVF5	43.32	43.58	1553	Aquificae	Bacteria	Ensembl
**224325**	Archaeoglobus fulgidus DSM 4304	Aful	48.60	49.36	2405	Euryarchaeota	Archaea	Ensembl

TaxId	Species	Nick-name	Ann. GC%	CDS GC%	Num CDSs	Phylum	Domain	Source
**224914**	Brucella melitensis bv. 1 str. 16M	Bmel	57.24	58.28	3194	Proteobacteria	Bacteria	Ensembl
**226185**	Enterococcus faecalis V583	Efae	37.35	37.95	3241	Firmicutes	Bacteria	Ensembl
**226186**	Bacteroides thetaiotaomicron VPI-5482	Bthe	42.82	43.91	4825	Bacteroidetes	Bacteria	Ensembl
**227377**	Coxiella burnetii RSA 493	Cbur	42.34	43.22	1828	Proteobacteria	Bacteria	Ensembl
**227882**	Streptomyces avermitilis MA-4680 = NBRC 14893	Save	70.60	71.12	7661	Actinobacteria	Bacteria	Ensembl
**228410**	Nitrosomonas europaea ATCC 19718	Neur	50.70	51.57	2462	Proteobacteria	Bacteria	Ensembl
**228908**	Nanoarchaeum equitans	Nequ	31.60	31.20	536	Nanoarchaeota	Archaea	Ensembl
**233412**	Haemophilus ducreyi 35000HP	Hduc	38.20	38.74	1694	Proteobacteria	Bacteria	Ensembl
**234267**	Candidatus Solibacter usitatus Ellin6076	Susi	61.90	62.43	7825	Acidobacteria	Bacteria	Ensembl
**235909**	Geobacillus kaustophilus HTA426	Gkau	51.99	52.84	3531	Firmicutes	Bacteria	Ensembl
**237561**	Candida albicans SC5314	Calb	33.48	35.23	14102	Ascomycota	Eukaryota	Ensembl
**240015**	Acidobacterium capsulatum ATCC 51196	Acap	60.50	61.10	3376	Acidobacteria	Bacteria	Ensembl
**242507**	Magnaporthe oryzae	Mory	51.59	57.72	12746	Ascomycota	Eukaryota	Ensembl
**243090**	Rhodopirellula baltica SH 1	Rbal	55.40	55.46	7325	Planctomycetes	Bacteria	Ensembl
**243159**	Acidithiobacillus ferrooxidans ATCC 23270	Afer23270	58.80	59.32	3129	Proteobacteria	Bacteria	Ensembl
**243230**	Deinococcus radiodurans R1	Drad	66.61	67.23	3050	Deinococcus-Thermus	Bacteria	Ensembl
**243232**	Methanocaldococcus jannaschii DSM 2661	Mjan	31.27	31.85	1755	Euryarchaeota	Archaea	Ensembl
**243233**	Methylococcus capsulatus str. Bath	Mcap	63.60	63.96	2959	Proteobacteria	Bacteria	Ensembl
**243265**	Photorhabdus luminescens subsp. laumondii TTO1	Plum	42.80	44.16	4680	Proteobacteria	Bacteria	Ensembl
**243273**	Mycoplasma genitalium G37	Mgen	31.70	31.55	476	Tenericutes	Bacteria	Ensembl
**243274**	Thermotoga maritima MSB8	Tmar	46.20	46.40	1800	Thermotogae	Bacteria	Ensembl
**243275**	Treponema denticola ATCC 35405	Tden35405	37.90	38.27	2726	Spirochaetes	Bacteria	Ensembl
**243365**	Chromobacterium violaceum ATCC 12472	Cvio	64.80	65.71	4399	Proteobacteria	Bacteria	Ensembl
**251221**	Gloeobacter violaceus PCC 7421	Gvio	62.00	62.86	4357	Cyanobacteria	Bacteria	Ensembl
**255470**	Dehalococcoides mccartyi CBDB1	DmccCBDB1	48.90	47.85	1456	Chloroflexi	Bacteria	Ensembl
**257314**	Lactobacillus johnsonii NCC 533	Ljoh	34.60	34.96	1819	Firmicutes	Bacteria	Ensembl
**258594**	Rhodopseudomonas palustris CGA009	Rpal	66.00	65.53	4814	Proteobacteria	Bacteria	Ensembl
**259536**	Psychrobacter arcticus 273-4	Parc	42.80	44.59	2119	Proteobacteria	Bacteria	Ensembl
**262768**	Onion yellows phytoplasma OY-M	Oyel	27.80	29.07	744	Tenericutes	Bacteria	Ensembl
**263358**	Verrucosispora maris AB-18-032	Vmar	70.89	71.28	5978	Actinobacteria	Bacteria	Ensembl

TaxId	Species	Nick-name	Ann. GC%	CDS GC%	Num CDSs	Phylum	Domain	Source
**263820**	Picrophilus torridus DSM 9790	Ptor	36.00	37.08	1534	Euryarchaeota	Archaea	Ensembl
**264462**	Bdellovibrio bacteriovorus HD100	Bbac	43.30	51.01	3581	Proteobacteria	Bacteria	Ensembl
**266834**	Sinorhizobium meliloti 1021	Smel	62.16	62.86	6228	Proteobacteria	Bacteria	Ensembl
**266940**	Kineococcus radiotolerans SRS30216 = ATCC BAA-149	Krad	74.21	74.34	4653	Actinobacteria	Bacteria	Ensembl
**267377**	Methanococcus maripaludis S2	MmarS2	33.30	34.01	1712	Euryarchaeota	Archaea	Ensembl
**267608**	Ralstonia solanacearum GMI1000	Rsol	66.96	67.56	5097	Proteobacteria	Bacteria	Ensembl
**267671**	Leptospira interrogans serovar Copenhageni str. Fiocruz L1-130	Lint	35.01	36.68	3658	Spirochaetes	Bacteria	Ensembl
**269084**	Synechococcus elongatus PCC 6301	Selo	55.50	56.13	2485	Cyanobacteria	Bacteria	Ensembl
**269800**	Thermobifida fusca YX	Tfus	67.50	68.13	3107	Actinobacteria	Bacteria	Ensembl
**272557**	Aeropyrum pernix K1	Aper	56.30	56.97	1695	Crenarchaeota	Archaea	Ensembl
**272558**	Bacillus halodurans C-125	Bhal	43.70	44.32	4039	Firmicutes	Bacteria	Ensembl
**272567**	Geobacillus stearothermophilus 10	Gste	52.61	53.68	3303	Firmicutes	Bacteria	Ensembl
**272623**	Lactococcus lactis subsp. lactis Il1403	Llac	35.30	36.18	2258	Firmicutes	Bacteria	Ensembl
**272626**	Listeria innocua Clip11262	Linn	37.35	37.79	3040	Firmicutes	Bacteria	Ensembl
**272631**	Mycobacterium leprae TN	Mlep	57.80	60.12	1605	Actinobacteria	Bacteria	Ensembl
**272632**	Mycoplasma mycoides subsp. mycoides SC str. PG1	Mmyc	24.00	24.09	1012	Tenericutes	Bacteria	Ensembl
**272633**	Mycoplasma penetrans HF-2	Mpen	25.70	26.48	1033	Tenericutes	Bacteria	Ensembl
**272634**	Mycoplasma pneumoniae M129	Mpne	40.00	40.75	688	Tenericutes	Bacteria	Ensembl
**272635**	Mycoplasma pulmonis UAB CTIP	Mpul	26.60	27.29	775	Tenericutes	Bacteria	Ensembl
**272844**	Pyrococcus abyssi GE5	Paby	44.70	45.14	1782	Euryarchaeota	Archaea	Ensembl
**273063**	Sulfolobus tokodaii str. 7	Stok	32.80	33.52	2811	Crenarchaeota	Archaea	Ensembl
**273075**	Thermoplasma acidophilum DSM 1728	Taci1728	46.00	47.28	1478	Euryarchaeota	Archaea	Ensembl
**273116**	Thermoplasma volcanium GSS1	Tvol	39.90	40.99	1525	Euryarchaeota	Archaea	Ensembl
**273121**	Wolinella succinogenes DSM 1740	Wsuc	48.50	48.91	2044	Proteobacteria	Bacteria	Ensembl
**280463**	Emiliania huxleyi CCMP1516	Ehux	64.50	69.09	36050		Eukaryota	Ensembl
**280699**	Cyanidioschyzon merolae	Cmer	55.02	56.72	4951		Eukaryota	Ensembl
**281090**	Leifsonia xyli subsp. xyli str. CTCB07	Lxyl	68.30	68.39	2019	Actinobacteria	Bacteria	Ensembl
**283166**	Bartonella henselae str. Houston-1	Bhen	38.20	40.03	1488	Proteobacteria	Bacteria	Ensembl
**284811**	Eremothecium gossypii ATCC 10895 (assembly ASM9102v4)	Egos	51.69	52.80	4748	Ascomycota	Eukaryota	NCBI
**284812**	Schizosaccharomyces pombe (strain 972 / ATCC 24843)	Spom	36.04	39.61	5141	Ascomycota	Eukaryota	Ensembl
**288705**	Renibacterium salmoninarum	Rsal	56.30	56.61	3505	Actinobacteria	Bacteria	Ensembl

TaxId	Species	Nick-name	Ann. GC%	CDS GC%	Num CDSs	Phylum	Domain	Source
	ATCC 33209							
**289376**	Thermodesulfovibrio yellowstonii DSM 11347	Tyel	34.10	34.17	2030	Nitrospirae	Bacteria	Ensembl
**289377**	Thermodesulfobacterium commune DSM 2178	Tcom	37.00	37.33	1453	Thermodesulfobacteria	Bacteria	Ensembl
**290633**	Gluconobacter oxydans 621H	Goxy	60.84	61.47	2662	Proteobacteria	Bacteria	Ensembl
**295405**	Bacteroides fragilis YCH46	Bfra	43.24	44.16	4414	Bacteroidetes	Bacteria	NCBI
**296543**	Thalassiosira pseudonana	Tpse	46.91	47.95	11061	Bacillariophyta	Eukaryota	Ensembl
**298386**	Photobacterium profundum SS9	Ppro	41.75	42.67	5469	Proteobacteria	Bacteria	Ensembl
**300852**	Thermus thermophilus HB8	TtheHB8	69.49	69.66	2221	Deinococcus-Thermus	Bacteria	Ensembl
**309799**	Dictyoglomus thermophilum H-6-12	Dthe-6-12	33.70	33.81	1908	Dictyoglomi	Bacteria	Ensembl
**309801**	Thermomicrobium roseum DSM 5159	Tros	64.26	64.18	2856	Chloroflexi	Bacteria	Ensembl
**312017**	Tetrahymena thermophila SB210	Tthe	22.30	27.72	24128		Eukaryota	Ensembl
**313596**	Robiginitalea biformata HTCC2501	Rbif	55.30	56.07	3192	Bacteroidetes	Bacteria	Ensembl
**313628**	Lentisphaera araneosa HTCC2155	Lara	41.00	41.63	5042	Lentisphaerae	Bacteria	Ensembl
**314225**	Erythrobacter litoralis HTCC2594	Elit	63.10	63.43	3000	Proteobacteria	Bacteria	Ensembl
**314260**	Parvularcula bermudensis HTCC2503	Pber	60.70	60.96	2677	Proteobacteria	Bacteria	Ensembl
**314278**	Nitrococcus mobilis Nb-231	Nmob	59.90	60.75	3482	Proteobacteria	Bacteria	Ensembl
**316274**	Herpetosiphon aurantiacus DSM 785	Haur	50.89	51.41	5278	Chloroflexi	Bacteria	Ensembl
**316279**	Synechococcus sp. CC9902	SCC9	54.20	54.87	2302	Cyanobacteria	Bacteria	Ensembl
**316407**	Escherichia coli str. K-12 substr. W3110	EcolW3110	50.45	51.90	4222	Proteobacteria	Bacteria	Ensembl
**319795**	Deinococcus geothermalis DSM 11300 str. DSM11300	Dgeo	66.57	66.86	3051	Deinococcus-Thermus	Bacteria	Ensembl
**322098**	Aster yellows witches'-broom phytoplasma AYWB	Ayel	26.83	28.41	683	Tenericutes	Bacteria	Ensembl
**324602**	Chloroflexus aurantiacus J-10-fl	Caur	56.70	57.13	3852	Chloroflexi	Bacteria	Ensembl
**326298**	Sulfurimonas denitrificans DSM 1251	Sden	34.50	34.78	2096	Proteobacteria	Bacteria	Ensembl
**326427**	Chloroflexus aggregans DSM 9485	Cagg	56.40	56.77	3730	Chloroflexi	Bacteria	Ensembl
**330214**	Nitrospira defluvii	Ndef	59.00	59.27	4262	Nitrospirae	Bacteria	Ensembl
**331104**	Blattabacterium sp. (Blattella germanica) str. Bge	BBge	23.84	27.25	589	Bacteroidetes	Bacteria	Ensembl
**331113**	Simkania negevensis Z	Sneg	41.62	42.26	2466	Chlamydiae	Bacteria	Ensembl
**333146**	Ferroplasma acidarmanus fer1	Faci	36.50	37.56	1942	Euryarchaeota	Archaea	Ensembl
**335284**	Psychrobacter cryohalolentis K5	Pcry	42.25	43.98	2511	Proteobacteria	Bacteria	Ensembl
**336722**	Zymoseptoria tritici	Ztri	52.12	55.56	10780	Ascomycota	Eukaryota	Ensembl

TaxId	Species	Nick-name	Ann. GC%	CDS GC%	Num CDSs	Phylum	Domain	Source
**339860**	Methanosphaera stadtmanae DSM 3091	Msta	27.60	29.10	1507	Euryarchaeota	Archaea	Ensembl
**345663**	Chryseobacterium greenlandense	Cgre	34.10	35.10	3587	Bacteroidetes	Bacteria	Ensembl
**347257**	Mycoplasma agalactiae PG2	Maga	29.70	30.11	751	Tenericutes	Bacteria	Ensembl
**347515**	Leishmania major strain Friedlin	Lmaj	59.71	62.45	8299		Eukaryota	Ensembl
**349741**	Akkermansia muciniphila ATCC BAA-835	Amuc	55.80	56.76	2137	Verrucomicrobi a	Bacteria	Ensembl
**351607**	Acidothermus cellulolyticus 11B	Acel	66.90	66.76	2156	Actinobacteria	Bacteria	Ensembl
**352472**	Dictyostelium discoideum AX4	Ddis	22.46	27.40	12859		Eukaryota	Ensembl
**353152**	Cryptosporidium parvum Iowa II	CparII	30.25	31.88	3761	Apicomplexa	Eukaryota	Ensembl
**353154**	Theileria annulata strain Ankara	Tann	32.55	35.72	3792	Apicomplexa	Eukaryota	Ensembl
**358681**	Brevibacillus brevis NBRC 100599	Bbre	47.30	47.88	5934	Firmicutes	Bacteria	Ensembl
**360911**	Exiguobacterium sp. AT1b	EAT1	48.50	49.10	3015	Firmicutes	Bacteria	Ensembl
**362976**	Haloquadratum walsbyi DSM 16790	Hwal	47.69	48.75	2548	Euryarchaeota	Archaea	Ensembl
**365046**	Ramlibacter tataouinensis TTB310	Rtat	70.00	70.36	3854	Proteobacteria	Bacteria	Ensembl
**373903**	Halothermothrix orenii H 168	Hore	37.90	38.89	2341	Firmicutes	Bacteria	Ensembl
**374847**	Candidatus Korarchaeum cryptofilum OPF8	Kcry	49.00	49.54	1602	Candidatus Korarchaeota	Archaea	Ensembl
**379066**	Gemmatimonas aurantiaca T-27	Gaur	64.30	64.49	3934	Gemmatimonad etes	Bacteria	Ensembl
**381306**	Thiohalorhabdus denitrificans	Tden	68.90	69.71	2403	Proteobacteria	Bacteria	Ensembl
**381764**	Fervidobacterium nodosum Rt17-B1	Fnod	35.00	35.23	1746	Thermotogae	Bacteria	Ensembl
**383372**	Roseiflexus castenholzii DSM 13941	Rcas	60.70	60.94	4330	Chloroflexi	Bacteria	Ensembl
**388396**	Vibrio fischeri MJ11	Vfis	38.37	38.85	4039	Proteobacteria	Bacteria	Ensembl
**391009**	Thermosipho melanesiensis BI429	Tmel	31.40	31.23	1875	Thermotogae	Bacteria	Ensembl
**391165**	Granulibacter bethesdensis CGDNIH1	Gbet	59.10	59.62	2435	Proteobacteria	Bacteria	Ensembl
**391603**	Flavobacteriales bacterium ALC-1	FALC	32.40	32.87	3428	Bacteroidetes	Bacteria	Ensembl
**391623**	Thermococcus barophilus MP	Tbar	41.71	42.08	2173	Euryarchaeota	Archaea	Ensembl
**393595**	Alcanivorax borkumensis SK2	Abor	54.70	55.24	2755	Proteobacteria	Bacteria	Ensembl
**398720**	Leeuwenhoekiella blandensis MED217	Lbla	39.80	40.39	3715	Bacteroidetes	Bacteria	Ensembl
**398767**	Geobacter lovleyi SZ	Glov	54.77	55.33	3200	Proteobacteria	Bacteria	Ensembl
**400667**	Acinetobacter baumannii ATCC 17978	Abau	39.00	40.13	3826	Proteobacteria	Bacteria	Ensembl
**400682**	Amphimedon queenslandica	Aque	37.50	41.36	27593	Porifera	Eukaryota	Ensembl
**402612**	Flavobacterium psychrophilum JIP02/86	Fpsy	32.50	33.24	2397	Bacteroidetes	Bacteria	Ensembl

TaxId	Species	Nick-name	Ann. GC%	CDS GC%	Num CDSs	Phylum	Domain	Source
**402881**	Parvibaculum lavamentivorans DS-1	Plav	62.30	62.74	3635	Proteobacteria	Bacteria	Ensembl
**403833**	Petrotoga mobilis SJ95	Pmob	34.10	34.20	1896	Thermotogae	Bacteria	Ensembl
**405948**	Saccharopolyspora erythraea NRRL 2338	Sery	71.10	71.60	7164	Actinobacteria	Bacteria	Ensembl
**407035**	Salinicoccus halodurans	Shal	44.50	45.55	2643	Firmicutes	Bacteria	Ensembl
**410358**	Methanocorpusculum labreanum Z	Mlab	50.00	51.10	1738	Euryarchaeota	Archaea	Ensembl
**411154**	Gramella forsetii KT0803	Gfor	36.60	37.26	3573	Bacteroidetes	Bacteria	Ensembl
**412030**	Paramecium tetraurelia strain d4-2	Ptet	28.20	30.13	39433		Eukaryota	Ensembl
**412133**	Trichomonas vaginalis G3	Tvag	32.90	35.55	56271		Eukaryota	Ensembl
**414004**	Cenarchaeum symbiosum A	Csym	57.40	57.79	2010	Thaumarchaeota	Archaea	Ensembl
**418459**	Puccinia graminis f. sp. tritici	Pgra	43.80	49.67	15958	Basidiomycota	Eukaryota	Ensembl
**419610**	Methylobacterium extorquens PA1	Mext	68.20	69.02	4819	Proteobacteria	Bacteria	Ensembl
**420247**	Methanobrevibacter smithii ATCC 35061	Msmi	31.00	32.05	1731	Euryarchaeota	Archaea	Ensembl
**420778**	Diplodia seriata	Dser	56.50	60.75	9343	Ascomycota	Eukaryota	Ensembl
**420890**	Lactococcus garvieae Lg2	Lgar	38.80	39.63	1963	Firmicutes	Bacteria	Ensembl
**423536**	Perkinsus marinus ATCC 50983	Pmar50983	47.40	51.21	20630		Eukaryota	Ensembl
**429572**	Sulfolobus islandicus L.S.2.15	Sisl	35.10	35.57	2735	Crenarchaeota	Archaea	Ensembl
**431895**	Monosiga brevicollis MX1	Mbre	54.33	57.25	9049		Eukaryota	Ensembl
**431947**	Porphyromonas gingivalis ATCC 33277	Pgin	48.40	49.41	2082	Bacteroidetes	Bacteria	Ensembl
**432331**	Sulfurihydrogenibium yellowstonense SS-5	Syel	32.80	32.69	1570	Aquificae	Bacteria	Ensembl
**435906**	Salegentibacter salarius	Ssal	37.00	37.75	2932	Bacteroidetes	Bacteria	Ensembl
**436017**	Ostreococcus lucimarinus	Oluc	60.44	59.01	7571	Chlorophyta	Eukaryota	Ensembl
**436308**	Nitrosopumilus maritimus SCM1	Nmar	34.20	34.59	1792	Thaumarchaeota	Archaea	Ensembl
**436907**	Vanderwaltozyma polyspora DSM 70294	Vpol	33.00	34.95	5332	Ascomycota	Eukaryota	Ensembl
**439292**	Bacillus selenitireducens MLS10	Bsel	48.70	49.43	2819	Firmicutes	Bacteria	Ensembl
**441768**	Acholeplasma laidlawii PG-8A	Alai	31.90	32.23	1377	Tenericutes	Bacteria	Ensembl
**443254**	Marinitoga piezophila KA3	Mpie	29.18	29.10	2034	Thermotogae	Bacteria	Ensembl
**443906**	Clavibacter michiganensis subsp. michiganensis NCPPB 382	Cmic	72.42	72.71	3059	Actinobacteria	Bacteria	Ensembl
**445932**	Elusimicrobium minutum Pei191	Emin	40.00	40.69	1526	Elusimicrobia	Bacteria	Ensembl
**446470**	Stackebrandtia nassauensis DSM 44728	Snas	68.10	68.66	6366	Actinobacteria	Bacteria	Ensembl
**449447**	Microcystis aeruginosa NIES-843	Maer	42.30	42.90	6306	Cyanobacteria	Bacteria	Ensembl
**452637**	Opitutus terrae PB90-1	Oter	65.30	65.47	4610	Verrucomicrobia	Bacteria	Ensembl

TaxId	Species	Nick-name	Ann. GC%	CDS GC%	Num CDSs	Phylum	Domain	Source
**452652**	Kitasatospora setae KM-6054	Kset	74.20	74.44	7477	Actinobacteria	Bacteria	Ensembl
**456481**	Leptospira biflexa serovar Patoc strain 'Patoc 1 (Paris)'	Lbif	38.90	39.07	2678	Spirochaetes	Bacteria	
**457570**	Natranaerobius thermophilus JW/NM-WN-LF	Nthe	36.29	36.77	2903	Firmicutes	Bacteria	Ensembl
**469371**	Thermobispora bispora DSM 43833	Tbis	72.40	72.48	3535	Actinobacteria	Bacteria	Ensembl
**469382**	Halogeometricum borinquense DSM 11551	Hbor	59.97	61.05	3890	Euryarchaeota	Archaea	Ensembl
**469383**	Conexibacter woesei DSM 14684	Cwoe	72.40	72.93	5902	Actinobacteria	Bacteria	Ensembl
**469599**	Fusobacterium periodonticum 2_1_31	Fper	28.60	28.28	2327	Fusobacteria	Bacteria	Ensembl
**469615**	Fusobacterium gonidiaformans ATCC 25563	Fgon	32.90	32.79	1600	Fusobacteria	Bacteria	Ensembl
**476282**	Bradyrhizobium japonicum SEMIA 5079	Bjap	63.70	64.41	8646	Proteobacteria	Bacteria	Ensembl
**477974**	Candidatus Desulforudis audaxviator MP104C	Daud	60.80	62.05	2157	Firmicutes	Bacteria	Ensembl
**478009**	Halobacterium salinarum R1	HsalR1	65.92	66.81	2701	Euryarchaeota	Archaea	Ensembl
**479433**	Catenulispora acidiphila DSM 44928	Caci	69.80	70.24	8884	Actinobacteria	Bacteria	Ensembl
**479434**	Sphaerobacter thermophilus DSM 20745	Sthe	68.10	68.34	3484	Chloroflexi	Bacteria	Ensembl
**481448**	Methylacidiphilum infernorum V4	Minf	45.50	45.85	2451	Verrucomicrobi a	Bacteria	Ensembl
**484019**	Thermosipho africanus TCF52B	Tafr	30.80	30.73	1954	Thermotogae	Bacteria	Ensembl
**484906**	Babesia bovis T2Bo	Bbov	41.61	43.87	3699	Apicomplexa	Eukaryota	Ensembl
**485913**	Ktedonobacter racemifer DSM 44963	Krac	53.80	55.11	11437	Chloroflexi	Bacteria	Ensembl
**486041**	Laccaria bicolor S238N-H82	Lbic	47.10	50.56	18172	Basidiomycota	Eukaryota	Ensembl
**491915**	Anoxybacillus flavithermus WK1	Afla	41.80	42.02	2824	Firmicutes	Bacteria	Ensembl
**498848**	Thermus aquaticus Y51MC23	Taqu	68.04	68.36	2521	Deinococcus-Thermus	Bacteria	Ensembl
**500635**	Mitsuokella multacida DSM 20544	Mmul	58.00	59.41	2541	Firmicutes	Bacteria	Ensembl
**504728**	Meiothermus ruber DSM 1279	Mrub	63.40	64.12	3014	Deinococcus-Thermus	Bacteria	Ensembl
**505682**	Ureaplasma parvum serovar 3 str. ATCC 27815	Upar	25.50	25.69	609	Tenericutes	Bacteria	
**507754**	Acidiplasma aeolicum str. VT	Aaeo	34.20	35.21	1663	Euryarchaeota	Archaea	Ensembl
**508771**	Toxoplasma gondii ME49	Tgon	52.29	58.10	7917	Apicomplexa	Eukaryota	Ensembl
**511051**	Caldisericum exile AZM16c01	Cexi	35.40	35.51	1578	Caldiserica	Bacteria	Ensembl
**511145**	Escherichia coli str. K-12 substr. MG1655	EcolG165 5	50.45	51.97	4031	Proteobacteria	Bacteria	NCBI
**515635**	Dictyoglomus turgidum DSM 6724	Dtur	34.00	33.99	1744	Dictyoglomi	Bacteria	Ensembl
**517417**	Chlorobaculum parvum NCIB 8327	Cpar	55.80	57.18	2042	Chlorobi	Bacteria	Ensembl
**517418**	Chloroherpeton thalassium	Ctha	45.00	46.14	2709	Chlorobi	Bacteria	Ensembl

TaxId	Species	Nick-name	Ann. GC%	CDS GC%	Num CDSs	Phylum	Domain	Source
	ATCC 35110							
**518766**	Rhodothermus marinus DSM 4252	Rmar	64.27	65.07	2860	Bacteroidetes	Bacteria	Ensembl
**519441**	Streptobacillus moniliformis DSM 12112	Smon	26.27	26.16	1420	Fusobacteria	Bacteria	Ensembl
**521011**	Methanosphaerula palustris E1-9c	Mpal	55.40	56.79	2650	Euryarchaeota	Archaea	Ensembl
**521045**	Kosmotoga olearia TBF 19.5.1	Kole	41.50	41.55	2115	Thermotogae	Bacteria	Ensembl
**521097**	Capnocytophaga ochracea DSM 7271	Coch	39.60	40.57	2164	Bacteroidetes	Bacteria	Ensembl
**521674**	Planctopirus limnophila DSM 3776	Plim	53.72	54.43	4258	Planctomycetes	Bacteria	Ensembl
**522772**	Denitrovibrio acetiphilus DSM 12809	Dace	42.50	43.20	2964	Deferribacteres	Bacteria	Ensembl
**523841**	Haloferax mediterranei ATCC 33500	Hmed	60.26	61.67	3825	Euryarchaeota	Archaea	Ensembl
**525903**	Thermanaerovibrio acidaminovorans DSM 6589	Taci	63.80	64.38	1733	Synergistetes	Bacteria	Ensembl
**525904**	Thermobaculum terrenum ATCC BAA-798	Tter	53.54	53.82	2832		Bacteria	Ensembl
**525909**	Acidimicrobium ferrooxidans DSM 10331	Afer	68.30	68.37	1963	Actinobacteria	Bacteria	Ensembl
**525919**	Anaerococcus prevotii DSM 20548	Apre	35.67	36.09	1801	Firmicutes	Bacteria	Ensembl
**526218**	Sebaldella termitidis ATCC 33386	Ster	33.42	34.62	4128	Fusobacteria	Bacteria	Ensembl
**526224**	Brachyspira murdochii DSM 12563	Bmur	27.80	29.00	2800	Spirochaetes	Bacteria	Ensembl
**543302**	Alicyclobacillus acidocaldarius LAA1	Aaci	61.86	62.32	3006	Firmicutes	Bacteria	Ensembl
**547144**	Hydrogenobaculum sp. HO	HHO	34.80	34.88	1577	Aquificae	Bacteria	Ensembl
**548479**	Mobiluncus curtisii ATCC 43063	Mcur	55.40	55.89	1841	Actinobacteria	Bacteria	Ensembl
**552811**	Dehalogenimonas lykanthroporepellens BL-DC-9	Dlyk	55.00	55.99	1655	Chloroflexi	Bacteria	Ensembl
**553190**	Gardnerella vaginalis 409-05	Gvag	42.00	42.77	1258	Actinobacteria	Bacteria	Ensembl
**554373**	Moniliophthora perniciosa FA553	Mper	47.70	49.78	9748	Basidiomycota	Eukaryota	Ensembl
**555500**	Galbibacter marinus	Gmar	37.00	37.90	3079	Bacteroidetes	Bacteria	Ensembl
**555778**	Halothiobacillus neapolitanus c2	Hnea	54.70	55.49	2354	Proteobacteria	Bacteria	Ensembl
**555779**	Desulfonatronospira thiodismutans ASO3-1	Dthi	51.30	52.52	3660	Proteobacteria	Bacteria	Ensembl
**556484**	Phaeodactylum tricornutum CCAP 1055/1	Ptri	48.84	50.96	12172	Bacillariophyta	Eukaryota	JGI
**559292**	Saccharomyces cerevisiae S288c	Scer	38.16	39.67	5787	Ascomycota	Eukaryota	YeastGenome (SGD)
**561896**	Postia placenta Mad-698-R	Ppla	52.70	56.71	8904	Basidiomycota	Eukaryota	Ensembl
**564608**	Micromonas pusilla CCMP1545	Mpus	65.70	67.40	10615	Chlorophyta	Eukaryota	JGI
**572478**	Vulcanisaeta distributa DSM 14429	Vdis	45.40	46.26	2491	Crenarchaeota	Archaea	Ensembl

TaxId	Species	Nick-name	Ann. GC%	CDS GC%	Num CDSs	Phylum	Domain	Source
**572544**	Ilyobacter polytropus DSM 2926	Ipol	34.36	35.28	2870	Fusobacteria	Bacteria	Ensembl
**573065**	Asticcacaulis excentricus CB 48	Aexc	59.53	60.39	3761	Proteobacteria	Bacteria	Ensembl
**574087**	Acetohalobium arabaticum DSM 5501	Aara	36.60	37.34	2278	Firmicutes	Bacteria	Ensembl
**574566**	Coccomyxa subellipsoidea C-169	Csub	52.90	61.34	9603	Chlorophyta	Eukaryota	JGI
**575540**	Isosphaera pallida ATCC 43644	Ipal	62.45	63.04	3722	Planctomycetes	Bacteria	Ensembl
**578458**	Schizophyllum commune H4-8	Scom	57.40	60.03	13171	Basidiomycota	Eukaryota	Ensembl
**578462**	Allomyces macrogynus ATCC 38327	Amac	60.50	64.94	16745	Blastocladiomycota	Eukaryota	Ensembl
**580340**	Thermovirga lienii DSM 17291	Tlie	47.10	47.43	1874	Synergistetes	Bacteria	Ensembl
**582515**	Rubidibacter lacunae KORDI 51-2	Rlac	56.20	57.45	3411	Cyanobacteria	Bacteria	Ensembl
**583355**	Coraliomargarita akajimensis DSM 45221	Caka	53.60	53.93	3118	Verrucomicrobia	Bacteria	Ensembl
**583356**	Ignisphaera aggregans DSM 17230	Iagg	35.70	36.01	1927	Crenarchaeota	Archaea	Ensembl
**585394**	Roseburia hominis A2-183	Rhom	48.50	49.34	3351	Firmicutes	Bacteria	Ensembl
**589924**	Ferroglobus placidus DSM 10642	Fpla	44.10	44.71	2478	Euryarchaeota	Archaea	Ensembl
**592010**	Abiotrophia defectiva ATCC 49176	Adef	47.00	47.60	1943	Firmicutes	Bacteria	Ensembl
**592029**	Nonlabens dokdonensis DSW-6	Ndok	35.30	35.94	3613	Bacteroidetes	Bacteria	Ensembl
**593117**	Thermococcus gammatolerans EJ3	Tgam	53.60	54.14	2156	Euryarchaeota	Archaea	Ensembl
**595528**	Capsaspora owczarzaki ATCC 30864	Cowc	53.70	58.01	8627		Eukaryota	Ensembl
**596323**	Leptotrichia goodfellowii F0264	Lgoo	31.60	32.20	2266	Fusobacteria	Bacteria	Ensembl
**608538**	Hydrogenobacter thermophilus TK-6	Hthe	44.00	44.13	1894	Aquificae	Bacteria	Ensembl
**633147**	Olsenella uli DSM 7084	Ouli	64.70	65.18	1735	Actinobacteria	Bacteria	Ensembl
**633149**	Brevundimonas subvibrioides ATCC 15264	Bsub15264	68.40	68.81	3243	Proteobacteria	Bacteria	Ensembl
**635003**	Fragilariopsis cylindrus CCMP1102	Fcyl	39.00	41.66	2790	Bacillariophyta	Eukaryota	NCBI
**638303**	Thermocrinis albus DSM 14484	Talb	46.90	47.01	1593	Aquificae	Bacteria	Ensembl
**639282**	Deferribacter desulfuricans SSM1	Ddes	30.30	30.48	2374	Deferribacteres	Bacteria	Ensembl
**641526**	Winogradskyella psychrotolerans RS-3	Wpsy	33.50	34.03	4001	Bacteroidetes	Bacteria	Ensembl
**642492**	Clostridium lentocellum DSM 5427	Clen	34.30	34.83	4166	Firmicutes	Bacteria	Ensembl
**644295**	Methanohalobium evestigatum Z-7303	Meve	36.40	37.58	2251	Euryarchaeota	Archaea	Ensembl
**645134**	Spizellomyces punctatus DAOM BR117	Spun	47.60	49.84	9421	Chytridiomycota	Eukaryota	Ensembl
**648996**	Thermovibrio ammonificans	Tamm	52.12	52.26	1812	Aquificae	Bacteria	Ensembl

TaxId	Species	Nick-name	Ann. GC%	CDS GC%	Num CDSs	Phylum	Domain	Source
	HB-1							
**649638**	Truepera radiovictrix DSM 17093	Trad	68.10	68.71	2940	Deinococcus-Thermus	Bacteria	Ensembl
**651182**	Desulfobacula toluolica Tol2	Dtol	41.40	42.28	4374	Proteobacteria	Bacteria	Ensembl
**653733**	Desulfurispirillum indicum S5	Dind	56.10	56.80	2570	Chrysiogenetes	Bacteria	Ensembl
**655815**	Zunongwangia profunda SM-A87	Zpro	36.20	37.10	4617	Bacteroidetes	Bacteria	Ensembl
**660470**	Mesotoga prima MesG1.Ag.4.2	Mpri	45.50	45.70	2565	Thermotogae	Bacteria	Ensembl
**661478**	Fimbriimonas ginsengisoli Gsoil 348	Fgin	60.80	61.32	4819	Armatimonadetes	Bacteria	Ensembl
**667014**	Thermodesulfatator indicus DSM 15286	Tind	42.40	42.61	2195	Thermodesulfobacteria	Bacteria	Ensembl
**670487**	Oceanithermus profundus DSM 14977	Opro	69.79	70.31	2370	Deinococcus-Thermus	Bacteria	Ensembl
**691883**	Fonticula alba	Falb	64.30	68.38	6306		Eukaryota	Ensembl
**694429**	Pyrolobus fumarii 1A	Pfum	54.90	54.95	1967	Crenarchaeota	Archaea	Ensembl
**695850**	Saprolegnia parasitica CBS 223.65	Spar	57.50	62.29	19578		Eukaryota	Ensembl
**696747**	Arthrospira platensis NIES-39	Apla	44.30	44.57	6625	Cyanobacteria	Bacteria	Ensembl
**703613**	Bifidobacterium animalis subsp. animalis ATCC 25527	Bani	60.50	61.40	1537	Actinobacteria	Bacteria	Ensembl
**742818**	Slackia piriformis YIT 12062	Spir	57.60	58.19	1792	Actinobacteria	Bacteria	Ensembl
**743299**	Acidithiobacillus ferrivorans SS3	AferSS3	56.60	57.27	3090	Proteobacteria	Bacteria	Ensembl
**743718**	Isoptericola variabilis 225	Ivar	73.90	74.05	2868	Actinobacteria	Bacteria	Ensembl
**744533**	Naegleria gruberi strain NEG-M	Ngru	35.00	34.47	15571		Eukaryota	Ensembl
**746697**	Aequorivita sublithincola DSM 14238	Asub	36.20	36.90	3137	Bacteroidetes	Bacteria	Ensembl
**751945**	Thermus oshimai JL-2	Tosh	68.60	68.84	2119	Deinococcus-Thermus	Bacteria	Ensembl
**753081**	Bigelowiella natans	Bnat	44.90	49.10	21512		Eukaryota	Ensembl
**754035**	Mesorhizobium australicum WSM2073	Maus	65.00	63.48	5786	Proteobacteria	Bacteria	Ensembl
**755732**	Fluviicola taffensis DSM 16823	Ftaf	36.50	36.96	4030	Bacteroidetes	Bacteria	Ensembl
**760142**	Hippea maritima DSM 10411	Hmar10411	37.50	37.48	1675	Proteobacteria	Bacteria	Ensembl
**762948**	Rothia dentocariosa ATCC 17931	Rden	53.70	54.79	2213	Actinobacteria	Bacteria	Ensembl
**762983**	Succinatimonas hippei YIT 12066	Ship	40.30	41.31	2148	Proteobacteria	Bacteria	Ensembl
**765420**	Oscillochloris trichoides DG-6	Otri	59.10	60.04	3231	Chloroflexi	Bacteria	Ensembl
**765952**	Parachlamydia acanthamoebae UV-7	Paca	39.00	39.73	2544	Chlamydiae	Bacteria	Ensembl
**767434**	Frateuria aurantia DSM 6220	Faur	63.40	63.85	3097	Proteobacteria	Bacteria	Ensembl
**768670**	Calditerrivibrio nitroreducens DSM 19672	Cnit	35.68	35.92	2099	Deferribacteres	Bacteria	Ensembl
**768671**	Thiocapsa marina 5811	Tmar5811	64.10	64.57	4893	Proteobacteria	Bacteria	Ensembl

TaxId	Species	Nick-name	Ann. GC%	CDS GC%	Num CDSs	Phylum	Domain	Source
**768679**	Thermoproteus tenax Kra 1	Tten	55.10	55.57	2048	Crenarchaeota	Archaea	Ensembl
**768706**	Desulfosporosinus orientis DSM 765	Dori	42.90	43.71	5232	Firmicutes	Bacteria	Ensembl
**795359**	Thermodesulfobacterium geofontis OPF15	Tgeo	30.60	30.67	1593	Thermodesulfob acteria	Bacteria	Ensembl
**797114**	Halosimplex carlsbadense 2-9-1	Hcar	67.70	68.81	4390	Euryarchaeota	Archaea	Ensembl
**797210**	Halopiger xanaduensis SH-6	Hxan	65.20	66.33	4205	Euryarchaeota	Archaea	Ensembl
**797304**	Natronobacterium gregoryi SP2	Ngre	62.20	63.19	3650	Euryarchaeota	Archaea	Ensembl
**859192**	Candidatus Nitrosoarchaeum limnia BG20	Nlim	32.50	33.08	2434	Thaumarchaeot a	Archaea	Ensembl
**861299**	Gemmatirosa kalamazoonesis	Gkal	72.64	72.88	6105	Gemmatimonad etes	Bacteria	Ensembl
**862908**	Halobacteriovorax marinus SJ	Hmar	36.70	37.01	2787	Proteobacteria	Bacteria	Ensembl
**866499**	Cloacibacillus evryensis DSM 19522	Cevr	56.00	58.05	1082	Synergistetes	Bacteria	Ensembl
**866895**	Halobacillus halophilus DSM 2266	Hhal	41.80	42.42	4108	Firmicutes	Bacteria	Ensembl
**867904**	Methanomethylovorans hollandica DSM 15978	Mhol	41.84	43.15	2554	Euryarchaeota	Archaea	Ensembl
**868864**	Desulfurobacterium thermolithotrophum DSM 11699	Dthe	34.90	34.75	1507	Aquificae	Bacteria	Ensembl
**869210**	Marinithermus hydrothermalis DSM 14884	Mhyd	68.10	68.53	2202	Deinococcus-Thermus	Bacteria	Ensembl
**880073**	Caldithrix abyssi DSM 13497	Caby	45.10	46.13	3746	Calditrichaeota	Bacteria	Ensembl
**883169**	Turicella otitidis ATCC 51513	Toti	71.00	71.26	1445	Actinobacteria	Bacteria	Ensembl
**885318**	Entamoeba histolytica HM-1:IMSS-A	Ehis	24.30	27.67	5998		Eukaryota	Ensembl
**886293**	Singulisphaera acidiphila DSM 18658	Saci	62.27	63.26	7248	Planctomycetes	Bacteria	Ensembl
**886377**	Muricauda ruestringensis DSM 13258	Mrue	41.40	42.09	3428	Bacteroidetes	Bacteria	Ensembl
**891968**	Anaerobaculum mobile DSM 13181	Amob	48.00	48.55	2013	Synergistetes	Bacteria	Ensembl
**903503**	Candidatus Moranella endobia PCIT	Mend	43.50	45.25	406	Proteobacteria	Bacteria	Ensembl
**905079**	Guillardia theta CCMP2712	Gthe	52.90	54.77	24237		Eukaryota	Ensembl
**910314**	Dialister microaerophilus UPII 345-E	Dmic	35.60	36.43	1298	Firmicutes	Bacteria	Ensembl
**911008**	Leclercia adecarboxylata ATCC 23216 = NBRC 102595	Lade	55.80	56.85	4592	Proteobacteria	Bacteria	Ensembl
**926550**	Caldilinea aerophila DSM 14535 = NBRC 104270	Caer	58.80	59.99	4119	Chloroflexi	Bacteria	Ensembl
**926559**	Joostella marina DSM 19592	Jmar	33.60	34.26	3848	Bacteroidetes	Bacteria	Ensembl
**926562**	Owenweeksia hongkongensis DSM 17368	Ohon	40.20	40.69	3485	Bacteroidetes	Bacteria	Ensembl
**926569**	Anaerolinea thermophila UNI-1	Athe	53.80	54.37	3167	Chloroflexi	Bacteria	Ensembl
**926571**	Nitrososphaera viennensis EN76	Nvie	52.70	54.07	3099	Thaumarchaeot a	Archaea	Ensembl

TaxId	Species	Nick-name	Ann. GC%	CDS GC%	Num CDSs	Phylum	Domain	Source
**929556**	Solitalea canadensis DSM 3403	Scan	37.30	38.07	4302	Bacteroidetes	Bacteria	Ensembl
**930946**	Fructobacillus fructosus KCTC 3544	Ffru	44.60	45.56	1439	Firmicutes	Bacteria	Ensembl
**930990**	Botryobasidium botryosum FD-172 SS1	Bbot	52.30	55.43	16391	Basidiomycota	Eukaryota	Ensembl
**931890**	Eremothecium cymbalariae DBVPG#7215	Ecym	40.32	41.38	4432	Ascomycota	Eukaryota	Ensembl
**937777**	Deinococcus peraridilitoris DSM 19664	Dper	63.71	64.41	4176	Deinococcus-Thermus	Bacteria	Ensembl
**944289**	Gymnopus luxurians FD-317 M1	Glux	45.10	48.37	14499	Basidiomycota	Eukaryota	Ensembl
**945553**	Hypholoma sublateritium FD-334 SS-4	Hsub	51.00	54.60	17010	Basidiomycota	Eukaryota	Ensembl
**945713**	Ignavibacterium album JCM 16511	Ialb	33.90	34.31	3188	Ignavibacteriae	Bacteria	Ensembl
**946077**	Imtechella halotolerans K1	Ihal	35.50	36.13	2687	Bacteroidetes	Bacteria	Ensembl
**946362**	Salpingoeca rosetta	Salosetta	55.50	60.40	11648		Eukaryota	Ensembl
**983544**	Lacinutrix sp. 5H-3-7-4	L5H-	30.80	31.35	2963	Bacteroidetes	Bacteria	Ensembl
**997884**	Bacteroides nordii	Bnor	40.80	41.80	4275	Bacteroidetes	Bacteria	Ensembl
**999415**	Eggerthia catenaformis OT 569 = DSM 20559	Ecat	32.80	32.70	1861	Firmicutes	Bacteria	Ensembl
**1002672**	Candidatus Pelagibacter sp. IMCC9063	PIMC	31.70	31.86	1443	Proteobacteria	Bacteria	Ensembl
**1006000**	Kluyvera ascorbata ATCC 33433	Kasc	54.30	55.69	4561	Proteobacteria	Bacteria	Ensembl
**1009370**	Acetonema longum DSM 6540	Alon	50.40	51.42	4197	Firmicutes	Bacteria	Ensembl
**1028800**	Neorhizobium galegae bv. orientalis str. HAMBI 540	Ngal	61.25	62.00	6163	Proteobacteria	Bacteria	Ensembl
**1033802**	Salinisphaera shabanensis E1L3A	Ssha	61.60	62.04	3515	Proteobacteria	Bacteria	Ensembl
**1033810**	Haloplasma contractile SSD-17B	Hcon	32.30	33.41	3017		Bacteria	Ensembl
**1033991**	Rhizobium leguminosarum bv. trifolii CB782	Rleg	61.17	61.84	6480	Proteobacteria	Bacteria	Ensembl
**1041607**	Wickerhamomyces ciferrii	Wcif	30.40	30.81	6702	Ascomycota	Eukaryota	Ensembl
**1046627**	Bizionia argentinensis JUB59	Barg	33.80	34.56	3088	Bacteroidetes	Bacteria	Ensembl
**1047168**	Zymoseptoria brevis	Zbre	51.20	55.67	10475	Ascomycota	Eukaryota	Ensembl
**1055104**	Cobetia amphilecti str. KMM 296	Camp	62.50	63.51	2704	Proteobacteria	Bacteria	Ensembl
**1056495**	Caldisphaera lagunensis DSM 15908	Clag	30.00	30.78	1475	Crenarchaeota	Archaea	Ensembl
**1069680**	Pneumocystis murina b123	Pmur	27.00	30.91	3602	Ascomycota	Eukaryota	Ensembl
**1072681**	Candidatus Haloredivivus sp. G17	HG17	42.00	42.70	1863	Candidatus Nanohaloarchaeota	Archaea	Ensembl
**1116230**	Wolbachia pipientis wAlbB	Wpip	33.80	34.36	961	Proteobacteria	Bacteria	Ensembl

TaxId	Species	Nick-name	Ann. GC%	CDS GC%	Num CDSs	Phylum	Domain	Source
**1121088**	Bacillus coagulans DSM 1 = ATCC 7050	Bcoa	46.90	47.65	3236	Firmicutes	Bacteria	Ensembl
**1121915**	Geoalkalibacter ferrihydriticus DSM 17813	Gfer	57.90	58.86	2897	Proteobacteria	Bacteria	Ensembl
**1123384**	Pseudothermotoga hypogea DSM 11164 = NBRC 106472	Phyp	49.50	49.63	2094	Thermotogae	Bacteria	Ensembl
**1125630**	Klebsiella pneumoniae subsp. pneumoniae HS11286	Kpne	57.14	58.25	5378	Proteobacteria	Bacteria	Ensembl
**1129897**	Nitrolancea hollandica Lb	Nhol	62.60	62.93	3954	Chloroflexi	Bacteria	Ensembl
**1142394**	Phycisphaera mikurensis NBRC 102666	Pmik	73.23	73.13	3283	Planctomycetes	Bacteria	Ensembl
**1157490**	Tumebacillus flagellatus	Tfla	56.50	57.75	4434	Firmicutes	Bacteria	Ensembl
**1165094**	Richelia intracellularis HH01	Rint	33.70	38.26	2258	Cyanobacteria	Bacteria	Ensembl
**1172194**	Hydrocarboniphaga effusa AP103	Heff	65.20	65.72	4680	Proteobacteria	Bacteria	Ensembl
**1177928**	Thalassospira profundimaris WP0211	Tpro	55.20	55.94	4034	Proteobacteria	Bacteria	Ensembl
**1177931**	Thiovulum sp. ES	TES	33.00	33.25	2022	Proteobacteria	Bacteria	Ensembl
**1182568**	Deinococcus puniceus	Dpun	62.60	63.72	2336	Deinococcus-Thermus	Bacteria	Ensembl
**1183438**	Gloeobacter kilaueensis JS1	Gkil	60.50	61.37	4395	Cyanobacteria	Bacteria	Ensembl
**1185651**	Enterovibrio norvegicus FF-454	Enor	47.60	48.17	4276	Proteobacteria	Bacteria	Ensembl
**1189619**	Psychroflexus gondwanensis ACAM 44	Pgon	35.80	36.41	2895	Bacteroidetes	Bacteria	Ensembl
**1189621**	Nitritalea halalkaliphila LW7	Nhal	48.60	49.35	3035	Bacteroidetes	Bacteria	Ensembl
**1198115**	Thaumarchaeota archaeon SCGC AB-539-E09	Tarc	43.30	44.52	605	Thaumarchaeota	Archaea	Ensembl
**1198449**	Aeropyrum camini SY1 = JCM 12091	Acam	56.70	57.31	1645	Crenarchaeota	Archaea	Ensembl
**1201294**	Methanoculleus bourgensis MS2	Mbou	60.60	61.54	2579	Euryarchaeota	Archaea	Ensembl
**1208320**	Thalassolituus oleivorans R6-15	Tole	46.60	46.98	3368	Proteobacteria	Bacteria	Ensembl
**1208660**	Bordetella parapertussis Bpp5	Bpar	67.78	68.14	4174	Proteobacteria	Bacteria	Ensembl
**1208920**	Candidatus Kinetoplastibacterium oncopeltii TCC290E	Konc	31.20	31.87	694	Proteobacteria	Bacteria	Ensembl
**1209989**	Tepidanaerobacter acetatoxydans Re1	Tace	37.50	38.31	2524	Firmicutes	Bacteria	Ensembl
**1223560**	Pythium vexans DAOM BR484	Pvex	58.70	61.38	11851		Eukaryota	Ensembl
**1227812**	Piscirickettsia salmonis LF-89 = ATCC VR-1361	Psal	39.62	40.82	3127	Proteobacteria	Bacteria	Ensembl
**1229908**	Candidatus Nitrosopumilus koreensis AR1	Nkor	34.20	34.69	1883	Thaumarchaeota	Archaea	Ensembl
**1236689**	Candidatus Methanomethylophilus alvus	Malv	55.60	56.62	1641	Euryarchaeota	Archaea	Ensembl

TaxId	Species	Nick-name	Ann. GC%	CDS GC%	Num CDSs	Phylum	Domain	Source
	Mx1201							
**1236703**	Candidatus Photodesmus katoptron Akat1	Pkat	31.06	31.78	854	Proteobacteria	Bacteria	Ensembl
**1237085**	Candidatus Nitrososphaera gargensis Ga9.2	Ngar	48.30	49.80	3559	Thaumarchaeota	Archaea	Ensembl
**1245935**	Tolypothrix campylonemoides VB511288	Tcam	45.10	46.39	6844	Cyanobacteria	Bacteria	Ensembl
**1257118**	Acanthamoeba castellanii str. Neff	Acas	57.80	62.95	14229		Eukaryota	Ensembl
**1266370**	Nitrospina gracilis 3-211	Ngra	56.10	56.92	2947	Nitrospinae	Bacteria	Ensembl
**1266844**	Acetobacter pasteurianus 386B	Apas	53.20	53.58	2865	Proteobacteria	Bacteria	Ensembl
**1273541**	Pyrodictium delaneyi	Pdel	53.90	54.37	2035	Crenarchaeota	Archaea	Ensembl
**1287680**	Neofusicoccum parvum UCRNP2	Npar	56.70	60.86	10366	Ascomycota	Eukaryota	Ensembl
**1292022**	Curtobacterium flaccumfaciens UCD-AKU	Cfla	70.80	71.02	3365	Actinobacteria	Bacteria	Ensembl
**1295009**	Candidatus Methanomassiliicoccus intestinalis Issoire-Mx1 str. Mx1-Issoire	Mint	41.30	42.14	1826	Euryarchaeota	Archaea	Ensembl
**1298851**	Thermosulfidibacter takaii ABI70S6	Ttak	43.00	42.99	1757	Aquificae	Bacteria	Ensembl
**1303518**	Chthonomonas calidirosea T49	Ccal	54.60	55.16	2805	Armatimonadetes	Bacteria	Ensembl
**1304892**	Xanthomonas axonopodis Xac29-1	Xaxo	64.72	65.21	3289	Proteobacteria	Bacteria	Ensembl
**1307761**	Salinispira pacifica	Spac	51.90	52.30	3397	Spirochaetes	Bacteria	
**1313172**	Ilumatobacter coccineus YM16-304	Icoc	67.30	67.47	4289	Actinobacteria	Bacteria	Ensembl
**1319815**	Cetobacterium somerae ATCC BAA-474	Csom	28.60	28.95	2889	Fusobacteria	Bacteria	Ensembl
**1321371**	Holospora undulata HU1	Hund	36.10	37.52	1218	Proteobacteria	Bacteria	Ensembl
**1330330**	Kosmotoga pacifica	Kpac	42.50	42.81	1897	Thermotogae	Bacteria	Ensembl
**1341181**	Flavobacterium limnosediminis JC2902	Flim	38.50	39.45	2901	Bacteroidetes	Bacteria	Ensembl
**1343739**	Palaeococcus pacificus DY20341	Ppac	43.00	43.55	1988	Euryarchaeota	Archaea	Ensembl
**1347342**	Formosa agariphila KMM 3901	Faga	33.60	34.27	3567	Bacteroidetes	Bacteria	Ensembl
**1379270**	Gemmatimonas phototrophica	Gpho	64.40	64.58	3388	Gemmatimonadetes	Bacteria	Ensembl
**1379858**	Mucispirillum schaedleri ASF457	Msch	31.20	31.94	2124	Deferribacteres	Bacteria	Ensembl
**1397361**	Sporothrix schenckii 1099-18	Ssch	55.00	61.56	10288	Ascomycota	Eukaryota	Ensembl
**1408204**	Candidatus Endomicrobium trichonymphae	Etri	35.80	36.79	2768	Elusimicrobia	Bacteria	Ensembl
**1427984**	Candidatus Hepatoplasma crinochetorum Av	Hcri	22.50	22.73	567	Tenericutes	Bacteria	Ensembl

TaxId	Species	Nick-name	Ann. GC%	CDS GC%	Num CDSs	Phylum	Domain	Source
**1429438**	Candidatus Entotheonella sp. TSY1	EntTSY1	55.30	56.83	8139	Candidatus Tectomicrobia	Bacteria	Ensembl
**1429439**	Candidatus Entotheonella sp. TSY2	EntTSY2	55.30	56.69	8264	Candidatus Tectomicrobia	Bacteria	Ensembl
**1432061**	Dehalococcoides mccartyi CG5	DmccCG5	48.90	48.04	1428	Chloroflexi	Bacteria	Ensembl
**1432562**	Salinicoccus sediminis	Ssed	48.70	49.84	2485	Firmicutes	Bacteria	Ensembl
**1432656**	Thermococcus guaymasensis DSM 11113	Tgua	52.90	53.61	2085	Euryarchaeota	Archaea	Ensembl
**1435057**	Agrobacterium tumefaciens LBA4213 (Ach5)	Atum	59.87	59.37	5420	Proteobacteria	Bacteria	Ensembl
**1439331**	Lelliottia amnigena CHS 78	Lamn	54.30	56.12	4511	Proteobacteria	Bacteria	Ensembl
**1441628**	Leptospirillum ferriphilum YSK	Lfer	54.60	54.92	2260	Nitrospirae	Bacteria	Ensembl
**1454006**	Siansivirga zeaxanthinifaciens CC-SAMT-1	Szea	33.50	34.33	2761	Bacteroidetes	Bacteria	Ensembl
**1469144**	Streptomyces thermoautotrophicus	Strphicus	69.20	70.88	3626	Actinobacteria	Bacteria	Ensembl
**1502293**	Marine Group I thaumarchaeote SCGC AAA799-N04	tSCG	34.20	34.72	1670	Thaumarchaeota	Archaea	Ensembl
**1514904**	Ahrensia marina str. LZD062	Amar	50.10	50.77	3143	Proteobacteria	Bacteria	Ensembl
**1519565**	Fistulifera solaris	Fsol	45.60	48.45	20365	Bacillariophyta	Eukaryota	NCBI
**1529318**	Cryobacterium sp. MLB-32	CMLB	67.53	65.31	3045	Actinobacteria	Bacteria	Ensembl
**1574623**	Lyngbya confervoides BDU141951	Lcon	55.00	56.67	5685	Cyanobacteria	Bacteria	Ensembl
**1577684**	Candidatus Nanopusillus acidilobi	Naci	24.20	24.14	580	Nanoarchaeota	Archaea	Ensembl
**1618331**	Berkelbacteria bacterium GW2011_GWA1_36_9	Ber1_36_9	35.90	36.10	907	Candidatus Berkelbacteria	Bacteria	Ensembl
**1618369**	Candidatus Beckwithbacteria bacterium GW2011_GWA2_43_10	Bec_43_10	43.00	43.30	663	Candidatus Beckwithbacteria	Bacteria	Ensembl
**1618380**	Candidatus Collierbacteria bacterium GW2011_GWA2_44_99	CGW2	43.80	44.05	733	Candidatus Collierbacteria	Bacteria	
**1618405**	Candidatus Curtissbacteria bacterium GW2011_GWA1_40_16	Cur_40_16	40.80	41.15	1014	Candidatus Curtissbacteria	Bacteria	Ensembl
**1618443**	Candidatus Gottesmanbacteria bacterium GW2011_GWA2_43_14	GGW2	43.20	43.69	1684	Candidatus Gottesmanbacteria	Bacteria	Ensembl
**1618595**	Candidatus Woesebacteria bacterium GW2011_GWD2_40_19	WGW2	40.10	40.32	777	Candidatus Woesebacteria	Bacteria	Ensembl
**1618609**	Candidatus Azambacteria bacterium GW2011_GWA1_42_19	Aza_42_19	41.50	41.91	585	Candidatus Azambacteria	Bacteria	Ensembl
**1618623**	Candidatus Azambacteria bacterium	Aza_46_48	46.10	46.72	582	Candidatus Azambacteria	Bacteria	Ensembl

TaxId	Species	Nick-name	Ann. GC%	CDS GC%	Num CDSs	Phylum	Domain	Source
	GW2011_GWD2_46_48							
**1618643**	Candidatus Falkowbacteria bacterium GW2011_GWF2_43_32	FGW2	43.30	44.37	789	Candidatus Falkowbacteria	Bacteria	Ensembl
**1618662**	Candidatus Jorgensenbacteria bacterium GW2011_GWA2_45_13	JGW2	45.20	46.02	631	Candidatus Jorgensenbacteria	Bacteria	Ensembl
**1618671**	Candidatus Kaiserbacteria bacterium GW2011_GWA2_52_12	Kai_52_12	52.00	52.62	966	Candidatus Kaiserbacteria	Bacteria	Ensembl
**1618673**	Candidatus Kaiserbacteria bacterium GW2011_GWB1_50_17	Kai_50_17	50.00	50.55	458	Candidatus Kaiserbacteria	Bacteria	Ensembl
**1618729**	Candidatus Nomurabacteria bacterium GW2011_GWA1_37_20	Nom_37_20	36.90	37.10	590	Candidatus Nomurabacteria	Bacteria	Ensembl
**1618742**	Candidatus Nomurabacteria bacterium GW2011_GWB1_37_5	Nom1_37_5	36.70	37.24	783	Candidatus Nomurabacteria	Bacteria	Ensembl
**1618775**	Candidatus Nomurabacteria bacterium GW2011_GWF2_36_19	Nom_36_19	36.20	36.81	795	Candidatus Nomurabacteria	Bacteria	Ensembl
**1618777**	Candidatus Nomurabacteria bacterium GW2011_GWF2_40_31	Nom_40_31	39.60	39.96	578	Candidatus Nomurabacteria	Bacteria	Ensembl
**1618821**	Parcubacteria group bacterium GW2011_GWA2_42_18	Par_42_18	41.60	42.09	584		Bacteria	Ensembl
**1618840**	Parcubacteria group bacterium GW2011_GWA2_47_10b	Par47_10b	47.10	47.34	845		Bacteria	Ensembl
**1618841**	Parcubacteria group bacterium GW2011_GWA2_47_12	Par_47_12	46.80	47.44	753		Bacteria	Ensembl
**1618924**	Parcubacteria group bacterium GW2011_GWC2_40_31	Par_40_31	40.40	40.91	813		Bacteria	Ensembl
**1619005**	Candidatus Wolfebacteria bacterium GW2011_GWA2_47_9b	Wol_47_9b	46.70	47.48	1053	Candidatus Wolfebacteria	Bacteria	Ensembl
**1619029**	Candidatus Yanofskybacteria bacterium GW2011_GWC2_41_9	YGW2	41.30	41.76	640	Candidatus Yanofskybacteria	Bacteria	Ensembl
**1619051**	Candidatus Magasanikbacteria bacterium GW2011_GWD2_43_18	MGW2	43.00	43.27	1142	Candidatus Magasanikbacteria	Bacteria	Ensembl
**1619068**	Candidatus Peregrinibacteria bacterium GW2011_GWF2_43_17	PGW2	43.10	43.40	1124	Candidatus Peregrinibacteria	Bacteria	Ensembl
**1619079**	candidate division TM6 bacterium GW2011_GWF2_32_72	dTM6	32.70	33.16	880		Bacteria	Ensembl
**1630693**	Gemmata sp. SH-PL17	GSH-	64.20	64.99	7691	Planctomycetes	Bacteria	Ensembl
**173740**	Nanohaloarchaea archaeon	Narc	46.40	46.95	1183	Candidatus	Archaea	Ensembl

TaxId	Species	Nick-name	Ann. GC%	CDS GC%	Num CDSs	Phylum	Domain	Source
**3**	SG9					Nanohaloarchaeota		

Explanation of table columns: **TaxId** – NCBI taxonomic identifier. **Species** – Species name and strain. **Nick-name** – Short species identifiers (used in other figures). **Ann. GC%** – Genomic GC-content as annotated in NCBI. **CDS GC%** – Mean GC-content in CDS sequences (for comparison, not used in analysis). **Num CDSs** – Number of CDSs included in species ΔLFE profile. **Phylum** – Parent phylum of species. **Domain** – Parent domain of species. **Source** – Source of CDS sequences and genome annotations for species (see main text).

Table S2. Phyla representation

TaxId	Domain	Phylum	NumFamilies	NumGenuses	NumOrders	NumSpecies
**51967**	Archaea	Candidatus Korarchaeota	0	1	0	1
**1462430**	Archaea	Candidatus Nanohaloarchaeota	0	0	0	2
**28889**	Archaea	Crenarchaeota	5	9	4	11
**28890**	Archaea	Euryarchaeota	18	31	12	40
**192989**	Archaea	Nanoarchaeota	2	2	1	2
**651137**	Archaea	Thaumarchaeota	3	4	3	8
	Archaea	[Total]	0	0	0	64
**57723**	Bacteria	Acidobacteria	2	2	2	2
**201174**	Bacteria	Actinobacteria	20	31	17	35
**200783**	Bacteria	Aquificae	3	9	2	10
**67819**	Bacteria	Armatimonadetes	2	2	2	2
**976**	Bacteria	Bacteroidetes	9	31	5	35
**67814**	Bacteria	Caldiserica	1	1	1	1
**1930617**	Bacteria	Calditrichaeota	1	1	1	1
**1752741**	Bacteria	Candidatus Azambacteria	0	0	0	2
**1752726**	Bacteria	Candidatus Beckwithbacteria	0	0	0	1
**1618330**	Bacteria	Candidatus Berkelbacteria	0	0	0	1
**1752725**	Bacteria	Candidatus Collierbacteria	0	0	0	1
**1752717**	Bacteria	Candidatus Curtissbacteria	0	0	0	1
**1752728**	Bacteria	Candidatus Falkowbacteria	0	0	0	1
**1752720**	Bacteria	Candidatus Gottesmanbacteria	0	0	0	1
**1752739**	Bacteria	Candidatus Jorgensenbacteria	0	0	0	1
**1752734**	Bacteria	Candidatus Kaiserbacteria	0	0	0	2
**1752731**	Bacteria	Candidatus Magasanikbacteria	0	0	0	1
**1752729**	Bacteria	Candidatus Nomurabacteria	0	0	0	4
**1619053**	Bacteria	Candidatus Peregrinibacteria	0	0	0	1
**1802339**	Bacteria	Candidatus Tectomicrobia	0	1	0	2
**1752722**	Bacteria	Candidatus Woesebacteria	0	0	0	1
**1752735**	Bacteria	Candidatus Wolfebacteria	0	0	0	1
**1752733**	Bacteria	Candidatus Yanofskybacteria	0	0	0	1
**204428**	Bacteria	Chlamydiae	3	3	2	5
**1090**	Bacteria	Chlorobi	1	2	1	3
**200795**	Bacteria	Chloroflexi	10	12	8	14
**200938**	Bacteria	Chrysiogenetes	1	1	1	1
**1117**	Bacteria	Cyanobacteria	10	13	5	15
**200930**	Bacteria	Deferribacteres	1	4	1	4
**1297**	Bacteria	Deinococcus-Thermus	3	6	2	11
**68297**	Bacteria	Dictyoglomi	1	1	1	2
**74152**	Bacteria	Elusimicrobia	2	2	2	2

TaxId	Domain	Phylum	NumFamilies	NumGenuses	NumOrders	NumSpecies
**65842**	Bacteria	Fibrobacteres	1	1	1	1
**1239**	Bacteria	Firmicutes	23	34	10	44
**32066**	Bacteria	Fusobacteria	2	6	1	8
**142182**	Bacteria	Gemmatimonadetes	1	2	1	3
**1134404**	Bacteria	Ignavibacteriae	1	1	1	1
**256845**	Bacteria	Lentisphaerae	1	1	1	1
**1293497**	Bacteria	Nitrospinae	1	1	1	1
**40117**	Bacteria	Nitrospirae	1	4	1	4
**203682**	Bacteria	Planctomycetes	4	6	2	6
**1224**	Bacteria	Proteobacteria	55	84	35	92
**203691**	Bacteria	Spirochaetes	3	5	2	6
**508458**	Bacteria	Synergistetes	1	4	1	4
**544448**	Bacteria	Tenericutes	2	5	2	11
**200940**	Bacteria	Thermodesulfobacteria	1	2	1	3
**200918**	Bacteria	Thermotogae	3	8	3	10
**74201**	Bacteria	Verrucomicrobia	4	4	4	4
	Bacteria	[Unknown]	0	0	0	7
	Bacteria	[Total]	0	0	0	371
**5794**	Eukaryota	Apicomplexa	5	5	2	5
**6656**	Eukaryota	Arthropoda	1	1	1	1
**4890**	Eukaryota	Ascomycota	10	13	8	16
**2836**	Eukaryota	Bacillariophyta	4	4	3	4
**5204**	Eukaryota	Basidiomycota	9	9	5	9
**451459**	Eukaryota	Blastocladiomycota	1	1	1	1
**3041**	Eukaryota	Chlorophyta	6	6	2	6
**4761**	Eukaryota	Chytridiomycota	1	1	1	1
**6073**	Eukaryota	Cnidaria	1	1	1	1
**10197**	Eukaryota	Ctenophora	1	1	1	1
**10226**	Eukaryota	Placozoa	0	1	0	1
**6040**	Eukaryota	Porifera	1	1	1	1
**10190**	Eukaryota	Rotifera	1	1	1	1
**35493**	Eukaryota	Streptophyta	2	2	2	2
	Eukaryota	[Unknown]	0	0	0	28
	Eukaryota	[Total]	0	0	0	78
	[All]	[Total]	245	384	169	513

Explanation of table columns: **TaxId** – NCBI taxonomic identifier for phylum. **Domain** – Parent domain of phylum. **Phylum** – Name of phylum. **Num Families** – Number of families represented in dataset. **Num Orders** – Number of orders in dataset. **Num Genuses** – Number of genuses in dataset. **Num Species** – Number of species in dataset.

Table S3. Genomic and environmental properties

Tax Id	Species	Nick-name	Genome Size Mb	Genomic ENc'	Genomic GC%	Growth TempC	Growth Time Hours	In Phylo Tree	Is Endosymbiont	Endosymbiont Ref	Translation Tbl
592010	Abiotrophia defectiva ATCC 49176	Adef	2.0	53.33	47.00			+			11
1257118	Acanthamoeba castellanii str. Neff	Acas	42.0	49.81	57.80			+			1
1266844	Acetobacter pasteurianus 386B	Apas	3.0	50.76	53.20			+			11
574087	Acetohalobium arabaticum DSM 5501	Aara	2.5	53.49	36.60			+			11
1009370	Acetonema longum DSM 6540	Alon	4.3	50.94	50.40			+			11
441768	Acholeplasma laidlawii PG-8A	Alai	1.5	51.76	31.90	37.0		+			11
525909	Acidimicrobium ferrooxidans DSM 10331	Afer	2.2	50.33	68.30			+			11
507754	Acidiplasma aeolicum str. VT	Aaeo		49.45	34.20						11
743299	Acidithiobacillus ferrivorans SS3	AferSS3	3.2	53.39	56.60			+			11
243159	Acidithiobacillus ferrooxidans ATCC 23270	Afer23270	3.0	52.52	58.80	32.5					11
240015	Acidobacterium capsulatum ATCC 51196	Acap	4.1	49.92	60.50	30.0					11
351607	Acidothermus cellulolyticus 11B	Acel	2.4	53.02	66.90	58.0		+			11
400667	Acinetobacter baumannii ATCC 17978	Abau	4.3	50.71	39.00	37.0			+	(1)	11
104782	Adineta vaga	Avag	217.9	47.36	31.20						1
746697	Aequorivita sublithincola DSM 14238	Asub	3.5	55.48	36.20			+			11
1198449	Aeropyrum camini SY1 = JCM 12091	Acam	1.6	47.68	56.70						11
272557	Aeropyrum pernix K1	Aper	1.7	48.11	56.30	92.5	4.00				11
176299	Agrobacterium fabrum str. C58	Afab	5.7	49.35	59.06	26.5					11
1435057	Agrobacterium tumefaciens LBA4213 (Ach5)	Atum	7.3	49.96	59.87	26.5	3.00				11
1514904	Ahrensia marina str. LZD062	Amar		50.90	50.10						11
349741	Akkermansia muciniphila ATCC BAA-835	Amuc	2.7	48.02	55.80	37.0		+			11
65357	Albugo candida	Acan	32.9	57.43	43.20						1
393595	Alcanivorax borkumensis SK2	Abor	3.1	51.30	54.70		10.00	+			11
543302	Alicyclobacillus acidocaldarius LAA1	Aaci	3.2	51.58	61.86	62.5		+			11
187272	Alkalilimnicola ehrlichii MLHE-1	Aehr	3.3	47.12	67.50			+			11
578462	Allomyces macrogynus ATCC 38327	Amac	57.1	50.11	60.50			+			1

Tax Id	Species	Nick-name	Genome Size Mb	Genomic ENc'	Genomic GC%	Growth TempC	Growth Time Hours	In Phylo Tree	Is Endosymbiont	Endosymbiont Ref	Translation Tbl
400682	Amphimedon queenslandica	Aque	166.7	56.04	37.50			+			1
46234	Anabaena sp. 90	A90	5.3	54.00	38.09						11
891968	Anaerobaculum mobile DSM 13181	Amob	2.2	55.05	48.00			+			11
525919	Anaerococcus prevotii DSM 20548	Apre	2.0	53.01	35.67			+			11
926569	Anaerolinea thermophila UNI-1	Athe	3.5	51.81	53.80			+			11
491915	Anoxybacillus flavithermus WK1	Afla	2.8	50.61	41.80	62.5		+			11
224324	Aquifex aeolicus VF5	AaeoVF5	1.6	48.34	43.32	96.0	1.80	+			11
224325	Archaeoglobus fulgidus DSM 4304	Aful	2.2	49.67	48.60	83.0	4.00	+			11
696747	Arthrospira platensis NIES-39	Apla	6.8	55.65	44.30			+			11
5061	Aspergillus niger	Anig	34.6	58.40	50.30						1
322098	Aster yellows witches'-broom phytoplasma AYWB	Ayel	0.7	51.65	26.83			+	+	(2)	11
573065	Asticcacaulis excentricus CB 48	Aexc	4.3	49.49	59.53			+			11
44056	Aureococcus anophagefferens	Aano	56.7	46.19	67.40			+			1
484906	Babesia bovis T2Bo	Bbov		57.75	41.61			+			1
1121088	Bacillus coagulans DSM 1 = ATCC 7050	Bcoa	3.4	50.66	46.90						11
272558	Bacillus halodurans C-125	Bhal	4.2	56.37	43.70		0.60				11
439292	Bacillus selenitireducens MLS10	Bsel	3.6	53.93	48.70			+			11
224308	Bacillus subtilis subsp. subtilis str. 168	Bsub	4.2	54.95	43.50	30.0	0.43				11
295405	Bacteroides fragilis YCH46	Bfra	5.3	54.64	43.24	37.0	0.63				11
997884	Bacteroides nordii	Bnor	5.7	54.40	40.80						11
226186	Bacteroides thetaiotaomicron VPI-5482	Bthe	6.3	53.90	42.82		1.47				11
283166	Bartonella henselae str. Houston-1	Bhen	1.9	51.31	38.20	37.0	3.00		+	(3)	11
264462	Bdellovibrio bacteriovorus HD100	Bbac	3.0	49.57	43.30		1.40	+	+	(4)	11
1618331	Berkelbacteria bacterium GW2011_GWA1_36_9	Ber1_36_9	0.9	56.75	35.90			+			11
703613	Bifidobacterium animalis subsp. animalis ATCC 25527	Bani	1.9	47.53	60.50			+			11
753081	Bigelowiella natans	Bnat	91.4	58.83	44.90			+			1
1046627	Bizionia argentinensis JUB59	Barg	3.3	54.42	33.80			+			11
331104	Blattabacterium sp. (Blattella germanica) str. Bge	BBge	0.6	50.77	23.84				+	(5)	11
120866	Bordetella parapertussis Bpp5	Bpar	4.9	43.93	67.78						11

Tax Id	Species	Nick-name	Genome Size Mb	Genomic ENc'	Genomic GC%	Growth TempC	Growth Time Hours	In Phylo Tree	Is Endosymbiont	Endosymbiont Ref	Translation Tbl
**0**											
**930990**	Botryobasidium botryosum FD-172 SS1	Bbot	46.7	58.59	52.30			+			1
**526224**	Brachyspira murdochii DSM 12563	Bmur	3.2	49.86	27.80			+			11
**476282**	Bradyrhizobium japonicum SEMIA 5079	Bjap	9.2	47.94	63.70		20.00	+			11
**358681**	Brevibacillus brevis NBRC 100599	Bbre	6.3	56.24	47.30			+			11
**633149**	Brevundimonas subvibrioides ATCC 15264	Bsub15264	3.4	45.68	68.40			+			11
**224914**	Brucella melitensis bv. 1 str. 16M	Bmel	3.3	48.02	57.24	37.0	2.00		+	(6)	11
**107806**	Buchnera aphidicola str. APS (Acyrthosiphon pisum)	Baph	0.6	52.03	25.30		36.00		+	(7)	11
**926550**	Caldilinea aerophila DSM 14535 = NBRC 104270	Caer	5.1	51.50	58.80	55.0		+			11
**511051**	Caldisericum exile AZM16c01	Cexi	1.6	52.74	35.40			+			11
**1056495**	Caldisphaera lagunensis DSM 15908	Clag	1.5	52.55	30.00			+			11
**768670**	Calditerrivibrio nitroreducens DSM 19672	Cnit	2.2	54.86	35.68	55.0		+			11
**880073**	Caldithrix abyssi DSM 13497	Caby	5.0	49.13	45.10			+			11
**192222**	Campylobacter jejuni subsp. jejuni NCTC 11168 = ATCC 700819	Cjej	1.6	51.61	30.50		1.50	+			11
**237561**	Candida albicans SC5314	Calb	14.3	53.57	33.48						1
**1618609**	Candidatus Azambacteria bacterium GW2011_GWA1_42_19	Aza_42_19	0.5	52.24	41.50			+			11
**1618623**	Candidatus Azambacteria bacterium GW2011_GWD2_46_48	Aza_46_48	0.6	51.16	46.10			+			11
**1618369**	Candidatus Beckwithbacteria bacterium GW2011_GWA2_43_10	Bec_43_10	0.6	51.74	43.00			+			11
**203907**	Candidatus Blochmannia floridanus	Bflo	0.7	51.66	27.40		36.00	+	+	(8)	11
**1618380**	Candidatus Collierbacteria bacterium GW2011_GWA2_44_99	CGW2	0.6	56.02	43.80			+			11
**1618405**	Candidatus Curtissbacteria bacterium GW2011_GWA1_40_16	Cur_40_16	1.0	57.57	40.80			+			11
**477974**	Candidatus Desulforudis audaxviator MP104C	Daud	2.3	50.46	60.80			+			11
**1408204**	Candidatus Endomicrobium trichonymphae	Etri	3.4	54.02	35.80			+			11
**1429438**	Candidatus Entotheonella sp. TSY1	EntTSY1	9.7	52.78	55.30			+			11
**1429439**	Candidatus Entotheonella sp. TSY2	EntTSY2	9.7	53.13	55.30			+			11
**161**	Candidatus Falkowbacteria	FGW2	0.9	47.89	43.30			+			11

Tax Id	Species	Nick-name	Genome Size Mb	Genomic ENc'	Genomic GC%	Growth TempC	Growth Time Hours	In Phylo Tree	Is Endosymbiont	Endosymbiont Ref	Translation Tbl
8643	bacterium GW2011_GWF2_43_32										
1618443	Candidatus Gottesmanbacteria bacterium GW2011_GWA2_43_14	GGW2	1.7	53.84	43.20			+			11
1072681	Candidatus Haloredivivus sp. G17	HG17	1.2	54.59	42.00			+			11
1427984	Candidatus Hepatoplasma crinochetorum Av	Hcri	0.7	52.06	22.50			+			4
1618662	Candidatus Jorgensenbacteria bacterium GW2011_GWA2_45_13	JGW2	0.6	54.68	45.20			+			11
1618671	Candidatus Kaiserbacteria bacterium GW2011_GWA2_52_12	Kai_52_12	0.9	53.52	52.00			+			11
1618673	Candidatus Kaiserbacteria bacterium GW2011_GWB1_50_17	Kai_50_17	0.4	55.64	50.00			+			11
1208920	Candidatus Kinetoplastibacterium oncopeltii TCC290E	Konc	0.8	53.13	31.20			+	+	(9)	11
374847	Candidatus Korarchaeum cryptofilum OPF8	Kcry	1.6	47.16	49.00			+			11
1619051	Candidatus Magasanikbacteria bacterium GW2011_GWD2_43_18	MGW2	1.1	53.69	43.00			+			11
29290	Candidatus Magnetobacterium bavaricum	Mbav	6.3	56.19	47.30						11
1295009	Candidatus Methanomassiliicoccus intestinalis Issoire-Mx1 str. Mx1-Issoire	Mint		54.62	41.30			+			11
1236689	Candidatus Methanomethylophilus alvus Mx1201	Malv	1.7	45.32	55.60			+			11
903503	Candidatus Moranella endobia PCIT	Mend	0.5	53.19	43.50			+			11
1577684	Candidatus Nanopusillus acidilobi	Naci	0.6	50.92	24.20	80.0					11
859192	Candidatus Nitrosoarchaeum limnia BG20	Nlim	1.9	52.76	32.50						11
1229908	Candidatus Nitrosopumilus koreensis AR1	Nkor	1.6	52.20	34.20	25.0		+			11
1237085	Candidatus Nitrososphaera gargensis Ga9.2	Ngar	2.8	53.82	48.30						11
1618729	Candidatus Nomurabacteria bacterium GW2011_GWA1_37_20	Nom_37_20	0.6	55.70	36.90			+			11
1618742	Candidatus Nomurabacteria bacterium GW2011_GWB1_37_5	Nom1_37_5	0.7	57.03	36.70			+			11
1618775	Candidatus Nomurabacteria bacterium GW2011_GWF2_36_19	Nom_36_19	0.8	55.88	36.20						11
161877	Candidatus Nomurabacteria bacterium	Nom_40_31	0.6	56.95	39.60			+			11

Tax Id	Species	Nick-name	Genome Size Mb	Genomic ENc'	Genomic GC%	Growth TempC	Growth Time Hours	In Phylo Tree	Is Endosymbiont	Endosymbiont Ref	Translation Tbl
7	GW2011_GWF2_40_31										
1002672	Candidatus Pelagibacter sp. IMCC9063	PIMC	1.3	54.70	31.70			+			11
1619068	Candidatus Peregrinibacteria bacterium GW2011_GWF2_43_17	PGW2	1.2	54.69	43.10			+			11
1236703	Candidatus Photodesmus katoptron Akat1	Pkat	1.0	50.44	31.06			+	+	(10)	11
234267	Candidatus Solibacter usitatus Ellin6076	Susi	10.0	50.63	61.90	30.0					11
1618595	Candidatus Woesebacteria bacterium GW2011_GWD2_40_19	WGW2	0.7	55.50	40.10			+			11
1619005	Candidatus Wolfebacteria bacterium GW2011_GWA2_47_9b	Wol_47_9b	1.0	56.02	46.70			+			11
1619029	Candidatus Yanofskybacteria bacterium GW2011_GWC2_41_9	YGW2	0.6	53.07	41.30			+			11
521097	Capnocytophaga ochracea DSM 7271	Coch	2.6	51.52	39.60	36.0		+			11
595528	Capsaspora owczarzaki ATCC 30864	Cowc	28.0	53.71	53.70			+			1
479433	Catenulispora acidiphila DSM 44928	Caci	10.5	47.12	69.80			+			11
190650	Caulobacter crescentus CB15	Ccre	4.0	45.55	67.20	35.0	1.50				11
979	Cellulophaga lytica	Clyt	3.8	51.33	32.10			+			11
414004	Cenarchaeum symbiosum A	Csym	2.0	51.98	57.40	10.0		+			11
1319815	Cetobacterium somerae ATCC BAA-474	Csom	3.1	50.26	28.60			+			11
218497	Chlamydia abortus S26-3	Cabo		55.75	39.90		24.00	+	+	(11)	11
3055	Chlamydomonas reinhardtii	Crei	120.4	51.49	61.95						1
115713	Chlamydophila pneumoniae CWL029	CpneWL029	1.2	55.80	40.60	37.0			+	(12)	11
138677	Chlamydophila pneumoniae J138	CpneJ138		55.82	40.60		24.00		+	(12)	11
517417	Chlorobaculum parvum NCIB 8327	Cpar	2.3	49.88	55.80			+			11
194439	Chlorobium tepidum TLS	Ctep	2.2	49.98	56.50	48.0	2.00				11
326427	Chloroflexus aggregans DSM 9485	Cagg	4.7	53.71	56.40						11
324602	Chloroflexus aurantiacus J-10-fl	Caur	5.3	53.19	56.70	56.0	6.00	+			11
517418	Chloroherpeton thalassium ATCC 35110	Ctha	3.3	50.46	45.00			+			11
2769	Chondrus crispus (carragheen)	Ccri		59.00	52.86						1
243365	Chromobacterium violaceum ATCC 12472	Cvio	4.8	43.58	64.80	25.0	0.80	+			11
345663	Chryseobacterium greenlandense	Cgre	4.0	54.24	34.10						11

Tax Id	Species	Nick-name	Genome Size Mb	Genomic ENc'	Genomic GC%	Growth TempC	Growth Time Hours	In Phylo Tree	Is Endosymbiont	Endosymbiont Ref	Translation Tbl
**1303518**	Chthonomonas calidirosea T49	Ccal	3.4	56.15	54.60			+			11
**443906**	Clavibacter michiganensis subsp. michiganensis NCPPB 382	Cmic	3.4	45.00	72.42	26.5					11
**866499**	Cloacibacillus evryensis DSM 19522	Cevr	3.5	49.66	56.00	37.0		+			11
**642492**	Clostridium lentocellum DSM 5427	Clen	4.7	54.09	34.30	40.0		+			11
**212717**	Clostridium tetani E88	Ctet	2.9	52.83	28.59	37.0	0.50				11
**1055104**	Cobetia amphilecti str. KMM 296	Camp		45.14	62.50			+			11
**574566**	Coccomyxa subellipsoidea C-169	Csub	48.8	52.76	52.90						1
**469383**	Conexibacter woesei DSM 14684	Cwoe	5.7	44.37	72.40			+			11
**583355**	Coraliomargarita akajimensis DSM 45221	Caka	3.8	53.84	53.60			+			11
**196164**	Corynebacterium efficiens YS-314	Ceff	3.2	47.89	62.93	37.5					11
**196627**	Corynebacterium glutamicum ATCC 13032	Cglu	3.3	52.51	53.80	35.0	1.20				11
**227377**	Coxiella burnetii RSA 493	Cbur	2.2	54.47	42.34	37.0	8.00		+	(13)	11
**216432**	Croceibacter atlanticus HTCC2559	Catl	3.0	53.28	33.90			+			11
**1529318**	Cryobacterium sp. MLB-32	CMLB	4.0	51.31	67.53			+			11
**214684**	Cryptococcus neoformans var. neoformans JEC21	Cneo	19.1	56.73	48.54						1
**2898**	Cryptomonas paramecium	Crymecium	0.6	58.46	27.81						1
**353152**	Cryptosporidium parvum Iowa II	CparII	9.1	54.92	30.25			+	+	(14)	1
**1292022**	Curtobacterium flaccumfaciens UCD-AKU	Cfla	3.7	45.69	70.80			+			11
**280699**	Cyanidioschyzon merolae	Cmer	16.5	58.02	55.02			+			1
**6669**	Daphnia pulex	Dpul	197.2	57.94	42.40			+			1
**639282**	Deferribacter desulfuricans SSM1	Ddes	2.5	54.66	30.30	62.5		+			11
**255470**	Dehalococcoides mccartyi CBDB1	DmccCBDB1	1.5	51.38	48.90	35.0		+			11
**1432061**	Dehalococcoides mccartyi CG5	DmccCG5	1.5	51.27	48.90	35.0					11
**552811**	Dehalogenimonas lykanthroporepellens BL-DC-9	Dlyk	1.7	50.82	55.00			+			11
**319795**	Deinococcus geothermalis DSM 11300 str. DSM11300	Dgeo		49.99	66.57			+			11
**937777**	Deinococcus peraridilitoris DSM 19664	Dper	4.5	50.08	63.71						11
**1182568**	Deinococcus puniceus	Dpun	3.0	48.03	62.60						11

Tax Id	Species	Nick-name	Genome Size Mb	Genomic ENc'	Genomic GC%	Growth TempC	Growth Time Hours	In Phylo Tree	Is Endosymbiont	Endosymbiont Ref	Translation Tbl
**243230**	Deinococcus radiodurans R1	Drad	3.3	48.45	66.61	33.5	1.50				11
**522772**	Denitrovibrio acetiphilus DSM 12809	Dace	3.2	52.97	42.50	36.0		+			11
**651182**	Desulfobacula toluolica Tol2	Dtol	5.2	53.14	41.40			+			11
**555779**	Desulfonatronospira thiodismutans ASO3-1	Dthi	4.1	50.21	51.30			+			11
**768706**	Desulfosporosinus orientis DSM 765	Dori	5.9	56.91	42.90			+			11
**882**	Desulfovibrio vulgaris str. Hildenborough	Dvul	4.0	51.11	67.10		14.00				11
**653733**	Desulfurispirillum indicum S5	Dind	2.9	48.29	56.10			+			11
**868864**	Desulfurobacterium thermolithotrophum DSM 11699	Dthe	1.5	50.12	34.90	70.0		+			11
**910314**	Dialister microaerophilus UPII 345-E	Dmic	1.3	51.76	35.60			+			11
**309799**	Dictyoglomus thermophilum H-6-12	Dthe-6-12	2.0	52.02	33.70	78.0		+			11
**515635**	Dictyoglomus turgidum DSM 6724	Dtur	1.9	51.47	34.00			+			11
**352472**	Dictyostelium discoideum AX4	Ddis	34.2	47.44	22.46			+			1
**420778**	Diplodia seriata	Dser	37.3	51.20	56.50						1
**3046**	Dunaliella salina	Dsal	343.7	54.15	40.10						1
**999415**	Eggerthia catenaformis OT 569 = DSM 20559	Ecat	1.9	52.64	32.80			+			11
**445932**	Elusimicrobium minutum Pei191	Emin	1.6	50.23	40.00			+			11
**280463**	Emiliania huxleyi CCMP1516	Ehux	167.7	51.18	64.50			+			1
**885318**	Entamoeba histolytica HM-1:IMSS-A	Ehis	20.8	49.55	24.30						1
**226185**	Enterococcus faecalis V583	Efae	3.4	52.84	37.35	37.0	0.50				11
**1185651**	Enterovibrio norvegicus FF-454	Enor	5.4	53.22	47.60						11
**931890**	Eremothecium cymbalariae DBVPG#7215	Ecym	9.7	57.74	40.32			+			1
**284811**	Eremothecium gossypii ATCC 10895 (assembly ASM9102v4)	Egos		56.86	51.69						1
**314225**	Erythrobacter litoralis HTCC2594	Elit	3.1	48.36	63.10			+			11
**511145**	Escherichia coli str. K-12 substr. MG1655	EcolG1655	5.6	48.83	50.45	37.0					11
**316407**	Escherichia coli str. K-12 substr. W3110	EcolW3110	5.6	48.97	50.45	37.0	0.35	+			11
**360911**	Exiguobacterium sp. AT1b	EAT1	3.0	50.44	48.50			+			11
**589924**	Ferroglobus placidus DSM 10642	Fpla	2.2	50.05	44.10	85.0		+			11
**333146**	Ferroplasma acidarmanus fer1	Faci	1.9	52.66	36.50	40.0		+			11
**381764**	Fervidobacterium nodosum Rt17-B1	Fnod	1.9	55.00	35.00	70.0		+			11
**59374**	Fibrobacter succinogenes subsp. succinogenes S85	Fsuc	3.8	48.94	48.00	37.0					11

Tax Id	Species	Nick-name	Genome Size Mb	Genomic ENc'	Genomic GC%	Growth TempC	Growth Time Hours	In Phylo Tree	Is Endosymbiont	Endosymbiont Ref	Translation Tbl
**661478**	Fimbriimonas ginsengisoli Gsoil 348	Fgin	5.2	52.65	60.80			+			11
**1519565**	Fistulifera solaris	Fsol	49.7	56.79	45.60						1
**391603**	Flavobacteriales bacterium ALC-1	FALC	3.8	54.07	32.40						11
**1341181**	Flavobacterium limnosediminis JC2902	Flim	3.5	54.91	38.50	30.0					11
**402612**	Flavobacterium psychrophilum JIP02/86	Fpsy	2.9	55.34	32.50			+			11
**755732**	Fluviicola taffensis DSM 16823	Ftaf	4.6	54.77	36.50	20.0		+			11
**691883**	Fonticula alba	Falb	31.3	51.31	64.30			+			1
**1347342**	Formosa agariphila KMM 3901	Faga	4.2	53.70	33.60			+			11
**635003**	Fragilariopsis cylindrus CCMP1102	Fcyl	80.5	55.19	39.00						1
**767434**	Frateuria aurantia DSM 6220	Faur	3.6	46.11	63.40			+			11
**930946**	Fructobacillus fructosus KCTC 3544	Ffru	1.5	52.35	44.60			+			11
**469615**	Fusobacterium gonidiaformans ATCC 25563	Fgon	1.9	52.17	32.90						11
**190304**	Fusobacterium nucleatum subsp. nucleatum ATCC 25586	Fnuc	2.2	49.86	27.20	37.0	0.72	+			11
**469599**	Fusobacterium periodonticum 2_1_31	Fper	2.5	49.53	28.60						11
**555500**	Galbibacter marinus	Gmar	3.6	57.03	37.00			+			11
**130081**	Galdieria sulphuraria	Gsul	13.7	56.06	37.90						1
**553190**	Gardnerella vaginalis 409-05	Gvag	1.6	49.61	42.00			+			11
**49280**	Gelidibacter algens	Galg	4.5	56.43	37.30						11
**1630693**	Gemmata sp. SH-PL17	GSH-	9.0	49.95	64.20						11
**379066**	Gemmatimonas aurantiaca T-27	Gaur	4.6	50.34	64.30	30.0		+			11
**1379270**	Gemmatimonas phototrophica	Gpho	4.7	51.07	64.40	25.0					11
**861299**	Gemmatirosa kalamazoonesis	Gkal	7.5	43.90	72.64			+			11
**1121915**	Geoalkalibacter ferrihydriticus DSM 17813	Gfer	3.8	49.77	57.90	30.0		+			11
**235909**	Geobacillus kaustophilus HTA426	Gkau	3.6	48.08	51.99			+			11
**272567**	Geobacillus stearothermophilus 10	Gste	3.7	47.54	52.61	72.0					11
**398767**	Geobacter lovleyi SZ	Glov	4.0	50.06	54.77	35.0		+			11
**184922**	Giardia lamblia ATCC 50803	Glam	11.2	58.54	49.20			+			1

Tax Id	Species	Nick-name	Genome Size Mb	Genomic ENc'	Genomic GC%	Growth TempC	Growth Time Hours	In Phylo Tree	Is Endosymbiont	Endosymbiont Ref	Translation Tbl
1183438	Gloeobacter kilaueensis JS1	Gkil	4.7	51.52	60.50	28.0					11
251221	Gloeobacter violaceus PCC 7421	Gvio	4.7	50.38	62.00		72.00	+			11
290633	Gluconobacter oxydans 621H	Goxy	2.9	49.90	60.84	27.5	0.94	+			11
411154	Gramella forsetii KT0803	Gfor	3.8	56.12	36.60		4.17	+			11
391165	Granulibacter bethesdensis CGDNIH1	Gbet	2.7	50.36	59.10			+			11
905079	Guillardia theta CCMP2712	Gthe	87.1	54.90	52.90			+			1
944289	Gymnopus luxurians FD-317 M1	Glux	66.3	58.85	45.10			+			1
233412	Haemophilus ducreyi 35000HP	Hduc	1.7	50.03	38.20	36.0	1.80				11
866895	Halobacillus halophilus DSM 2266	Hhal	4.2	56.94	41.80			+			11
862908	Halobacteriovorax marinus SJ	Hmar	3.4	52.67	36.70			+	+	(15)	11
64091	Halobacterium salinarum NRC-1	HsalNRC-1	2.7	49.99	65.70	50.0	9.00				11
478009	Halobacterium salinarum R1	HsalR1	2.6	49.94	65.92	42.0		+			11
523841	Haloferax mediterranei ATCC 33500	Hmed	3.9	49.56	60.26			+			11
469382	Halogeometricum borinquense DSM 11551	Hbor	3.9	50.95	59.97	40.0		+			11
797210	Halopiger xanaduensis SH-6	Hxan	4.4	46.79	65.20			+			11
1033810	Haloplasma contractile SSD-17B	Hcon	3.4	55.86	32.30			+			11
362976	Haloquadratum walsbyi DSM 16790	Hwal	3.3	52.24	47.69		24.00	+			11
797114	Halosimplex carlsbadense 2-9-1	Hcar	4.7	47.11	67.70			+			11
373903	Halothermothrix orenii H 168	Hore	2.6	51.33	37.90	60.0		+			11
555778	Halothiobacillus neapolitanus c2	Hnea	2.6	52.68	54.70	35.0		+			11
85962	Helicobacter pylori 26695	Hpyl	1.7	48.19	38.90	37.0	2.40				11
316274	Herpetosiphon aurantiacus DSM 785	Haur	6.8	47.40	50.89		20.00	+			11
760142	Hippea maritima DSM 10411	Hmar10411	1.7	54.39	37.50	53.0		+			11
1321371	Holospora undulata HU1	Hund	1.4	55.06	36.10			+	+	(16)	11
1172194	Hydrocarboniphaga effusa AP103	Heff	5.2	45.27	65.20			+			11
608538	Hydrogenobacter thermophilus TK-6	Hthe	1.7	50.60	44.00			+			11
547144	Hydrogenobaculum sp. HO	HHO	1.6	51.57	34.80	58.0		+			11
945553	Hypholoma sublateritium FD-334 SS-4	Hsub	48.0	58.69	51.00			+			1

Tax Id	Species	Nick-name	Genome Size Mb	Genomic ENc'	Genomic GC%	Growth TempC	Growth Time Hours	In Phylo Tree	Is Endosymbiont	Endosymbiont Ref	Translation Tbl
945713	Ignavibacterium album JCM 16511	Ialb	3.7	53.23	33.90			+			11
583356	Ignisphaera aggregans DSM 17230	Iagg	1.9	51.32	35.70	95.0		+			11
1313172	Ilumatobacter coccineus YM16-304	Icoc	4.8	46.63	67.30			+			11
572544	Ilyobacter polytropus DSM 2926	Ipol	3.1	52.99	34.36	32.2		+			11
946077	Imtechella halotolerans K1	Ihal	3.1	55.90	35.50			+			11
743718	Isoptericola variabilis 225	Ivar	3.3	44.32	73.90			+			11
575540	Isosphaera pallida ATCC 43644	Ipal	5.5	53.13	62.45			+			11
926559	Joostella marina DSM 19592	Jmar	4.5	55.36	33.60			+			11
266940	Kineococcus radiotolerans SRS30216 = ATCC BAA-149	Krad	5.0	46.24	74.21	32.0		+			11
452652	Kitasatospora setae KM-6054	Kset	8.8	44.67	74.20			+			11
1125630	Klebsiella pneumoniae subsp. pneumoniae HS11286	Kpne	5.7	46.34	57.14						11
1006000	Kluyvera ascorbata ATCC 33433	Kasc	4.9	47.11	54.30			+			11
521045	Kosmotoga olearia TBF 19.5.1	Kole	2.3	56.34	41.50			+			11
1330330	Kosmotoga pacifica	Kpac	2.2	56.58	42.50	70.0					11
485913	Ktedonobacter racemifer DSM 44963	Krac	13.7	55.04	53.80	30.5		+			11
486041	Laccaria bicolor S238N-H82	Lbic	64.9	59.01	47.10			+			1
983544	Lacinutrix sp. 5H-3-7-4	L5H-	3.3	51.53	30.80			+			11
257314	Lactobacillus johnsonii NCC 533	Ljoh	2.0	52.22	34.60	30.0	0.90				11
220668	Lactobacillus plantarum WCFS1	Lpla	3.3	53.30	44.45	30.0	1.60				11
420890	Lactococcus garvieae Lg2	Lgar	2.0	52.24	38.80			+			11
272623	Lactococcus lactis subsp. lactis Il1403	Llac	2.4	51.51	35.30	40.0	0.70				11
911008	Leclercia adecarboxylata ATCC 23216 = NBRC 102595	Lade	4.8	46.92	55.80			+			11
398720	Leeuwenhoekiella blandensis MED217	Lbla	4.2	54.68	39.80			+			11
281090	Leifsonia xyli subsp. xyli str. CTCB07	Lxyl	2.7	49.36	68.30		5.00	+			11
347515	Leishmania major strain Friedlin	Lmaj	32.9	53.46	59.71				+	(17)	1
1439331	Lelliottia amnigena CHS 78	Lamn	4.6	47.60	54.30			+			11
313628	Lentisphaera araneosa HTCC2155	Lara	6.0	54.23	41.00			+			11
456481	Leptospira biflexa serovar Patoc strain 'Patoc 1 (Paris)'	Lbif	4.0	55.31	38.90			+			11

Tax Id	Species	Nick-name	Genome Size Mb	Genomic ENc'	Genomic GC%	Growth TempC	Growth Time Hours	In Phylo Tree	Is Endosymbiont	Endosymbiont Ref	Translation Tbl
**267671**	Leptospira interrogans serovar Copenhageni str. Fiocruz L1-130	Lint	4.7	54.65	35.01	29.0	9.00				11
**1441628**	Leptospirillum ferriphilum YSK	Lfer	2.4	51.77	54.60			+			11
**596323**	Leptotrichia goodfellowii F0264	Lgoo	2.3	51.46	31.60			+			11
**272626**	Listeria innocua Clip11262	Linn	3.1	53.51	37.35	33.5	0.60	+			11
**169963**	Listeria monocytogenes EGD-e	Lmon	2.9	53.37	38.00	33.5	1.00		+	(18)	11
**1574623**	Lyngbya confervoides BDU141951	Lcon	8.8	52.75	55.00						11
**242507**	Magnaporthe oryzae	Mory	41.0	56.33	51.59						1
**156889**	Magnetococcus marinus MC-1	Mmar	4.7	49.97	54.20			+			11
**1502293**	Marine Group I thaumarchaeote SCGC AAA799-N04	tSCG	1.3	51.73	34.20			+			11
**869210**	Marinithermus hydrothermalis DSM 14884	Mhyd	2.3	48.30	68.10			+			11
**443254**	Marinitoga piezophila KA3	Mpie	2.2	53.34	29.18			+			11
**504728**	Meiothermus ruber DSM 1279	Mrub	3.1	46.92	63.40	55.0		+			11
**754035**	Mesorhizobium australicum WSM2073	Maus	5.9	47.82	65.00			+			11
**660470**	Mesotoga prima MesG1.Ag.4.2	Mpri	3.0	54.94	45.50			+			11
**420247**	Methanobrevibacter smithii ATCC 35061	Msmi	1.9	52.58	31.00	38.5		+			11
**243232**	Methanocaldococcus jannaschii DSM 2661	Mjan	1.7	52.24	31.27	85.0	0.50	+			11
**267377**	Methanococcus maripaludis S2	MmarS2	1.8	52.50	33.30			+			11
**410358**	Methanocorpusculum labreanum Z	Mlab	1.8	52.38	50.00	37.0		+			11
**1201294**	Methanoculleus bourgensis MS2	Mbou	2.8	50.63	60.60			+			11
**28892**	Methanofollis liminatans DSM 4140	Mlim	2.5	50.00	61.00			+			11
**644295**	Methanohalobium evestigatum Z-7303	Meve	2.4	54.62	36.40	50.0		+			11
**867904**	Methanomethylovorans hollandica DSM 15978	Mhol	2.7	55.09	41.84	35.5		+			11
**190192**	Methanopyrus kandleri AV19	Mkan	1.7	52.31	61.20	98.0	0.83	+			11
**188937**	Methanosarcina acetivorans C2A	Mace	5.8	54.78	42.70	37.5					11
**213585**	Methanosarcina mazei S-6	Mmaz	4.1	53.11	41.40						11
**339860**	Methanosphaera stadtmanae DSM 3091	Msta	1.8	50.55	27.60	38.0		+			11
**521011**	Methanosphaerula palustris E1-9c	Mpal	2.9	51.93	55.40	30.0		+			11
**187420**	Methanothermobacter thermautotrophicus str. Delta H	Mthe	1.8	47.42	49.50	67.5	1.00	+			11

Tax Id	Species	Nick-name	Genome Size Mb	Genomic ENc'	Genomic GC%	Growth TempC	Growth Time Hours	In Phylo Tree	Is Endosymbiont	Endosymbiont Ref	Translation Tbl
481448	Methylacidiphilum infernorum V4	Minf	2.3	54.50	45.50			+			11
419610	Methylobacterium extorquens PA1	Mext	5.5	48.13	68.20	27.5	4.20	+			11
243233	Methylococcus capsulatus str. Bath	Mcap	3.3	49.27	63.60	45.0	1.87	+			11
449447	Microcystis aeruginosa NIES-843	Maer	5.8	54.59	42.30						11
564608	Micromonas pusilla CCMP1545	Mpus	22.0	48.66	65.70						1
500635	Mitsuokella multacida DSM 20544	Mmul	2.6	43.29	58.00			+			11
27923	Mnemiopsis leidyi	Mlei	155.9	57.30	39.10						1
548479	Mobiluncus curtisii ATCC 43063	Mcur	2.1	53.83	55.40			+			11
554373	Moniliophthora perniciosa FA553	Mper	26.7	58.52	47.70						1
431895	Monosiga brevicollis MX1	Mbre	41.7	53.88	54.33			+			1
1379858	Mucispirillum schaedleri ASF457	Msch	2.3	50.08	31.20			+			11
886377	Muricauda ruestringensis DSM 13258	Mrue	3.8	53.98	41.40			+			11
272631	Mycobacterium leprae TN	Mlep	3.3	55.25	57.80	37.0	240.00		+	(19)	11
83332	Mycobacterium tuberculosis H37Rv	Mtub	4.4	52.13	65.60	37.0	19.00		+	(20)	11
347257	Mycoplasma agalactiae PG2	Maga	0.9	52.20	29.70			+			4
243273	Mycoplasma genitalium G37	Mgen	0.6	54.12	31.70	37.0	12.00				4
272632	Mycoplasma mycoides subsp. mycoides SC str. PG1	Mmyc	1.2	49.28	24.00	37.0	2.20				11
272633	Mycoplasma penetrans HF-2	Mpen	1.4	50.21	25.70	37.0			+	(21)	4
272634	Mycoplasma pneumoniae M129	Mpne	0.8	52.37	40.00	37.0	6.00				4
272635	Mycoplasma pulmonis UAB CTIP	Mpul	1.0	50.52	26.60	37.0	1.50				11
744533	Naegleria gruberi strain NEG-M	Ngru		50.45	35.00			+			1
228908	Nanoarchaeum equitans	Nequ	0.5	53.05	31.60		0.75	+	+	(22)	11
1737403	Nanohaloarchaea archaeon SG9	Narc	1.1	51.11	46.40						11
457570	Natranaerobius thermophilus JW/NM-WN-LF	Nthe	3.2	56.40	36.29			+			11
797304	Natronobacterium gregoryi SP2	Ngre	3.8	48.80	62.20			+			11
122586	Neisseria meningitidis MC58	Nmen	2.3	48.07	51.50	36.0	0.72				11
45351	Nematostella vectensis	Nvec	356.6	59.19	41.90			+			1
1287680	Neofusicoccum parvum UCRNP2	Npar	42.6	50.99	56.70						1
102880	Neorhizobium galegae bv. orientalis str. HAMBI 540	Ngal	6.5	47.94	61.25			+			11

Tax Id	Species	Nick-name	Genome Size Mb	Genomic ENc'	Genomic GC%	Growth TempC	Growth Time Hours	In Phylo Tree	Is Endosymbiont	Endosymbiont Ref	Translation Tbl
0											
1189621	Nitritalea halalkaliphila LW7	Nhal	3.6	55.40	48.60			+			11
314278	Nitrococcus mobilis Nb-231	Nmob	3.6	53.69	59.90			+			11
1129897	Nitrolancea hollandica Lb	Nhol	3.9	52.82	62.60			+			11
228410	Nitrosomonas europaea ATCC 19718	Neur	2.8	53.08	50.70		18.50	+			11
436308	Nitrosopumilus maritimus SCM1	Nmar	1.6	51.08	34.20						11
926571	Nitrososphaera viennensis EN76	Nvie	2.5	50.75	52.70			+			11
1266370	Nitrospina gracilis 3-211	Ngra		48.61	56.10						11
330214	Nitrospira defluvii	Ndef	4.3	53.65	59.00			+			11
196162	Nocardioides sp. JS614	NJS6	5.3	46.58	71.48	30.0		+			11
592029	Nonlabens dokdonensis DSW-6	Ndok	3.9	55.55	35.30			+			11
63737	Nostoc punctiforme PCC 73102	Npun	9.1	55.96	41.34						11
670487	Oceanithermus profundus DSM 14977	Opro	2.4	45.17	69.79	60.0		+			11
221109	Oceanobacillus iheyensis HTE831	Oihe	3.6	54.93	35.70	30.0		+			11
203123	Oenococcus oeni PSU-1	Ooen	1.8	54.56	37.90	21.0	10.50	+			11
633147	Olsenella uli DSM 7084	Ouli	2.1	48.31	64.70	37.0		+			11
262768	Onion yellows phytoplasma OY-M	Oyel	0.9	51.44	27.80				+	(23)	11
452637	Opitutus terrae PB90-1	Oter	6.0	49.55	65.30			+			11
765420	Oscillochloris trichoides DG-6	Otri	4.4	50.42	59.10	29.0		+			11
436017	Ostreococcus lucimarinus	Oluc	13.2	50.73	60.44						1
926562	Owenweeksia hongkongensis DSM 17368	Ohon	4.0	55.54	40.20			+			11
1343739	Palaeococcus pacificus DY20341	Ppac	1.9	54.00	43.00			+			11
765952	Parachlamydia acanthamoebae UV-7	Paca	3.1	55.72	39.00			+			11
153151	Parageobacillus toebii	Ptoe	3.3	51.77	42.10	60.0					11
412030	Paramecium tetraurelia strain d4-2	Ptet	72.1	57.73	28.20			+			6
1618821	Parcubacteria group bacterium GW2011_GWA2_42_18	Par_42_18	0.6	52.80	41.60			+			11
1618840	Parcubacteria group bacterium GW2011_GWA2_47_10b	Par47_10b	0.8	53.23	47.10			+			11
161	Parcubacteria group bacterium	Par_47	0.6	53.01	46.80			+			11

Tax Id	Species	Nick-name	Genome Size Mb	Genomic ENc'	Genomic GC%	Growth TempC	Growth Time Hours	In Phylo Tree	Is Endosymbiont	Endosymbiont Ref	Translation Tbl
8841	GW2011_GWA2_47_12	_12									
1618924	Parcubacteria group bacterium GW2011_GWC2_40_31	Par_40_31	0.8	53.67	40.40			+			11
402881	Parvibaculum lavamentivorans DS-1	Plav	3.9	48.61	62.30			+			11
314260	Parvularcula bermudensis HTCC2503	Pber	2.9	52.99	60.70	30.0		+			11
747	Pasteurella multocida str. ATCC 43137	Pmul		49.34	40.30		1.00	+			11
423536	Perkinsus marinus ATCC 50983	Pmar50983	86.6	57.36	47.40			+			1
123214	Persephonella marina EX-H1	PmarEX-H1	2.0	46.05	37.12	73.0		+			11
403833	Petrotoga mobilis SJ95	Pmob	2.2	56.26	34.10		12.00	+			11
556484	Phaeodactylum tricornutum CCAP 1055/1	Ptri	27.5	57.66	48.84						1
298386	Photobacterium profundum SS9	Ppro	6.4	53.42	41.75	15.0	2.50	+			11
243265	Photorhabdus luminescens subsp. laumondii TTO1	Plum	5.7	54.82	42.80		0.50	+			11
1142394	Phycisphaera mikurensis NBRC 102666	Pmik	3.9	46.81	73.23			+			11
3218	Physcomitrella patens	Ppat	477.9	58.62	34.30						1
164328	Phytophthora ramorum	Pram	66.7	52.82	53.00			+			1
263820	Picrophilus torridus DSM 9790	Ptor	1.5	46.65	36.00	60.0	6.00	+			11
1227812	Piscirickettsia salmonis LF-89 = ATCC VR-1361	Psal	3.5	53.32	39.62			+	+	(24)	11
521674	Planctopirus limnophila DSM 3776	Plim	5.5	54.76	53.72			+			11
36329	Plasmodium falciparum 3D7	Pfal	23.3	57.62	19.36			+	+	(25)	1
4781	Plasmopara halstedii	Phal	75.3	56.75	45.70						1
1069680	Pneumocystis murina b123	Pmur	7.5	53.09	27.00						1
431947	Porphyromonas gingivalis ATCC 33277	Pgin	2.4	55.17	48.40	37.0	2.70				11
561896	Postia placenta Mad-698-R	Ppla	90.9	58.13	52.70			+			1
167546	Prochlorococcus marinus str. MIT 9301	Pmar	1.8	53.78	36.40		17.00	+			11
208964	Pseudomonas aeruginosa PAO1	Paer	6.3	43.26	66.60	27.5	0.50				11
96563	Pseudomonas stutzeri	Pstu	4.7	45.32	60.60	32.0					11
1123384	Pseudothermotoga hypogea DSM 11164 = NBRC 106472	Phyp	2.2	52.39	49.50	70.0					11
259536	Psychrobacter arcticus 273-4	Parc	2.7	50.60	42.80	22.0					11
335284	Psychrobacter cryohalolentis K5	Pcry	3.1	50.87	42.25			+			11

Tax Id	Species	Nick-name	Genome Size Mb	Genomic ENc'	Genomic GC%	Growth TempC	Growth Time Hours	In Phylo Tree	Is Endosymbiont	Endosymbiont Ref	Translation Tbl
1189619	Psychroflexus gondwanensis ACAM 44	Pgon	3.3	56.90	35.80	20.0		+			11
418459	Puccinia graminis f. sp. tritici	Pgra	88.7	58.01	43.80						1
178306	Pyrobaculum aerophilum str. IM2	PaerIM2	2.2	53.55	51.40	100.0	3.00	+			11
272844	Pyrococcus abyssi GE5	Paby	1.8	50.78	44.70	103.0	0.62				11
186497	Pyrococcus furiosus DSM 3638	Pfur	1.9	53.70	40.80	100.0	0.62	+			11
70601	Pyrococcus horikoshii OT3	Phor	1.7	52.96	41.90	98.0	0.62				11
1273541	Pyrodictium delaneyi	Pdel	2.0	54.00	53.90						11
694429	Pyrolobus fumarii 1A	Pfum	1.8	54.07	54.90	106.0		+			11
1223560	Pythium vexans DAOM BR484	Pvex	33.8	50.15	58.70						1
267608	Ralstonia solanacearum GMI1000	Rsol	5.8	44.93	66.96		4.00	+			11
365046	Ramlibacter tataouinensis TTB310	Rtat	4.1	42.50	70.00	30.0		+			11
145458	Rathayibacter toxicus	Rtox	2.3	55.18	61.50						11
288705	Renibacterium salmoninarum ATCC 33209	Rsal	3.2	55.88	56.30	15.0		+			11
1033991	Rhizobium leguminosarum bv. trifolii CB782	Rleg	6.9	48.10	61.17			+			11
243090	Rhodopirellula baltica SH 1	Rbal	7.1	52.94	55.40	28.0	10.00	+			11
258594	Rhodopseudomonas palustris CGA009	Rpal	5.3	45.97	66.00	27.5	9.00				11
518766	Rhodothermus marinus DSM 4252	Rmar	3.4	48.08	64.27	65.0		+			11
1165094	Richelia intracellularis HH01	Rint	3.2	55.08	33.70			+	+	(26)	11
313596	Robiginitalea biformata HTCC2501	Rbif	3.5	49.01	55.30	30.0		+			11
585394	Roseburia hominis A2-183	Rhom	3.6	49.70	48.50			+			11
383372	Roseiflexus castenholzii DSM 13941	Rcas	5.7	51.69	60.70	50.0		+			11
762948	Rothia dentocariosa ATCC 17931	Rden	2.5	53.87	53.70			+			11
582515	Rubidibacter lacunae KORDI 51-2	Rlac	4.2	54.56	56.20	30.0		+			11
559292	Saccharomyces cerevisiae S288c	Scer	12.2	56.61	38.16						1
405948	Saccharopolyspora erythraea NRRL 2338	Sery	8.2	46.03	71.10			+			11
435906	Salegentibacter salarius	Ssal	3.3	55.41	37.00						11
407035	Salinicoccus halodurans	Shal	2.8	52.87	44.50						11
45670	Salinicoccus roseus	Sros	2.6	51.05	50.00						11

Tax Id	Species	Nick-name	Genome Size Mb	Genomic ENc'	Genomic GC%	Growth TempC	Growth Time Hours	In Phylo Tree	Is Endosymbiont	Endosymbiont Ref	Translation Tbl
**1432562**	Salinicoccus sediminis	Ssed	2.6	50.88	48.70						11
**1033802**	Salinisphaera shabanensis E1L3A	Ssha	3.8	48.43	61.60			+			11
**1307761**	Salinispira pacifica	Spac	3.8	50.38	51.90	35.0		+			1
**99287**	Salmonella enterica subsp. enterica serovar Typhimurium str. LT2	Sent	5.1	48.94	51.88	37.0	0.40		+	(27)	11
**946362**	Salpingoeca rosetta	Salosetta	55.4	52.04	55.50			+			1
**695850**	Saprolegnia parasitica CBS 223.65	Spar	53.1	46.48	57.50			+			1
**578458**	Schizophyllum commune H4-8	Scom	38.5	55.02	57.40			+			1
**284812**	Schizosaccharomyces pombe (strain 972 / ATCC 24843)	Spom		55.70	36.04			+			1
**526218**	Sebaldella termitidis ATCC 33386	Ster	4.5	51.66	33.42			+			11
**211586**	Shewanella oneidensis MR-1	Sone	5.1	52.66	45.93		0.66	+			11
**1454006**	Siansivirga zeaxanthinifaciens CC-SAMT-1	Szea	3.3	53.62	33.50	27.5					11
**331113**	Simkania negevensis Z	Sneg	2.6	55.21	41.62			+	+	(28)	11
**886293**	Singulisphaera acidiphila DSM 18658	Saci	9.8	53.18	62.27			+			11
**266834**	Sinorhizobium meliloti 1021	Smel	6.7	49.74	62.16	27.5	1.50		+	(29)	11
**742818**	Slackia piriformis YIT 12062	Spir	2.1	50.11	57.60			+			11
**929556**	Solitalea canadensis DSM 3403	Scan	5.2	55.87	37.30			+			11
**479434**	Sphaerobacter thermophilus DSM 20745	Sthe	4.0	49.14	68.10			+			11
**158189**	Sphaerochaeta globosa str. Buddy	Sglo	3.3	55.24	48.90			+			11
**29656**	Spirodela polyrhiza	Spol	136.7	56.18	42.72						1
**645134**	Spizellomyces punctatus DAOM BR117	Spun	24.1	58.96	47.60			+			1
**1397361**	Sporothrix schenckii 1099-18	Ssch	32.4	52.84	55.00						1
**446470**	Stackebrandtia nassauensis DSM 44728	Snas	6.8	46.75	68.10			+			11
**93061**	Staphylococcus aureus subsp. aureus NCTC 8325	Saur	2.8	51.57	32.90	33.5	0.40				11
**176280**	Staphylococcus epidermidis ATCC 12228	Sepi	2.6	52.65	32.05	33.5	0.80				11
**519441**	Streptobacillus moniliformis DSM 12112	Smon	1.7	50.81	26.27			+			11
**160490**	Streptococcus pyogenes M1 GAS	Spyo	1.9	53.41	38.50	32.5	0.40				11
**227882**	Streptomyces avermitilis MA-4680 = NBRC 14893	Save	10.5	48.18	70.60						11

Tax Id	Species	Nick-name	Genome Size Mb	Genomic ENc'	Genomic GC%	Growth TempC	Growth Time Hours	In Phylo Tree	Is Endosymbiont	Endosymbiont Ref	Translation Tbl
**100226**	Streptomyces coelicolor A3(2)	Scoe	9.1	46.90	71.98	30.0	2.20	+			11
**1469144**	Streptomyces thermoautotrophicus	Strphicus	5.0	46.55	69.20	60.0					11
**762983**	Succinatimonas hippei YIT 12066	Ship	2.3	51.99	40.30			+			11
**429572**	Sulfolobus islandicus L.S.2.15	Sisl	2.7	55.84	35.10	80.0					11
**273063**	Sulfolobus tokodaii str. 7	Stok	2.7	54.82	32.80	80.0	6.00				11
**204536**	Sulfurihydrogenibium azorense Az-Fu1	Sazo	1.6	50.81	32.80	68.0		+			11
**432331**	Sulfurihydrogenibium yellowstonense SS-5	Syel	1.5	53.08	32.80	70.0					11
**326298**	Sulfurimonas denitrificans DSM 1251	Sden	2.2	52.73	34.50	22.5		+			11
**269084**	Synechococcus elongatus PCC 6301	Selo	2.7	53.98	55.50		6.10				11
**316279**	Synechococcus sp. CC9902	SCC9	2.2	55.61	54.20			+			11
**1148**	Synechocystis sp. PCC 6803	SPCC	3.9	51.92	47.35		12.00				11
**1209989**	Tepidanaerobacter acetatoxydans Re1	Tace	2.8	57.16	37.50			+			11
**312017**	Tetrahymena thermophila SB210	Tthe	103.0	56.34	22.30			+			6
**296543**	Thalassiosira pseudonana	Tpse	32.4	56.81	46.91			+			1
**1208320**	Thalassolituus oleivorans R6-15	Tole	3.9	52.37	46.60			+			11
**1177928**	Thalassospira profundimaris WP0211	Tpro	4.4	47.49	55.20			+			11
**1198115**	Thaumarchaeota archaeon SCGC AB-539-E09	Tarc	0.5	58.56	43.30			+			11
**353154**	Theileria annulata strain Ankara	Tann		57.63	32.55						1
**525903**	Thermanaerovibrio acidaminovorans DSM 6589	Taci	1.8	43.30	63.80	55.0		+			11
**525904**	Thermobaculum terrenum ATCC BAA-798	Tter	3.1	55.88	53.54	67.0		+			11
**269800**	Thermobifida fusca YX	Tfus	3.6	49.85	67.50	52.5		+			11
**469371**	Thermobispora bispora DSM 43833	Tbis	4.2	45.66	72.40			+			11
**391623**	Thermococcus barophilus MP	Tbar	2.1	53.84	41.71						11
**163003**	Thermococcus cleftensis	Tcle	2.0	45.96	55.80	85.0					11
**593117**	Thermococcus gammatolerans EJ3	Tgam	2.0	48.55	53.60	88.0					11
**1432656**	Thermococcus guaymasensis DSM 11113	Tgua	1.9	48.89	52.90	88.0					11
**195522**	Thermococcus nautili	Tnau	2.0	46.43	54.80						11

Tax Id	Species	Nick-name	Genome Size Mb	Genomic ENc'	Genomic GC%	Growth TempC	Growth Time Hours	In Phylo Tree	Is Endosymbiont	Endosymbiont Ref	Translation Tbl
638303	Thermocrinis albus DSM 14484	Talb	1.5	49.57	46.90			+			11
667014	Thermodesulfatator indicus DSM 15286	Tind	2.3	53.76	42.40	70.0		+			11
289377	Thermodesulfobacterium commune DSM 2178	Tcom	1.8	50.53	37.00	70.0		+			11
795359	Thermodesulfobacterium geofontis OPF15	Tgeo	1.6	49.84	30.60			+			11
289376	Thermodesulfovibrio yellowstonii DSM 11347	Tyel	2.0	50.81	34.10	65.0		+			11
309801	Thermomicrobium roseum DSM 5159	Tros	2.9	53.14	64.26	70.0		+			11
273075	Thermoplasma acidophilum DSM 1728	Taci1728	1.6	51.06	46.00	59.0	2.50	+			11
273116	Thermoplasma volcanium GSS1	Tvol	1.6	55.00	39.90	60.0	2.50				11
768679	Thermoproteus tenax Kra 1	Tten	1.8	51.18	55.10						11
484019	Thermosipho africanus TCF52B	Tafr	2.0	53.57	30.80	75.0		+			11
391009	Thermosipho melanesiensis BI429	Tmel	1.9	55.29	31.40	70.0					11
1298851	Thermosulfidibacter takaii ABI70S6	Ttak	1.8	53.49	43.00						11
243274	Thermotoga maritima MSB8	Tmar	1.9	50.62	46.20	80.0	1.20	+			11
648996	Thermovibrio ammonificans HB-1	Tamm	1.8	45.66	52.12	75.0		+			11
580340	Thermovirga lienii DSM 17291	Tlie	2.0	54.93	47.10	58.0		+			11
498848	Thermus aquaticus Y51MC23	Taqu	2.3	44.81	68.04	67.5					11
751945	Thermus oshimai JL-2	Tosh	2.4	44.39	68.60			+			11
300852	Thermus thermophilus HB8	TtheHB8	2.1	44.13	69.49		2.50				11
768671	Thiocapsa marina 5811	Tmar5811	5.6	50.99	64.10			+			11
381306	Thiohalorhabdus denitrificans	Tden	2.9	44.19	68.90			+			11
1177931	Thiovulum sp. ES	TES	2.1	51.48	33.00			+			11
1245935	Tolypothrix campylonemoides VB511288	Tcam	9.5	56.42	45.10						11
508771	Toxoplasma gondii ME49	Tgon	65.7	56.40	52.29			+	+	(30)	1
243275	Treponema denticola ATCC 35405	Tden35405	2.8	55.05	37.90	36.0	5.00	+			11
203124	Trichodesmium erythraeum IMS101	Tery	7.8	54.62	34.10		25.00	+			11
412133	Trichomonas vaginalis G3	Tvag	176.4	53.67	32.90			+			1
10228	Trichoplax adhaerens	Tadh	105.6	57.34	34.50			+			1
203267	Tropheryma whipplei str. Twist	Twhi	0.9	57.37	46.30	37.0	28.00		+	(31)	11
649638	+pera radiovictrix DSM 17093	Trad	3.3	47.28	68.10	50.0		+			11

Tax Id	Species	Nick-name	Genome Size Mb	Genomic ENc'	Genomic GC%	Growth TempC	Growth Time Hours	In Phylo Tree	Is Endosymbiont	Endosymbiont Ref	Translation Tbl
**5693**	Trypanosoma cruzi	Tcru	89.9	57.03	51.70				+	(32)	1
**1157490**	Tumebacillus flagellatus	Tfla	4.9	46.11	56.50			+			11
**883169**	Turicella otitidis ATCC 51513	Toti	2.1	44.36	71.00			+			11
**505682**	Ureaplasma parvum serovar 3 str. ATCC 27815	Upar	0.8	47.77	25.50			+			11
**436907**	Vanderwaltozyma polyspora DSM 70294	Vpol	14.7	51.58	33.00			+			1
**263358**	Verrucosispora maris AB-18-032	Vmar	6.7	46.99	70.89			+			11
**388396**	Vibrio fischeri MJ11	Vfis	4.3	50.71	38.37	24.0	0.30	+			11
**223926**	Vibrio parahaemolyticus RIMD 2210633	Vpar	5.2	51.82	45.40	25.0	0.20				11
**196600**	Vibrio vulnificus YJ016	Vvul	5.3	52.79	46.67	25.0	0.16				11
**3067**	Volvox carteri	Vcar	137.7	57.51	55.30						1
**572478**	Vulcanisaeta distributa DSM 14429	Vdis	2.4	49.56	45.40	87.5		+			11
**4927**	Wickerhamomyces anomalus NRRL Y-366-8	Wano	14.1	48.08	35.00						1
**1041607**	Wickerhamomyces ciferrii	Wcif	15.9	46.02	30.40						1
**641526**	Winogradskyella psychrotolerans RS-3	Wpsy	4.3	54.66	33.50			+			11
**1116230**	Wolbachia pipientis wAlbB	Wpip	1.2	56.57	33.80				+	(33)	11
**273121**	Wolinella succinogenes DSM 1740	Wsuc	2.1	50.32	48.50		1.00	+			11
**1304892**	Xanthomonas axonopodis Xac29-1	Xaxo	5.3	45.38	64.72		7.00	+			11
**190485**	Xanthomonas campestris pv. campestris str. ATCC 33913	Xcam	5.1	45.06	65.10	27.5	3.00				11
**160492**	Xylella fastidiosa 9a5c	Xfas9a5c	2.7	54.70	52.64	27.0	5.13				11
**155920**	Xylella fastidiosa subsp. sandyi Ann-1	XfasAnn-1	2.7	54.52	52.64	27.0		+			11
**655815**	Zunongwangia profunda SM-A87	Zpro	5.1	56.34	36.20			+			11
**1047168**	Zymoseptoria brevis	Zbre	36.1	56.50	51.20						1
**336722**	Zymoseptoria tritici	Ztri	39.7	56.39	52.12						1
**1619079**	candidate division TM6 bacterium GW2011_GWF2_32_72	dTM6	1.0	54.19	32.70			+			11

Explanation of table columns: **TaxId** – NCBI taxonomic identifier for species. **Species** – Species name and strain. **Nick-name** – Short species identifiers (used in other figures). **GenomeSizeMb** – Annotate genome size in megabases. **Genomic-ENc'** – ENc' value for species. **Genomic-GC%** – Mean GC% for species. **GrowthTempC** – Optimum growth-temperature (°C). **GrowthTimeHours** – Minimum growth time (hours). **InPhyloTree** – Is species included in species tree used for GLS analysis. **IsEndosymbiont** –

Is species considered a known endosymbiont/intracellular organism. **EndosymbiontRef** – Reference for endosymbiont status (in "Supplementary references"). **TranslationTbl** – Nuclear genetic code for species.

Figure S1. Correlations of traits with ΔLFE are not present in its individual components

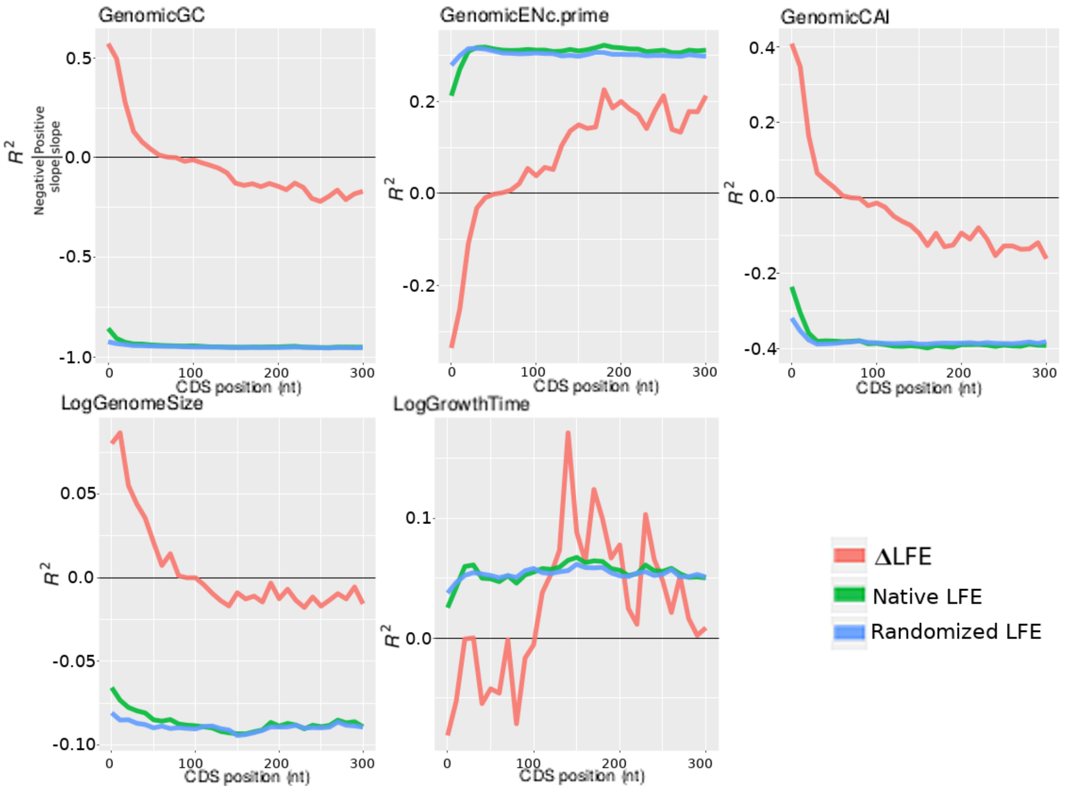

Coefficient of determination (R^2) for GLS regression of the specified trait with ΔLFE and its components (ΔLFE - red; native LFE - green; randomized LFE - blue), at different positions relative to CDS start. Negative R^2 values indicate negative regression slope. The observed correlation between each trait and ΔLFE is not observed with the individial components (native or randomized LFE).

Figure S2. The ΔLFE profile is more conserved than other genomic traits

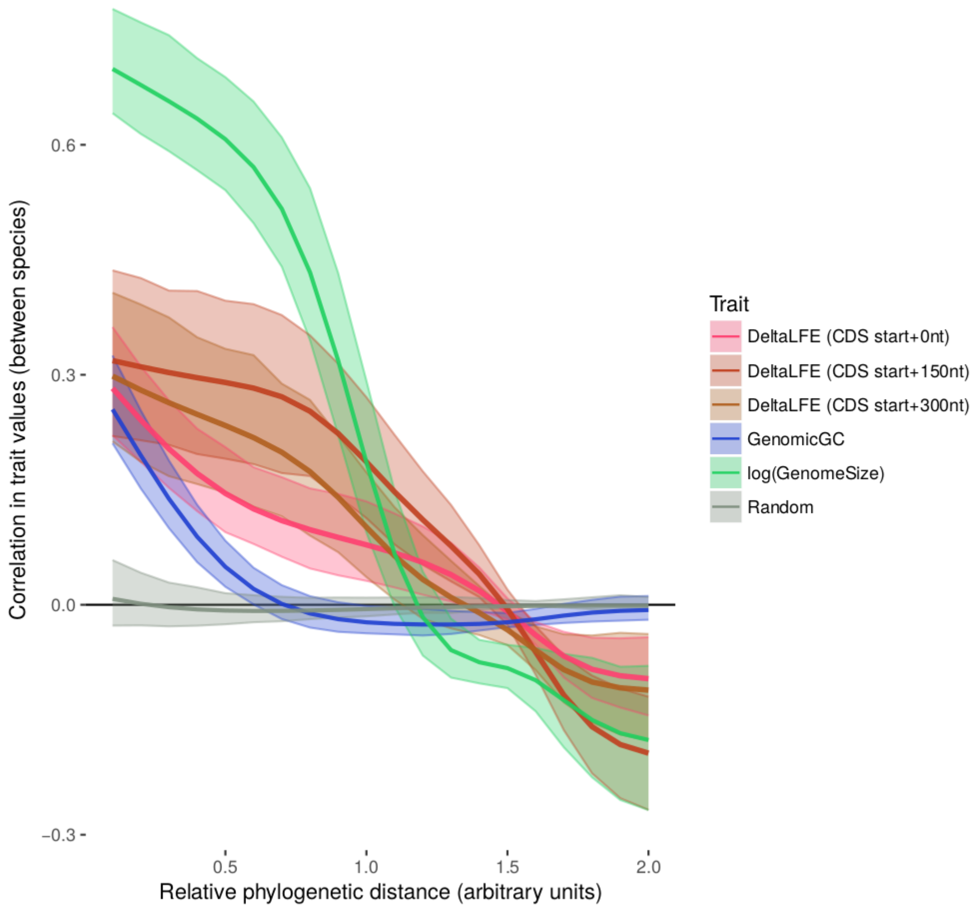

Correlation (expressed using Moran's I coefficient) between the values of different traits, for pairs of species of different phylogenetic distances. Genomic-GC% is positively correlated at short distances. ΔLFE values (at different positions relative to CDS start) are more strongly correlated than genomic-GC% at most phylogenetic distances, but less correlated than genome sizes. Confidence intervals represent 95% confidence calculated using 500 bootstrap samples. The 'Random' trait is a normally-distributed uncorrelated variable.

Figure S3. Local CUB vs. Local ΔLFE

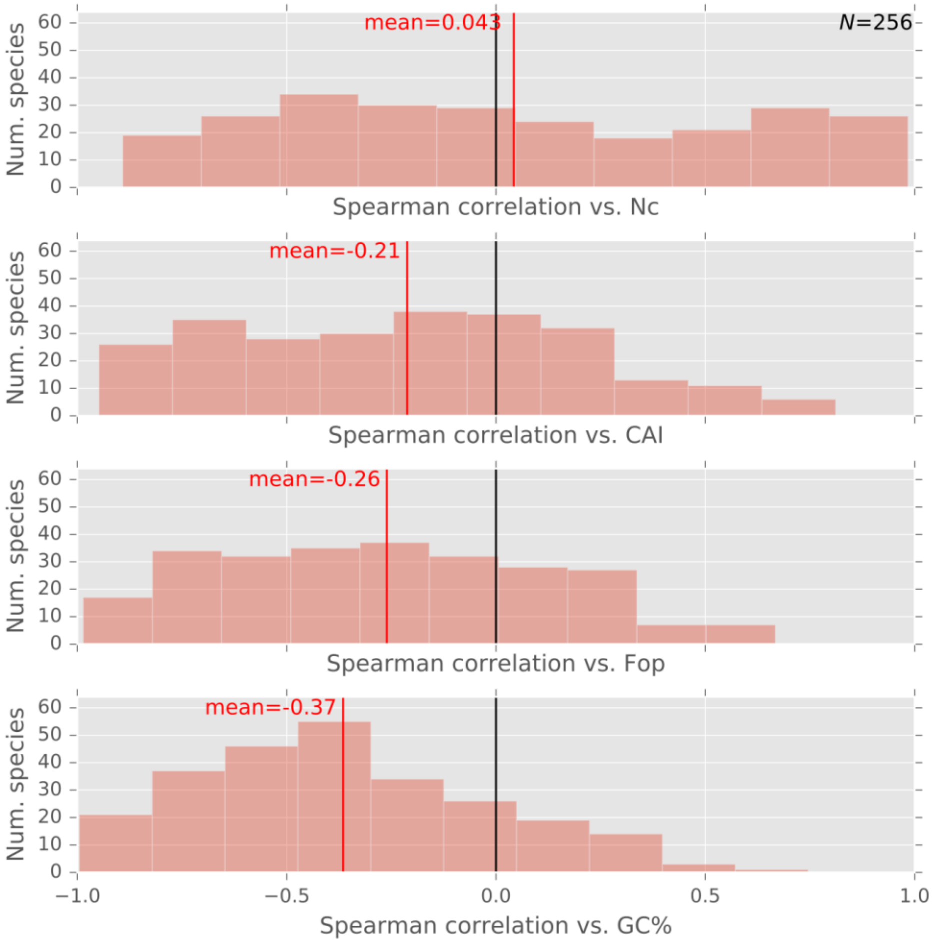

Spearman correlations between the ΔLFE profile (i.e., mean value for a given species at each position relative to CDS start) and the corresponding CUB profiles (i.e., CUB for all CDSs for a given species at this position relative to CDS start) show no direct correspondence, indicating the ΔLFE profiles are not simply a side-effect of direct selection operating on CUB in different CDS regions. CUB measures were calculated for the sequences contained in the same 40nt windows, starting at positions 0-300nt relative to CDS start, with all the sequences for each species concatenated, for a random sample of N=256 species.

From top to bottom, Nc (Effective Number of Codons), CAI (Codon Adaptation Index), Fop (Frequency of Optimal Codons), GC% (GC-content).

Figure S4. Comparison between ΔLFE calculated using CDS-wide and position-specific (“vertical”) randomization

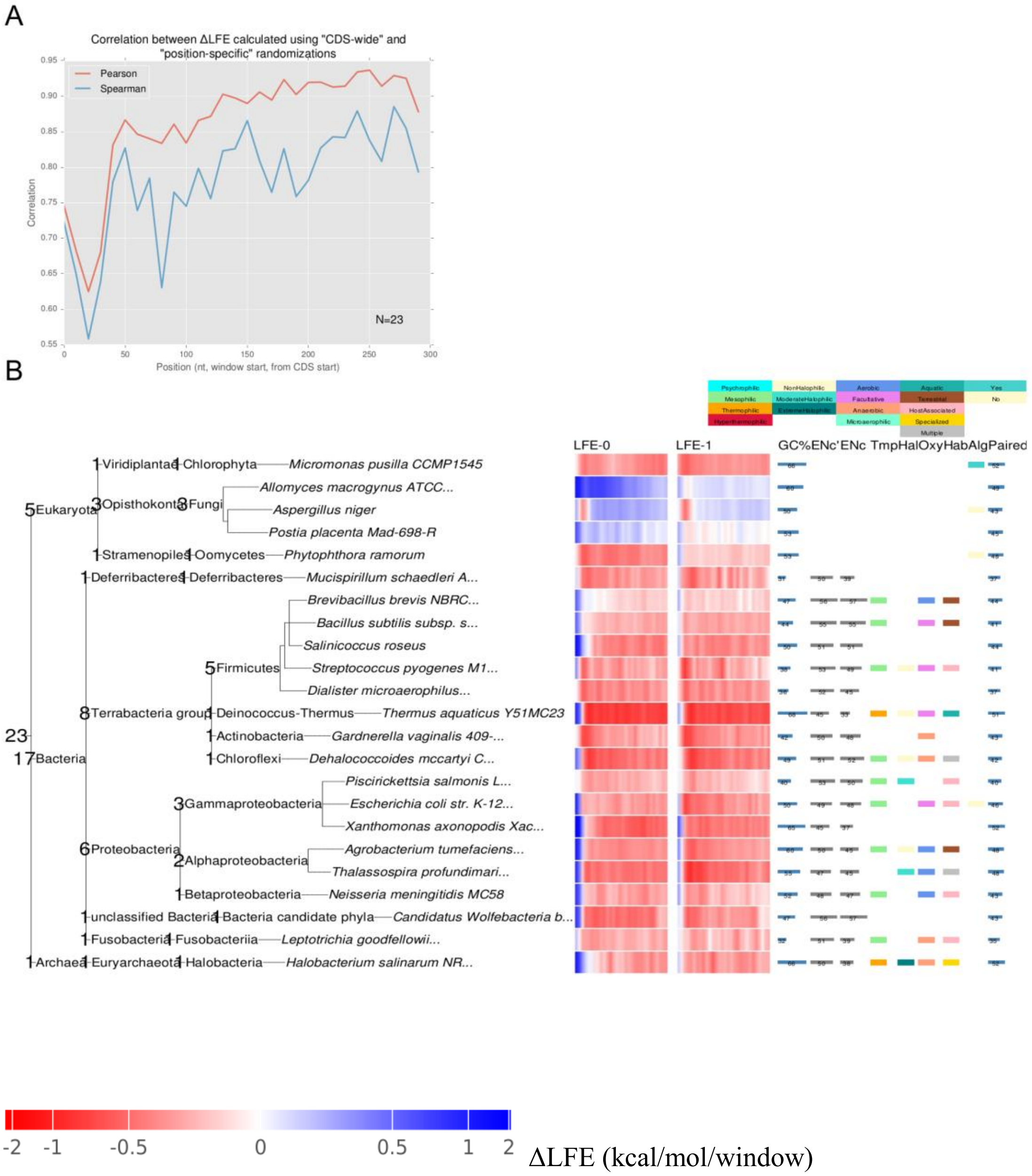

Position-specific randomization (maintaining the encoded AA sequences as well as the codon frequency in each position (across all CDSs belonging to the same species) yields qualitatively similary results to the CDS-wide randomization used throughout the rest of this paper. This supports the conclusion that the observed ΔLFE profiles are not merely a result of position-dependent biases in codon composition. **A** Correlation between ΔLFE calculated using “CDS-wide” and “position-specific” randomizations (see methods), at each position relative to CDS start. Correlations were calculated for a random sample (*N*=23) of species. **B** Comparison of individual mean ΔLFE profiles calculated using “CDS-wide” (LFE-0) and “position-specific” (LFE-1) randomizations.

Figure S5. ΔLFE is stronger in highly expressed genes and genes encoding for highly abundant proteins

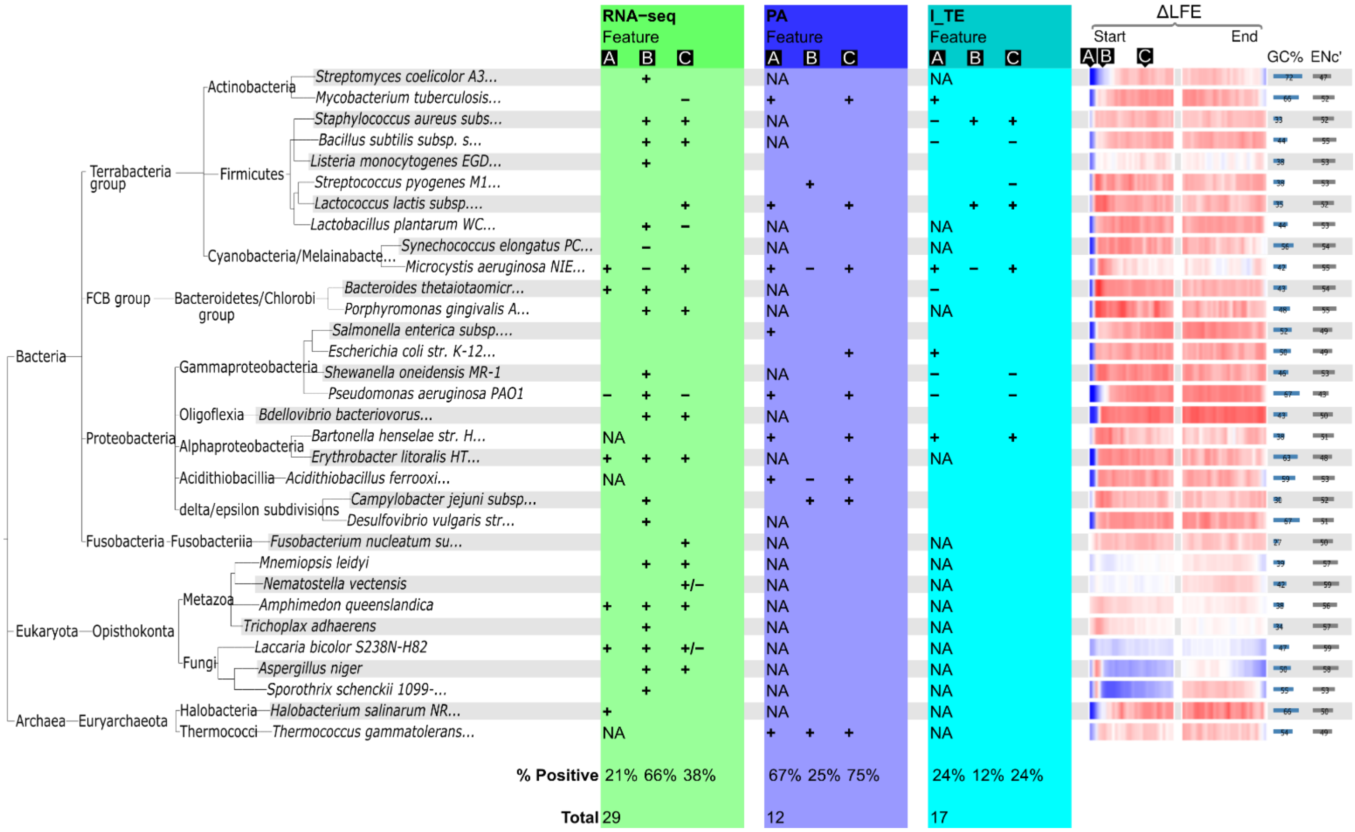

The observed average ΔLFE features are generally more prominent in highly expressed genes and in genes encoding for highly abundant proteins. This figure shows results for 32 species, plotted according to their position on a taxonomic tree (Left). Results are summarized for highly expressed genes based on transcriptomic RNA-sequencing for 29 species (green region) and for experimentally measured protein-abundance (PA) for 12 species (blue region). Also shown are results for purely computational translation elongation optimization scores, I_TE(34) (cyan region). For each evidence type, results are shown for regions [**A**]-[**C**] (as defined in Fig. 1A).

Note that the increase in regions [**B**]-[**C**] in highly expressed genes in *Listeria monocytogenes* and *Bacillus subtilis* was previously reported in (35 Figs. 4,5). They report similar results in *E. coli* region [**B**] (which is marginally significant in our data).

For each region, the following symbols identify the relation between the "high" and "low" groups:

+ The trend observed in this region (i.e., increased or decreased folding strength) is more extreme in highly expressed or highly abundant genes.

– The trend observed in this region (i.e., increased or decreased folding strength) is less extreme in highly expressed or highly abundant genes (or the opposite trend is observed).

(no symbol)	There is no consistent and statistically significant difference between the groups (or there is no ΔLFE trend in this region).
**+/−**	Inconsistent or contradictory results in different positions.
**NA**	Data was not available for this species.

Determination of each symbol (**+/−)** was based on results of a Mann-Whitney U test between the two groups of genes across the appropriate region, once for each direction (with the null hypothesis being that a value sampled from one group is not likely to be greater than an item from the other group). Fraction of positive species and total number of species are shown below for each evidence type.

On the right side, the table shows a summary of relevant characteristics for each species. From right to left - the average ΔLFE "heat-map" for this species, for the 300nt region at the beginning (left) and end (right) of the CDS, the average GC% for the genome, and the average ENc' (CUB) for the genome.

RNA sequencing data was obtained through ENA from the experiments detailed in the table below. Species were chosen based on availability of data using for the same strain or a closely related strain and using short-read sequencing technology compatible with the pipeline described here. Experiments are transcriptomic in their design and the control sample from each experiment was used (from the logarithmic growth phase if possible).

Normalized read counts were calculated as follows. Trimmomatic(36) version 0.38, using the single-end or paired-end mode and the Illumina adapters, sliding window with window size 4nt and quality threshold 15, leading and trailing below 3 and minimum length of 36nt. Reads were mapped to reference genomes obtained from Ensembl genomes (37), except for *E. coli* that was obtained from NCBI(38). Reads were mapped to genomic positions with Bowtie2(39) version 2.3.4.3 using local alignment with the default settings. Read were then assigned to coding sequences using htseq-count(40) version 0.11.2 in union mode with non-unique matches included and ignoring expected strand. Normlalized counts for each CDS were finally obtained by dividing by the CDS length. Genes were divided to the "low" and "high" groups based on the median normalized read count for each species, with genes having no reads counted as 0.

PA results were obtained from PaxDB(41) using the "Integrated" dataset. Genes were divided to the "low" and "high" groups based on the median count for each species, with genes having no reads counted as 0.

I_TE (34), a CUB measure designed to measure codon optimization for translation elongation, was computed using DAMBE7(42) based on the included codon frequency tables for each species.

Sources for RNA-seq data

Species	NCBI TaxId	SRA accessions			Reference
		Run accession	Sample accession	Series accession	
**Streptomyces coelicolor A3(2)**	100226	SRR6782725	GSM3022567	GSE111126	(43)
**Mycobacterium tuberculosis H37Rv**	83332	SRR7504771	GSM3262910	GSE116859	(44)
**Staphylococcus aureus subsp. aureus NCTC 8325**	93061	SRR10355925	GSM4142361	GSE139494	(45)
**Bacillus subtilis subsp. subtilis str. 168**	224308	SRR3466199	GSM2137056	GSE80786	(46)
**Listeria monocytogenes EGD-e**	169963	SRR9167346	GSM3758086	GSE130971	(47)
**Streptococcus pyogenes M1 GAS**	160490	SRR8752237	GSM3679080	GSE128534	(48)

Species	NCBI TaxId	SRA accessions			Reference
**Lactococcus lactis subsp. lactis Il1403**	272623	SRR6308319	GSM2861629	GSE107135	
**Lactobacillus plantarum WCFS1**	220668	SRR7276154	GSM3178379	GSE115448	(49)
**Synechococcus elongatus PCC 6301**	269084	SRR10421010	GSM4154628	GSE140121	
**Microcystis aeruginosa NIES-843**	449447	SRR6363352	GSM2881838	GSE107852	(50)
**Bacteroides thetaiotaomicron VPI-5482**	226186	SRR8874378	GSM3716497	GSE129572	(51)
**Porphyromonas gingivalis ATCC 33277**	431947	SRR8788484	GSM3688067	GSE128899	(52)
**Salmonella enterica subsp. enterica serovar**	99287	SRR8269283	GSM3498247	GSE123195	(53)
**Escherichia coli str. K-12**	511145	SRR9919224	GSM4013462	GSE135516	(54)
**Shewanella oneidensis MR-1**	211586	SRR6170086	GSM2810879	GSE104952	(55)
**Pseudomonas aeruginosa PAO1**	208964	SRR10259076	GSM4117161	GSE138731	
**Bdellovibrio bacteriovorus HD100**	264462	SRR3605968	GSM2181634	GSE82035	(56)
**Erythrobacter litoralis HTCC2594**	314225	SRR8571520	GSM3603106	GSE126532	(57)
**Campylobacter jejuni subsp. jejuni**	192222	SRR9640970	GSM3927297	GSE133783	(58)
**Desulfovibrio vulgaris str. Hildenborough**	882	SRR5871740	GSM2718521	GSE101911	(59)
**Fusobacterium nucleatum subsp. nucleatum ATCC 25586**	190304	SRR9010011	GSM3752362	GSE130714	(60)
**Mnemiopsis leidyi**	27923	SRR6768520	GSM3021563	GSE111068	(61)
**Nematostella vectensis**	45351	SRR5839397	GSM2710835	GSE101604	(62)
**Amphimedon queenslandica**	400682	SRR6768514	GSM3021561	GSE111068	(61)
**Trichoplax adhaerens**	10228	SRR6768521	GSM3021564	GSE111068	(61)
**Laccaria bicolor S238N-H82**	486041	SRR6709977	GSM2992239	GSE110485	(63)
**Aspergillus niger**	5061	SRR7772837	GSM3369695	GSE119311	(64)
**Sporothrix schenckii 1099-18**	1397361	SRR9602168	GSM3905741	GSE133322	
**Halobacterium salinarum NRC-1**	64091	SRR5651597	GSM2651026	GSE99730	(65)

Figure S6. Unsupervised discovery of ΔLFE profile regions

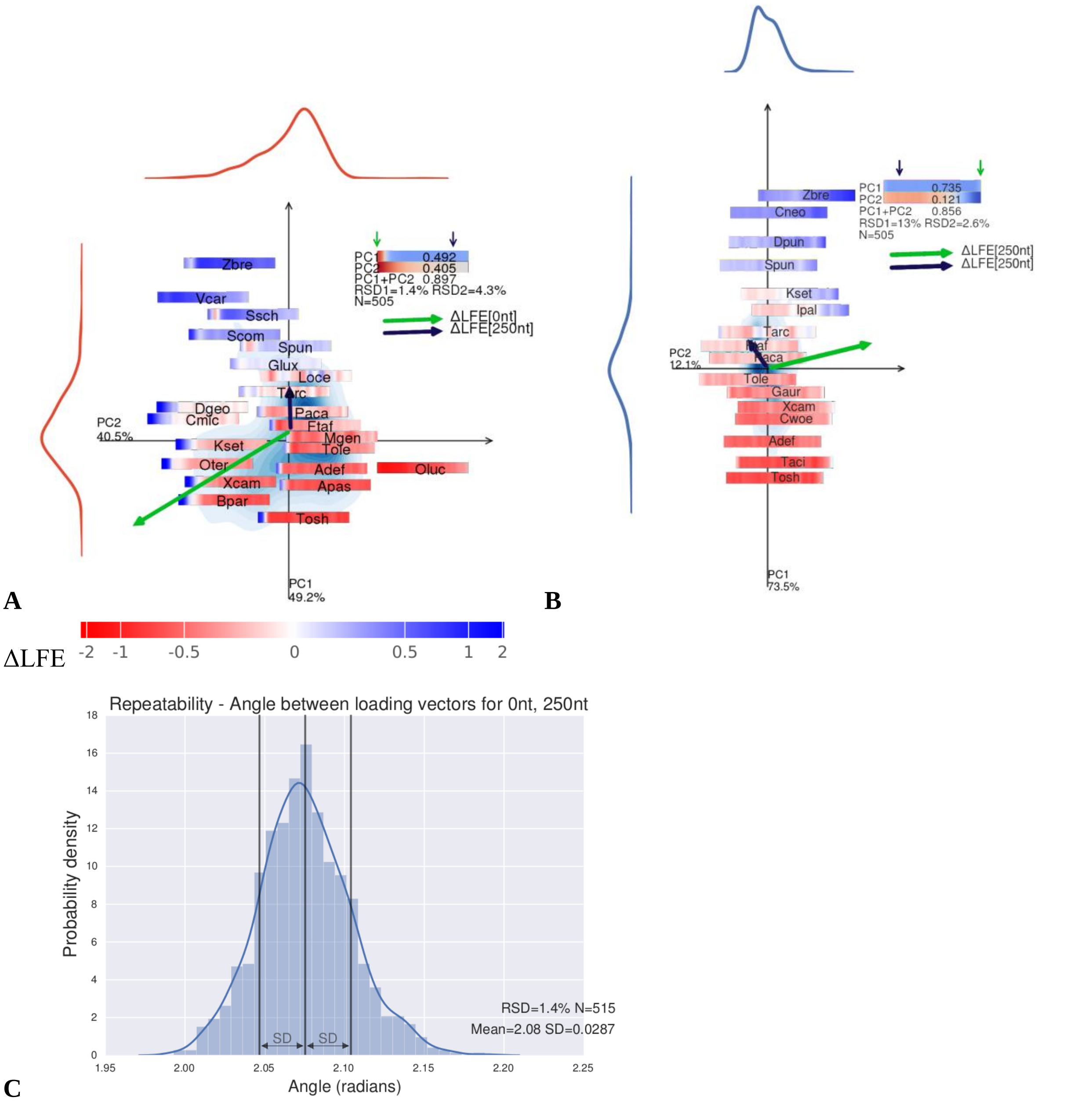

Principal Component Analysis (PCA) of the ΔLFE profiles uncovers two components, with different relative weights for the CDS-edge and mid-CDS regions. **A** PCA plot for ΔLFE profiles at positions 0-300nt relative to CDS start (represented as vectors of length 31), shown by plotting each ΔLFE profile in its position in PCA space (with 2 dimensions), with overlapping profiles hidden to avoid clutter. The density of profiles in each region is illustrated using shading and the marginal distributions are shown on the axes. Loading vectors for positions 0nt and 250nt (relative to CDS start) are shown. To verify this analysis is robust, bootstrapping using 1000 repeats was used to measure the following values: RSD1 - Relative standard-deviation (SD/mean) for the angle between the loading vectors shown (i.e., those for

ΔLFE profile positions 0nt and 250nt). Distribution of angles shown in **C**. RSD2 - Relative standard-deviation (SD/mean) for the explained variance of PC1. **B** PCA plot for ΔLFE profiles at positions 0-300nt relative to CDS end (created using the same method as **A**). **C** Distribution of angles between shown loading vectors (i.e., those for ΔLFE profile positions 0nt and 250nt) using 1000 bootstrap samples. The distribution mean is 2.08 radians (119°) and the relative standard deviation (also shown as RSD1 on **A**) is 1.4%.

This procedure was repeated for all species and for each domain individially (see also Fig. 4D). In each case, the first two PCs explain >80% of the variation. The loading vectors for positions 0nt and 250nt are not parralel nor orthogonal (and this is robust to sampling and persists in smaller groups, see Fig. 4D), indicating some level of dependence between the two positions (also indicated in Fig. 3E, Fig. S6).

Figure S7. ΔLFE profiles for all species

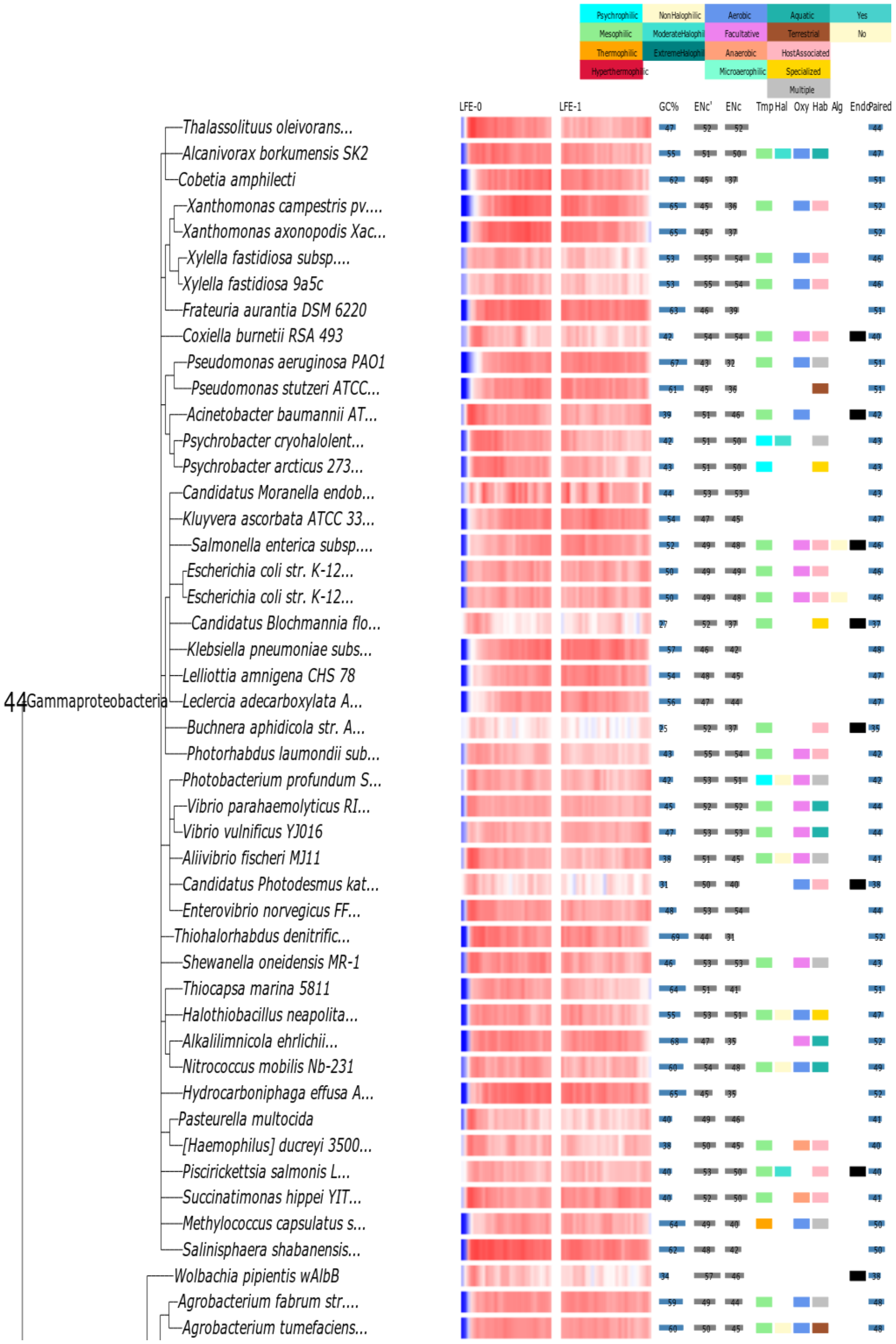

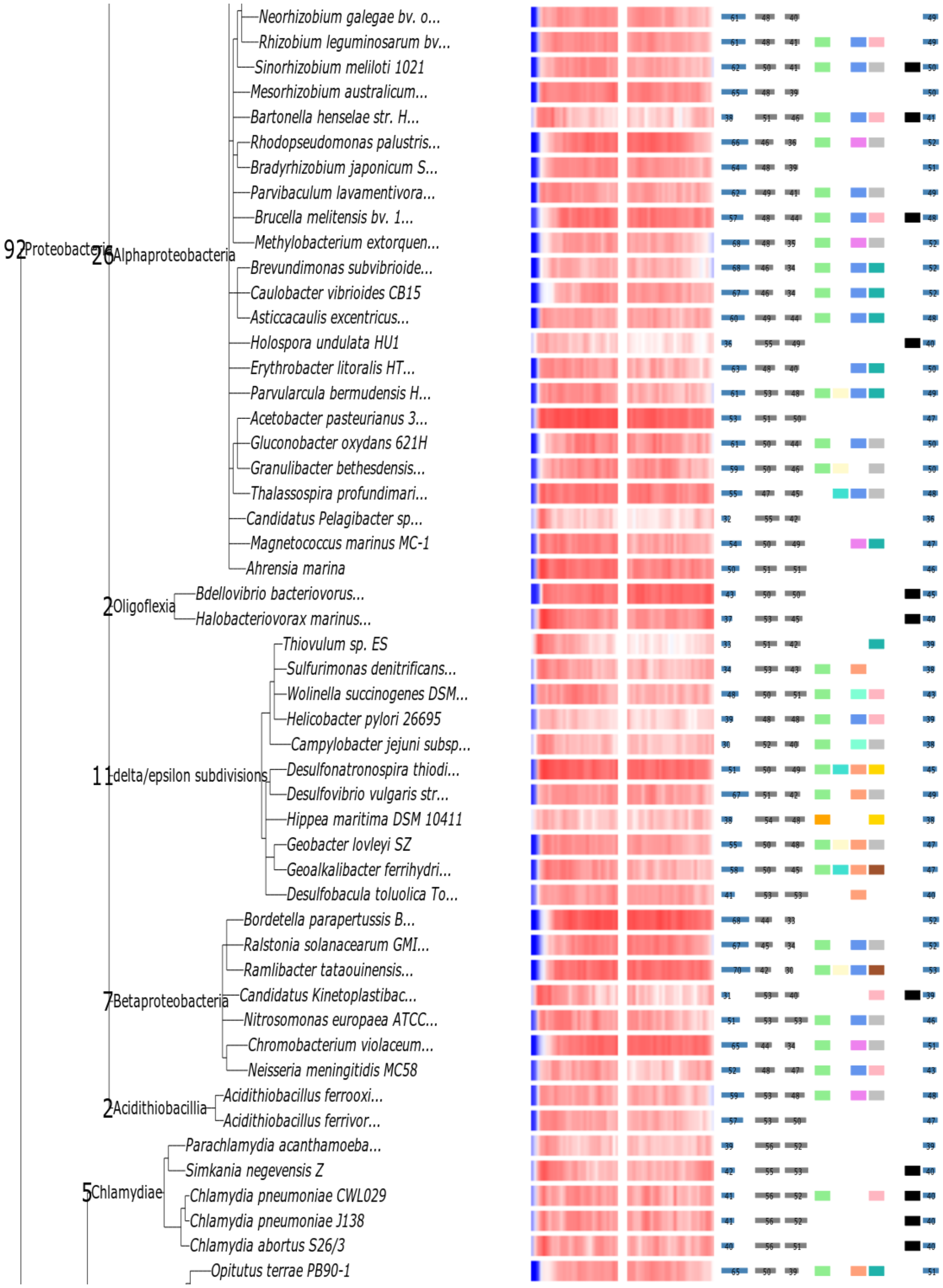
92 Proteobacteria
26 Alphaproteobacteria
Neorhizobium galegae bv. o...
Rhizobium leguminosarum bv...
Sinorhizobium meliloti 1021
Mesorhizobium australicum...
Bartonella henselae str. H...
Rhodopseudomonas palustris...
Bradyrhizobium japonicum S...
Parvibaculum lavamentivora...
Brucella melitensis bv. 1...
Methylobacterium extorquen...
Brevundimonas subvibrioide...
Caulobacter vibrioides CB15
Asticcacaulis excentricus...
Holospora undulata HU1
Erythrobacter litoralis HT...
Parvularcula bermudensis H...
Acetobacter pasteurianus 3...
Gluconobacter oxydans 621H
Granulibacter bethesdensis...
Thalassospira profundimari...
Candidatus Pelagibacter sp...
Magnetococcus marinus MC-1
Ahrensia marina
2 Oligoflexia
Bdellovibrio bacteriovorus...
Halobacteriovorax marinus...
11 delta/epsilon subdivisions
Thiovulum sp. ES
Sulfurimonas denitrificans...
Wolinella succinogenes DSM...
Helicobacter pylori 26695
Campylobacter jejuni subsp...
Desulfonatronospira thiodi...
Desulfovibrio vulgaris str...
Hippea maritima DSM 10411
Geobacter lovleyi SZ
Geoalkalibacter ferrihydri...
Desulfobacula toluolica To...
7 Betaproteobacteria
Bordetella parapertussis B...
Ralstonia solanacearum GMI...
Ramlibacter tataouinensis...
Candidatus Kinetoplastibac...
Nitrosomonas europaea ATCC...
Chromobacterium violaceum...
Neisseria meningitidis MC58
2 Acidithiobacillia
Acidithiobacillus ferrooxi...
Acidithiobacillus ferrivor...
5 Chlamydiae
Parachlamydia acanthamoeba...
Simkania negevensis Z
Chlamydia pneumoniae CWL029
Chlamydia pneumoniae J138
Chlamydia abortus S26/3
Opitutus terrae PB90-1

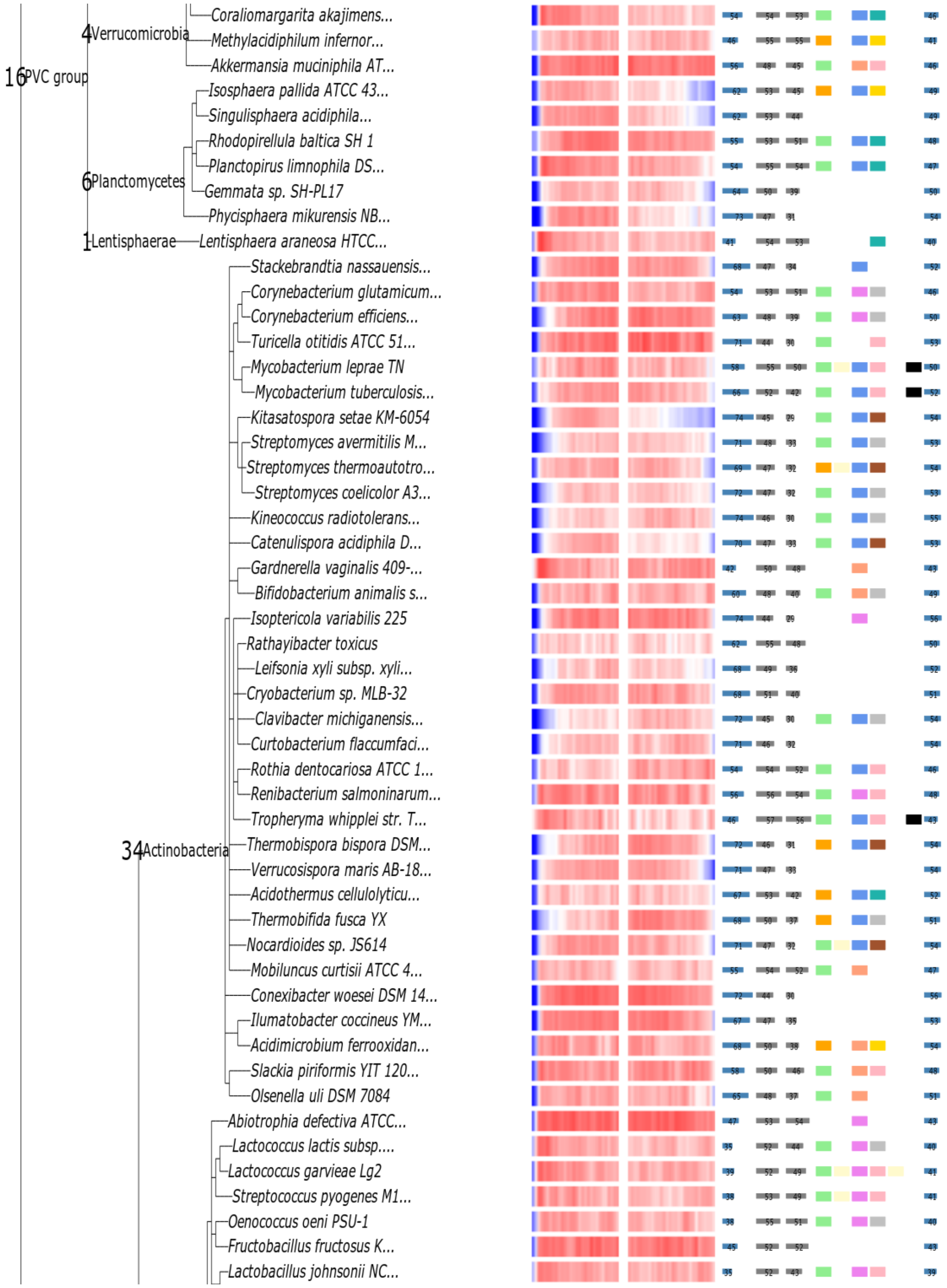
16 PVC group
4 Verrucomicrobia
Coraliomargarita akajimens...
Methylacidiphilum infernor...
Akkermansia muciniphila AT...
6 Planctomycetes
Isosphaera pallida ATCC 43...
Singulisphaera acidiphila...
Rhodopirellula baltica SH 1
Planctopirus limnophila DS...
Gemmata sp. SH-PL17
Phycisphaera mikurensis NB...
1 Lentisphaerae
Lentisphaera araneosa HTCC...
34 Actinobacteria
Stackebrandtia nassauensis...
Corynebacterium glutamicum...
Corynebacterium efficiens...
Turicella otitidis ATCC 51...
Mycobacterium leprae TN
Mycobacterium tuberculosis...
Kitasatospora setae KM-6054
Streptomyces avermitilis M...
Streptomyces thermoautotro...
Streptomyces coelicolor A3...
Kineococcus radiotolerans...
Catenulispora acidiphila D...
Gardnerella vaginalis 409-...
Bifidobacterium animalis s...
Isoptericola variabilis 225
Rathayibacter toxicus
Leifsonia xyli subsp. xyli...
Cryobacterium sp. MLB-32
Clavibacter michiganensis...
Curtobacterium flaccumfaci...
Rothia dentocariosa ATCC 1...
Renibacterium salmoninarum...
Tropheryma whipplei str. T...
Thermobispora bispora DSM...
Verrucosispora maris AB-18...
Acidothermus cellulolyticu...
Thermobifida fusca YX
Nocardioides sp. JS614
Mobiluncus curtisii ATCC 4...
Conexibacter woesei DSM 14...
Ilumatobacter coccineus YM...
Acidimicrobium ferrooxidan...
Slackia piriformis YIT 120...
Olsenella uli DSM 7084
Abiotrophia defectiva ATCC...
Lactococcus lactis subsp....
Lactococcus garvieae Lg2
Streptococcus pyogenes M1...
Oenococcus oeni PSU-1
Fructobacillus fructosus K...
Lactobacillus johnsonii NC...

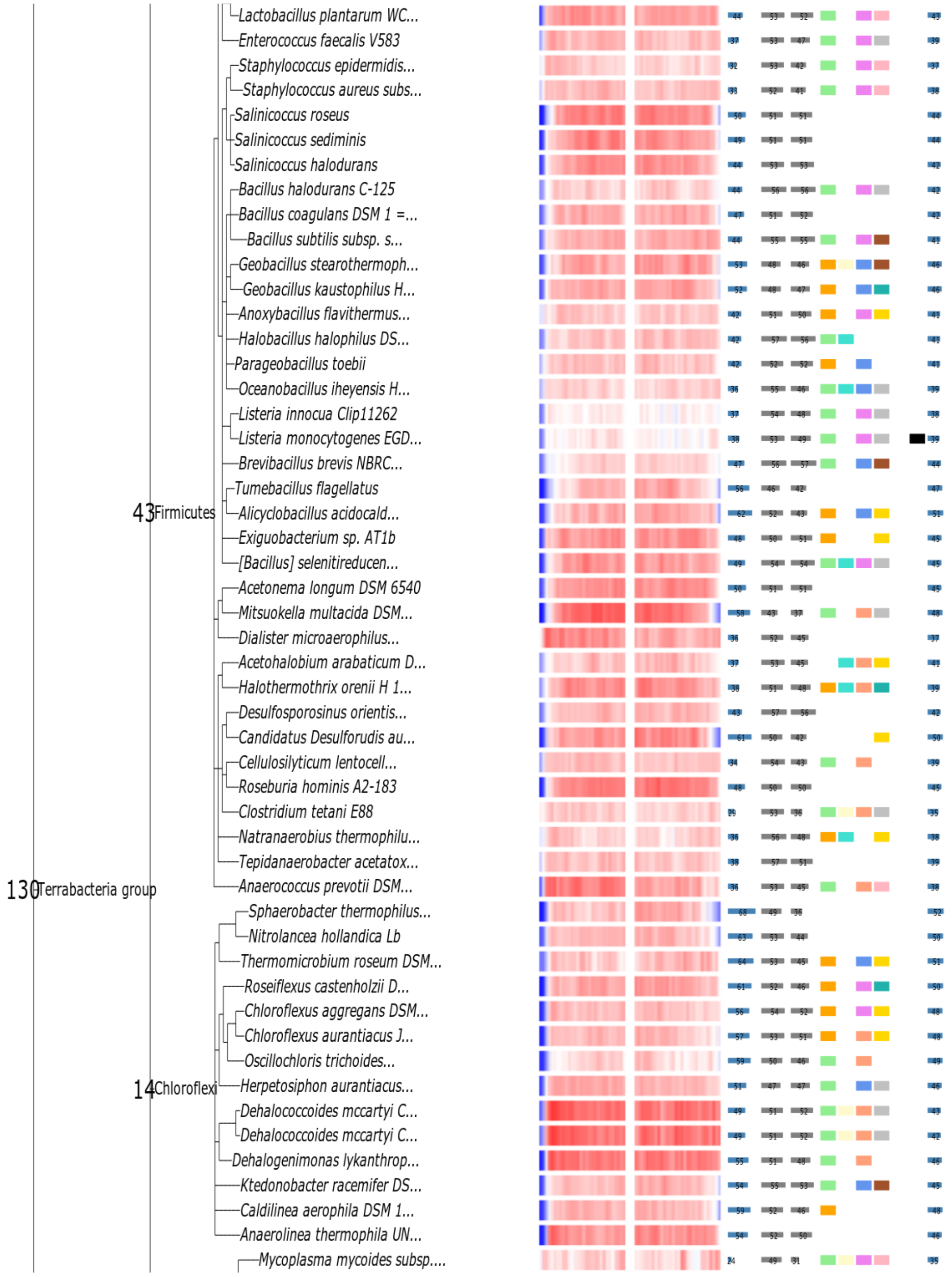

Lactobacillus plantarum WC...
Enterococcus faecalis V583
Staphylococcus epidermidis...
Staphylococcus aureus subs...
Salinicoccus roseus
Salinicoccus sediminis
Salinicoccus halodurans
Bacillus halodurans C-125
Bacillus coagulans DSM 1 =...
Bacillus subtilis subsp. s...
Geobacillus stearothermoph...
Geobacillus kaustophilus H...
Anoxybacillus flavithermus...
Halobacillus halophilus DS...
Parageobacillus toebii
Oceanobacillus iheyensis H...
Listeria innocua Clip11262
Listeria monocytogenes EGD...
Brevibacillus brevis NBRC...
Tumebacillus flagellatus
43 Firmicutes
Alicyclobacillus acidocald...
Exiguobacterium sp. AT1b
[Bacillus] selenitireducen...
Acetonema longum DSM 6540
Mitsuokella multacida DSM...
Dialister microaerophilus...
Acetohalobium arabaticum D...
Halothermothrix orenii H 1...
Desulfosporosinus orientis...
Candidatus Desulforudis au...
Cellulosilyticum lentocell...
Roseburia hominis A2-183
Clostridium tetani E88
Natranaerobius thermophilu...
Tepidanaerobacter acetatox...
130 Terrabacteria group
Anaerococcus prevotii DSM...
Sphaerobacter thermophilus...
Nitrolancea hollandica Lb
Thermomicrobium roseum DSM...
Roseiflexus castenholzii D...
Chloroflexus aggregans DSM...
Chloroflexus aurantiacus J...
Oscillochloris trichoides...
14 Chloroflexi
Herpetosiphon aurantiacus...
Dehalococcoides mccartyi C...
Dehalococcoides mccartyi C...
Dehalogenimonas lykanthrop...
Ktedonobacter racemifer DS...
Caldilinea aerophila DSM 1...
Anaerolinea thermophila UN...
Mycoplasma mycoides subsp....

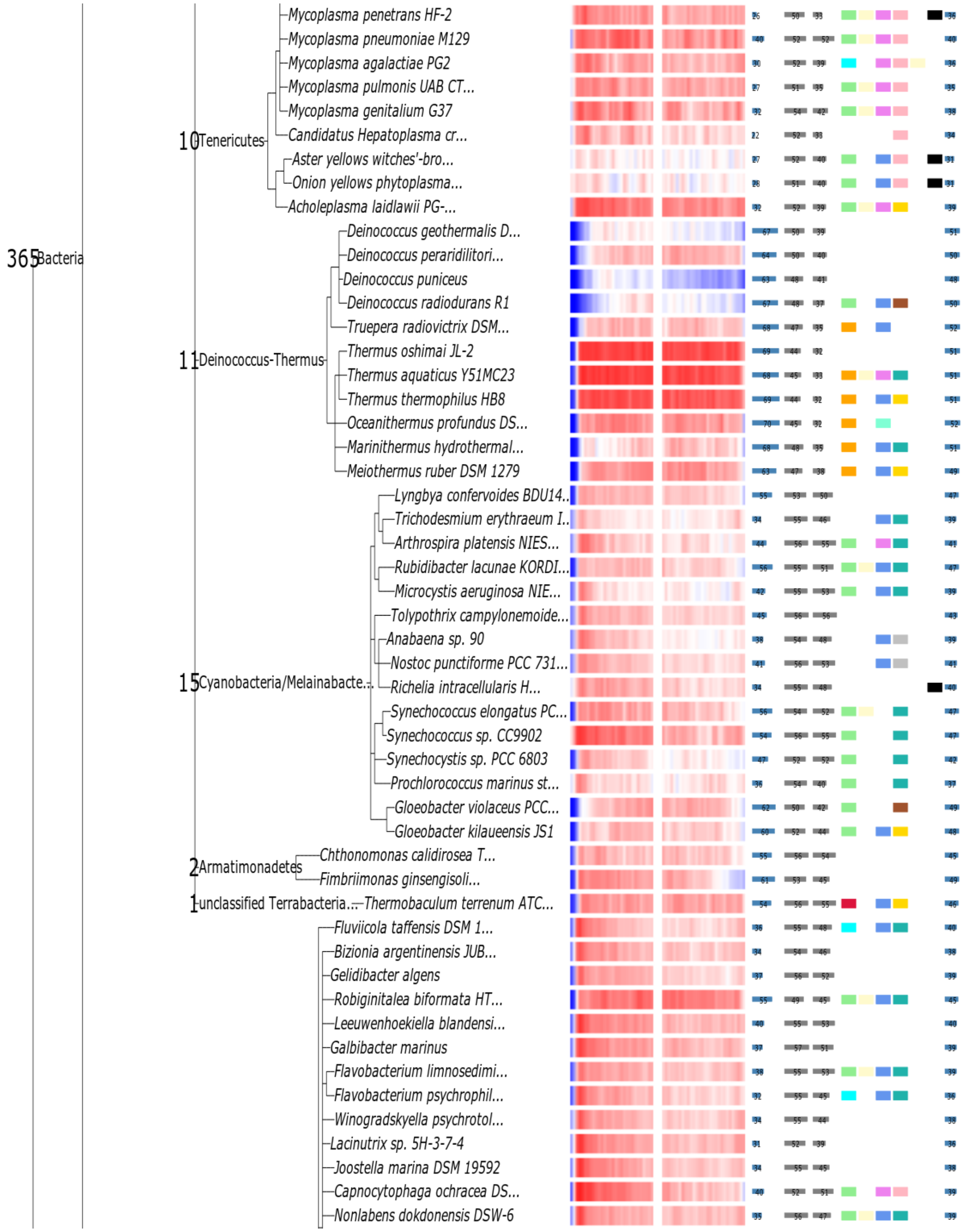
365 Bacteria
10 Tenericutes
Mycoplasma penetrans HF-2
Mycoplasma pneumoniae M129
Mycoplasma agalactiae PG2
Mycoplasma pulmonis UAB CT...
Mycoplasma genitalium G37
Candidatus Hepatoplasma cr...
Aster yellows witches'-bro...
Onion yellows phytoplasma...
Acholeplasma laidlawii PG-...
11 Deinococcus-Thermus
Deinococcus geothermalis D...
Deinococcus peraridilitori...
Deinococcus puniceus
Deinococcus radiodurans R1
Truepera radiovictrix DSM...
Thermus oshimai JL-2
Thermus aquaticus Y51MC23
Thermus thermophilus HB8
Oceanithermus profundus DS...
Marinithermus hydrothermal...
Meiothermus ruber DSM 1279
15 Cyanobacteria/Melainabacte...
Lyngbya confervoides BDU14...
Trichodesmium erythraeum I...
Arthrospira platensis NIES...
Rubidibacter lacunae KORDI...
Microcystis aeruginosa NIE...
Tolypothrix campylonemoide...
Anabaena sp. 90
Nostoc punctiforme PCC 731...
Richelia intracellularis H...
Synechococcus elongatus PC...
Synechococcus sp. CC9902
Synechocystis sp. PCC 6803
Prochlorococcus marinus st...
Gloeobacter violaceus PCC...
Gloeobacter kilaueensis JS1
2 Armatimonadetes
Chthonomonas calidirosea T...
Fimbriimonas ginsengisoli...
1 unclassified Terrabacteria...
Thermobaculum terrenum ATC...
Fluviicola taffensis DSM 1...
Bizionia argentinensis JUB...
Gelidibacter algens
Robiginitalea biformata HT...
Leeuwenhoekiella blandensi...
Galbibacter marinus
Flavobacterium limnosedimi...
Flavobacterium psychrophil...
Winogradskyella psychrotol...
Lacinutrix sp. 5H-3-7-4
Joostella marina DSM 19592
Capnocytophaga ochracea DS...
Nonlabens dokdonensis DSW-6

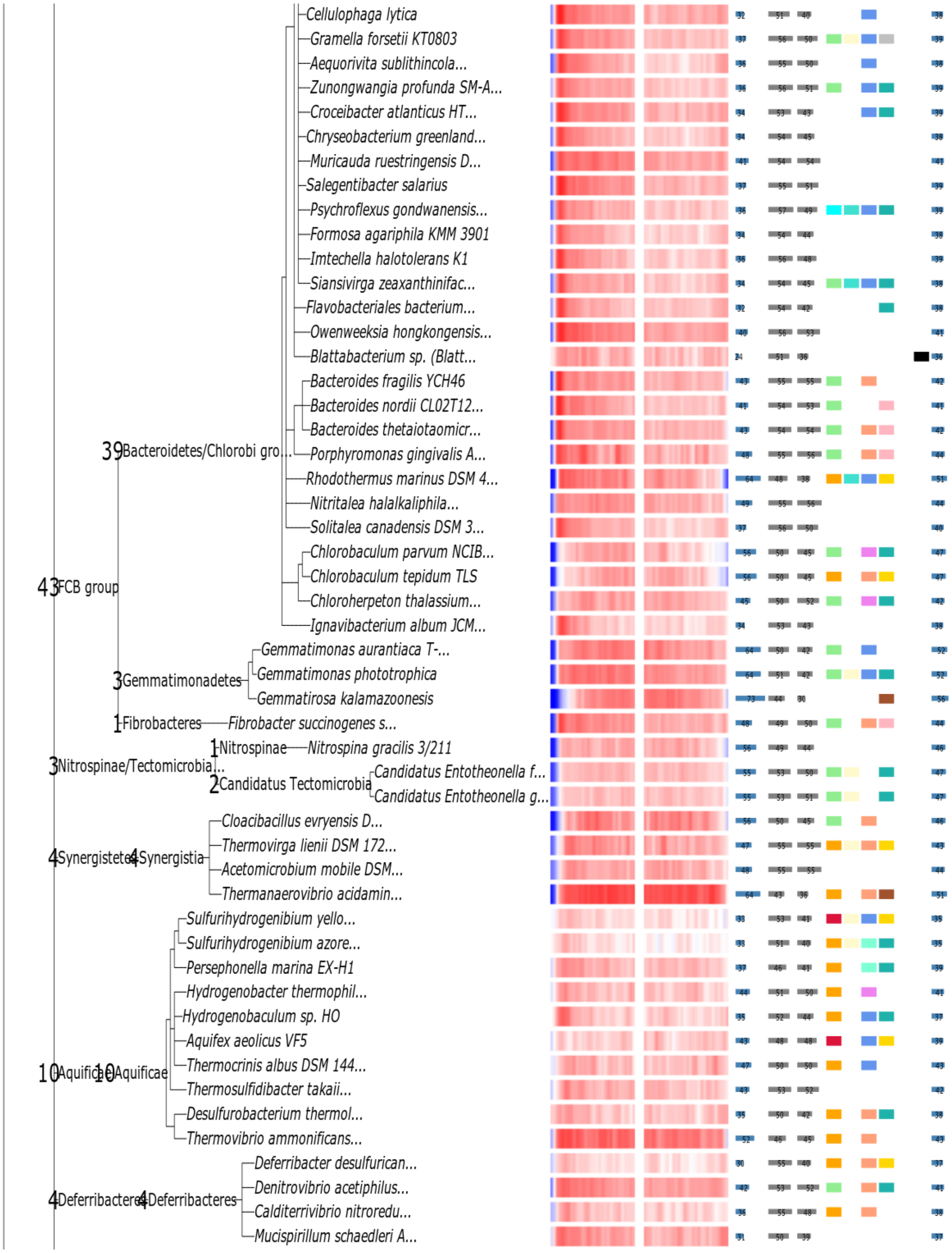
Cellulophaga lytica
Gramella forsetii KT0803
Aequorivita sublithincola...
Zunongwangia profunda SM-A...
Croceibacter atlanticus HT...
Chryseobacterium greenland...
Muricauda ruestringensis D...
Salegentibacter salarius
Psychroflexus gondwanensis...
Formosa agariphila KMM 3901
Imtechella halotolerans K1
Siansivirga zeaxanthinifac...
Flavobacteriales bacterium...
Owenweeksia hongkongensis...
Blattabacterium sp. (Blatt...
Bacteroides fragilis YCH46
Bacteroides nordii CL02T12...
Bacteroides thetaiotaomicr...
Porphyromonas gingivalis A...
39 Bacteroidetes/Chlorobi gro...
Rhodothermus marinus DSM 4...
Nitritalea halalkaliphila...
Solitalea canadensis DSM 3...
Chlorobaculum parvum NCIB...
Chlorobaculum tepidum TLS
43 FCB group
Chloroherpeton thalassium...
Ignavibacterium album JCM...
Gemmatimonas aurantiaca T-...
3 Gemmatimonadetes
Gemmatimonas phototrophica
Gemmatirosa kalamazoonesis
1 Fibrobacteres
Fibrobacter succinogenes s...
1 Nitrospinae
Nitrospina gracilis 3/211
3 Nitrospinae/Tectomicrobia...
Candidatus Entotheonella f...
2 Candidatus Tectomicrobia
Candidatus Entotheonella g...
Cloacibacillus evryensis D...
Thermovirga lienii DSM 172...
4 Synergistetes
4 Synergistia
Acetomicrobium mobile DSM...
Thermanaerovibrio acidamin...
Sulfurihydrogenibium yello...
Sulfurihydrogenibium azore...
Persephonella marina EX-H1
Hydrogenobacter thermophil...
Hydrogenobaculum sp. HO
Aquifex aeolicus VF5
10 Aquificae
10 Aquificae
Thermocrinis albus DSM 144...
Thermosulfidibacter takaii...
Desulfurobacterium thermol...
Thermovibrio ammonificans...
Deferribacter desulfurican...
Denitrovibrio acetiphilus...
4 Deferribacteres
4 Deferribacteres
Calditerrivibrio nitroredu...
Mucispirillum schaedleri A...

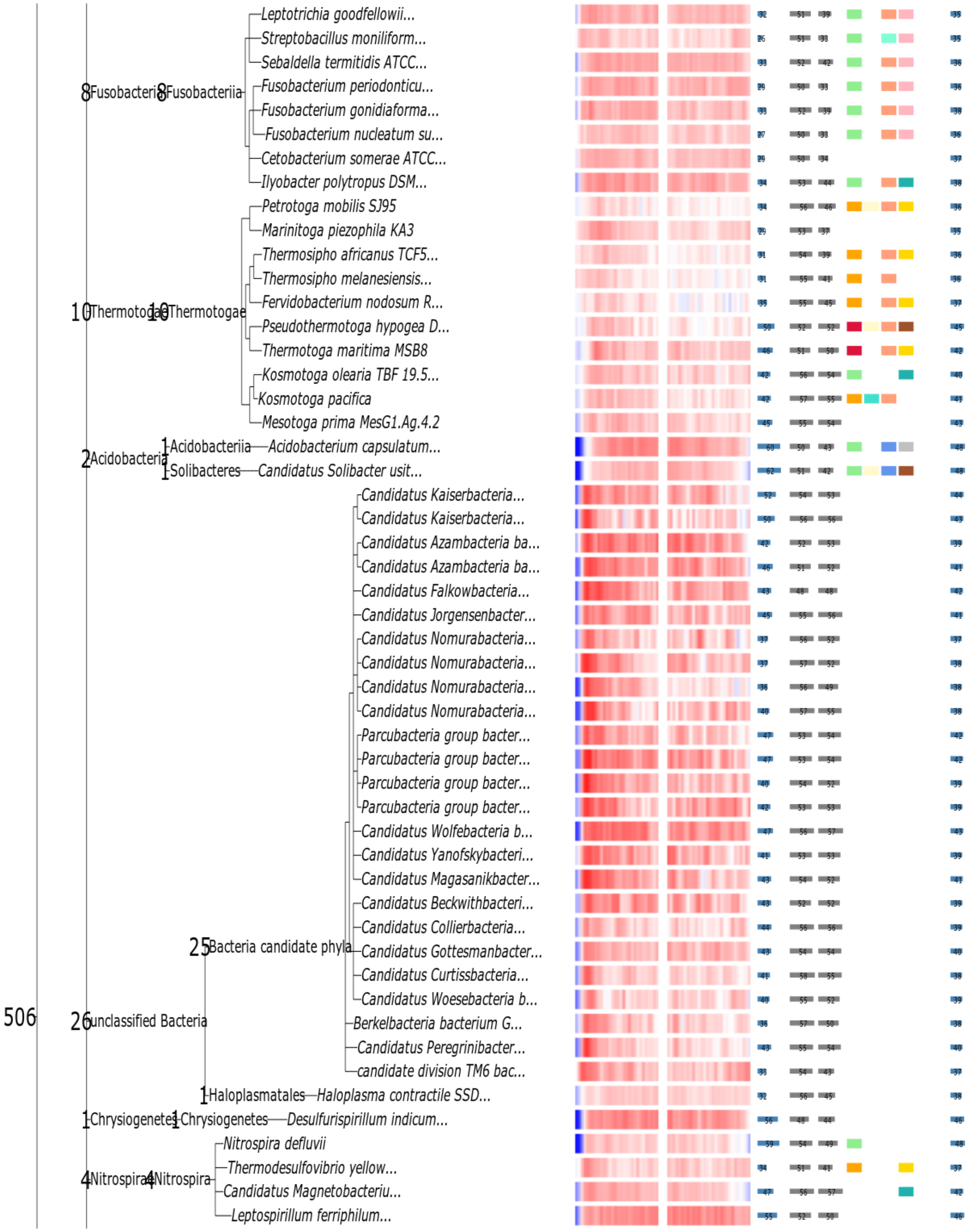
506
8 Fusobacteria 8 Fusobacteriia
Leptotrichia goodfellowii...
Streptobacillus moniliform...
Sebaldella termitidis ATCC...
Fusobacterium periodonticu...
Fusobacterium gonidiaforma...
Fusobacterium nucleatum su...
Cetobacterium somerae ATCC...
Ilyobacter polytropus DSM...
10 Thermotogae 10 Thermotogae
Petrotoga mobilis SJ95
Marinitoga piezophila KA3
Thermosipho africanus TCF5...
Thermosipho melanesiensis...
Fervidobacterium nodosum R...
Pseudothermotoga hypogea D...
Thermotoga maritima MSB8
Kosmotoga olearia TBF 19.5...
Kosmotoga pacifica
Mesotoga prima MesG1.Ag.4.2
2 Acidobacteria
1 Acidobacteriia Acidobacterium capsulatum...
1 Solibacteres Candidatus Solibacter usit...
26 unclassified Bacteria
25 Bacteria candidate phyla
Candidatus Kaiserbacteria...
Candidatus Kaiserbacteria...
Candidatus Azambacteria ba...
Candidatus Azambacteria ba...
Candidatus Falkowbacteria...
Candidatus Jorgensenbacter...
Candidatus Nomurabacteria...
Candidatus Nomurabacteria...
Candidatus Nomurabacteria...
Candidatus Nomurabacteria...
Parcubacteria group bacter...
Parcubacteria group bacter...
Parcubacteria group bacter...
Parcubacteria group bacter...
Candidatus Wolfebacteria b...
Candidatus Yanofskybacteri...
Candidatus Magasanikbacter...
Candidatus Beckwithbacteri...
Candidatus Collierbacteria...
Candidatus Gottesmanbacter...
Candidatus Curtissbacteria...
Candidatus Woesebacteria b...
Berkelbacteria bacterium G...
Candidatus Peregrinibacter...
candidate division TM6 bac...
1 Haloplasmatales Haloplasma contractile SSD...
1 Chrysiogenetes 1 Chrysiogenetes Desulfurispirillum indicum...
4 Nitrospira 4 Nitrospira
Nitrospira defluvii
Thermodesulfovibrio yellow...
Candidatus Magnetobacteriu...
Leptospirillum ferriphilum...

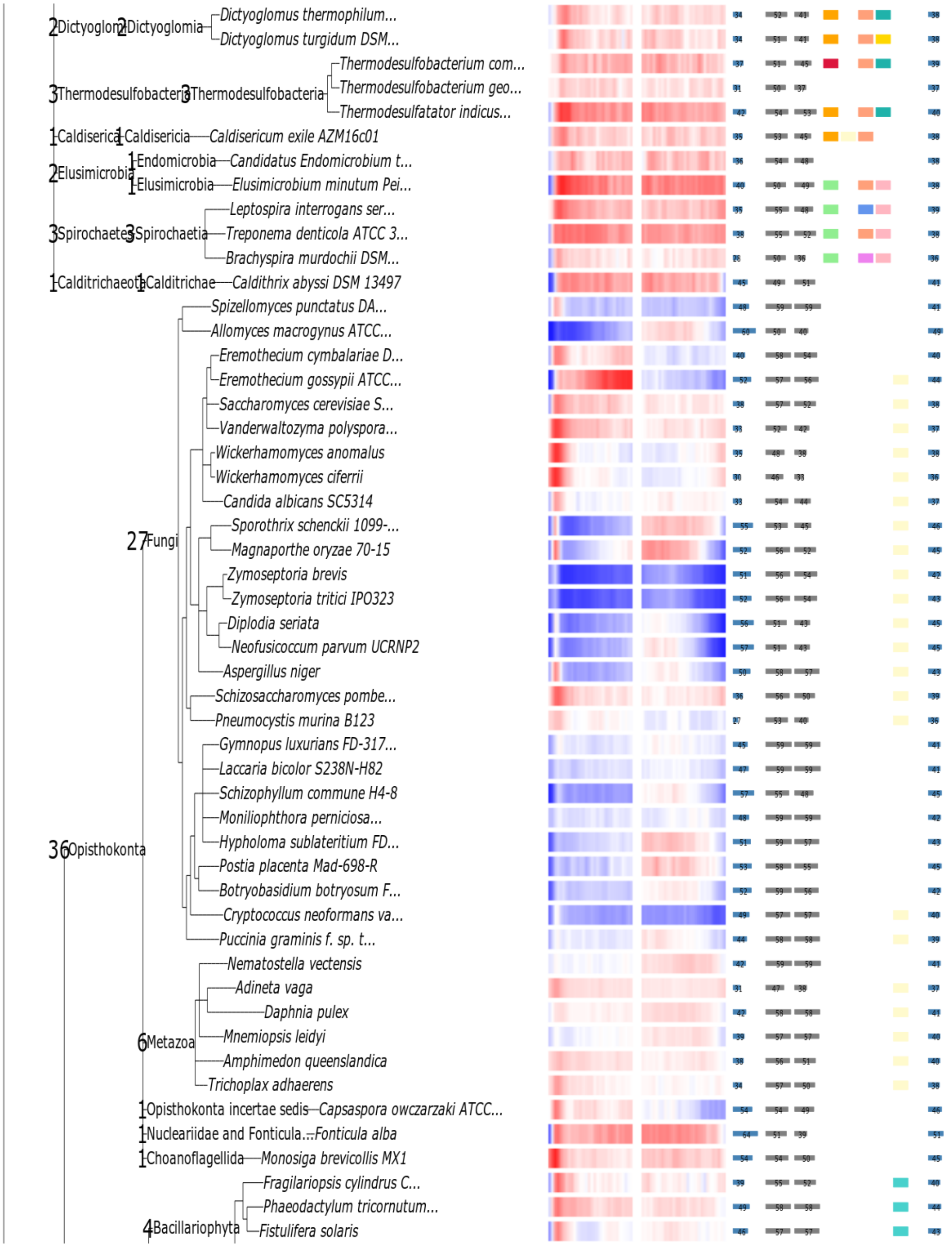
2 Dictyoglomi
2 Dictyoglomia
Dictyoglomus thermophilum...
Dictyoglomus turgidum DSM...
3 Thermodesulfobacteria
3 Thermodesulfobacteria
Thermodesulfobacterium com...
Thermodesulfobacterium geo...
Thermodesulfatator indicus...
1 Caldiserica
1 Caldisericia
Caldisericum exile AZM16c01
2 Elusimicrobia
1 Endomicrobia
Candidatus Endomicrobium t...
1 Elusimicrobia
Elusimicrobium minutum Pei...
3 Spirochaetes
3 Spirochaetia
Leptospira interrogans ser...
Treponema denticola ATCC 3...
Brachyspira murdochii DSM...
1 Calditrichaeota
1 Calditrichae
Caldithrix abyssi DSM 13497
36 Opisthokonta
27 Fungi
Spizellomyces punctatus DA...
Allomyces macrogynus ATCC...
Eremothecium cymbalariae D...
Eremothecium gossypii ATCC...
Saccharomyces cerevisiae S...
Vanderwaltozyma polyspora...
Wickerhamomyces anomalus
Wickerhamomyces ciferrii
Candida albicans SC5314
Sporothrix schenckii 1099-...
Magnaporthe oryzae 70-15
Zymoseptoria brevis
Zymoseptoria tritici IPO323
Diplodia seriata
Neofusicoccum parvum UCRNP2
Aspergillus niger
Schizosaccharomyces pombe...
Pneumocystis murina B123
Gymnopus luxurians FD-317...
Laccaria bicolor S238N-H82
Schizophyllum commune H4-8
Moniliophthora perniciosa...
Hypholoma sublateritium FD...
Postia placenta Mad-698-R
Botryobasidium botryosum F...
Cryptococcus neoformans va...
Puccinia graminis f. sp. t...
6 Metazoa
Nematostella vectensis
Adineta vaga
Daphnia pulex
Mnemiopsis leidyi
Amphimedon queenslandica
Trichoplax adhaerens
1 Opisthokonta incertae sedis
Capsaspora owczarzaki ATCC...
1 Nucleariidae and Fonticula...
Fonticula alba
1 Choanoflagellida
Monosiga brevicollis MX1
4 Bacillariophyta
Fragilariopsis cylindrus C...
Phaeodactylum tricornutum...
Fistulifera solaris

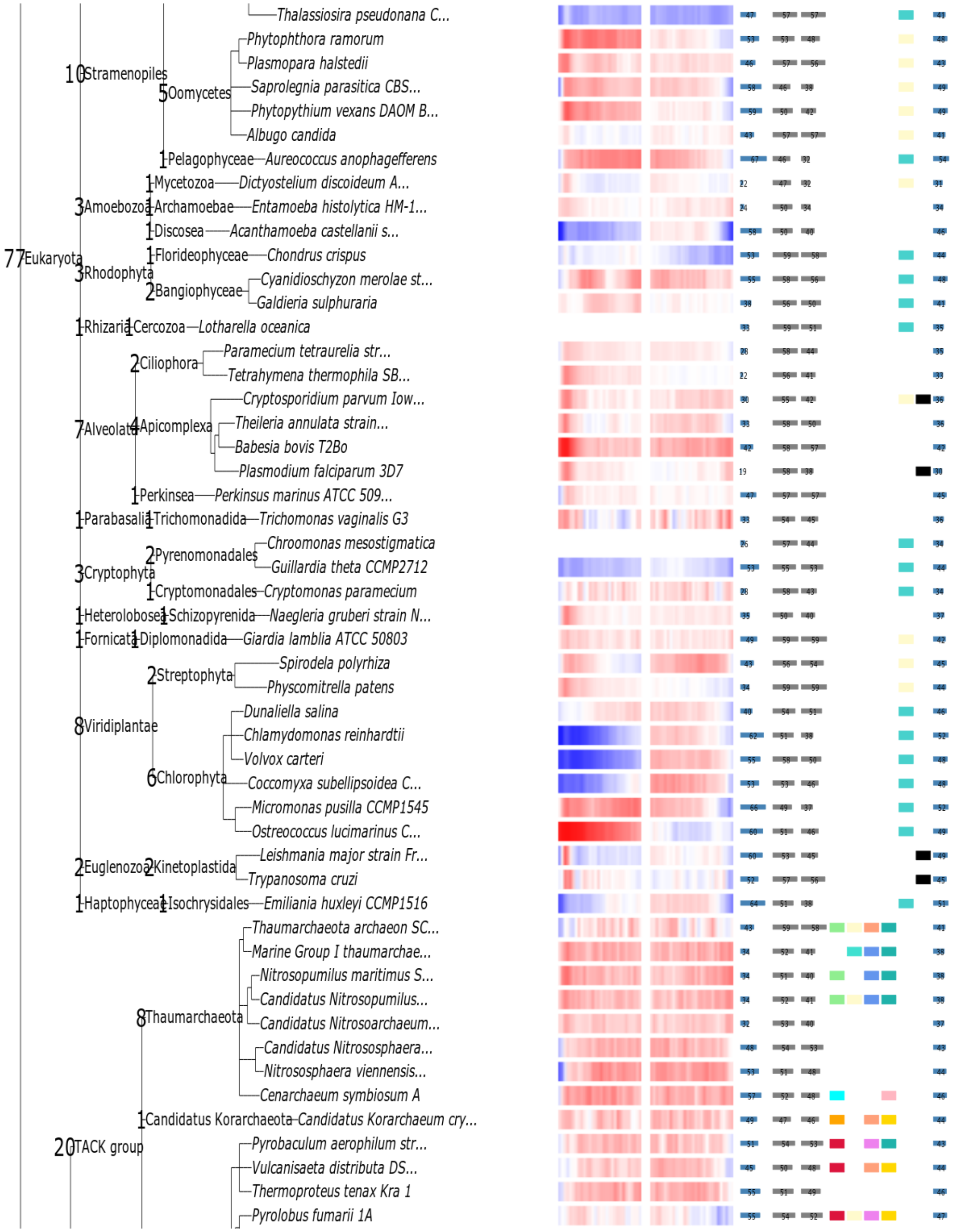

77 Eukaryota
10 Stramenopiles
Thalassiosira pseudonana C...
5 Oomycetes
Phytophthora ramorum
Plasmopara halstedii
Saprolegnia parasitica CBS...
Phytopythium vexans DAOM B...
Albugo candida
1 Pelagophyceae
Aureococcus anophagefferens
3 Amoebozoa
1 Mycetozoa
Dictyostelium discoideum A...
1 Archamoebae
Entamoeba histolytica HM-1...
1 Discosea
Acanthamoeba castellanii s...
3 Rhodophyta
1 Florideophyceae
Chondrus crispus
2 Bangiophyceae
Cyanidioschyzon merolae st...
Galdieria sulphuraria
1 Rhizaria
1 Cercozoa
Lotharella oceanica
7 Alveolata
2 Ciliophora
Paramecium tetraurelia str...
Tetrahymena thermophila SB...
4 Apicomplexa
Cryptosporidium parvum Iow...
Theileria annulata strain...
Babesia bovis T2Bo
Plasmodium falciparum 3D7
1 Perkinsea
Perkinsus marinus ATCC 509...
1 Parabasalia
1 Trichomonadida
Trichomonas vaginalis G3
3 Cryptophyta
2 Pyrenomonadales
Chroomonas mesostigmatica
Guillardia theta CCMP2712
1 Cryptomonadales
Cryptomonas paramecium
1 Heterolobosea
1 Schizopyrenida
Naegleria gruberi strain N...
1 Fornicata
1 Diplomonadida
Giardia lamblia ATCC 50803
8 Viridiplantae
2 Streptophyta
Spirodela polyrhiza
Physcomitrella patens
6 Chlorophyta
Dunaliella salina
Chlamydomonas reinhardtii
Volvox carteri
Coccomyxa subellipsoidea C...
Micromonas pusilla CCMP1545
Ostreococcus lucimarinus C...
2 Euglenozoa
2 Kinetoplastida
Leishmania major strain Fr...
Trypanosoma cruzi
1 Haptophyceae
1 Isochrysidales
Emiliania huxleyi CCMP1516
20 TACK group
8 Thaumarchaeota
Thaumarchaeota archaeon SC...
Marine Group I thaumarchae...
Nitrosopumilus maritimus S...
Candidatus Nitrosopumilus...
Candidatus Nitrosoarchaeum...
Candidatus Nitrososphaera...
Nitrososphaera viennensis...
Cenarchaeum symbiosum A
1 Candidatus Korarchaeota
Candidatus Korarchaeum cry...
Pyrobaculum aerophilum str...
Vulcanisaeta distributa DS...
Thermoproteus tenax Kra 1
Pyrolobus fumarii 1A

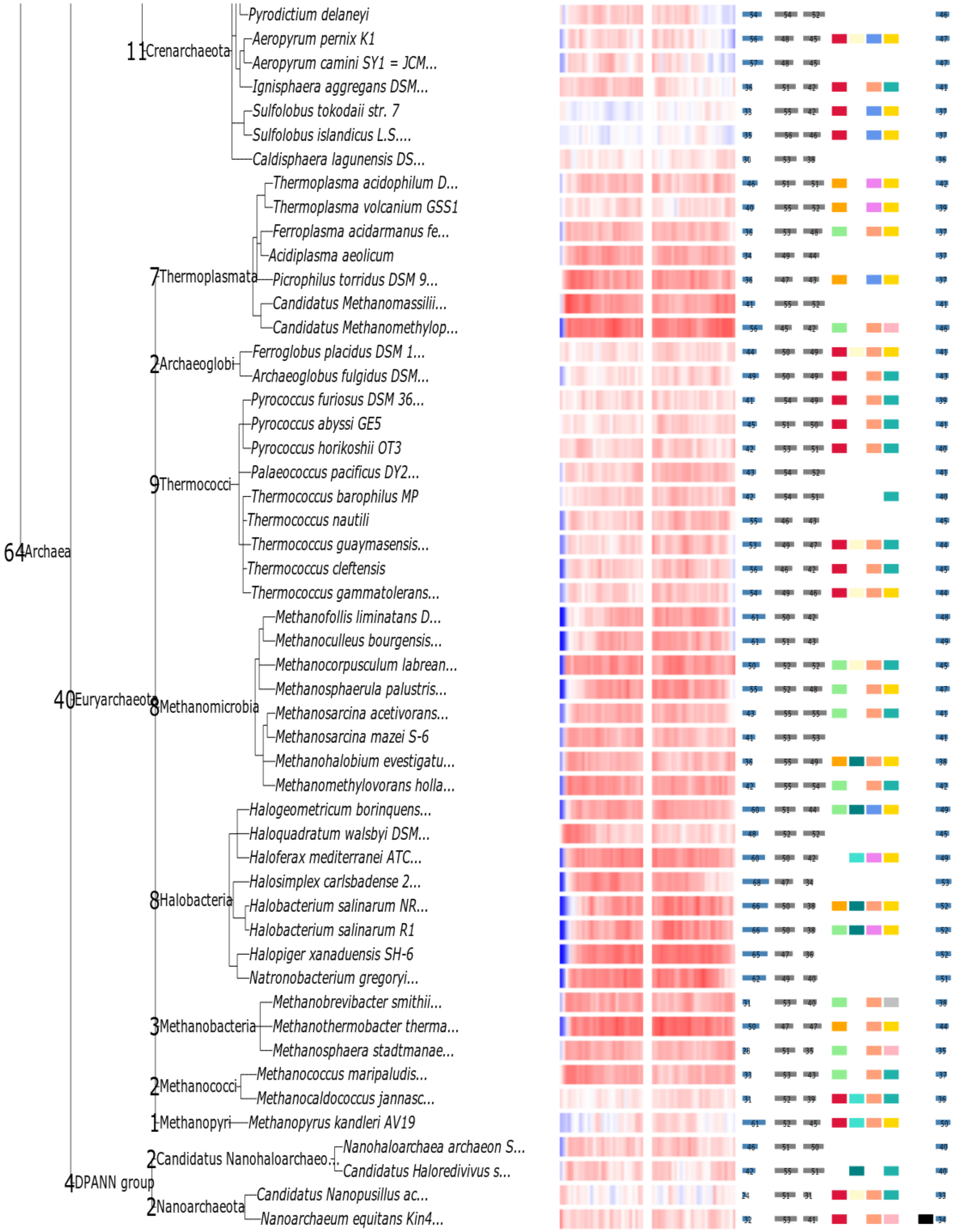

ΔLFE profiles calculated using the CDS-wide randomization for individual species arranged by NCBI taxonomy. The ΔLFE profiles shown are for positions 0-300nt relative to CDS start (left) and CDS end (right). The numbers of species included in each group is shown to the left of the group name.

Figure S8. Comparison between ΔLFE profiles in different domains

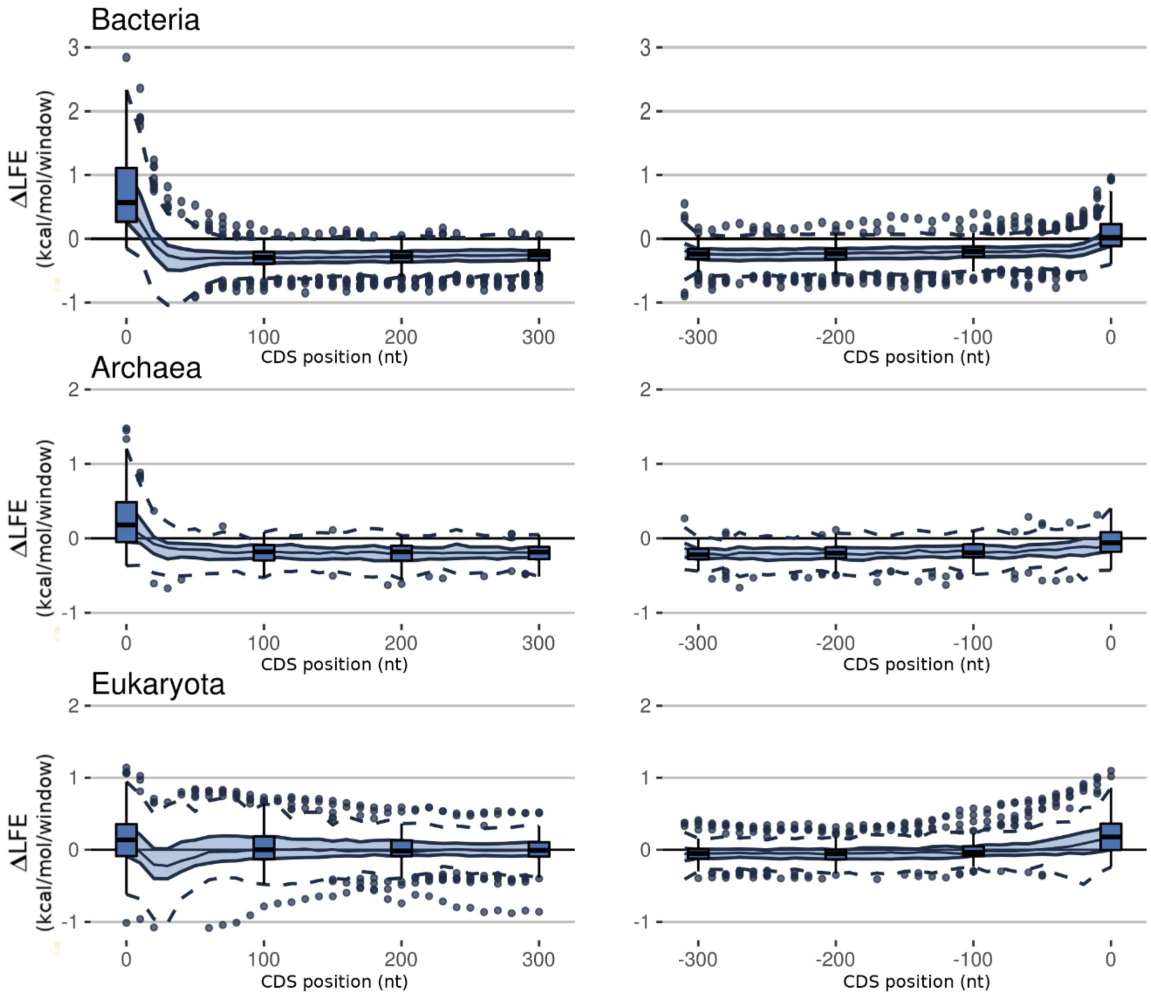

Distribution of ΔLFE profiles relative to CDS start (left) and end (right), for species belonging to each domain. In bacteria and archaea, only one species has positive ΔLFE in the mid-CDS region, despite this being common in eukaryotes.

Figure S9. Autocorrelation between ΔLFE profile regions

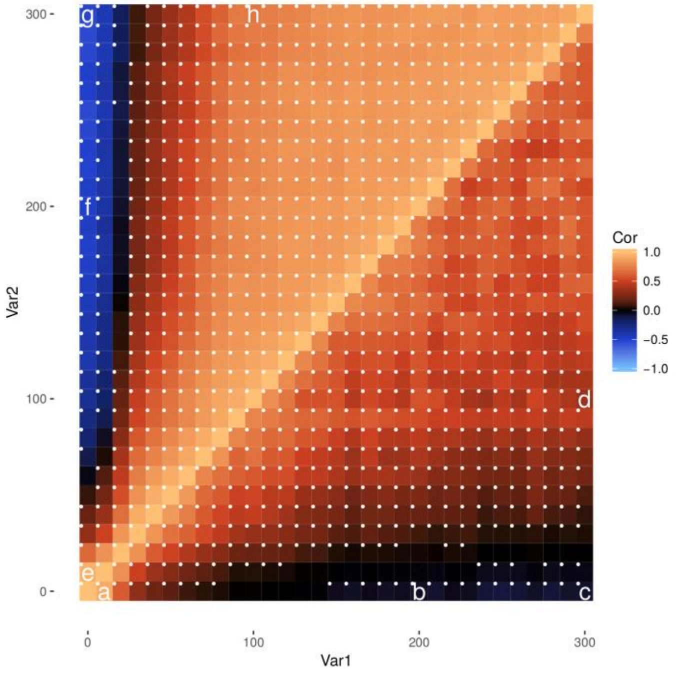

A

B

Pos1 (nt)	Pos2 (nt)	Label	Correlation	p−val
10	0	a	0.918	0.0e+00
200	0	b	−0.054	1.8e−05
300	0	c	−0.071	1.0e−06
300	100	d	0.346	0.0e+00
0	10	e	0.933	0
0	200	f	−0.494	0
0	300	g	−0.520	0
100	300	h	0.705	0

A Autocorrelation for ΔLFE between positions relative to CDS start. Above main diagonal - Pearson's correation. Below main diagonal - coefficient of determination (R^2) for GLS regression. Values for positions a-h indicated on **B**. Significant positions (*p*-value<0.01) indicated by white dots. **B** Numerical values (a-d - R^2, e-h - Pearson's-*r*) and *p*-values for positions marked in **A**.
This supports the robustness of the values in Fig. 3E.

Figure S10. Trait correlations in taxonomic subgroups

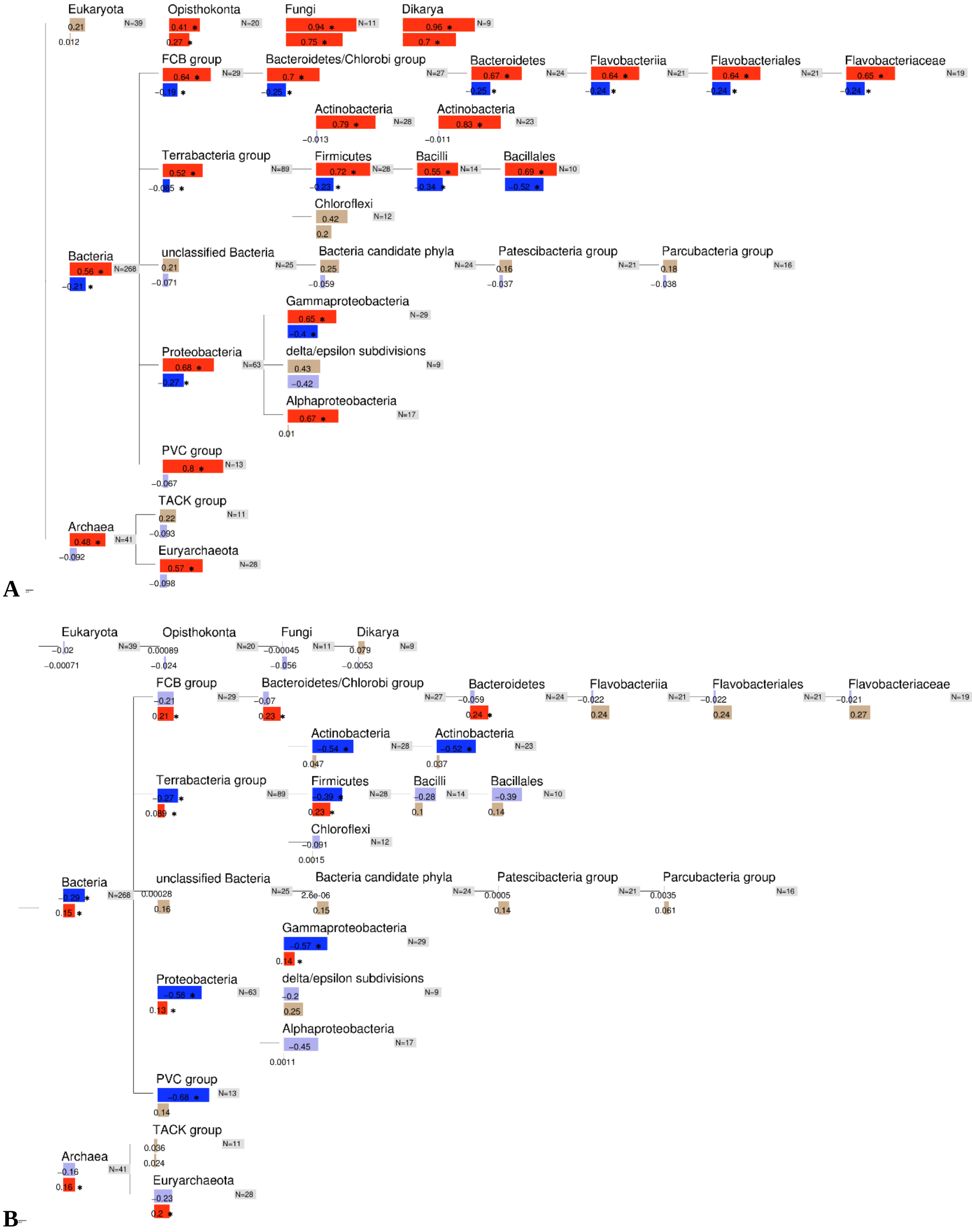

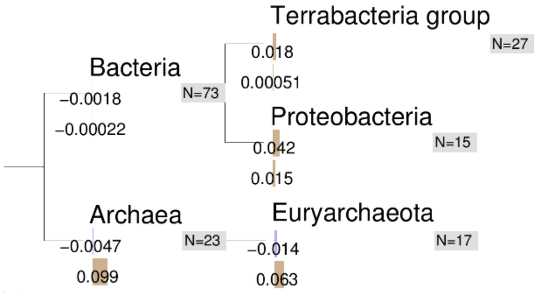

C

Coefficient of determination (R^2) and regression direction for GLS regression between genomic-GC% and mean ΔLFE in different taxonomic subgroups, for two regions relative to CDS-start. Top bar. 0-20nt; Bottom bar, 70-300nt. Sign of regression slope is indicated by color - Red - positive (reinforcing) effect; Blue - negative (compensating) effect. Significant results (FDR, *p*-value<0.01) are indicated by color intensity and marked with a '∗'. Included taxonomic groups have 9 or more species in the dataset.

A Genomic GC. **B** Genomic ENc'. **C** Optimum Temperature.

Figure S11. Correlation of ΔLFE with different genomic measures of CUB is consistent

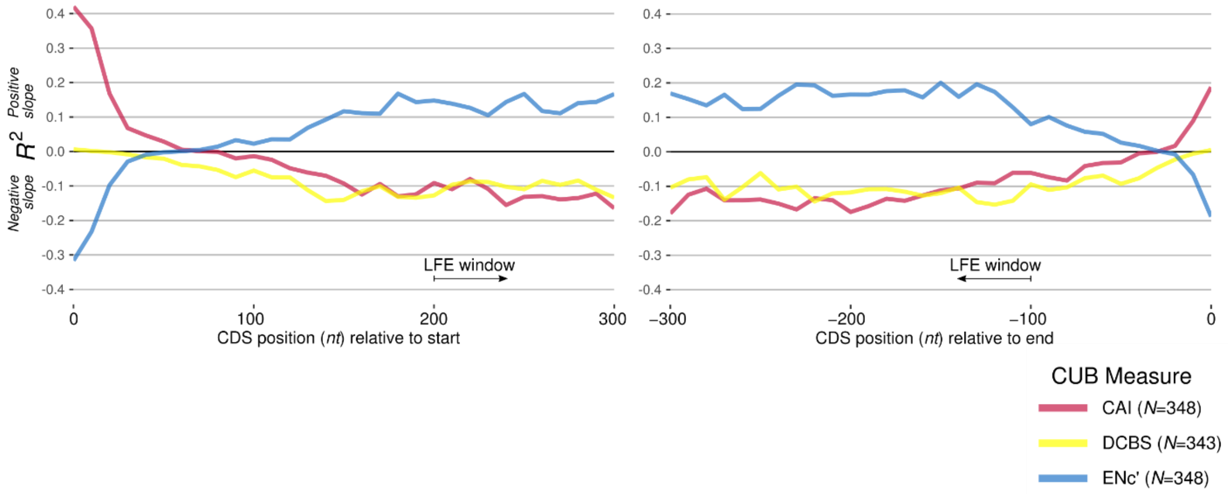

Using different measures of CUB generally leads to the same conclusion about the interaction between CUB and ΔLFE. Note that for CAI and DCBS, increasing values indicate stronger bias, whereas for ENc', decreasing values indicate stronger bias.

The following measures were used to estimate genomic CUB. CAI (66) was computed using codonw(67) version 1.4.4, using the entire genome as the reference set. ENc' (68) was calculated using *ENCprime* (github user jnovembre, commit 0ead568, Oct. 2016). DCBS(69) was calculated as described in the paper. All CUB measures were averaged for each genome and the resulting values were used in GLS regression against the ΔLFE at each position.

Figure S12. Genomic-ENc’ correlates with ΔLFE magnitude, not shape

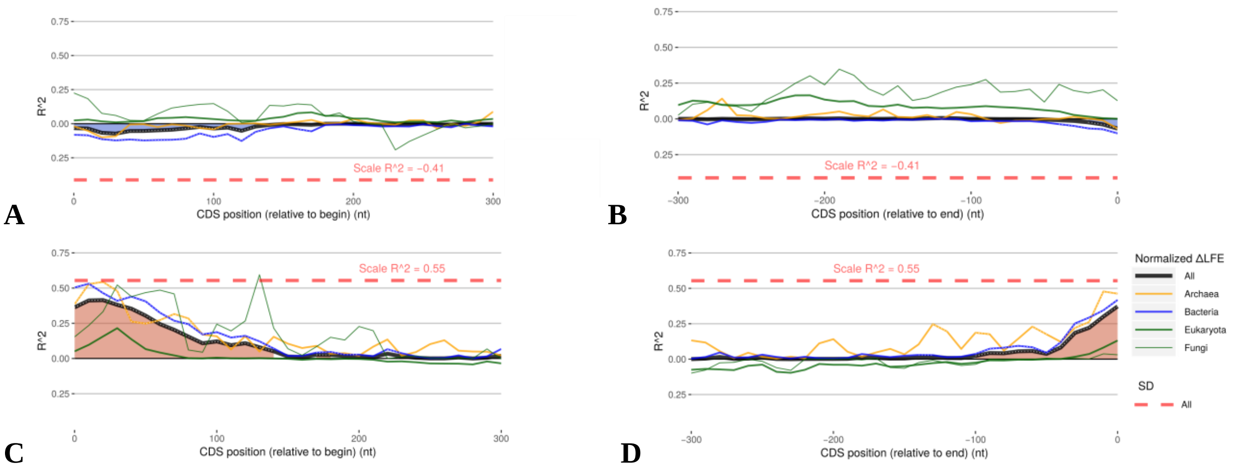

To test if correlation between genomic-ENc′ and ΔLFE is related to the general magnitude of ΔLFE or to position-specific aspects of the ΔLFE profile, we performed the following test: we decomposed the values by normalizing each genomic profile by its standard-deviation (as a measure of its scale), thus getting profiles of equal scale. We then checked for correlation between the normalized ΔLFE profiles with genomic-ENc′. There was no correlation after this normalization (Fig. S9), but the correlation between genomic-ENc′ and the scaling factor was strong. This suggests that the correlation of ENc′ (in contrast to GC-content) is indeed caused by the magnitude of ΔLFE.

The observed correlation of ΔLFE with Genomic-ENc’ (Fig. 6) is due to correlation with the magnitude of the ΔLFE profile. When all profiles are normalized to have the same scale (by dividing the values of each profile by their standard deviation so the resulting profiles all have standard deviation 1), most of the correlation is removed (**A**,**B**). For comparison, the same procedure is followed for genomic-GC (**C**,**D**). Values represent coefficient of determination (R^2) for GLS regression of each trait (genomic-ENc’ or genomic-GC%) vs. the normalized ΔLFE profile at different position relative to CDS edges, with the sign representing the regression coefficient. Regressions for different taxons are shown using different line colors and widths (black is for all species), and white dots show areas in which the regression is significant (*p*-value<0.01). The dashed red line represents R^2 for regression against the standard deviation for each ΔLFE profile (i.e., the scaling factor). **A** Genomic-ENc’ vs. ΔLFE, CDS start. **B** Genomic-ENc’ vs. ΔLFE, CDS end. **C** Genomic-GC vs. ΔLFE, CDS start. **D** Genomic-GC vs. ΔLFE, CDS end.

Figure S13. Genomic-GC and genomic-ENc' both predict ΔLFE

A

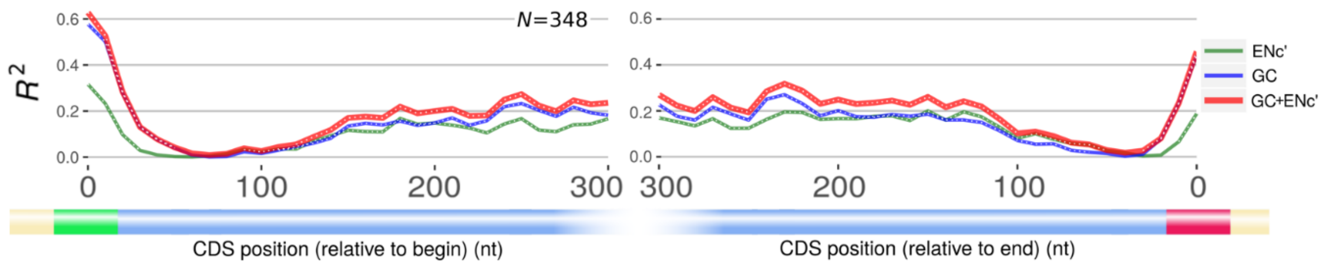

B

CDS Reference	Positions (nt)	*p*-value (GC)	*p*-value (ENc')	R^2 (GLS)	*N*	Group
**Start**	0-20	**(*)** 1.65E-38	**(*)** 5.51E-06	0.534	348	All species
**Start**	150-300	**(*)** 2.40E-10	**(*)** 5.59E-06	0.259	348	All species
**End**	150-300	**(*)** 3.62E-12	**(*)** 9.65E-08	0.310	348	All species
**End**	0-20	**(*)** 5.04E-20	2.72E-01	0.287	348	All species

A. *Comparison of* R^2 *values for GLS regression using genomic-GC (blue), genomic-ENc' (green), and both factors (red). Significance of the regression slope (determined using* t-*test) is indicated by white dots. Genomic-GC and genomic-ENc' have similar explanatory power in the mid-CDS region, but they explain somewhat different parts of the variation, so adding the second factor improved the regression fit and the slope of the second factor (in this case, ENc') is significant in most position within the CDS.*

B. *Numeric regression results for multiple regression using genomic-GC and genomic-ENc' in 4 regions of the CDS shows slopes for both factors are significant in most regions. This indicates each factor improves upon the prediction of the other factor. Significance is determined using* t-*test.*

*Explanation of table columns: **CDS Reference** – point in CDS (start/end) for defining relative positions within all CDSs. **Positions**: range of positions within CDS (relative to the reference) for which ΔLFE values are averaged.* **p-*value (GC)***: p-*value (using* t-*test) for Genomic-GC factor, in multiple regression (including factors GenmoicGC, GenomicENc') using GLS.* **p-*value (ENc')***: p-*value (using* t-*test) for Genomic-ENc' factor, in multiple regression (including factors GenmoicGC, GenomicENc') using GLS.* **R^2 *(GLS)***: *coefficient of determination* (R^2) *for regression using the factors GenmoicGC+GenomicENc'.* **N**: *number of species included in GLS regression. **Group**: taxonomic group for this analysis.*

Figure S14. Endosymbionts have weaker ΔLFE

**CDS Reference**	**Positions (nt)**	***OLS Is-intracellular***	***GLS GenmoicGC + GenomicENc' + Is-intracellular***					**Group**
		p-value (is Intracellular)	*p*-value (is Intracellular)	R^2 (without Intracellular)	R^2 (with Intracellular)	*Slope*	*N*	
**Start**	0-20	**(*)** 3.01E-03	1.54E-01	0.534	0.537	-	348	All species
**Start**	150-300	**(*)** 3.01E-03	2.01E-01	0.259	0.263	+	348	All species
**End**	150-300	1.80E-01	1.16E-01	0.310	0.315	+	348	All species
**End**	0-20	**(*)** 1.05E-02	**(*)** 5.51E-03	0.287	0.303	-	348	All species
**Start**	0-20	**(*)** 1.27E-03	4.19E-01	0.596	0.597	-	268	Bacteria
**Start**	150-300	1.61E-01	2.06E-01	0.351	0.355	+	268	Bacteria
**End**	150-300	1.22E-01	9.36E-02	0.306	0.314	+	268	Bacteria
**End**	0-20	**(*)** 3.00E-03	**(*)** 8.45E-03	0.291	0.309	-	268	Bacteria
**Start**	0-20	**(*)** 1.32E-03	1.25E-01	0.751	0.761	-	63	Proteobacteria
**Start**	150-300	**(*)** 4.42E-03	**(*)** 3.89E-02	0.409	0.450	+	63	Proteobacteria
**End**	150-300	**(*)** 1.44E-02	**(*)** 4.65E-02	0.331	0.375	+	63	Proteobacteria
**End**	0-20	**(*)** 2.37E-02	**(*)** 1.11E-02	0.222	0.303	-	63	Proteobacteria
**Start**	0-20	**(*)** 1.50E-02	2.80E-01	0.748	0.760	-	29	Gammaproteobacteria
**Start**	150-300	**(*)** 8.65E-04	9.82E-02	0.612	0.653	+	29	Gammaproteobacteria
**End**	150-300	**(*)** 4.23E-03	1.85E-01	0.418	0.458	+	29	Gammaproteobacteria
**End**	0-20	5.58E-01	**(*)** 2.60E-02	0.164	0.317	-	29	Gammaproteobacteria
**Start**	0-20	**(*)** 1.45E-02	5.52E-01	0.569	0.571	-	89	Terrabacteria group
**Start**	150-300	3.57E-01	5.72E-01	0.177	0.180	+	89	Terrabacteria group
**End**	150-300	7.19E-02	2.67E-01	0.129	0.142	+	89	Terrabacteria group
**End**	0-20	5.11E-01	1.71E-01	0.295	0.310	-	89	Terrabacteria group

Numeric regression results for GLS multiple regression using genomic-GC, genomic-ENc' and intracellular classification in 4 regions of the CDS, for several taxonomic groups (which contain a sufficient number of intracellular species). p-*values shown for GLS are for the categorical Is-intracellular classification factor (determined using* t-*test), indicating this factor improves upon the predictions made using the two numerical factors in some cases (even after controlling for evolutionary relatedness using GLS), but not in others.* R^2 *values are shown for the regression without and with intracellular classification.*

Explanation of table columns: ***CDS Reference*** *– point in CDS (start/end) for defining relative positions within all CDSs.* ***Positions****: range of positions within CDS (relative to the reference) for which ΔLFE values are averaged.* ***OLS*** **p*****-value****:* p-*value (using* t-*test) for Is-intracellular factor, in single regression using OLS (uncorrected for phylogenetic distances). This regression includes all available species (including those which are not contained in the phylogenetic tree so are not used in GLS regression).* ***GLS*** **p*****-value****:* p-*value (using* t-*test) for Is-intracellular factor, in multiple regression (including factors GenmoicGC, GenomicENc') using GLS.* $\mathbf{R^2}$ ***without Is-intracellular****: coefficient of determination* (R^2) *for regression using the factors GenmoicGC+GenomicENc', as baseline for comparing improvement from the additional factor Is-intracellular.* $\mathbf{R^2}$ ***with Is-intracellular****: coefficient of determination* (R^2) *for regression using the factors GenmoicGC+GenomicENc'+Is-intracellular.* ***Slope****: direction of slope for factor Is-intracellular (positive or negative). This indicates intracellular species have weaker ΔLFE in*

the ranges shown. **N***: number of species included in GLS regression.* ***Group****: taxonomic group for this analysis.*

Figure S15. Range robustness for GLS regressions between ΔLFE and related traits

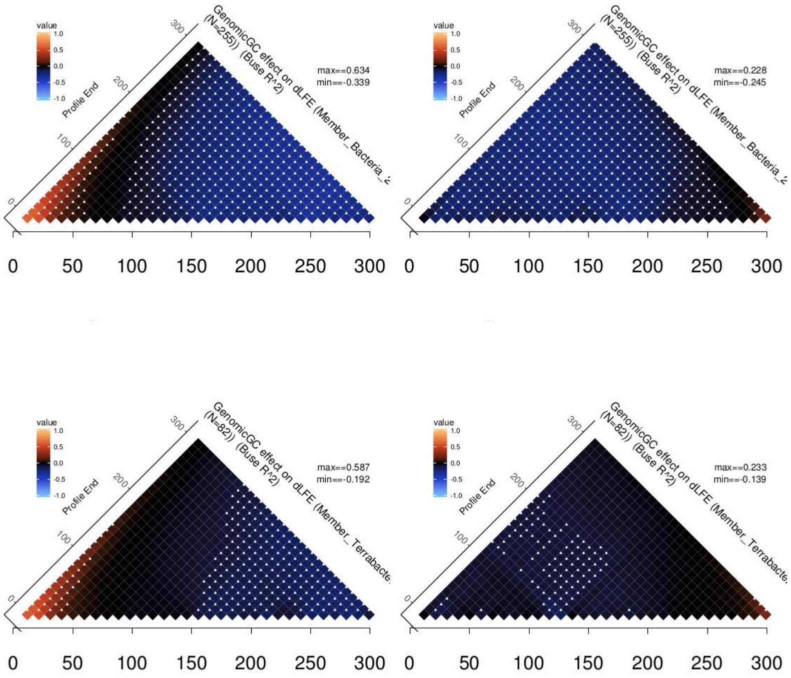

A

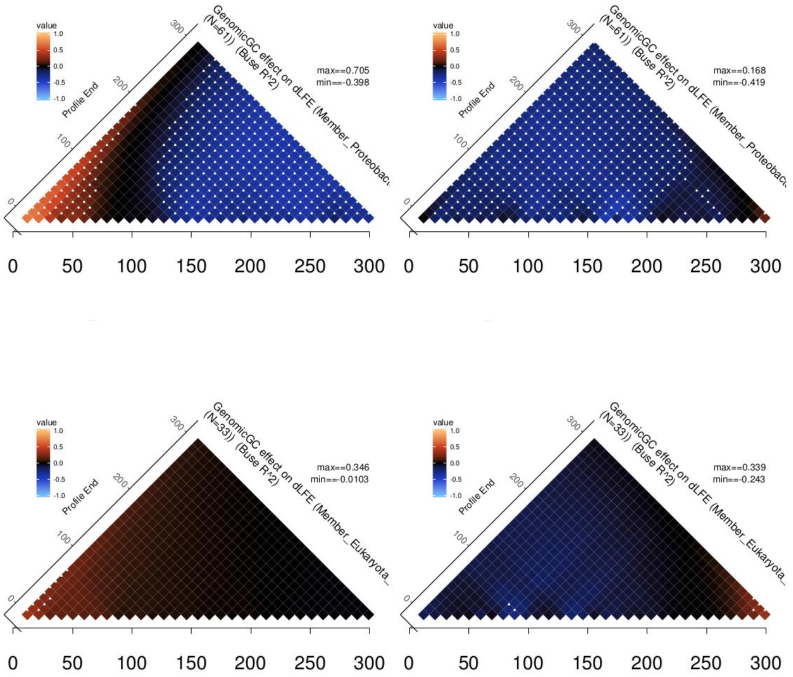

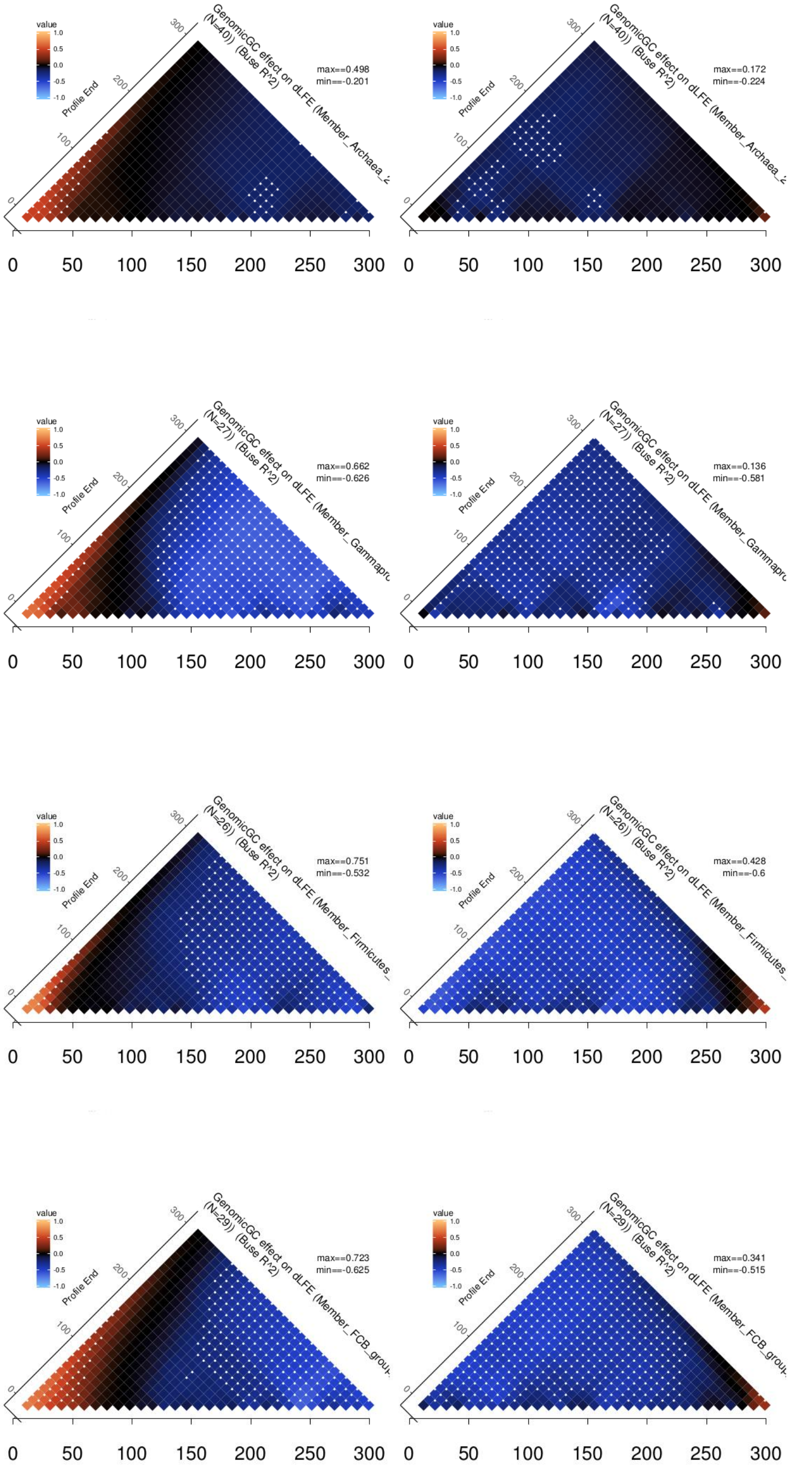
GenomicGC effect on dLFE (Member_Archaea_2
(N=40)) (Buse R^2)
max==0.498
min==-0.201
max==0.172
min==-0.224
GenomicGC effect on dLFE (Member_Gammapro
(N=27)) (Buse R^2)
max==0.662
min==-0.626
max==0.136
min==-0.581
GenomicGC effect on dLFE (Member_Firmicutes_
(N=26)) (Buse R^2)
max==0.751
min==-0.532
max==0.428
min==-0.6
GenomicGC effect on dLFE (Member_FCB_group
(N=29)) (Buse R^2)
max==0.723
min==-0.625
max==0.341
min==-0.515
value
Profile End
0 50 100 150 200 250 300

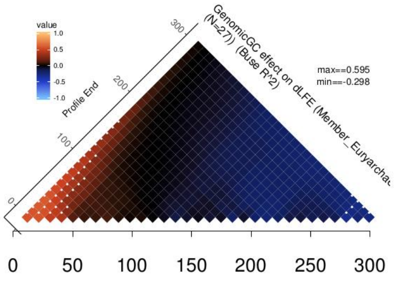
value
1.0
0.5
0.0
-0.5
-1.0
Profile End
GenomicGC effect on dLFE (Member_Euryarchae
(N=27)) (Buse R^2)
max==0.595
min==-0.298
0 50 100 150 200 250 300

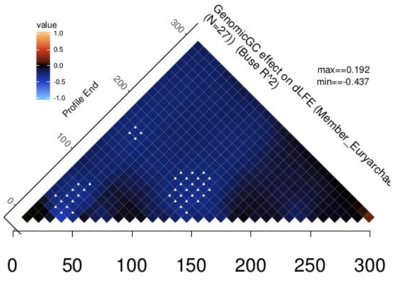
value
1.0
0.5
0.0
-0.5
-1.0
Profile End
GenomicGC effect on dLFE (Member_Euryarchae
(N=27)) (Buse R^2)
max==0.192
min==-0.437
0 50 100 150 200 250 300

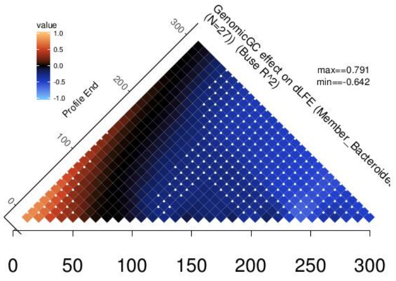
value
1.0
0.5
0.0
-0.5
-1.0
Profile End
GenomicGC effect on dLFE (Member_Bacteroide
(N=27)) (Buse R^2)
max==0.791
min==-0.642
0 50 100 150 200 250 300

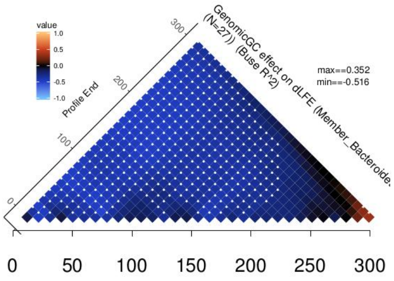
value
1.0
0.5
0.0
-0.5
-1.0
Profile End
GenomicGC effect on dLFE (Member_Bacteroide
(N=27)) (Buse R^2)
max==0.352
min==-0.516
0 50 100 150 200 250 300

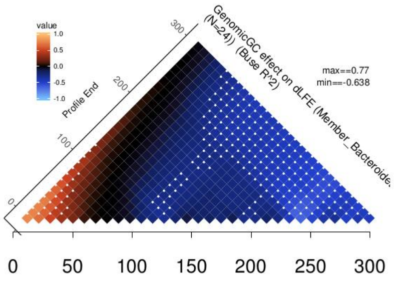
value
1.0
0.5
0.0
-0.5
-1.0
Profile End
GenomicGC effect on dLFE (Member_Bacteroide
(N=24)) (Buse R^2)
max==0.77
min==-0.638
0 50 100 150 200 250 300

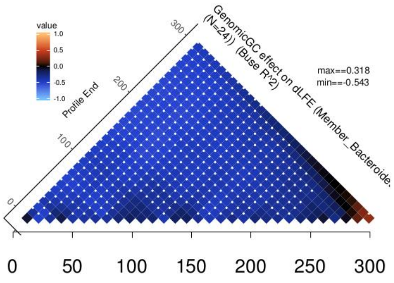
value
1.0
0.5
0.0
-0.5
-1.0
Profile End
GenomicGC effect on dLFE (Member_Bacteroide
(N=24)) (Buse R^2)
max==0.318
min==-0.543
0 50 100 150 200 250 300

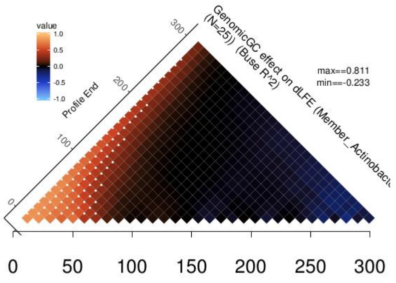
value
1.0
0.5
0.0
-0.5
-1.0
Profile End
GenomicGC effect on dLFE (Member_Actinobact
(N=25)) (Buse R^2)
max==0.811
min==-0.233
0 50 100 150 200 250 300

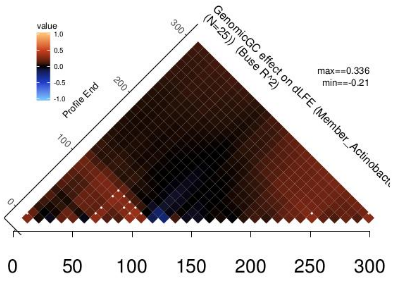
value
1.0
0.5
0.0
-0.5
-1.0
Profile End
GenomicGC effect on dLFE (Member_Actinobact
(N=25)) (Buse R^2)
max==0.336
min==-0.21
0 50 100 150 200 250 300

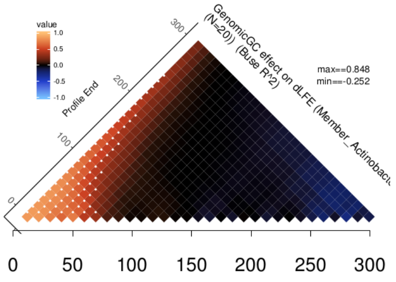
GenomicGC effect on dLFE (Member_Actinobact
(N=20)) (Buse R^2)
max==0.848
min==-0.252
value
Profile End
0 50 100 150 200 250 300

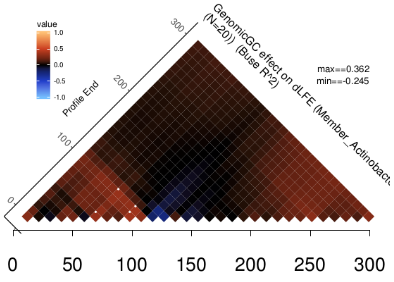
GenomicGC effect on dLFE (Member_Actinobact
(N=20)) (Buse R^2)
max==0.362
min==-0.245
value
Profile End
0 50 100 150 200 250 300

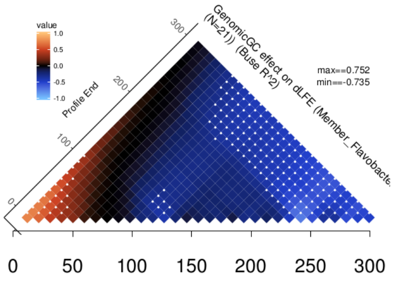
GenomicGC effect on dLFE (Member_Flavobacte
(N=21)) (Buse R^2)
max==0.752
min==-0.735
value
Profile End
0 50 100 150 200 250 300

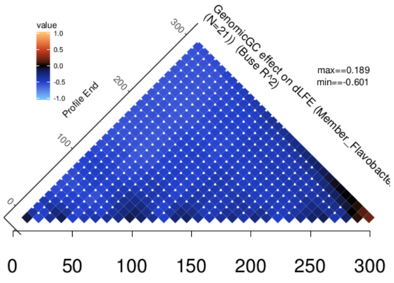
GenomicGC effect on dLFE (Member_Flavobacte
(N=21)) (Buse R^2)
max==0.189
min==-0.601
value
Profile End
0 50 100 150 200 250 300

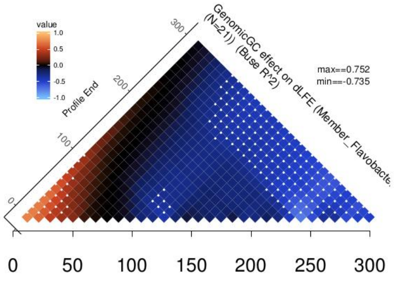
GenomicGC effect on dLFE (Member_Flavobacte
(N=21)) (Buse R^2)
max==0.752
min==-0.735
value
Profile End
0 50 100 150 200 250 300

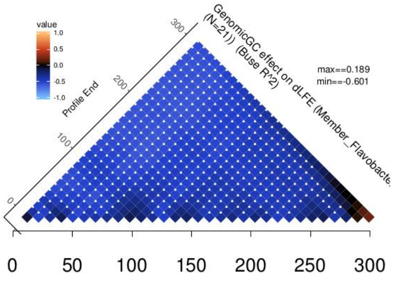
GenomicGC effect on dLFE (Member_Flavobacte
(N=21)) (Buse R^2)
max==0.189
min==-0.601
value
Profile End
0 50 100 150 200 250 300

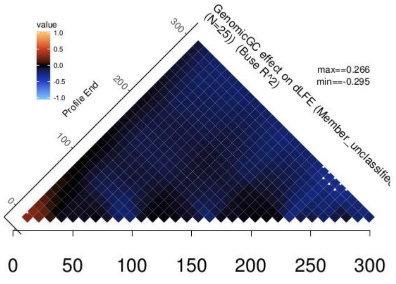
GenomicGC effect on dLFE (Member_unclassifie
(N=25)) (Buse R^2)
max==0.266
min==-0.295
value
Profile End
0 50 100 150 200 250 300

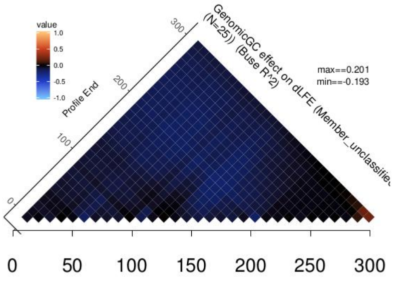
GenomicGC effect on dLFE (Member_unclassifie
(N=25)) (Buse R^2)
max==0.201
min==-0.193
value
Profile End
0 50 100 150 200 250 300

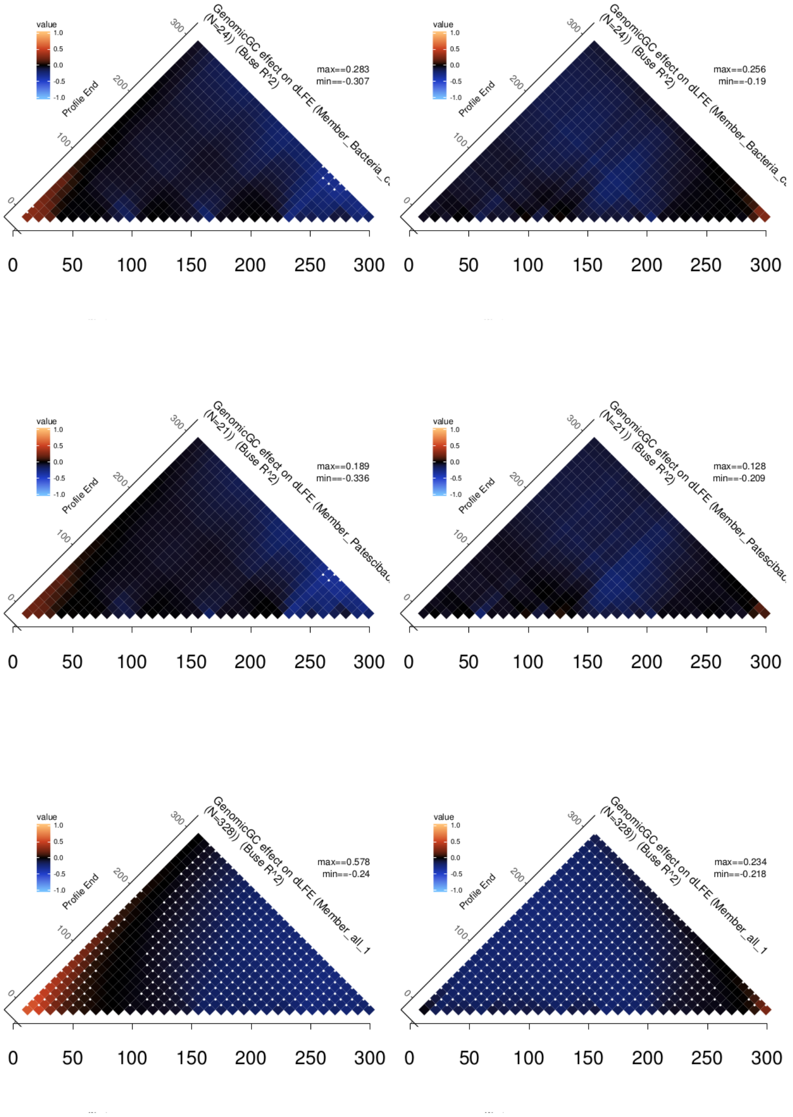

Coefficient of determination (R^2) and regression direction (red - positive slope, blue, negative slope) for GLS regression between different traits and mean ΔLFE in regions relative to CDS start and end, for different taxonomic subgroups. Significant values (p-value < 0.01) are marked with white dots.

A Genomic-GC%.

Figure S16. Additional controls for phenomenon related to translation initiation

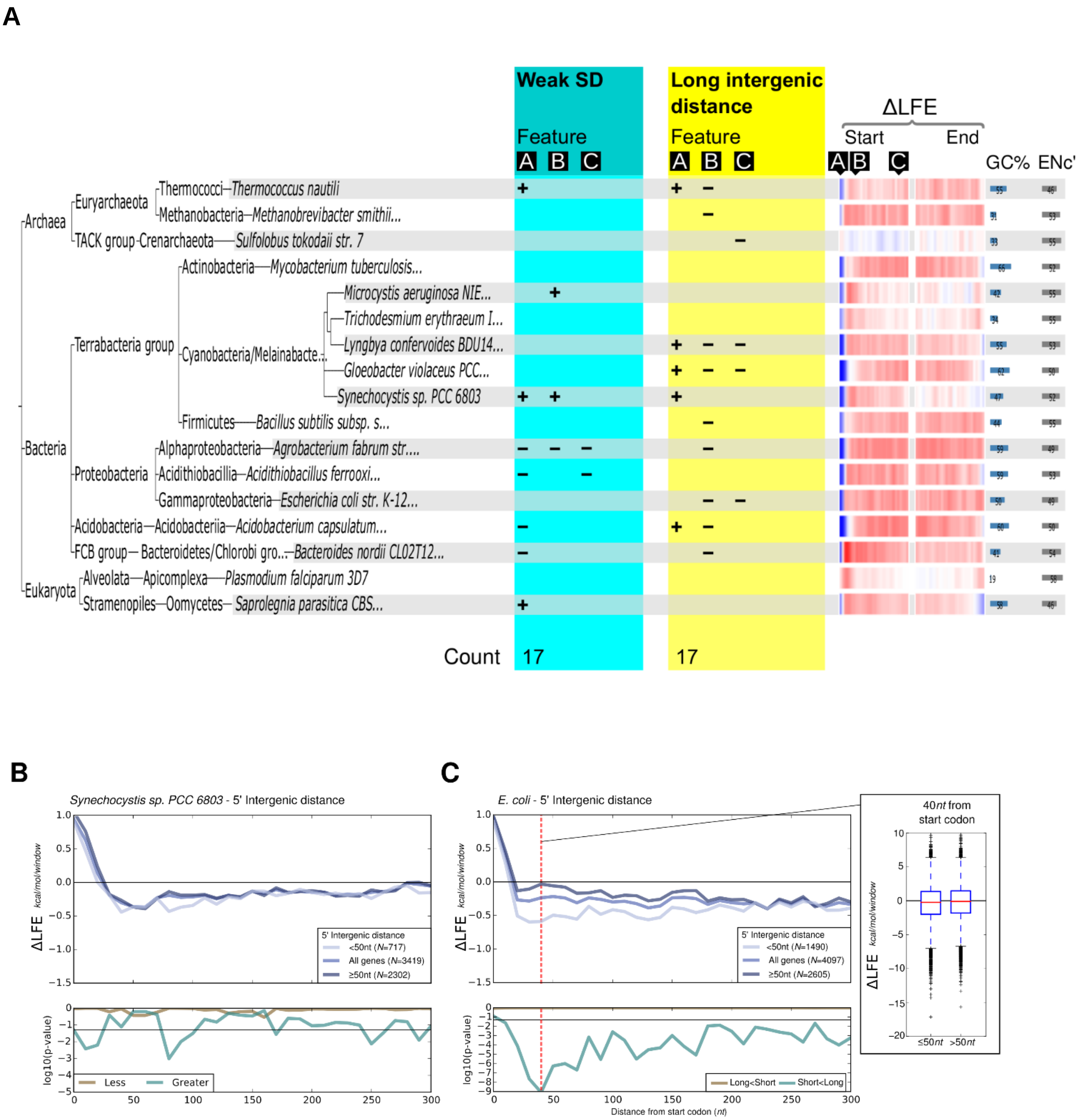

Additional controls for two potentially confounding effects relating to translation initiation.

Genes having weak SD sequence may require stronger contribution of other initiation-promoting mechanisms to ensure efficient translation initiation, and therefore might have stronger ΔLFE at the CDS start (feature [**A**]). This effect, previously reported in the 5'UTRs of S. sp. PCC6803 in(70), is also observed here.

CDS that overlap with a previous CDS may have biased ΔLFE results close to the overlapping region (see e.g. (35) for an explanation of this phenomemon in *E. coli*). As a simple control for this, we show the difference between genes with 5' intergenic distances shorter than 50nt (including overlapping genes) and other genes. Results show significant but small differences near the CDS start in some but not all species (see e.g. S. sp. And E. coli, panels **B**,**C**). Additional differences observed at other points in the CDS may be related to operonic structure. In E. coli, for example, a large decrease in mean ΔLFE is observed in genes with long intergenic distances, but the distributions of the two groups remain similar (inset on the right shows the distributions at the position 40nt from CDS start, where the effect is strongest).

For both controls, determination of each symbol (**+/−)** was based on results of a Mann-Whitney U test between the two groups of genes across the appropriate region, once for each direction (with the null hypothesis being that a value sampled from one group is not likely to be greater than an item from the other group). Fraction of positive species and total number of species are shown below for each evidence type.

SD strength was calculated according to (71), using the minimum anti-SD hybridization energy in the 20nt upstream of the start codon. The "weak SD" group includes genes with minimum energy greater than -1 *kcal/mol*.

Figure S17. Dependence of ΔLFE profiles on temperature

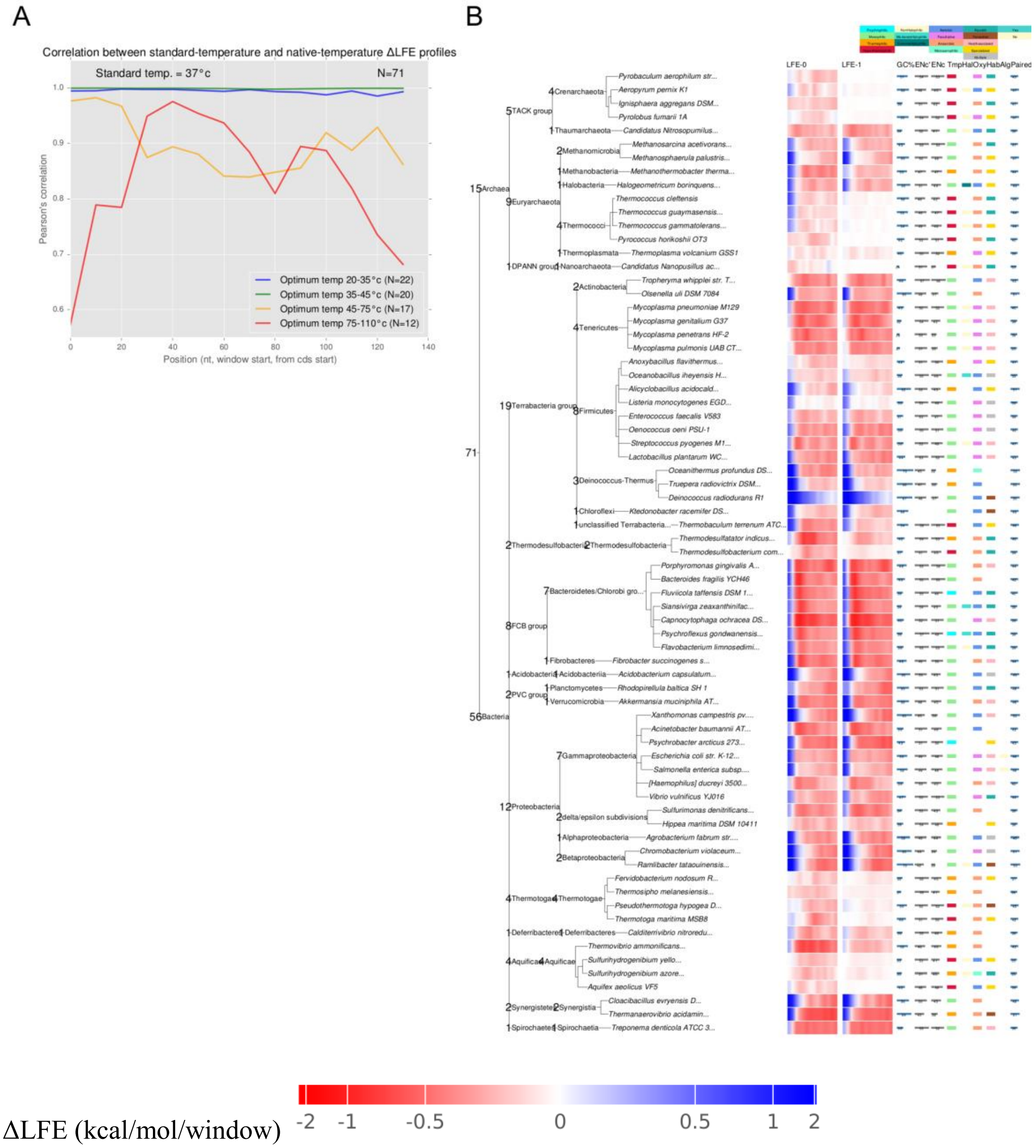

A Correlation between ΔLFE calculated using standard temperature (37°C) and native temperature (see methods), at each position relative to CDS start, for species grouped by native temperature range. Correlations were calculated for a random sample (*N*=71) of species (bacteria and archaea) for which native temperature data is available. **B** Comparison of individual mean ΔLFE profiles using calculated using standard temperature (37°C) and native temperature.

Supplementary references

1. Bist,P., Dikshit,N., Koh,T.H., Mortellaro,A., Tan,T.T. and Sukumaran,B. (2014) The Nod1, Nod2, and Rip2 Axis Contributes to Host Immune Defense against Intracellular Acinetobacter baumannii Infection. *Infect. Immun.*, **82**, 1112–1122.

2. Bai,X., Zhang,J., Ewing,A., Miller,S.A., Jancso Radek,A., Shevchenko,D.V., Tsukerman,K., Walunas,T., Lapidus,A., Campbell,J.W., *et al.* (2006) Living with genome instability: the adaptation of phytoplasmas to diverse environments of their insect and plant hosts. *J. Bacteriol.*, **188**, 3682–3696.

3. Lu,Y.-Y., Franz,B., Truttmann,M.C., Riess,T., Gay-Fraret,J., Faustmann,M., Kempf,V.A.J. and Dehio,C. (2013) Bartonella henselae trimeric autotransporter adhesin BadA expression interferes with effector translocation by the VirB/D4 type IV secretion system. *Cell. Microbiol.*, **15**, 759–778.

4. Rendulic,S., Jagtap,P., Rosinus,A., Eppinger,M., Baar,C., Lanz,C., Keller,H., Lambert,C., Evans,K.J., Goesmann,A., *et al.* (2004) A Predator Unmasked: Life Cycle of Bdellovibrio bacteriovorus from a Genomic Perspective. *Science*, **303**, 689–692.

5. Kambhampati,S., Alleman,A. and Park,Y. (2013) Complete genome sequence of the endosymbiont Blattabacterium from the cockroach Nauphoeta cinerea (Blattodea: Blaberidae). *Genomics*, **102**, 479–483.

6. DelVecchio,V.G., Kapatral,V., Redkar,R.J., Patra,G., Mujer,C., Los,T., Ivanova,N., Anderson,I., Bhattacharyya,A., Lykidis,A., *et al.* (2002) The genome sequence of the facultative intracellular pathogen Brucella melitensis. *Proc. Natl. Acad. Sci.*, **99**, 443–448.

7. Cassone,B.J., Wenger,J.A. and Michel,A.P. (2015) Whole Genome Sequence of the Soybean Aphid Endosymbiont Buchnera aphidicola and Genetic Differentiation among Biotype-Specific Strains. *J. Genomics*, **3**, 85–94.

8. Gil,R., Silva,F.J., Zientz,E., Delmotte,F., González-Candelas,F., Latorre,A., Rausell,C., Kamerbeek,J., Gadau,J., Hölldobler,B., *et al.* (2003) The genome sequence of Blochmannia floridanus: comparative analysis of reduced genomes. *Proc. Natl. Acad. Sci. U. S. A.*, **100**, 9388–9393.

9. Alves,J.M.P., Serrano,M.G., Maia da Silva,F., Voegtly,L.J., Matveyev,A.V., Teixeira,M.M.G., Camargo,E.P. and Buck,G.A. (2013) Genome Evolution and Phylogenomic Analysis of Candidatus Kinetoplastibacterium, the Betaproteobacterial Endosymbionts of Strigomonas and Angomonas. *Genome Biol. Evol.*, **5**, 338–350.

10. Hendry,T.A., de Wet,J.R. and Dunlap,P.V. (2014) Genomic signatures of obligate host dependence in the luminous bacterial symbiont of a vertebrate. *Environ. Microbiol.*, **16**, 2611–2622.

11. Thomson,N.R., Yeats,C., Bell,K., Holden,M.T.G., Bentley,S.D., Livingstone,M., Cerdeño-Tárraga,A.M., Harris,B., Doggett,J., Ormond,D., *et al.* (2005) The Chlamydophila abortus genome sequence reveals an array of variable proteins that contribute to interspecies variation. *Genome Res.*, **15**, 629–640.

12. Kalman,S., Mitchell,W., Marathe,R., Lammel,C., Fan,J., Hyman,R.W., Olinger,L., Grimwood,J., Davis,R.W. and Stephens,R.S. (1999) Comparative genomes of Chlamydia pneumoniae and C. trachomatis. *Nat. Genet.*, **21**, 385–389.

13. Seshadri,R., Paulsen,I.T., Eisen,J.A., Read,T.D., Nelson,K.E., Nelson,W.C., Ward,N.L., Tettelin,H., Davidsen,T.M., Beanan,M.J., *et al.* (2003) Complete genome sequence of the Q-fever pathogen Coxiella burnetii. *Proc. Natl. Acad. Sci. U. S. A.*, **100**, 5455–5460.

14. WEEDALL,G.D. and HALL,N. (2015) Sexual reproduction and genetic exchange in parasitic protists. *Parasitology*, **142**, S120–S127.

15. Enos,B.G., Anthony,M.K., DeGiorgis,J.A. and Williams,L.E. (2018) Prey Range and Genome Evolution of Halobacteriovorax marinus Predatory Bacteria from an Estuary. *mSphere*, **3**.

16. Dohra,H., Suzuki,H., Suzuki,T., Tanaka,K. and Fujishima,M. (2013) Draft Genome Sequence of Holospora undulata Strain HU1, a Micronucleus-Specific Symbiont of the Ciliate Paramecium caudatum. *Genome Announc.*, **1**.

17. Ivens,A.C., Peacock,C.S., Worthey,E.A., Murphy,L., Aggarwal,G., Berriman,M., Sisk,E., Rajandream,M.-A., Adlem,E., Aert,R., *et al.* (2005) The Genome of the Kinetoplastid Parasite, Leishmania major. *Science*, **309**, 436–442.

18. Gaillard,J.L., Berche,P., Mounier,J., Richard,S. and Sansonetti,P. (1987) In vitro model of penetration and intracellular growth of Listeria monocytogenes in the human enterocyte-like cell line Caco-2. *Infect. Immun.*, **55**, 2822–2829.

19. Sibley,L.D., Franzblau,S.G. and Krahenbuhl,J.L. (1987) Intracellular fate of Mycobacterium leprae in normal and activated mouse macrophages. *Infect. Immun.*, **55**, 680–685.

20. Cole,S.T., Brosch,R., Parkhill,J., Garnier,T., Churcher,C., Harris,D., Gordon,S.V., Eiglmeier,K., Gas,S., Barry Iii,C.E., *et al.* (1998) Deciphering the biology of *Mycobacterium tuberculosis* from the complete genome sequence. *Nature*, **393**, 537–544.

21. Sasaki,Y., Ishikawa,J., Yamashita,A., Oshima,K., Kenri,T., Furuya,K., Yoshino,C., Horino,A., Shiba,T., Sasaki,T., *et al.* (2002) The complete genomic sequence of Mycoplasma

penetrans, an intracellular bacterial pathogen in humans. *Nucleic Acids Res.*, **30**, 5293–5300.

22. Waters,E., Hohn,M.J., Ahel,I., Graham,D.E., Adams,M.D., Barnstead,M., Beeson,K.Y., Bibbs,L., Bolanos,R., Keller,M., *et al.* (2003) The genome of Nanoarchaeum equitans: insights into early archaeal evolution and derived parasitism. *Proc. Natl. Acad. Sci. U. S. A.*, **100**, 12984–12988.

23. OSHIMA,K., MIYATA,S., SAWAYANAGI,T., KAKIZAWA,S., NISHIGAWA,H., JUNG,H.-Y., FURUKI,K., YANAZAKI,M., SUZUKI,S., WEI,W., *et al.* (2002) Minimal Set of Metabolic Pathways Suggested from the Genome of Onion Yellows Phytoplasma. *J. Gen. Plant Pathol.*, **68**, 225–236.

24. Fryer,J.L. and Hedrick,R.P. (2003) Piscirickettsia salmonis: a Gram-negative intracellular bacterial pathogen of fish. *J. Fish Dis.*, **26**, 251–262.

25. Maier,A.G., Cooke,B.M., Cowman,A.F. and Tilley,L. (2009) Malaria parasite proteins that remodel the host erythrocyte. *Nat. Rev. Microbiol.*, **7**, 341–354.

26. Zeev,E.B., Yogev,T., Man-Aharonovich,D., Kress,N., Herut,B., Béjà,O. and Berman-Frank,I. (2008) Seasonal dynamics of the endosymbiotic, nitrogen-fixing cyanobacterium *Richelia intracellularis* in the eastern Mediterranean Sea. *ISME J.*, **2**, 911–923.

27. Richter-Dahlfors,A., Buchan,A.M.J. and Finlay,B.B. (1997) Murine Salmonellosis Studied by Confocal Microscopy: Salmonella typhimurium Resides Intracellularly Inside Macrophages and Exerts a Cytotoxic Effect on Phagocytes In Vivo. *J. Exp. Med.*, **186**, 569–580.

28. Knab,S., Mushak,T.M., Schmitz-Esser,S., Horn,M. and Haferkamp,I. (2011) Nucleotide Parasitism by Simkania negevensis (Chlamydiae). *J. Bacteriol.*, **193**, 225–235.

29. Finan,T.M., Weidner,S., Wong,K., Buhrmester,J., Chain,P., Vorhölter,F.J., Hernandez-Lucas,I., Becker,A., Cowie,A., Gouzy,J., *et al.* (2001) The complete sequence of the 1,683-kb pSymB megaplasmid from the N2-fixing endosymbiont Sinorhizobium meliloti. *Proc. Natl. Acad. Sci.*, **98**, 9889–9894.

30. Soldati,D. and Boothroyd,J.C. (1993) Transient transfection and expression in the obligate intracellular parasite Toxoplasma gondii. *Science*, **260**, 349–352.

31. Raoult,D., Ogata,H., Audic,S., Robert,C., Suhre,K., Drancourt,M. and Claverie,J.-M. (2003) Tropheryma whipplei Twist: A Human Pathogenic Actinobacteria With a Reduced Genome. *Genome Res.*, **13**, 1800–1809.

32. Rassi,A., Rassi,A. and Marin-Neto,J.A. (2010) Chagas disease. *The Lancet*, **375**, 1388–1402.

33. Newton,I.L.G., Clark,M.E., Kent,B.N., Bordenstein,S.R., Qu,J., Richards,S., Kelkar,Y.D. and Werren,J.H. (2016) Comparative Genomics of Two Closely Related Wolbachia with Different Reproductive Effects on Hosts. *Genome Biol. Evol.*, **8**, 1526–1542.

34. Xia,X. (2015) A Major Controversy in Codon-Anticodon Adaptation Resolved by a New Codon Usage Index. *Genetics*, **199**, 573–579.

35. Xia,X. (2019) Optimizing Phage Translation Initiation. *OBM Genet.*, **3**, 1–1.

36. Bolger,A.M., Lohse,M. and Usadel,B. (2014) Trimmomatic: a flexible trimmer for Illumina sequence data. *Bioinformatics*, **30**, 2114–2120.

37. Kersey,P.J., Allen,J.E., Allot,A., Barba,M., Boddu,S., Bolt,B.J., Carvalho-Silva,D., Christensen,M., Davis,P., Grabmueller,C., *et al.* (2017) Ensembl Genomes 2018: an integrated omics infrastructure for non-vertebrate species. *Nucleic Acids Res.*, 10.1093/nar/gkx1011.

38. Sayers,E.W., Beck,J., Brister,J.R., Bolton,E.E., Canese,K., Comeau,D.C., Funk,K., Ketter,A., Kim,S., Kimchi,A., *et al.* Database resources of the National Center for Biotechnology Information. *Nucleic Acids Res.*, 10.1093/nar/gkz899.

39. Langmead,B. and Salzberg,S.L. (2012) Fast gapped-read alignment with Bowtie 2. *Nat. Methods*, **9**, 357–359.

40. Anders,S., Pyl,P.T. and Huber,W. (2015) HTSeq—a Python framework to work with high-throughput sequencing data. *Bioinformatics*, **31**, 166–169.

41. Wang,M., Weiss,M., Simonovic,M., Haertinger,G., Schrimpf,S.P., Hengartner,M.O. and von Mering,C. (2012) PaxDb, a Database of Protein Abundance Averages Across All Three Domains of Life. *Mol. Cell. Proteomics*, **11**, 492–500.

42. Xia,X. (2018) DAMBE7: New and Improved Tools for Data Analysis in Molecular Biology and Evolution. *Mol. Biol. Evol.*, **35**, 1550–1552.

43. González-Quiñónez,N., Corte-Rodríguez,M., Álvarez-Fernández-García,R., Rioseras,B., López-García,M.T., Fernández-García,G., Montes-Bayón,M., Manteca,A. and Yagüe,P. (2019) Cytosolic copper is a major modulator of germination, development and secondary metabolism in Streptomyces coelicolor. *Sci. Rep.*, **9**, 1–18.

44. Serafini,A., Tan,L., Horswell,S., Howell,S., Greenwood,D.J., Hunt,D.M., Phan,M.-D., Schembri,M., Monteleone,M., Montague,C.R., *et al.* (2019) Mycobacterium tuberculosis requires glyoxylate shunt and reverse methylcitrate cycle for lactate and pyruvate metabolism. *Mol. Microbiol.*, **112**, 1284–1307.

45. Wang,X., Wu,H., Niu,T., Bi,J., Hou,H., Hao,H. and Zhang,G. (2019) Downregulated Expression of Virulence Factors Induced by Benzyl Isothiocyanate in Staphylococcus Aureus: A Transcriptomic Analysis. *Int. J. Mol. Sci.*, **20**, 5441.

46. Forrest,D., James,K., Yuzenkova,Y. and Zenkin,N. (2017) Single-peptide DNA-dependent RNA polymerase homologous to multi-subunit RNA polymerase. *Nat. Commun.*, **8**, 1–8.

47. Dorey,A.L., Lee,B.-H., Rotter,B. and O'Byrne,C.P. (2019) Blue Light Sensing in Listeria monocytogenes Is Temperature-Dependent and the Transcriptional Response to It Is Predominantly SigB-Dependent. *Front. Microbiol.*, **10**.

48. Do,H., Makthal,N., Chandrangsu,P., Olsen,R.J., Helmann,J.D., Musser,J.M. and Kumaraswami,M. (2019) Metal sensing and regulation of adaptive responses to manganese limitation by MtsR is critical for group A streptococcus virulence. *Nucleic Acids Res.*, **47**, 7476–7493.

49. Filannino,P., Angelis,M.D., Cagno,R.D., Gozzi,G., Riciputi,Y. and Gobbetti,M. (2018) How Lactobacillus plantarum shapes its transcriptome in response to contrasting habitats. *Environ. Microbiol.*, **20**, 3700–3716.

50. Srivastava,A., Jeong,H., Ko,S.-R., Ahn,C.-Y., Choi,J.W., Park,Y.I., Neilan,B.A. and Oh,H.-M. (2019) Phenotypic niche partitioning and transcriptional responses of Microcystis aeruginosa in a spatially heterogeneous environment. *Algal Res.*, **41**, 101551.

51. Briliūtė,J., Urbanowicz,P.A., Luis,A.S., Baslé,A., Paterson,N., Rebello,O., Hendel,J., Ndeh,D.A., Lowe,E.C., Martens,E.C., *et al.* (2019) Complex N -glycan breakdown by gut Bacteroides involves an extensive enzymatic apparatus encoded by multiple co-regulated genetic loci. *Nat. Microbiol.*, **4**, 1571–1581.

52. Coats,S.R., Kantrong,N., To,T.T., Jain,S., Genco,C.A., McLean,J.S. and Darveau,R.P. (2019) The Distinct Immune-Stimulatory Capacities of Porphyromonas gingivalis Strains 381 and ATCC 33277 Are Determined by the fimB Allele and Gingipain Activity. *Infect. Immun.*, **87**.

53. Fan,Y., Thompson,L., Lyu,Z., Cameron,T.A., De Lay,N.R., Krachler,A.M. and Ling,J. (2019) Optimal translational fidelity is critical for Salmonella virulence and host interactions. *Nucleic Acids Res.*, **47**, 5356–5367.

54. Anand,A., Chen,K., Catoiu,E., Sastry,A.V., Olson,C.A., Sandberg,T.E., Seif,Y., Xu,S., Szubin,R., Yang,L., *et al.* OxyR Is a Convergent Target for Mutations Acquired during Adaptation to Oxidative Stress-Prone Metabolic States. *Mol. Biol. Evol.*, 10.1093/molbev/msz251.

55. Meibom,K.L., Cabello,E.M. and Bernier-Latmani,R. (2018) The Small RNA RyhB Is a Regulator of Cytochrome Expression in Shewanella oneidensis. *Front. Microbiol.*, **9**.

56. Dwidar,M., Im,H., Seo,J.K. and Mitchell,R.J. (2017) Attack-Phase Bdellovibrio bacteriovorus Responses to Extracellular Nutrients Are Analogous to Those Seen During Late Intraperiplasmic Growth. *Microb. Ecol.*, **74**, 937–946.

57. Fiebig,A., Varesio,L.M., Navarreto,X.A. and Crosson,S. (2019) Regulation of the Erythrobacter litoralis DSM 8509 general stress response by visible light. *Mol. Microbiol.*, **112**, 442–460.

58. Tram,G., Klare,W.P., Cain,J.A., Mourad,B., Cordwell,S.J., Korolik,V. and Day,C.J. (2019) Assigning a role for chemosensory signal transduction in Campylobacter jejuni biofilms using a combined omics approach. *bioRxiv*, 10.1101/862151.

59. Chen,Z., Gao,S., Jin,M., Sun,S., Lu,J., Yang,P., Bond,P.L., Yuan,Z. and Guo,J. (2019) Physiological and transcriptomic analyses reveal CuO nanoparticle inhibition of anabolic and catabolic activities of sulfate-reducing bacterium. *Environ. Int.*, **125**, 65–74.

60. Cochrane,K., Robinson,A.V., Holt,R.A. and Allen-Vercoe,E. (2019) A survey of Fusobacterium nucleatum genes modulated by host cell infection. *Microb. Genomics*, 10.1099/mgen.0.000300.

61. Sebé-Pedrós,A., Chomsky,E., Pang,K., Lara-Astiaso,D., Gaiti,F., Mukamel,Z., Amit,I., Hejnol,A., Degnan,B.M. and Tanay,A. (2018) Early metazoan cell type diversity and the evolution of multicellular gene regulation. *Nat. Ecol. Evol.*, **2**, 1176–1188.

62. Wijesena,N., Simmons,D.K. and Martindale,M.Q. (2017) Antagonistic BMP–cWNT signaling in the cnidarian Nematostella vectensis reveals insight into the evolution of mesoderm. *Proc. Natl. Acad. Sci.*, **114**, E5608–E5615.

63. Nicolás,C., Martin-Bertelsen,T., Floudas,D., Bentzer,J., Smits,M., Johansson,T., Troein,C., Persson,P. and Tunlid,A. (2019) The soil organic matter decomposition mechanisms in ectomycorrhizal fungi are tuned for liberating soil organic nitrogen. *ISME J.*, **13**, 977–988.

64. Schäpe,P., Kwon,M.J., Baumann,B., Gutschmann,B., Jung,S., Lenz,S., Nitsche,B., Paege,N., Schütze,T., Cairns,T.C., *et al.* (2019) Updating genome annotation for the microbial cell factory Aspergillus niger using gene co-expression networks. *Nucleic Acids Res.*, **47**, 559–569.

65. Dulmage,K.A., Darnell,C.L., Vreugdenhil,A. and Schmid,A.K. (2018) Copy number variation is associated with gene expression change in archaea. *Microb. Genomics*, **4**, e000210.

66. Sharp,P.M. and Li,W.H. (1987) The codon Adaptation Index--a measure of directional synonymous codon usage bias, and its potential applications. *Nucleic Acids Res.*, **15**, 1281–1295.

67. Peden,J. (1997) CodonW. *Trinity Coll.*

68. Novembre,J.A. (2002) Accounting for Background Nucleotide Composition When Measuring Codon Usage Bias. *Mol. Biol. Evol.*, **19**, 1390–1394.

69. Sabi,R. and Tuller,T. (2014) Modelling the Efficiency of Codon–tRNA Interactions Based on Codon Usage Bias. *DNA Res.*, **21**, 511–526.

70. Wei,Y. and Xia,X. (2019) Unique Shine–Dalgarno Sequences in Cyanobacteria and Chloroplasts Reveal Evolutionary Differences in Their Translation Initiation. *Genome Biol. Evol.*, **11**, 3194–3206.

71. Bahiri Elitzur,S., Cohen-Kupiec,R., Fine,L., Yacobi,D., Apt,B., Diament,A. and Tuller,T. (2020) Prokaryotic rRNA-mRNA interactions are involved in all translation steps and shape bacterial transcripts. *Rev.*
