# Supplementary material for: High-resolution modeling of the selection on local mRNA folding strength in coding sequences across the tree of life
Source: Genome Biol. 2020 Mar 9;21:63. doi: 10.1186/s13059-020-01971-y (PMC7063772; doi:10.1186/s13059-020-01971-y)
Supplement: Supplementary file 4 — Additional file 4. Review history. [file 13059_2020_1971_MOESM4_ESM.docx]

Review History

**First round of review**

**Reviewer 1**

**Are you able to assess all statistics in the manuscript, including the appropriateness of statistical tests used?**

Yes, and I have assessed the statistics in my report.

**Comments to author:**

This study is exploratory, without specific hypotheses to test or biological problems to solve. The main finding, highlighted in the abstract, is that coding sequences have "weak folding at the two ends of the coding region and for strong folding elsewhere in the coding sequence."

There are many papers where quantifying secondary structure leads to significant biological insights. For example, a set of about 10 yeast genes are transcribed but not translated until the yeast cells experience nutrient limitation, and this set of yeast genes share the feature of weak secondary structure at 5'UTR immediately upstream of the start codon that facilitate internal ribosome entry when the global cap-dependent translation is largely shutdown due to nutrient limitation. The translation of these genes allow the yeast cells to burrow down to make use of nutrients below the surface of culture medium (Gilbert et al., 2007). In particular, this association between weak secondary structure and IRES activity is consistent in both yeast and fruit fly (Xia and Holcik, 2009). In fact, association between strength of secondary structure of mRNA and nutrient limitation has also been documented in fruit flies (Zid et al., 2009). Zid et al. (2009) characterized secondary structure stability by using DAMBE which implements a sliding-window approach to characterize local secondary structure strength (LFE in this paper). The authors should be aware of these conceptual and methodological progresses.

As to the main finding of "weak folding at the two ends of the coding region and for strong folding elsewhere in the coding sequence", this is not new and has already been reported before, at least in bacterial species, with the interpretation that translation initiation and termination signals should not be embedded in a stable secondary structure, especially in highly expressed genes (Xia, 2017; Xia, 2018). See Figure 3 in Xia (2017) and Figure 8.6 in Xia (2018).

In Methods, "Since some node identifiers do not match exactly between the tree (Supplementary Dataset 2) and accessions table (Supplementary Table 1), species names were matched conservatively using edit-distance (with a hand-tuned scoring function)", this is not acceptable. How likely is one to have a mismatch? Why is it necessary to allow a chance to err? Overall, the section "Phylogenetic tree preparation" is so vague that no expert in the field could possibly replicate the result. For example, it mentions "tree distances" which has never been defined. Is it Robinson-Foulds distance or what?

For "Phylogenetically-controlled regression", one paper cited "Aitken 1936" has no relevance to "Phylogenetically-controlled regression".

In short, the paper in its current form is far from publication quality.

Gilbert WV, Zhou K, Butler TK, Doudna JA. 2007. Cap-independent translation is required for starvation-induced differentiation in yeast. Science 317(5842):1224-7.
Xia X. 2017. DAMBE6: New Tools for Microbial Genomics, Phylogenetics, and Molecular Evolution. J Hered 108(4):431-437.
Xia X. 2018. Bioinformatics and Translation Initiation. Bioinformatics and the Cell: modern computational approaches in genomics, proteomics and transcriptomics. Springer, Cham. p. 173-195.
Xia X, Holcik M. 2009. Strong Eukaryotic IRESs Have Weak Secondary Structure. PLoS ONE 4(1):e4136.
Zid BM, Rogers AN, Katewa SD, Vargas MA, Kolipinski MC, Lu TA, Benzer S, Kapahi P. 2009. 4E-BP extends lifespan upon dietary restriction by enhancing mitochondrial activity in Drosophila. Cell 139(1):149-60.

**Reviewer 2**

**Are you able to assess all statistics in the manuscript, including the appropriateness of statistical tests used?**

No, I do not feel adequately qualified to assess the statistics.

**Comments to author:**

This is a highly interesting and timely study of the mRNA folding strength, apparently the most comprehensive and technically solid to date. I have no doubts that it will be of great relevance for the journal readership. Scientifically, it is virtually impeccable and very convincing. In particular, the authors took great care to control for possible biases in their calculations.

Unfortunately there is one technical problem that necessitates a revision: the structure of the figures. They are overloaded with detail and insufficiently explained. Upon trying to comprehend them for a few days I am giving up.

Let us start with figure 2a. It is supposed to demonstrate that the short initial regions are less structured as the random control and thus would have negative dLFE values and hence colored blue. I spent several days looking at the printed version of Figure 1 and could not see anything blue on the left end. It then occured to me to look at the same figure in the PDF file and that is how I found that, indeed, the left end tends to be blue, but the blue stripes are so narrow that they cannot be seen in print. Why are there different numbers of stripes? E.g., for Opisthokonta there are four stripes on the left and two on the right while for Terrabacteria there are three on the left and five on the right. Why is that and what is that supposed to mean?

I cannot comprehend the PC analysis shown in Figure 2b. I do not know what the abbreviations here and elswehre mean (Spun, Dser - apparently these are organism names, by where is the explanation?) and the message of this figure is not clear to me. Figure 2B is not discussed in the text at all, the only sentence I could find is "The division of the profile into the three regions described here is also apparent when the data is analyzed in an unsupervised manner via Principal Components Analysis". That does not explain anything.

In Figure 1 there are abbreviations A1, A2, B1, B2, and B which are not explained at all (I figured that these are probably different hypotheses being tested). GLC and CUB are explained in the methods, but should also be explained in the legend. ENc' is not explained at all.,


In Figure 3A the results for the two models 1 and 2 are vastly different. Why? WHere is this discussed and what are the implications?

I do not know what the arrows in Figure 4E mean and I could not find any discussion of this figure in the text. What does it say?

Figure 5. What is MINE? I managed to find the explanation for MIC in the methods. What does this mean: CDS start + 100 - 300? What does the tilted red line close to the zero line mean? Upon some consideration I figured that this is probably the linear model, because paremeters are also in red in the upper right corner. But then, what does the green color mean?

On all PC graphs, the loadings of individual PCs are shown (e.g. Figure 5B, PC1 has 80.3%), but I could not find any interpretation what the meaning of these first  two PCs could be.

Do not even ask me how to read Figure 9 - no idea.

I also do not like the fact that the first figure to mentioned in the text is Figure 3a.

In summary: this is a scientifically excellent study, but please make figures less busy, perhaps split them in better understandable items, and explain their meaning clearly in the text.

**Authors Response**

**Point-by-point responses to the reviewers’ comments:**

Reviewer #1

This study is exploratory, without specific hypotheses to test or biological problems to solve.

*We do not agree with the statement above. In fact, our paper was motivated by specific hypotheses (which are now made clearer in the abstract, introduction and the discussion). Specifically, we investigated the following hypotheses:*

*Local mRNA folding strength affects many intracellular processes and is thus expected to be under direct selection.*

*The strength of this selection should vary across the tree of life due to variation in relevant genomic and environmental factors (either affecting how beneficial secondary structure is for gene expression or how effectively it can be maintained under selection).*

*The strength of this selection should vary along the coding sequence since it affects gene expression regulation processes, which involve different factors or biophysics in different parts of the coding sequence.*

*Thus, the specific objective of the study is to perform the first analysis of local mRNA folding selection along the coding region and across all domains in the tree of life while accurately inferring association between local mRNA folding and genomic and environmental factors.*

The main finding, highlighted in the abstract, is that coding sequences have "weak folding at the two ends of the coding region and for strong folding elsewhere in the coding sequence."

*This statement is also not true. The point above is not the main finding. It is a restatement of previous findings we were able to confirm (using a much larger sample size and under stricter controls) while at the same time finding widespread exceptions for (Figure 1A). We report many additional findings, including:*

*We suggest a novel statistical framework for studying selection for local mRNA folding along the coding region and across taxonomic groups while controlling for confounding factors such as amino acid bias, codon bias, and evolutionary distance. The approach enables inferring statistical variables that may directly affect local mRNA folding.*

*The high correlation between the strengths of selection on local folding at different regions of the coding sequence, which indicates the existence of common factors influencing it everywhere*

*The identification of four factors having strong predictive effect on local mRNA folding selection within the coding sequence (i.e., GC-content, CUB, intracellular life stage, hyperthermophilic environment), linking this phenomenon to genomic and environmental traits and gene expression regulation.*

*Demonstrating that the prevalence of the different regions of local folding selection varies between taxons. For example, the selection for weak folding at the end of the coding region seems to be more common in eukaryotes while the selection for weak folding at the beginning of the coding region appears more commonly in prokaryotes.*

*Weaker than expected folding on average in mid-CDS is only found in one bacterial species out of 371 tested, despite being quite common in eukaryotes.*

*A transition peak of selection for strong mRNA folding around 30-70nt downstream of the start codon appears in ~50% of the analyzed organisms.*

There are many papers where quantifying secondary structure leads to significant biological insights. For example, a set of about 10 yeast genes are transcribed but not translated until the yeast cells experience nutrient limitation, and this set of yeast genes share the feature of weak secondary structure at 5'UTR immediately upstream of the start codon that facilitate internal ribosome entry when the global cap-dependent translation is largely shutdown due to nutrient limitation. The translation of these genes allow the yeast cells to burrow down to make use of nutrients below the surface of culture medium (Gilbert et al., 2007). In particular, this association between weak secondary structure and IRES activity is consistent in both yeast and fruit fly (Xia and Holcik, 2009). In fact, association between strength of secondary structure of mRNA and nutrient limitation has also been documented in fruit flies (Zid et al., 2009).

*Indeed, there are many studies about local mRNA folding (including more than 10 papers from our group). Yet, none of them includes the key results mentioned above. The papers mentioned by the reviewer focus on a very small number of genes with IRES or 4E-BP translation initiation,while we study all coding genes looking along the entire coding region. They focus on yeast and fruit fly, while we look on hundreds of species from the three domains of life. They focus on the 5’-UTR region, which our study excludes (though the results may be caused by the same mechanism in the relevant genes).*

*Specifically,*

*(Gilbert et al., 2007) studied translation initiation in 7 yeast genes that have IRES sites and find that mRNA secondary structure is not required for IRES-dependent translation initiation at these sites.*

*(Xia and Holcik, 2009) studied the effect of secondary structure on IRES in 12 yeast and 5 fruit fly genes. They find that secondary structure strength in the 5’-UTR is inversely correlated with IRES-dependent translation initiation.*

*(Zid et al., 2009) studied mitochondrial genes from the fruit fly genome for translation initiation using the 4E-BP mechanism.*

*These papers are complementary in that they demonstrate the effect of mRNA secondary structure in the 5’-UTR on different translation initiation mechanisms in higher eukaryotes. However, they don’t share our focus on the coding sequence, nor our scope of examining how this phenomenon varies and evolves across the tree of life.*

*To better place our work within the context of the significant small-scale and large-scale experimental evidence available (which we, in a way, are trying to generalize), we now cite more broadly in the introduction. In particular, we now cite all the papers mentioned above as examples of the specific ways mRNA folding (within and outside the coding sequence) can affect gene expression.*

Zid et al. (2009) characterized secondary structure stability by using DAMBE which implements a sliding-window approach to characterize local secondary structure strength (LFE in this paper).

The authors should be aware of these conceptual and methodological progresses.

*Thank you for the comment.*

*Zid et al. applied a sliding window approach (LFE) to the 5’-UTR of mitochondrial genes in Drosophila. For the purpose we are pursuing, this is not a “conceptual and methodological progress” but rather a step back. LFE doesn’t control for codon-bias and amino-acid composition (known to change in different regions of the coding sequence), hence the introduction of ΔLFE (as we explain in section 3 and section 6.1 under “Randomization procedures”). Claiming relatively novel selective forces based on computational evidence should have a high bar of evidence, and controlling at the very least for the amino-acid composition (which varies across the protein) should be a requirement.*

*Just to be clear, we do not claim the introduction of ΔLFE is a result of the study (and we cite many papers that used it previously). The original results are the biological conclusions derived from applying it across the tree of life and correlating its results using phylogenetically-controlled regression to find which factors control it.*

As to the main finding of "weak folding at the two ends of the coding region and for strong folding elsewhere in the coding sequence", this is not new and has already been reported before, at least in bacterial species, with the interpretation that translation initiation and termination signals should not be embedded in a stable secondary structure, especially in highly expressed genes (Xia, 2017; Xia, 2018). See Figure 3 in Xia (2017) and Figure 8.6 in Xia (2018).

*The papers mentioned by the reviewer are of course important. However:*

*First, please see the list of novel discoveries in our study (above), which contains many additional results (including how well the observation from E. coli generalizes to other species).*

*Second, note that Xia et al. only shows LFE (not ΔLFE) and only in 2,000 E. coli genes (and suggests that the folding is caused by rho-independent transcription termination). Via our control for codon usage bias and AA content (i.e., ΔLFE) and the analysis of 513 organisms from the three domains of life, we show that there is evidence of direct selection for weak folding near the end of the coding sequence in 37% of the organism tested. Weak or positive ΔLFE (not LFE) at the end of the coding sequence has not been previously reported (to our knowledge), and neither has the general trend (profile) of ΔLFE, present in most bacteria and some archaea and eukaryotes, which integrates the previously published observations.*

*Third, we cited previous works extensively (e.g. in Figure 1A) and attempt to avoid claiming novelty where this is unjustified. We previously focused mainly on papers controlling for the amino-acid and codon distributions (as explained above), but have now added a citation of Xia et al. as well as Shabalina (2006), which also showed similar results without controlling for amino-acid and codon biases.*

In Methods, "Since some node identifiers do not match exactly between the tree (Supplementary Dataset 2) and accessions table (Supplementary Table 1), species names were matched conservatively using edit-distance (with a hand-tuned scoring function)", this is not acceptable. How likely is one to have a mismatch? Why is it necessary to allow a chance to err?

*We are sorry for the lack in clarity here. We did accurately match all the species names (in some cases it was done manually). As we explain, the reason for this complication is that the paper cited (Hug et al.), which provides an invaluable resource for our work, unfortunately contains minor discrepancies in the identifiers they used. We tried to be transparent about this issue (which others attempting to reproduce our research might encounter), and have now improved the explanation in section 6.1.*

Overall, the section "Phylogenetic tree preparation" is so vague that no expert in the field could possibly replicate the result. For example, it mentions "tree distances" which has never been defined. Is it Robinson-Foulds distance or what?

*We are sorry for the lack in clarity here. We were referring to distances along the evolutionary tree induced by edge length and not distances between trees (indeed, there is only one tree used in this paper). We now better explain this point and addressed a few minor issues, and believe the section is now quite detailed and thorough.*

For "Phylogenetically-controlled regression", one paper cited "Aitken 1936" has no relevance to "Phylogenetically-controlled regression".

*The paper cited introduced GLS, which is the statistical method we are using, as well as the R2 calculation for GLS in Figures 4A and 5A. We also cite a relevant textbook chapter (Paradis) on this procedure.*

In short, the paper in its current form is far from publication quality.

*Thank you for all the comments. We addressed all of them in the current version and hope you’ll find our paper worthy. As explained above, we believe that the study which we have been working on for over 3 years has many important contributions to the field.*

Gilbert WV, Zhou K, Butler TK, Doudna JA. 2007. Cap-independent translation is required for starvation-induced differentiation in yeast. Science 317(5842):1224-7.

Xia X. 2017. DAMBE6: New Tools for Microbial Genomics, Phylogenetics, and Molecular Evolution. J Hered 108(4):431-437.

Xia X. 2018. Bioinformatics and Translation Initiation. Bioinformatics and the Cell: modern computational approaches in genomics, proteomics and transcriptomics. Springer, Cham. p. 173- 195.

Xia X, Holcik M. 2009. Strong Eukaryotic IRESs Have Weak Secondary Structure. PLoS ONE 4(1):e4136.

Zid BM, Rogers AN, Katewa SD, Vargas MA, Kolipinski MC, Lu TA, Benzer S, Kapahi P. 2009. 4E-BP extends lifespan upon dietary restriction by enhancing mitochondrial activity in Drosophila. Cell 139(1):149-60.

Reviewer #2

This is a highly interesting and timely study of the mRNA folding strength, apparently the most comprehensive and technically solid to date. I have no doubts that it will be of great relevance for the journal readership. Scientifically, it is virtually impeccable and very convincing. In particular, the authors took great care to control for possible biases in their calculations.

*We are very happy that the reviewer found our study relevant and scientifically valid.*

Unfortunately there is one technical problem that necessitates a revision: the structure of the figures. They are overloaded with detail and insufficiently explained. Upon trying to comprehend them for a few days I am giving up.

*In addition to the specific answers below, note that we rearranged the figures (and in some cases changed the division of panels into figures) to improve readability and ensure figures appear in the order they are introduced. The figure and section numbers refer to the revised manuscript.*

Let us start with figure 2a. It is supposed to demonstrate that the short initial regions are less structured as the random control and thus would have negative dLFE values and hence colored blue. I spent several days looking at the printed version of Figure 1 and could not see anything blue on the left end. It then occured to me to look at the same figure in the PDF file and that is how I found that, indeed, the left end tends to be blue, but the blue stripes are so narrow that they cannot be seen in print. Why are there different numbers of stripes? E.g., for Opisthokonta there are four stripes on the left and two on the right while for Terrabacteria there are three on the left and five on the right. Why is that and what is that supposed to mean?

*We updated this figure to increase clarity. We now plot only the regions within 150nt of the start and stop codons, so the weak folding should be clearly visible. We also added a box identifying the different elements shown, as well as a brief explanation (in the caption of Figure 3 and in section 6.2 under “Taxon characteristic profiles chart”) of the method used to group the species into clusters of similar profiles (which determines the number of “groups” shown for each taxon).*

I cannot comprehend the PC analysis shown in Figure 2b. I do not know what the abbreviations here and elswehre mean (Spun, Dser - apparently these are organism names, by where is the explanation?) and the message of this figure is not clear to me. Figure 2B is not discussed in the text at all, the only sentence I could find is "The division of the profile into the three regions described here is also apparent when the data is analyzed in an unsupervised manner via Principal Components Analysis". That does not explain anything.

*This panel (which is now in Figure 3B) is meant to present common types of ΔLFE profiles, highlight the variation and consistency found, and also introduce this type of PCA plot which is used in other figures. We now explain this better in the figure caption.*

*We added a brief explanation of the key points about this analysis (PCA), i.e., that it arranges elements on a 2D surface with similar species (in terms of their ΔLFE profiles) receiving nearby positions. This allows the reader to see at a glance which types of ΔLFE profiles were found and how other properties (e.g. codon usage bias, GC-content, temperature) are related to the ΔLFE profile. It is not crucial to "understand” the statistical/mathematical theory behind the two Principal Components (and they don’t have an intuitive meaning); they simply provide a suitable 2D arrangement of the ΔLFE profiles. It is not trivial that two dimensions are sufficient to describe most of the differences between the profiles at each end of the CDS (49.7%+40% at the beginning and 74.1%+12.4% at end). This means two parameters are needed to roughly describe each ΔLFE profile at either end of the coding sequence, and this is what we meant in the sentence you quoted. We rephrased this to make our point more clearly. In addition, we* *added a note explaining that the short species names (appearing on the PCA plots) are expanded in Supplementary Table S3.*

In Figure 1 there are abbreviations A1, A2, B1, B2, and B which are not explained at all (I figured that these are probably different hypotheses being tested). GLC and CUB are explained in the methods, but should also be explained in the legend. ENc' is not explained at all.,

*We removed some identifiers that did not serve to increase clarity, and referenced the relevant section in the methods where the different null models are explained. We added a note that ENc’ is a measure of CUB, and it is discussed in detail in the text (section 3.3).*

In Figure 3A the results for the two models 1 and 2 are vastly different. Why? WHere is this discussed and what are the implications?

*We added a discussion of this in the text (section 3.1). The two models differ in how they define the regions of weak folding at the edges of the CDS – model 1 requires significantly weaker than expected (positive) folding, while model 2 only requires the folding to be significantly weaker than in the mid-CDS region. Both models are arguably appropriate, since they are applied to the collection of genes within any genome, which may include an unknown fraction of genes with no significant selection on folding strength. Since model 1 is stricter, fewer organisms match it.*

I do not know what the arrows in Figure 4E mean and I could not find any discussion of this figure in the text. What does it say?

*We removed the arrows (PCA loading vectors) from all figures for the sake of simplicity. This panel shows that low-GC species tend to have a specific ΔLFE profile, since they appear close together in both panels. Looking at this region in the plots showing the matching ΔLFE profiles on the same axes (in the insets here or in Figure 3B), you can see that this is the region of weak ΔLFE.*

Figure 5. What is MINE? I managed to find the explanation for MIC in the methods. What does this mean: CDS start + 100 - 300? What does the tilted red line close to the zero line mean? Upon some consideration I figured that this is probably the linear model, because paremeters are also in red in the upper right corner. But then, what does the green color mean?

*We removed a few distracting details from the figure (including the word "MINE" and other details mentioned by the reviewer), and improved the labels and explanation. For the linear model, the connection between the graphic representation and the numeric results is now indicated with a connecting line, in addition to the red color, which, as you inferred, is used for all elements of the linear model. The green color shows parameters of MIC, a measure of non­linear dependency between variables. We also improved the figure caption to include all these explanations.*

On all PC graphs, the loadings of individual PCs are shown (e.g. Figure 5B, PC1 has 80.3%), but I could not find any interpretation what the meaning of these first two PCs could be.

*The Principal Components themselves don’t usually allow an intuitive interpretation, other than that they lay out the elements (i.e. the ΔLFE profiles) on a plane with similar elements placed nearby and dissimilar elements placed farther apart. The amount of variation explained by each PC (shown in percent next to it) indicates how well this plot (using 2 components) represents the data and the relative importance of each component.*

Do not even ask me how to read Figure 9 - no idea.

*We revised the presentation of this figure and hopefully it is clear now. It simply summarizes the results of the regression analysis for different traits (columns) in different taxons (rows) - for each combination there are two bars, corresponding to the correlation strength based on the GLS and OLS models. The aim of the figure is to show which traits have consistent effects, which traits have effects only in specific taxons, and which traits are not significantly correlated with ΔLFE at all.*

I also do not like the fact that the first figure to mentioned in the text is Figure 3a***.*** *This was also fixed in the current draft.*

In summary: this is a scientifically excellent study, but please make figures less busy, perhaps split them in better understandable items, and explain their meaning clearly in the text.

*Thank you for your many helpful suggestions as to how to improve the figures. We have spent much effort to thoroughly update them and took your input into full consideration. We hope that you find the current version of the paper worthy.*

**Second round of review**

**Reviewer 1**

The manuscript has been much improved. There are still a few things that needs to be done to solidify the conclusions.

If the position-specific secondary structure is indeed maintained by selection for translation efficiency (or accuracy), then highly expressed genes are expected to experience stronger selection than lowly expressed genes, so the former should exhibit a stronger pattern than the latter (and the latter should exhibit a stronger pattern than pseudogenes). This was shown in for several bacterial species (Figures 4-5 in Xia 2019), especially for E. coli where 306 pseudogenes are clearly annotated in the type genome. This has also been shown in Staphylococcus phages (Fig. 4 in Prabhakaran et al. 2015). A similar pattern is shown in the baker's yeast where the frequency of nucleotide A dramatically increases in sequences immediately upstream of the start codon and all other nucleotides decreases in frequency (Figure 2 in Xia et al. 2011) (Note that a stretch of A nucleotides cannot form secondary structure). This pattern is much stronger in highly expressed genes than in lowly expressed genes (Figure 3 in Xia et al. 2011).

There is perhaps no need to contrast highly expressed and lowly expressed genes for all species, but can be done for a few representative multicellular eukaryotic species.

Translation efficiency can be characterized by I_TE (Xia 2015) which extends CAI to accommodate the effect of background mutation bias (i.e., CAI is a special case of  I_TE when there is no background nucleotide bias). I_TE is implemented in DAMBE that you have cited, together with compiled codon usage tables for highly and lowly expressed genes in many bacterial and unicellular eukaryotes. A simple compilation is simply to read a GenBank genome into DAMBE to extract all CDS sequences. Choose all genes encoding ribosomal proteins (optionally adding whatever other proteins that one knows to be highly expressed). Their codon usage is of the highly expressed genes. Codon usage of the rest of the genes may be considered as background.

In bacterial species, you should take into consideration of the confounding effect when a stop codon and a start codon are next to each other. In other words, the sequences flanking a stop codon are at the same time flanking a start codon. As mentioned in Xia (2019), there are many bacterial genes with CDS start overlapping CDS end of the upstream gene in the configuration of UAAUG (where UAA is the stop codon of the upstream gene and AUG is the start codon of the downstream gene) and AUGA (where AUG is the start codon of the downstream gene and UGA is the stop codon of the upstream gene). To avoid this confounding effect, his Figures 4-5 were generated from non-overlapping genes with an intergenic sequence of at least 50 nt. You may consider doing the same.

'This "CDS-wide" randomization preserves the encoded proteins sequence ...". This is not clear to me. Is the permutation done only within synonymous codons? For example, a GGA codon at position i is swapped with a GGG codon at position j? If a GGA codon at position i is swapped with a GCG codon at position j, then the protein sequence is not preserved. By the way, either change "proteins" to "protein" or delete "sequence".

For bacterial species, it might be better to separate genes into those with well-positioned SD and those without because those genes whose mRNAs do not have a good SD/anti-SD interaction are in greater need to reduce secondary structure (Wei and Xia 2019).

You may briefly mention 1) the problem of poorly defined operon structure in bacterial species and 2) the frustrating pattern of transcripts starting at different sites of the same gene with different 5'UTRs (Some genes have transcripts starting directly with AUG but we do not know this from the genome). This is optional, but the authors should be aware of this in their future research.

Please think hard on the organization of the figures. Ideally one should be able to know the meaning of the figures without referring to the text. Show the figures to some of your colleagues and see if they can get the essence out of it (My guess is that they cannot).

There are multiple "Error! Refernece source not found" in the text. Please fix.

Prabhakaran R, Chithambaram S, Xia X. 2015. Escherichia coli and Staphylococcus phages: effect of translation initiation efficiency on differential codon adaptation mediated by virulent and temperate lifestyles. J Gen Virol, 96:1169-1179.
Wei Y, Xia X. 2019. Unique Shine-Dalgarno sequences in Cyanobacteria and chloroplasts reveal evolutionary differences in their translation initiation. Genome Biology and Evolution, DOI: 10.1093/gbe/evz227.
Xia X. 2015. A Major Controversy in Codon-Anticodon Adaptation Resolved by a New Codon Usage Index. Genetics, 199:573-579.
Xia X. 2019. Optimizing Phage Translation Initiation. OBM Genetics, 3:16.
Xia X, MacKay V, Yao X, Wu J, Miura F, Ito T, Morris DR. 2011. Translation Initiation: A Regulatory Role for Poly(A) Tracts in Front of the AUG Codon in Saccharomyces cerevisiae. Genetics, 189:469-478.

**Reviewer 2**

The authors have adequately addressed my concerns.

**Authors Response**

**Point-by-point responses to the reviewers’ comments:**

Reviewer #1: The manuscript has been much improved. There are still a few things that needs to be done to solidify the conclusions.

*We are happy that the reviewer feels that our manuscript has been much improved and appreciate the suggestions and additional controls. Below we answer his/her additional points.*

If the position-specific secondary structure is indeed maintained by selection for translation efficiency (or accuracy), then highly expressed genes are expected to experience stronger selection than lowly expressed genes, so the former should exhibit a stronger pattern than the latter (and the latter should exhibit a stronger pattern than pseudogenes). This was shown in for several bacterial species (Figures 4-5 in Xia 2019), especially for E. coli where 306 pseudogenes are clearly annotated in the type genome. This has also been shown in Staphylococcus phages (Fig. 4 in Prabhakaran et al. 2015). A similar pattern is shown in the baker's yeast where the frequency of nucleotide A dramatically increases in sequences immediately upstream of the start codon and all other nucleotides decreases in frequency (Figure 2 in Xia et al. 2011) (Note that a stretch of A nucleotides cannot form secondary structure). This pattern is much stronger in highly expressed genes than in lowly expressed genes (Figure 3 in Xia et al. 2011).

There is perhaps no need to contrast highly expressed and lowly expressed genes for all species, but can be done for a few representative multicellular eukaryotic species.

*We agree that having stronger folding bias in highly expressed genes is certainly a reasonable expectation, but there are additional factors worth considering when looking at a wide range of species with diverse ecological and evolutionary backgrounds. In species that are not highly optimized for translation efficiency and/or accuracy, there might not be a wide enough difference between highly and lowly expressed genes. More generally, this correlation is expected to be much stronger in unicellular organisms with larger effective population sizes (and thus higher selection efficiency) and not in multicellular organisms. It is also clear that this effect should be weaker than the overall genomic bias we reported due to the influence of noise in gene expression measurements. In addition, the groups of genes in this case are smaller than in genomic case and thus the statistical power is lower.*

*Nevertheless, following the reviewer comment, we performed the following analysis (Supplementary Figure S5): We compared ∆LFE strength in highly expressed and lowly expressed genes, based on RNA-seq and protein abundance data. We included 29 species with RNA-seq data and 12 species with protein-abundance data. The observed differences between the gene populations are small but in most species statistically significant. In general, the results support the hypothesis that highly expressed genes have stronger ∆LFE.*

*In addition to the points above, we should note that the strength of ∆LFE in different genes may be affected not just by the expression levels but by additional factors, and indeed we found wide variation in folding biases between genes that is not explained by transcript abundance, protein abundance or codon bias.*

Translation efficiency can be characterized by I_TE (Xia 2015) which extends CAI to accommodate the effect of background mutation bias (i.e., CAI is a special case of I_TE when there is no background nucleotide bias). I_TE is implemented in DAMBE that you have cited, together with compiled codon usage tables for highly and lowly expressed genes in many bacterial and unicellular eukaryotes. A simple compilation is simply to read a GenBank genome into DAMBE to extract all CDS sequences. Choose all genes encoding ribosomal proteins (optionally adding whatever other proteins that one knows to be highly expressed). Their codon usage is of the highly expressed genes. Codon usage of the rest of the genes may be considered as background.

*Following the reviewer comment, we now show a comparison of ∆LFE for genes with high and low I_TE for 17 species (Supplementary Figure S5, cyan column). The results for genes with highly adapted codons generally agree with those for genes encoding highly abundant proteins.*

In bacterial species, you should take into consideration of the confounding effect when a stop codon and a start codon are next to each other. In other words, the sequences flanking a stop codon are at the same time flanking a start codon. As mentioned in Xia (2019), there are many bacterial genes with CDS start overlapping CDS end of the upstream gene in the configuration of UAAUG (where UAA is the stop codon of the upstream gene and AUG is the start codon of the downstream gene) and AUGA (where AUG is the start codon of the downstream gene and UGA is the stop codon of the upstream gene). To avoid this confounding effect, his Figures 4-5 were generated from non-overlapping genes with an intergenic sequence of at least 50 nt. You may consider doing the same.

*Following the reviewer comment, we compared the ∆LFE for genes with different intergenic distances for 17 species (Supplementary Figure S16). Our results support the conclusion that there is indeed a statistically significant difference between ∆LFE in genes with short or overlapping 5’ intergenic distances and those with long intergenic distances in many bacterial species. This phenomenon may contribute to the overall folding bias we observe, and we now mention this possibility in the discussion. From our point of view it is one source of folding bias and is not a confounding effect (since the sum of all factors contributing to direct selection on folding strength is what we intended to measure). We should also note that the observed folding strength within the first few windows (i.e. the region directly affected by overlap with another CDS) is only slightly different in genes with overlapping (so the overlap does not directly disrupt the overall folding). More generally, this does not appear to be the only factor explaining the folding bias found in either group of genes, and both groups generally show roughly similar levels of ∆LFE (Supplementary Figure S16 panels B,C) in most cases.*

'This "CDS-wide" randomization preserves the encoded proteins sequence ...". This is not clear to me. Is the permutation done only within synonymous codons? For example, a GGA codon at position i is swapped with a GGG codon at position j? If a GGA codon at position i is swapped with a GCG codon at position j, then the protein sequence is not preserved. By the way, either change "proteins" to "protein" or delete "sequence".

*Thank you for your remarks. We updated the explanation to clarify this point. As you correctly inferred, the permutation is done only between synonymous codons found within a given CDS in order to guarantee that both the translated sequence and codon frequencies are maintained for each coding sequence after randomization. We also fixed the typo you mentioned.*

For bacterial species, it might be better to separate genes into those with well-positioned SD and those without because those genes whose mRNAs do not have a good SD/anti-SD interaction are in greater need to reduce secondary structure (Wei and Xia 2019).

*Following the reviewer comment, we added a comparison of ∆LFE in genes with different anti-SD hybridization energy for 17 species (Supplementary Figure S16). Our results for Synechocystic sp. for the beginning of the CDS agree with those reported for the 5’-UTR in (Wei and Xia 2019), with weaker folding immediately downstream of the start codon in genes with poor SD binding. Further downstream, the region of stronger folding (“transition peak” or region B) is even stronger in the same genes. Interestingly, these observations do not appear to be universal and we could not confirm them in some of the other species we tested. This subject warrants further study in the future. We should note that although this phenomenon may certainly contribute to the folding bias measured in our study in some species, it does not appear to adversely affect our analysis.*

You may briefly mention 1) the problem of poorly defined operon structure in bacterial species and 2) the frustrating pattern of transcripts starting at different sites of the same gene with different 5'UTRs (Some genes have transcripts starting directly with AUG but we do not know this from the genome). This is optional, but the authors should be aware of this in their future research.

*Thank you for your suggestion. We don’t believe these issues drastically affect the results of this study, but we agree these are important subjects that should be investigated further. Now we mention these research topics in the discussion section.*

Please think hard on the organization of the figures. Ideally one should be able to know the meaning of the figures without referring to the text. Show the figures to some of your colleagues and see if they can get the essence out of it (My guess is that they cannot).

*Thank you for your suggestion. We made several improvements to the figures to make the general meaning more intuitively clear.*

There are multiple "Error! Refernece source not found" in the text. Please fix.

*We are sorry for this issue and have fixed it in the new manuscript.*

Prabhakaran R, Chithambaram S, Xia X. 2015. Escherichia coli and Staphylococcus phages: effect of translation initiation efficiency on differential codon adaptation mediated by virulent and temperate lifestyles. J Gen Virol, 96:1169-1179.

Wei Y, Xia X. 2019. Unique Shine-Dalgarno sequences in Cyanobacteria and chloroplasts reveal evolutionary differences in their translation initiation. Genome Biology and Evolution, DOI: 10.1093/gbe/evz227.

Xia X. 2015. A Major Controversy in Codon-Anticodon Adaptation Resolved by a New Codon Usage Index. Genetics, 199:573-579.

Xia X. 2019. Optimizing Phage Translation Initiation. OBM Genetics, 3:16.

Xia X, MacKay V, Yao X, Wu J, Miura F, Ito T, Morris DR. 2011. Translation Initiation: A Regulatory Role for Poly(A) Tracts in Front of the AUG Codon in Saccharomyces cerevisiae. Genetics, 189:469-478.

Reviewer #2: ===

The authors have adequately addressed my concerns.

*We are happy that the reviewer found our paper worthy.*

**Third round of review**

**Reviewer 1**

The manuscript has been substantially improved. One ambiguity that I wish to see clarified concerning the selection regime. The authors observed variation in local folding energy (LFE), and compared the pattern against what is expected from the null hypothesis of no selection. However, it is not clear what selection is operating to generate the pattern. One would expect that LFE should be minimal at some location but there should be an optimal (i.e., not too low or too high) at some other locations. It would be nice for the authors to be more explicit about what is the selection that operates to generate the observed pattern. For example, many yeast genes have short poly(A) tracts immediately upstream of the start codon. One may hypothesis that the short poly(A) tracts reduces local secondary structure stability and facilitates the scanning of small subunit ribosome to find the start codon. This would predict that highly expressed genes would have the short poly(A) more frequently than lowly expressed genes, which is true (Xia et al. 2011 Genetics). However, it turns out that what is favored by selection is not the reduced secondary structure stability, because poly(U), which also reduces local secondary structure, is not used more frequently in highly expressed genes than lowly expressed genes. A search of literature showed that short poly(A) tracts upstream of the start codon bind to translation initiation factors to facilitate translation initiation, but longer poly(A) tracts would bind poly(A)-binding protein (PABP). If such binding occurs upstream of the start codon (PABP binds to long poly(A) tract more tightly than translation initiation factors), then the binding would inhibit translation initiation. This would predict that the poly(A) tract upstream of the start codon should not be longer than 12 (the binding size of yeast PABP), which is indeed true (Xia et al. 2011 Genetics). Furthermore, yeast genes with long poly(A) (>= 12) at 5’UTR tend to have significantly fewer ribosomes in their mRNA than those with short poly(A) (Xia et al. 2011 Genetics). In this sequence of findings, one can see that selection is operating in favor of short poly(A) tract, but against long poly(A),  in 5’UTR immediately upstream of the start codon (Yeast PABP gene is an exception because it uses a poly(A) in its 5’UTR for autoregulation). Note that the initial suspected importance/relevance of secondary structure was not substantiated.

I am more comfortable with this type of focused studies, and hope that the authors would spend more effort along this line. However, I am not demanding this as mandatory for this manuscript.

**Authors Response**

**Point-by-point responses to the reviewers’ comments:**

Reviewer #2:

The manuscript has been substantially improved.

*We are happy that the reviewer thinks that our manuscript has been substantially improved.*

One ambiguity that I wish to see clarified concerning the selection regime. The authors observed variation in local folding energy (LFE), and compared the pattern against what is expected from the null hypothesis of no selection. However, it is not clear what selection is operating to generate the pattern. One would expect that LFE should be minimal at some location but there should be an optimal (i.e., not too low or too high) at some other locations. It would be nice for the authors to be more explicit about what is the selection that operates to generate the observed pattern.

*We agree with the reviewer that there is still much to explore in term of the exact mechanisms related to the exact mechanisms related to selection for LFE. We describe one component of the overall selection, acting to increase or decrease folding energy at different regions of the CDS. This of course may be gene specific but we report patterns that can be seen at the genomic level (and we may miss some gene specific patterns). We discuss these issues in the discussion section.*

For example, many yeast genes have short poly(A) tracts immediately upstream of the start codon. One may hypothesis that the short poly(A) tracts reduces local secondary structure stability and facilitates the scanning of small subunit ribosome to find the start codon. This would predict that highly expressed genes would have the short poly(A) more frequently than lowly expressed genes, which is true (Xia et al. 2011 Genetics). However, it turns out that what is favored by selection is not the reduced secondary structure stability, because poly(U), which also reduces local secondary structure, is not used more frequently in highly expressed genes than lowly expressed genes. A search of literature showed that short poly(A) tracts upstream of the start codon bind to translation initiation factors to facilitate translation initiation, but longer poly(A) tracts would bind poly(A)-binding protein (PABP). If such binding occurs upstream of the start codon (PABP binds to long poly(A) tract more tightly than translation initiation factors), then the binding would inhibit translation initiation. This would predict that the poly(A) tract upstream of the start codon should not be longer than 12 (the binding size of yeast PABP), which is indeed true (Xia et al. 2011 Genetics). Furthermore, yeast genes with long poly(A) (>= 12) at 5'UTR tend to have significantly fewer ribosomes in their mRNA than those with short poly(A) (Xia et al. 2011 Genetics). In this sequence of findings, one can see that selection is operating in favor of short poly(A) tract, but against long poly(A), in 5'UTR immediately upstream of the start codon (Yeast PABP gene is an exception because it uses a poly(A) in its 5'UTR for autoregulation). Note that the initial suspected importance/relevance of secondary structure was not substantiated.

*Thank you for the interesting example and topic. We agree that related research topic should be further explored. Note however that we studied in our paper only the coding region and not the UTRs and our null model controls for the content at the UTR (thus, UTR features should not affect the conclusions) and for local patterns at the AA levels and basic features of the coding sequence such as GC content and codon frequencies We try to make this clear in the manuscript.*

I am more comfortable with this type of focused studies, and hope that the authors would spend more effort along this line. However, I am not demanding this as mandatory for this manuscript.

*Thank you. We agree that this is an important direction in which this research should proceed in the future.*
